# Boosting the Enantioselectivity of Conjugate Borylation of α,β‐Disubstituted Cyclobutenones with Monooxides of Chiral *C*_2_‐Symmetric Bis(phosphine) Ligands

**DOI:** 10.1002/chem.202202163

**Published:** 2022-09-01

**Authors:** Ming Cui, Zhi‐Yuan Zhao, Martin Oestreich

**Affiliations:** ^1^ Institut für Chemie Technische Universität Berlin Strasse des 17. Juni 115 10623 Berlin Germany

**Keywords:** asymmetric catalysis, conjugate borylation, copper catalysis, cyclobutenones, hemilabile ligands

## Abstract

Chiral bis(phosphine) monooxides (BPMOs) derived from *C*_2_‐symmetric bis(phosphines) have been found to induce superior levels of enantioselection in copper‐catalyzed conjugate borylation of α,β‐disubstituted cyclobutenones. More precisely, enantiomeric excesses as well as chemical yields are exceedingly high with (*R*,*R*)‐Bozphos as the chiral ligand while these values are low with parent (*R*,*R*)‐Me‐Duphos. A similar yet less pronounced effect was seen in the corresponding 1,6‐addition to *para*‐quinone methides.

## Graphical abstract

**A chiral bis(phosphine) monooxide (BPMO)** derived from a *C*_2_‐symmetric bis(phosphine) ligand does magic to the copper‐catalyzed conjugate borylation of α,β‐disubstituted cyclobutenones. Yields and levels of stereoselection, especially enantioselectivity, are exceedingly high (see scheme).

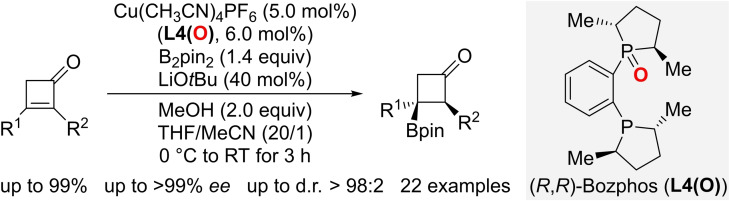

## Introduction

Chiral bis(phosphines) undoubtly belong to the most important ligands for asymmetric metal‐catalyzed reactions.^[1]^ By virtue of their ready availability, bis(phosphine) dioxides have also emerged as effective Lewis base catalysts in organocatalysis^[2]^ and, more recently, as chiral ligands in transition‐metal catalysis.^[3]^ In contrast, chiral bis(phosphine) monooxides (BPMOs) have been largely neglected, perhaps due to lack of demand when reactions can proceed satisfactorily by employing the parent bis(phosphines). However, these mixed phosphine‐phosphine oxide ligands are typical representatives of the class of hemilabile ligands containing a soft and a hard donor, that is P and P=O, respectively.^[4]^ BPMOs can act as either bi‐ or monodentate ligands in transition‐metal complexes and can therefore lead to reaction outcomes different from those obtained with bis(phosphines). There had been scattered examples in the literature[^5^, ^6^] before Charette and co‐workers identified BPMOs as superior ligands in enantioselective copper‐catalyzed (conjugate) addition reactions.[^7^, ^8^] Another prominent application of chiral BPMOs is their use in intermolecular asymmetric Heck reactions,^[9]^ and there are further relevant examples in palladium[^10^, ^11^] as well as rhodium[^12^, ^13^] catalysis.

**Scheme 1:**
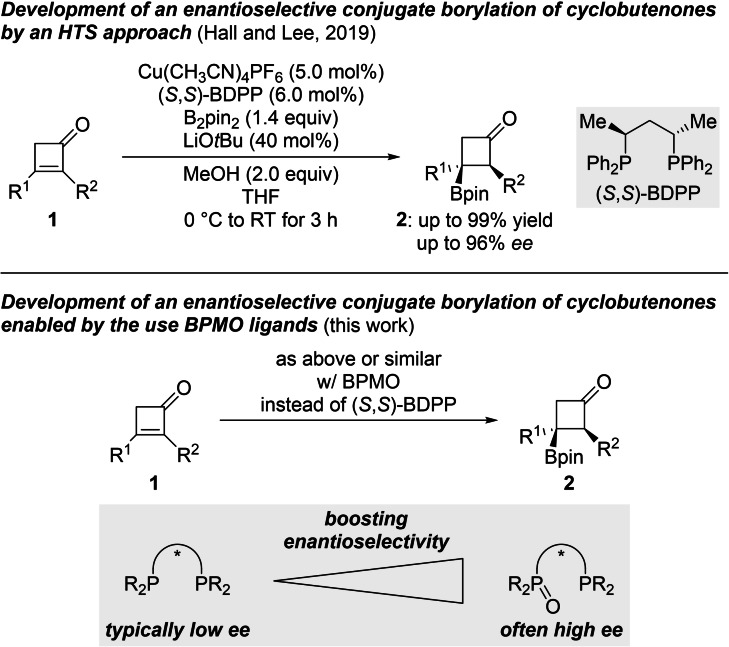
Enantioselective conjugate borylation of α,β‐disubstituted cyclobutenones under copper catalysis with chiral bis(phosphines) and mixed phosphine‐phosphine oxides as ligands, respectively. pin=pinacolato.

Copper‐catalyzed 1,4‐addition of boron nucleophiles is a powerful tool for the preparation of enantioenriched boron‐containing compounds.^[14]^ Bidentate bis(phosphine) ligands have been widely used in this field yet there have been no examples with the corresponding BPMOs reported. Three years ago, Hall, Lee, and co‐workers tackled the challenge of enantioselective conjugate borylation to α,β‐disubstituted cyclobutenones **1** to access synthetically versatile, borylated cyclobutanones **2** (Scheme 1, top).[^15^, ^16^, ^17^] For this, the authors utilized a chiral ligand high‐throughput screening (HTS) platform, and (*S*,*S*)‐BDPP was found to be the optimal ligand for achieving good yield as well as enantioselectivity ranging from 79 to 96 % *ee*, and diastereomeric ratios were high throughout. Among a library of 118 ligands, more than half were ineffective for yield and enantioinduction. Inspired by Charette's seminal work^[8]^ and on the basis of our own experience with BPMOs,^[9b]^ we began investigating copper‐catalyzed 1,4‐addition reactions of boron and silicon nucleophiles.^[18]^ We describe here an enantioselective conjugate borylation to cyclobutenones **1** where the use of BPMO ligands boosts the enantioinduction to afford tertiary borylated cyclobutanones **2** in good yields and with excellent enantiomeric excesses (Scheme 1, bottom).

## Results and Discussion

**Scheme 2:**
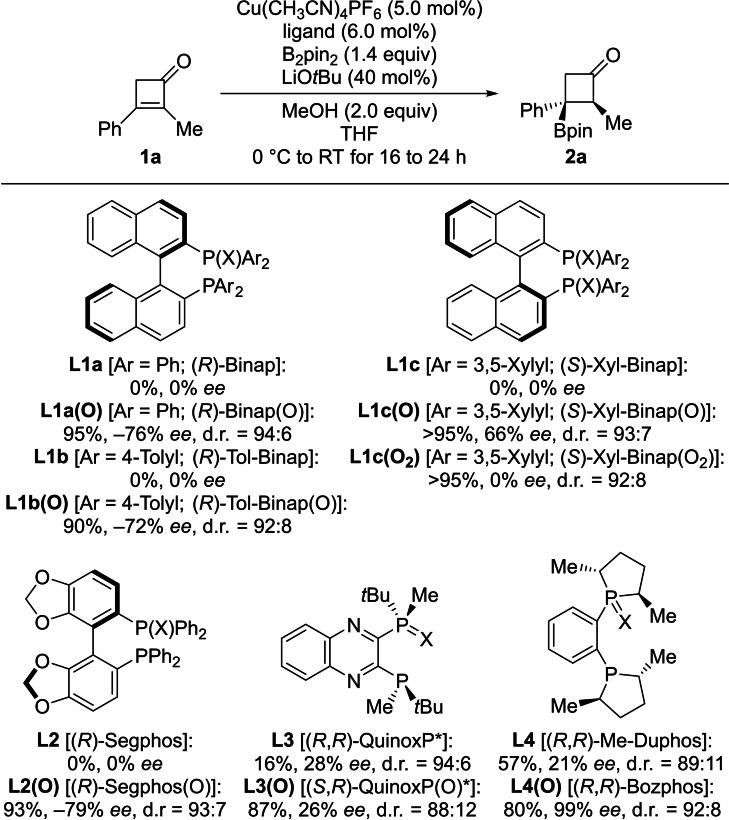
Ligand identification. All reactions were performed on a 0.10 mmol scale. Yields were determined by ^1^H NMR spectroscopic analysis of the crude reaction mixture by the addition of CH_2_Br_2_ as an internal standard. Enantiomeric excesses were determined by HPLC analysis on a chiral stationary phase after isolation of the major diastereomer. Diastereomeric ratios were estimated by ^1^H NMR spectroscopy. X=lone pair or O.

**Figure 1:**
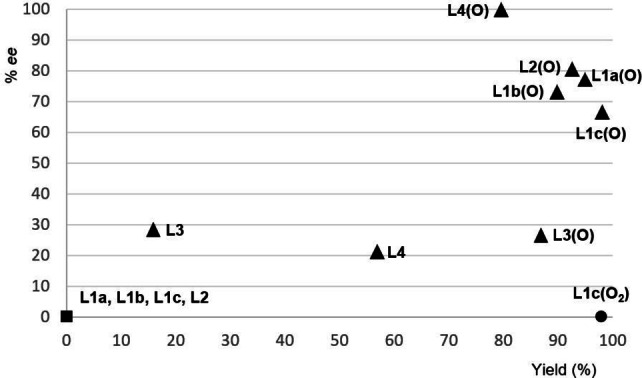
Overview of the comparison between the bis(phosphines) and the corresponding BPMOs. ▪ for bis(phosphine); ▴ for bis(phosphine) monooxide; • for bis(phosphine) dioxide.

Adopting the reaction setup employed by Hall and Lee (see Scheme 1, top), we began our investigation with comparing various chiral *C*_2_‐symmetric bis(phosphine) ligands and their monooxide counterparts (Scheme 2 and Figure 1). The borylation of cyclobutenone **1 a** to give the β‐borylated cyclobutanone **2 a** was chosen as the model reaction. No conversion of acceptor **1 a** was seen with (*R*)‐Binap (**L1 a**) and its congeners **L1 b**,**c**. In contrast, high yields and moderate enantioselection were obtained for **2 a** with BPMOs **L1 a(O)**–**c(O)**. A similar outcome was found for another pair of biaryl‐based ligands **L2** and **L2(O)**. Both BPMO **L3(O)** ^[10]^ and parent QuinoxP* (**L3**) induced low enantioselection but the yield was again markedly higher with the hemilabile ligand. The use of Bozphos **L4(O)**, introduced by Charette to enantioselective copper catalysis with BPMOs,^[8]^ brought the breakthrough in this reaction, affording the borylated product **2 a** in 80 % yield and 99 % *ee*. Confirming the previously observed trend, yield and enantioselectivity were significantly lower with the corresponding bis(phosphine) Me‐Duphos (**L4**). It must be mentioned that, to allow for a direct comparison with Hall's and Li's findings, our attempts to prepare the monooxide of (*S*,*S*)‐BDPP failed. The absolute configuration of the β‐borylated cyclobutanone **2 a** was assigned as 2*S*,3*R* by comparing its specific rotation value and HPLC traces with reported data.^[15]^

The diastereoselectivity in favor of the *trans* product can be rationalized by protolysis of the enolate intermediate from the less hindered face away from the bulky boryl group. Resubjection of a sample of **2 a** with d.r.=86 : 14 to the above reaction conditions using Bozphos **L4(O)** as the ligand only resulted in a minor change to d.r.=88 : 12 after 3 h. We then probed various reaction parameters regarding their influence on the diastereoselectivity (Table 1). The solvent had an effect but the ee value was lowest when the diastereomeric ratio was highest (entries 1–4). As epimerization did not occur over the course of time, the reaction time could be shortened to 3 h (entry 5). With MeCN being optimal in terms of diastereocontrol (entry 3) and THF for excellent enantiomeric excess (entry 5), we tested mixtures of these solvents with THF/MeCN 20/1 emerging as the best compromise (entries 6 and 7). Changing the proton source from methanol to isopropanol gave no improvement (92 % ee and d.r.=94 : 6). Other copper sources such as CuCl and CuBr provided lower stereoselectivities (not shown).

**Table 1:** Further reaction optimization with (*R*,*R*)‐Bozphos [**L4(O)**] as the chiral ligand.

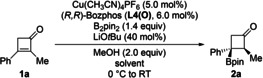				
Entry	Variation	Yield [%]^[a]^	*ee* [%]^[b]^	d.r^[c]^
1	none	80	99	92 : 8
2	Toluene instead of THF	89	92	89 : 11
3	MeCN instead of THF	>95	86	>95 : 5
4	1,4‐Dioxane instead of THF	85	97	95 : 5
5	3 h instead of 16 to 24 h	93	99	92 : 8
6	THF/MeCN (10/1) for 3 h	92	98	94 : 6
7	THF/MeCN (20/1) for 3 h	>95	98	>95 : 5

[a] Determined by ^1^H NMR spectroscopic analysis of the crude reaction mixture by the addition of CH_2_Br_2_ as an internal standard. [b] Determined by HPLC analysis on a chiral stationary phase after isolation of the major diastereomer. [c] Estimated by ^1^H NMR spectroscopy.

**Scheme 3:**
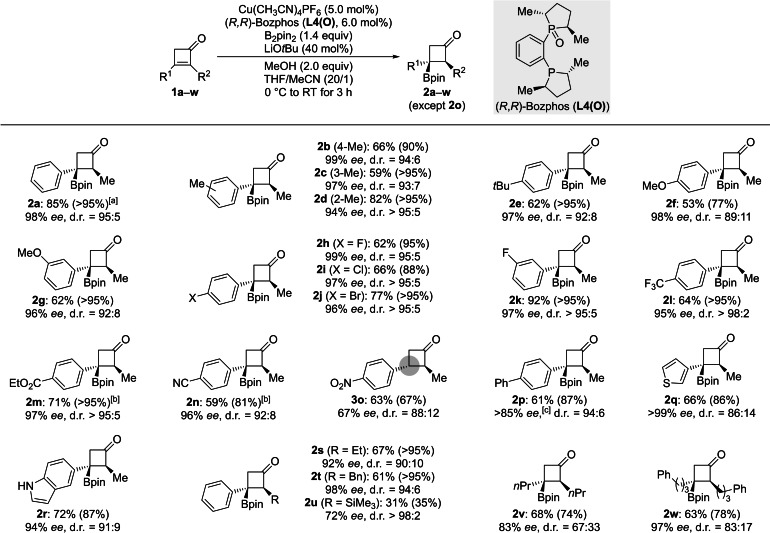
Scope of the enantioselective, conjugate borylation of cyclobutenones. All reactions were performed on a 0.20 mmol scale. Yields are isolated after flash chromatography on silica gel; yields in parentheses were determined by ^1^H NMR spectroscopic analysis of the crude reaction mixture by the addition of CH_2_Br_2_ as an internal standard. Enantiomeric excesses were determined by HPLC analysis on chiral stationary phases. Diastereomeric rations were estimated by ^1^H NMR spectroscopy. [a] 73 %, 98 % *ee*, d.r.=94 : 6 on a 2.0 mmol scale. [b] Run for 1 h. [c] No baseline separation in the HPLC trace.

With the optimized protocol in hand, we investigated the reaction scope (Scheme 3). (*R*,*R*)‐Bozphos (**L4(O)**) generally provided a higher level of enantioselection than the bis(phosphine) ligand (*S*,*S*)‐BDPP employed by Hall and Lee.^[15]^ A methyl group at the aryl substituent as in the three regioisomers **1 b**–**d** only had a minor effect, and **2 b**–**d** were obtained in enantiomeric excess of 94 % or higher as well as good diastereomeric ratios. The diastereoselectivity was lower with more electron‐donating groups such as a *tert*‐butyl as in **1 e** and methoxy groups as in **1 f** and **1 g**; *ee* values for **2 e**–**g** remained above 95 % throughout. Cyclobutenones **1 h**–**k** bearing halogenated aryl substituents reacted with consistently high stereocontrol. Acceptors with electron‐deficient aryl groups at the β carbon atom had furnished lower levels of enantioselectivity in Hall's and Lee's report,^[15]^ and **1 n** and **1 o** with a cyano and a nitro group in the *para* position, respectively, were not included in their survey. With BPMO **L4(O)** as the chiral ligand, these electron‐withdrawing groups as well as trifluoromethyl (as in **1 l**) and an ester (as in **1 m**) were tolerated, giving the desired products **2 l**–**n** yet not **2 o** with excellent enantioselectivities. In the case of nitro‐substituted **1 o**, protodeborylated **3 o** was isolated with a moderate enantiomeric excess (gray circle). In turn, that protodeborylation could be prevented for **1 m**→**2m** and **1 n**→**2 n** when the reaction time was reduced to 1 h; **2 n** had primarily undergone loss of the boryl group under Hall's and Lee's reaction conditions.^[15]^ Also, biphenyl‐ and heteroaryl‐substituted substrates yielded the borylated cyclobutanones with high enantioselection (**1 p**‐**r**→**2 p**–**r**). Changing the α‐substituent of the cyclobutenone from methyl to ethyl and benzyl, respectively, was well tolerated (**1 s**,**t**→**2 s**,**t**) while a bulkier trimethylsilyl group gave the adduct in a low yield and with a moderate *ee* value as a single diastereomer (**1 u**→**2 u**). Gratifyingly, α,β‐dialkylsubstituted cyclobutenones led to lower diastereomeric ratios but the enantioselectivity remained high (**1 v**,**w**→**2 v**,**w**).

**Scheme 4:**
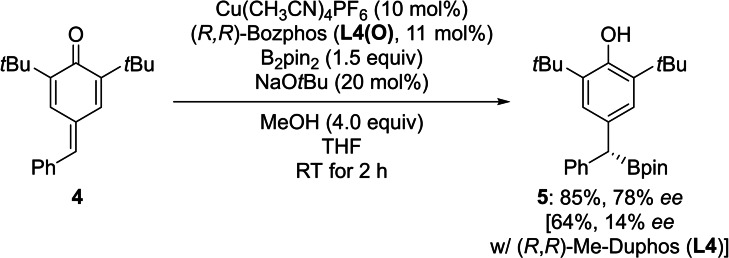
1,6‐Borylation of a *para*‐quinone methide. Both reactions were performed on a 0.10 mmol scale. Yields are isolated after flash chromatography on silica gel. Enantiomeric excesses were determined by HPLC analysis on a chiral stationary phase.

To probe the advantage of BPMOs in conjugate borylation reactions further, we conducted a copper‐catalyzed 1,6‐borylation of a *para*‐quinone methide^[19]^ (**4**→**5**, Scheme 4). Liao^[20a]^ and Tortosa^[20b]^ had independently disclosed effective protocols for this transformation employing either a chiral sulfoxide‐phosphine ligand (SOP) or a bis(phosphine) ligand. When (*R*,*R*)‐Bozphos (**L4(O)**) was used as a ligand, a good yield and a moderate enantioselectivity were obtained. For comparison, (*R*,*R*)‐Me‐DuPhos (**L4**) gave the benzhydryl boronate **5** in moderate yield with very low enantioselectivity (cf. Ref. [20b]). Other acceptors such as cyclohexanone did not give any enhanced enantioselectivity with BPMOs as ligands (not shown).

## Conclusion

We demonstrated here that the beneficial use of chiral bis(phosphine) monooxides (BPMOs) in copper catalysis goes beyond Charette's key discoveries.[^7^, ^8^] The reported enantioselective copper‐catalyzed conjugate borylation of cyclobutenones with (*R*,*R*)‐Bozphos complements the results of Hall and Lee with the bis(phosphine) ligand (*S*,*S*)‐BDPP.^[15]^ By this, highly enantioenriched borylated cyclobutanone building blocks have been obtained, and their synthetic versatility has already been illustrated.^[15]^ We believe that BPMOs ought to be included into routine ligand screenings, especially because these can be introduced as impurities of bis(phosphines).

## Conflict of interest

The authors declare no conflict of interest.

1

## Supporting information

As a service to our authors and readers, this journal provides supporting information supplied by the authors. Such materials are peer reviewed and may be re‐organized for online delivery, but are not copy‐edited or typeset. Technical support issues arising from supporting information (other than missing files) should be addressed to the authors.

Supporting Information

## Data Availability

The data that support the findings of this study are available from the corresponding author upon reasonable request.
