# Supplementary material for: Boosting the Enantioselectivity of Conjugate Borylation of α,β‐Disubstituted Cyclobutenones with Monooxides of Chiral C 2‐Symmetric Bis(phosphine) Ligands
Source: Chemistry. 2022 Sep 1;28(62):e202202163. doi: 10.1002/chem.202202163 (PMC9804767; doi:10.1002/chem.202202163)
Supplement: Supplementary file 1 — Supporting Information [file CHEM-28-0-s001.pdf]

Chemistry–A European Journal

Supporting Information

Boosting the Enantioselectivity of Conjugate Borylation of α,β-Disubstituted Cyclobutenones with Monooxides of Chiral C_2-Symmetric Bis(phosphine) Ligands

Ming Cui, Zhi-Yuan Zhao, and Martin Oestreich*

Table of Contents

**1**	**General Information**	**S3**
**2**	**Characterization of the α,β-Disubstituted Cyclobutenones**	**S4**
**3**	**Optimization Study of Enantioselective Conjugate Borylation of Cyclobutenones**	**S13**
**4**	**Experimental Details for Enantioselective Conjugate Borylation of Cyclobutenones**	**S15**
4.1	General Procedures for Enantioselectivie Conjugate Borylation of Cyclobutenones (**GP 1**)	S15
4.2	General Procedures for Synthesis of Racemic Cyclobutylboronates (**GP 2**)	S16
4.3	2.00 mmol Scale Reaction	S17
**5**	**Experimental Details for the 1,6-Borylation of a *para*-Quinone Methide**	**S34**
**6**	**HPLC Traces**	**S36**
**7**	**NMR Spectra**	**S64**
**8**	**References**	**S127**

1 General Information

All reactions were performed in flame-dried glassware using conventional Schlenk techniques under a static pressure of nitrogen unless stated otherwise. Liquids and solutions were transferred with syringes. Copper salts were purchased from commercial suppliers and used as received. *rac*-Binap(O) **L5**, (*R*)-Binap(O) **L1a(O)**, (*R*)-Tol-Binap(O) **L1b(O)**, (*S*)-Xyl-Binap(O) **L1c(O)**, (*S*)-xyl-Binap dioxide **L1c(O$_2$)**, (*R*)-Segphos(O) **L2(O)** and (*R*,*R*)-Quinoxp(O) **L3(O)** were prepared according to the literature.[S1] Other ligands were obtained from commercial suppliers. α,β-Disubstituted cyclobutenones **1** were prepared according to literature.[S2] All solvents (toluene, Et_2O, and THF) were dried and purified following standard procedures. Dry 1,4-dioxane and acetonitrile were directly purchased from commercial suppliers. Technical grade solvents for extraction or chromatography (cyclohexane, ethyl acetate) were distilled prior to use. Analytical thin layer chromatography (TLC) was performed on *ALUGRAM*® Xtra SIL G/UV_{254} TLC-Sheets by *Macherey-Nagel*. Flash column chromatography was performed on silica gel 60 (40–63 µm, 230–400 mesh, ASTM) by Grace using the indicated solvents. Automatic column chromatography was performed on an Isolera One™ (*Biotage*) using KP Sil columns (25, 50 g). ^{1}H, ^{11}B and ^{13}C NMR spectra were recorded in $CDCl_3$ on Bruker AV400 or AV500 instruments. Chemical shifts are reported in parts per million (ppm) and are referenced to the residual solvent resonance as the internal standard ($CHCl_3$: δ = 7.26 ppm for ^{1}H NMR and $CDCl_3$: δ = 77.16 ppm for ^{13}C NMR). Chemical shifts are reported to 0.01 ppm for ^{1}H NMR, and to 0.1 ppm for ^{13}C NMR, ^{19}F NMR and ^{11}B NMR spectra. Peaks that are within 0.01 ppm for ^{1}H NMR or 0.1 ppm for ^{13}C NMR but are still distinguishable are reported to 0.001 ppm and 0.01 ppm, respectively. Data are reported as follows: chemical shift, multiplicity (br = broad signal, s = singlet, d = doublet, t = triplet, q = quartet, sept = septet, m = multiplet, m_c = centrosymmetric multiplet), coupling constants (Hz), and integration. Infrared (IR) spectra were recorded on an *Agilent Technologies Cary* 630 FT-IR spectrometer equipped with an ATR unit and the signals are reported in wave-numbers (cm^{-1}). Melting points (m.p.) were determined with a *Stuart Scientific* SMP20 melting point apparatus and are not corrected. High resolution mass spectrometry (HRMS) analysis was performed by the Analytical Facility at the *Institut für Chemie, Technische Universität Berlin*. Optical rotations were measured on a *Schmidt & Haensch* Polatronic H532 polarimeter with $[\alpha]_\lambda$ values reported in 10^{-1} (° cm^2 g^{-1}); concentration c is in g/100 mL and λ as indicated. Enantiomeric excesses were determined by analytical high performance liquid chromatography (HPLC) analysis on an *Agilent Technologies* 1290 Infinity instrument with a chiral stationary phase using a *Daicel* Chiralcel IC-column, or OD-H column, or IB-column (*n*-heptane/ *i*-PrOH mixtures as solvent).

2 Characterization of the α,β-Disubstituted Cyclobutenones (1a–w)

2.1 2-Methyl-3-phenylcyclobut-2-en-1-one (1a)

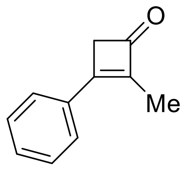

1a
$C_{11}H_{10}O$
M = 158.20 g/mol

^{1}H NMR (400 MHz, $CDCl_3$, 298 K) δ = 7.61–7.56 (m, 2H), 7.53–7.45 (m, 3H), 3.45 (q, *J* = 2.3 Hz, 2H), 2.00 (t, *J* = 2.3 Hz, 3H) ppm. **^{13}C NMR** (101 MHz, $CDCl_3$, 298 K) δ = 190.0, 163.5, 141.0, 132.8, 131.0, 129.2, 129.0, 48.2, 9.4 ppm. The NMR spectroscopic data are in accordance with those reported.[S2a]

2.2 2-Methyl-3-(*p*-tolyl)cyclobut-2-en-1-one (1b)

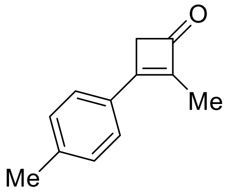

1b
$C_{12}H_{12}O$
M = 172.23 g/mol

^{1}H NMR (400 MHz, $CDCl_3$, 298 K) δ = 7.48 (d, *J* = 8.2 Hz, 2H), 7.30 (d, *J* = 7.9 Hz, 2H), 3.42 (q, *J* = 2.3 Hz, 2H), 2.43 (s, 3H), 1.98 (t, *J* = 2.3 Hz, 3H) ppm. **^{13}C NMR** (101 MHz, $CDCl_3$, 298 K) δ = 190.1, 163.6, 141.7, 140.0, 130.2, 129.7, 29.2, 48.1, 21.7, 9.4 ppm. The NMR spectroscopic data are in accordance with those reported.[S2a]

2.3 2-Methyl-3-(*m*-tolyl)cyclobut-2-en-1-one (1c)

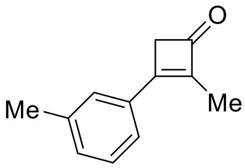

1c
$C_{12}H_{12}O$
M = 172.23 g/mol

^{1}H NMR (400 MHz, $CDCl_3$, 298 K) δ = 7.41–7.35 (m, 3H), 7.30–7.26 (m, 1H), 3.42 (q, *J* = 2.3 Hz, 2H), 2.42 (s, 3H), 1.99 (t, *J* = 2.3 Hz, 3H) ppm. **^{13}C NMR** (101 MHz, $CDCl_3$, 298 K) δ =

190.1, 163.8, 140.8, 138.7, 132.8, 131.8, 129.8, 128.9, 126.4, 48.2, 21.4, 9.4 ppm. The NMR spectroscopic data are in accordance with those reported.[2a]

2.4 2-Methyl-3-(*o*-tolyl)cyclobut-2-en-1-one (1d)

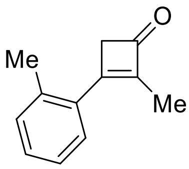

1d
$C_{12}H_{12}O$
M = 172.23 g/mol

^{1}H NMR (500 MHz, $CDCl_3$, 298 K) δ = 7.48–7.43 (m, 1H), 7.36–7.31 (m, 1H), 7.30–7.26 (m, 2H), 3.59 (q, *J* = 2.4 Hz, 2H), 2.45 (s, 3H), 1.89 (t, *J* = 2.4 Hz, 3H) ppm. **^{13}C NMR** (126 MHz, $CDCl_3$, 298 K) δ = 190.6, 165.8, 143.6, 137.0, 132.4, 131.4, 130.4, 129.1, 126.0, 51.7, 21.1, 9.5 ppm. The NMR spectroscopic data are in accordance with those reported.[2a]

2.5 3-(4-(*tert*-Butyl)phenyl)-2-methylcyclobut-2-en-1-one (1e)

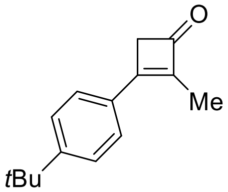

1e
$C_{15}H_{18}O$
M = 214.31 g/mol

^{1}H NMR (500 MHz, $CDCl_3$, 298 K) δ = 7.56–7.51 (m, 4H), 3.43 (q, *J* = 2.3 Hz, 2H), 1.99 (t, *J* = 2.3 Hz, 3H), 1.36 (s, 9H) ppm. **^{13}C NMR** (126 MHz, $CDCl_3$, 298 K) δ = 190.1, 163.5, 154.7, 140.1, 130.1, 129.1, 125.9, 48.1, 35.1, 31.1, 9.3 ppm. The NMR spectroscopic data are in accordance with those reported.[2a]

2.6 3-(4-Methoxyphenyl)-2-methylcyclobut-2-en-1-one (1f)

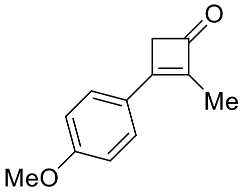

1f
$C_{12}H_{12}O_2$
M = 188.08 g/mol

Prepared according to the reported procedure with *N*,*N*-dimethylpropionamide (0.76 mL, 6.90 mmol, 1.00 equiv), 1-ethynyl-4-methoxybenzene (1.00 g, 7.60 mmol, 1.10 equiv), 2-

fluoropyridine (0.71 mL, 8.30 mmol, 1.20 equiv), Tf_2O (1.3 mL, 7.60 mmol, 1.10 equiv) and KO*t*Bu (232 mg, 0.30 equiv).[S2a] The crude material was purified by automatic column chromatography using Isolera™ One with cyclohexane/ethyl acetate as eluents (40:1 → 10:1) to provide a yellow slurry (119 mg, 9.2% yield over three steps). **IR** (ATR): $\tilde{\nu}$ = 2904, 2834, 1733, 1598, 1507, 1423, 1341, 1250, 1169, 1024, 972, 827, 769, 708 cm^{-1}. **^{1}H NMR** (500 MHz, $CDCl_3$, 298 K) δ = 7.55 (d, *J* = 8.8 Hz, 2H), 7.01 (d, *J* = 8.8 Hz, 2H), 3.41 (q, *J* = 2.3 Hz, 2H), 1.97 (t, *J* = 2.3 Hz, 3H) ppm. **^{13}C NMR** (101 MHz, $CDCl_3$, 298 K) δ = 189.9, 163.3, 161.8, 138.3, 131.2, 125.8, 114.5, 55.5, 48.0, 9.3 ppm. **HRMS** (APCI) exact mass for $[M+H]^+$ $C_{12}H_{13}O_2^+$: calculated 189.0910, found 189.0904.

2.7 3-(3-Methoxyphenyl)-2-methylcyclobut-2-en-1-one (1g)

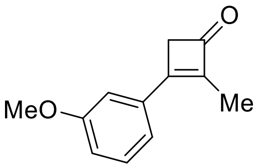

1g
$C_{12}H_{12}O_2$
M = 188.08 g/mol

^{1}H NMR (500 MHz, $CDCl_3$, 298 K) δ = 7.45–7.40 (m, 1H), 7.22–7.17 (m, 1H), 7.10–7.07 (m, 1H), 7.05–7.00 (m, 1H), 3.45 (q, *J* = 2.3 Hz, 2H), 2.00 (t, *J* = 2.3 Hz, 3H) ppm. **^{13}C NMR** (126 MHz, $CDCl_3$, 298 K) δ = 190.0, 163.4, 159.8, 141.3, 134.0, 130.0, 121.7, 116.5, 114.4, 55.4, 48.3, 9.3 ppm. The NMR spectroscopic data are in accordance with those reported.[S2a]

2.8 3-(4-Fluorophenyl)-2-methylcyclobut-2-en-1-one (1h)

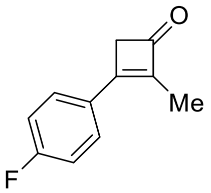

1h
$C_{11}H_9FO$
M = 176.06 g/mol

Prepared according to the reported procedure with *N*,*N*-dimethylpropionamide (0.91 mL, 8.28 mmol, 1.00 equiv), 1-ethynyl-4-fluorobenzene (1.20 g, 10.0 mmol, 1.20 equiv), 2-fluoropyridine (0.86 mL, 9.94 mmol, 1.20 equiv), Tf_2O (1.56 mL, 9.11 mmol, 1.10 equiv) and KO*t*Bu (279 mg, 0.300 equiv).[S2a] The crude material was purified by automatic column chromatography using cyclohexane/ethyl acetate as eluents (20:1 → 10:1) to provide a yellow solid (671 mg, 46% yield over three steps). **M.p.** = 82–83°C (cyclohexane). **IR** (ATR): $\tilde{\nu}$ = 3473, 3057, 2914, 1739, 1616, 1501, 1371, 1340, 1223, 1157, 1084, 1008, 970, 837 cm^{-1}. **^{1}H NMR** (500 MHz, $CDCl_3$, 298 K) δ = 7.61–7.55 (m, 2H), 7.21–7.16 (m, 2H), 3.43 (q, *J* = 2.4 Hz, 2H), 1.98 (t, *J* = 2.4 Hz,

3H) ppm. **^{13}C NMR** (126 MHz, $CDCl_3$, 298 K) δ = 189.5, 164.0 (d, *J* = 253.7 Hz), 162.2, 140.5, 131.3 (d, *J* = 8.8 Hz), 129.3 (d, *J* = 3.0 Hz), 116.3 (d, *J* = 22.0 Hz), 48.3, 9.3 ppm. **^{19}F NMR** (471 MHZ, $CDCl_3$, 298 K) δ = –107.3 ppm. **HRMS** (APCI) exact mass for $[M+H]^+$ $C_{11}H_{10}FO^+$: calculated 177.0710, found 177.0704.

2.9 3-(4-Chlorophenyl)-2-methylcyclobut-2-en-1-one (1i)

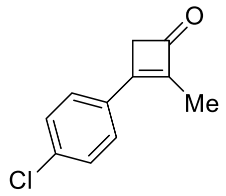

1i
$C_{11}H_9ClO$
M = 192.03 g/mol

^{1}H NMR (400 MHz, $CDCl_3$, 298 K) δ = 7.53–7.43 (m, 4H), 3.44 (q, *J* = 2.3 Hz, 2H), 1.99 (t, *J* = 2.3 Hz, 3H) ppm. **^{13}C NMR** (101 MHz, $CDCl_3$, 298 K) δ = 189.5, 162.0, 141.6, 137.1, 131.3, 130.3, 129.3, 48.3, 8.4 ppm. The NMR spectroscopic data are in accordance with those reported.[S2a]

2.10 3-(4-Bromophenyl)-2-methylcyclobut-2-en-1-one (1j)

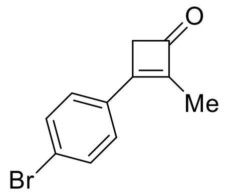

1j
$C_{11}H_9BrO$
M = 235.98 g/mol

^{1}H NMR (400 MHz, $CDCl_3$, 298 K) δ = 7.63 (d, *J* = 8.4 Hz, 2H), 7.43 (d, *J* = 8.4 Hz, 2H), 3.44 (q, *J* = 2.2 Hz, 2H), 1.98 (t, *J* = 2.2 Hz, 3H) ppm. **^{13}C NMR** (101 MHz, $CDCl_3$, 298 K) δ = 189.5, 162.1, 141.8, 132.3, 131.6, 130.4, 125.5, 48.3, 9.5 ppm. The NMR spectroscopic data are in accordance with those reported.[S2a]

2.11 3-(3-Fluorophenyl)-2-methylcyclobut-2-en-1-one (1k)

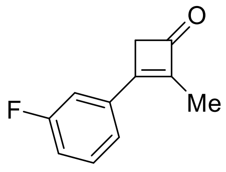

1k
$C_{11}H_9FO$
M = 176.06 g/mol

^{1}H NMR (400 MHz, $CDCl_3$, 298 K) δ = 7.52–7.43 (m, 1H), 7.39–7.34 (m, 1H), 7.28–7.23 (m, 1H), 7.20–7.13 (m, 1H), 3.45 (q, *J* = 2.3 Hz, 2H), 2.00 (t, *J* = 2.2 Hz, 3H) ppm. **^{13}C NMR** (101 MHz, $CDCl_3$, 298 K) δ = 189.7, 162.8 (d, *J* = 247.5 Hz), 162.1 (d, *J* = 2.4 Hz), 142.4, 134.7 (d, *J* = 7.7 Hz), 130.6 (d, *J* = 8.0 Hz), 124.9 (d, *J* = 2.9 Hz), 117.9 (d, *J* = 21.5 Hz), 115.6 (d, *J* = 21.7 Hz), 48.4, 9.4 ppm. The NMR spectroscopic data are in accordance with those reported.[S2a]

2.12 2-Methyl-3-(4-(trifluoromethyl)phenyl)cyclobut-2-en-1-one (1l)

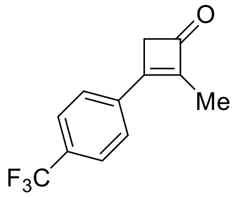

1l
$C_{12}H_9F_3O$
M = 226.06 g/mol

^{1}H NMR (400 MHz, $CDCl_3$, 298 K) δ = 7.75 (d, *J* = 8.2 Hz, 2H), 7.69 (d, *J* = 8.0 Hz, 2H), 3.51 (q, *J* = 2.4 Hz, 2H), 2.04 (t, *J* = 2.3 Hz, 3H) ppm. **^{13}C NMR** (101 MHz, $CDCl_3$, 298 K) δ = 189.6, 161.5, 143.5, 135.8,132.2 (q, *J* = 32.0 Hz), 129.2, 125.9 (q, *J* = 3.8 Hz), 123.7 (q, *J* = 272.3 Hz), 48.5, 8.5 ppm. The NMR spectroscopic data are in accordance with those reported.[S2a]

2.13 Ethyl 4-(2-methyl-3-oxocyclobut-1-en-1-yl)benzoate (1m)

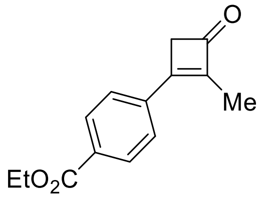

1m
$C_{14}H_{14}O_3$
M = 230.09 g/mol

Prepared according to the reported procedure with *N*,*N*-dimethylpropionamide (0.64 mL, 5.70 mmol, 1.00 equiv), 4-ethynylbenzoic acid methyl ester (1.00 g, 6.20 mmol, 1.10 equiv), 2-fluoropyridine (0.65 mL, 7.50 mmol, 1.20 equiv), Tf_2O (1.10 mL, 6.20 mmol, 1.10 equiv) and KO*t*Bu (192 mg, 0.300 equiv)) with EtOH for the final step.[S2a] The crude material was purified by automatic column chromatography using Isolera™ One with cyclohexane/ethyl acetate as eluents (20:1 → 5:1) to provide a light yellow solid (472 mg, 36% yield over three steps). **M.p.** = 135–136°C (cyclohexane). **IR** (ATR): $\tilde{\nu}$ = 2981, 2913, 1737, 1705, 1617, 1477, 1268, 1176, 1102, 1010, 853, 770, 693 cm^{-1}. **^{1}H NMR** (500 MHz, $CDCl_3$, 298 K) δ = 8.16 (d, *J* = 8.6 Hz, 2H), 7.64 (d, *J* = 8.4 Hz, 2H), 4.42 (q, *J* = 7.1 Hz, 2H), 3.50 (q, *J* = 2.3 Hz, 2H), 2.04 (t, *J* = 2.3 Hz, 3H), 1.42 (t, *J* = 7.1 Hz, 3H) ppm. **^{13}C NMR** (126 MHz, $CDCl_3$, 298 K) δ = 189.7, 165.8,

162.0, 143.3, 136.4, 132.1, 130.0, 128.8, 61.4, 48.4, 14.3, 9.5 ppm. **HRMS** (APCI) exact mass for $[M+H]^+$ $C_{14}H_{15}O_3^+$: calculated 231.1016, found 231.1016.

2.14 4-(2-Methyl-3-oxocyclobut-1-en-1-yl)benzonitrile (1n)

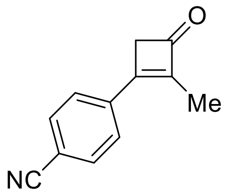

1n
$C_{12}H_9NO$
M = 183.07 g/mol

^{1}H NMR (400 MHz, $CDCl_3$, 298 K) δ = 7.78 (d, *J* = 8.1 Hz, 2H), 7.66 (d, *J* = 8.4 Hz, 2H), 3.50 (q, *J* = 2.3 Hz, 2H), 2.04 (t, *J* = 2.3 Hz, 3H) ppm. **^{13}C NMR** (101 MHz, $CDCl_3$, 298 K) δ = 189.1, 160.7, 144.7, 136.5, 132.7, 129.3, 118.2, 113.9, 48.5, 9.6 ppm. The NMR spectroscopic data are in accordance with those reported.[S2a]

2.15 2-Methyl-3-(4-nitrophenyl)cyclobut-2-en-1-one (1o)

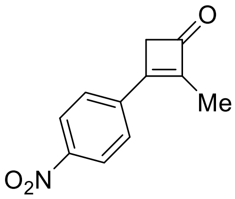

1o
$C_{11}H_9NO_3$
M = 203.06 g/mol

^{1}H NMR (500 MHz, $CDCl_3$, 298 K) δ = 8.35 (d, *J* = 8.9 Hz, 2H), 7.73 (d, *J* = 8.8 Hz, 2H), 3.55 (q, *J* = 2.3 Hz, 2H), 2.07 (t, *J* = 2.3 Hz, 3H) ppm. **^{13}C NMR** (126 MHz, $CDCl_3$, 298 K) δ = 189.0, 160.2, 148.5, 145.3, 138.2, 129.6, 124.2, 48.7, 9.6 ppm. The NMR spectroscopic data are in accordance with those reported.[S2a]

2.16 3-([1,1'-Biphenyl]-4-yl)-2-methylcyclobut-2-en-1-one (1p)

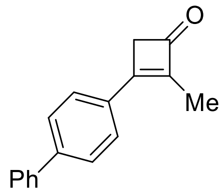

1p
$C_{17}H_{14}O$
M = 234.30 g/mol

^{1}H NMR (500 MHz, $CDCl_3$, 298 K) δ = 7.56–7.51 (m, 4H), 3.43 (q, *J* = 2.3 Hz, 2H), 1.99 (t, *J* = 2.3 Hz, 3H), 1.36 (s, 9H) ppm. **^{13}C NMR** (126 MHz, $CDCl_3$, 298 K) δ = 189.9, 163.1, 143.7, 140.9, 139.9, 131.7, 129.7, 129.0, 128.2, 127.6, 127.2, 48.2, 9.5 ppm. The NMR spectroscopic data are in accordance with those reported.[S2a]

2.17 2-Methyl-3-(thiophen-3-yl)cyclobut-2-en-1-one (1q)

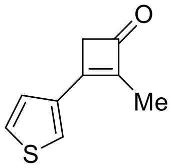

1q
C_9H_8OS
M = 164.03 g/mol

Prepared according to the reported procedure[S2a] with *N*,*N*-dimethylpropionamide (0.76 mL, 6.90 mmol, 1.00 equiv), 3-ethynylthiophene (0.820 g, 7.60 mmol, 1.10 equiv), 2-fluoropyridine (0.72 mL, 8.30 mmol, 1.20 equiv), Tf_2O (1.3 mL, 7.60 mmol, 1.10 equiv) and KO*t*Bu (232 mg, 0.300 equiv). The crude material was purified by automatic column chromatography using Isolera™ One with cyclohexane/ethyl acetate as eluents (40:1 → 10:1) to provide a yellow solid (124 mg, 11% yield over three steps). **M.p.** = 58–59°C (cyclohexane). **IR** (ATR): $\tilde{\nu}$ = 3094, 2918, 1733, 1608, 1510, 1413, 1302, 1222, 1066, 1002, 857, 801 cm^{-1}. **^{1}H NMR** (500 MHz, $CDCl_3$, 298 K) δ = 7.63–7.60 (m, 1H), 7.48–7.45 (m, 1H), 7.41–7.38 (m, 1H), 3.43 (q, *J* = 2.3 Hz, 2H), 1.93 (t, *J* = 2.3 Hz, 3H) ppm. **^{13}C NMR** (126 MHz, $CDCl_3$, 298 K) δ = 189.8, 157.8, 139.2, 135.3, 129.4, 127.4, 126.7, 48.8, 9.0 ppm. **HRMS** (APCI) exact mass for $[M+H]^+$ $C_9H_9OS^+$: calculated 165.0369, found 165.0364.

2.18 3-(1*H*-Indol-5-yl)-2-methylcyclobut-2-en-1-one (1r)

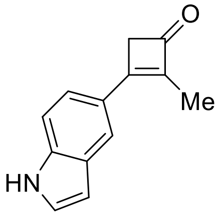

1r
$C_{13}H_{11}NO$
M = 197.08 g/mol

Prepared according to a reported procedure with *N*,*N*-dimethylpropionamide (0.33 mL, 3.00 mmol, 1.10 equiv),), 5-ethynyl-1-tosyl-1H-indole (840 mg, 2.80 mmol, 1.00 equiv),[S3] 2-fluoropyridine (0.30 mL, 3.40 mmol, 1.20 equiv), Tf_2O (0.53 mL, 3.10 mmol, 1.10 equiv) and KO*t*Bu (110 mg, 0.350 equiv).[S2a] The crude material was purified by automatic column chromatography using Isolera™ One with cyclohexane/ethyl acetate as eluents (10:1 → 3:1)

to provide a light yellow solid (78 mg, 14% yield over three steps). **M.p.** = 171–172°C (cyclohexane). **IR** (ATR): $\tilde{\nu}$ = 3217, 3145, 1726, 1595, 1375, 1343, 1013, 890, 799, 768, 736 cm^{-1}. **^{1}H NMR** (400 MHz, $CDCl_3$, 298 K) δ = 8.42 (s, 1H), 7.88 (s, 1H), 7.56–7.51 (m, 2H), 7.34–7.29 (m, 1H), 6.70–6.64 (m, 1H), 3.50 (q, *J* = 2.4 Hz, 2H), 2.05 (t, *J* = 2.2 Hz, 3H) ppm. **^{13}C NMR** (101 MHz, $CDCl_3$, 298 K) δ = 190.2, 165.4, 137.7, 137.0, 128.1, 125.7, 125.3, 123.4, 123.3, 111.6, 104.0, 48.2, 9.4 ppm. **HRMS** (APCI) exact mass for $[M+H]^+$ $C_{13}H_{12}NO^+$: calculated 198.0913, found 198.0909.

2.19 2-Ethyl-3-phenylcyclobut-2-en-1-one (1s)

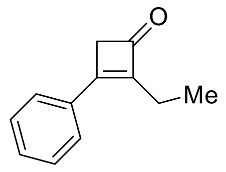

1s
$C_{12}H_{12}O$
M = 172.09 g/mol

^{1}H NMR (500 MHz, $CDCl_3$, 298 K) δ = 7.60–7.55 (m, 2H), 7.52–7.44 (m, 3H), 3.44 (t, *J* = 1.7 Hz, 2H), 2.47 (qt, *J* = 2.9 Hz, 2H), 1.23 (t, *J* = 7.7 Hz, 3H) ppm. **^{13}C NMR** (126 MHz, $CDCl_3$, 298 K) δ = 189.9, 162.4, 146.7, 132.8, 130.9, 129.1, 129.0, 48.3, 18.2, 11.4 ppm. The NMR spectroscopic data are in accordance with those reported.[S2a]

2.20 2-Benzyl-3-phenylcyclobut-2-en-1-one (1t)

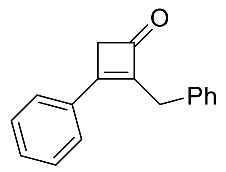

1t
$C_{17}H_{14}O$
M = 234.10 g/mol

^{1}H NMR (500 MHz, $CDCl_3$, 298 K) δ = 7.61–7.57 (m, 2H), 7.48–7.46 (m, 3H), 7.31–7.27 (m, 4H), 7.24–7.19 (m, 1H), 3.80 (t, *J* = 1.4 Hz, 2H), 3.54 (t, *J* = 1.4 Hz, 2H) ppm. **^{13}C NMR** (126 MHz, $CDCl_3$, 298 K) δ = 189.5, 164.1, 142.9, 137.0, 132.3, 131.3, 129.4, 129.0, 128.7, 128.4, 126.6, 48.5, 30.4 ppm. The NMR spectroscopic data are in accordance with those reported.[S2a]

2.21 3-Phenyl-2-(trimethylsilyl)cyclobut-2-en-1-one (1u)

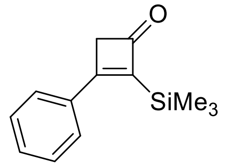

1u
$C_{13}H_{16}OSi$
M = 216.10 g/mol

^{1}H NMR (400 MHz, $CDCl_3$, 298 K) δ = 7.63–7.60 (m, 2H), 7.52–7.49 (m, 3H), 3.71 (s, 2H), 0.32 (s, 9H) ppm. **^{13}C NMR** (101 MHz, $CDCl_3$, 298 K) δ = 192.4, 178.0, 150.3, 132.6, 130.5, 130.0, 127.7, 53.3, 0.0 ppm. **^{1}H/^{29}Si HMQC NMR** (500/99 MHz, $CDCl_3$, 298 K, optimized for *J* = 7 Hz): δ = 0.32/–14.8 ppm. The NMR spectroscopic data are in accordance with those reported.[S2b]

2.22 2,3-Dipropylcyclobut-2-en-1-one (1v)

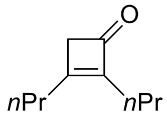

1v
$C_{10}H_{16}O$
M = 152.12 g/mol

^{1}H NMR (500 MHz, $CDCl_3$, 298 K) δ = 3.05 (s, 2H), 2.49 (t, *J* = 7.5 Hz, 2H), 2.01 (t, *J* = 7.5 Hz, 2H), 1.60 (q, *J* = 7.4 Hz, 2H), 1.49 (q, *J* =7.5 Hz, 2H), 0.96 (t, *J* = 7.4 Hz, 3H), 0.88 (t, *J* = 7.4 Hz, 3H) ppm. **^{13}C NMR** (126 MHz, $CDCl_3$, 298 K) δ = 190.2, 172.6, 148.2, 49.2, 31.6, 25.4, 20.7, 19.6, 14.1, 14.0 ppm. The NMR spectroscopic data are in accordance with those reported.[S2c]

2.23 2,3-Bis(3-phenylpropyl)cyclobut-2-en-1-one (1w)

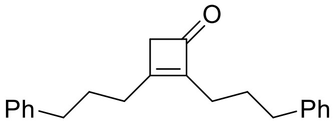

1w
$C_{22}H_{24}O$
M = 304.18 g/mol

^{1}H NMR (500 MHz, $CDCl_3$, 298 K) δ = 7.32–7.14 (m, 10H), 3.09–3.06 (m, 2H), 2.67 (t, *J* = 7.6 Hz, 2H), 2.61 (t, *J* = 7.6 Hz, 2H), 2.53 (t, *J* = 7.6 Hz, 2H), 2.08 (t, *J* = 7.6 Hz, 2H), 1.95–1.79 (m, 4H) ppm. **^{13}C NMR** (126 MHz, $CDCl_3$, 298 K) δ = 190.0, 172.3, 148.0, 141.7, 141.2, 128.5, 128.42, 128.36, 126.2, 125.9, 49.3, 35.7 (2C), 29.2, 28.7, 27.8, 23.2 ppm. The NMR spectroscopic data are in accordance with those reported.[S2a]

3 Optimization Study of Enantioselective Conjugate Borylation of Cyclobutenones

Table S1. Variation of the solvent.

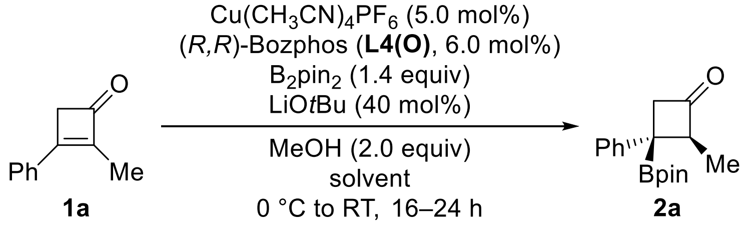

Entry	Solvent	Conversion	Yield (%)[a]	d.r.[b]	ee (%)[c]
1	Et_2O	100%	88	91:9	94
2	1,4-Dioxane	100%	85	>95:5	97
3	MeCN	99%	98	>95:5	86
4	Toluene	100%	89	89:11	92
5[d]	1,4-Dioxane	100%	97	>95:5	95
6[d]	THF	100%	93	92:8	99
7[d]	Dioxane/MeCN (10/1)	100%	85	94:6	98
8[d]	THF/MeCN (10/1)	100%	92	94:6	98
9[d]	THF/MeCN (20/1)	100%	>95	>95:5	98

All reactions were performed on a 0.10 mmol scale. [a] Determined by ^{1}H NMR spectroscopy of the crude reaction mixture by the addition of CH_2Br_2 as the internal standard. [b] Determined by the relative ^{1}H NMR peak height of the Me groups. [c] Determined by HPLC on chiral stationary phases after isolation of the major diastereomer. [d] The reaction was stirred for 3 hours.

Table S2. Variation of the base.

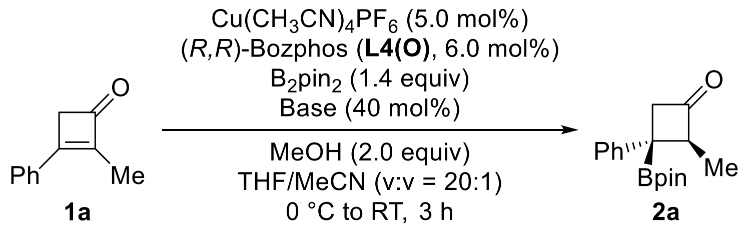

Entry	Base	Conversion	Yield (%)[a]	d.r.[b]	ee (%)[c]
1	NaOtBu	100%	>95	92:8	96
2	KOtBu	100%	>95	94:6	94
3	LiO*t*Bu	100%	>95	>95:5	98

All reactions were performed on a 0.10 mmol scale. [a] Determined by ^{1}H NMR spectroscopy of the crude reaction mixture by the addition of CH_2Br_2 as the internal standard. [b] Determined by the relative ^{1}H NMR peak height of the Me groups. [c] Determined by HPLC on chiral stationary phases after isolation of the major diastereomer.

Table S3. Variation of the proton source.

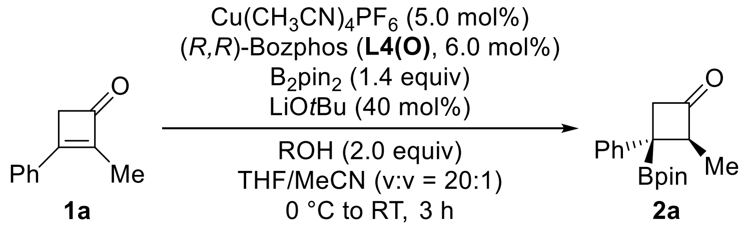

Entry	ROH	Conversion	Yield (%)[a]	d.r.[b]	ee (%)[c]
1	*i*PrOH	100%	90	94:6	92
2	*t*BuOH	100%	92	94:6	96
3	MeOH	100%	>95	>95:5	98

All reactions were performed on a 0.10 mmol scale. [a] Determined by ^{1}H NMR spectroscopy of the crude reaction mixture by the addition of CH_2Br_2 as the internal standard. [b] Determined by the relative ^{1}H NMR peak height of the Me groups. [c] Determined by HPLC on chiral stationary phases after isolation of the major diastereomer.

Table S4. Variation of the copper source.

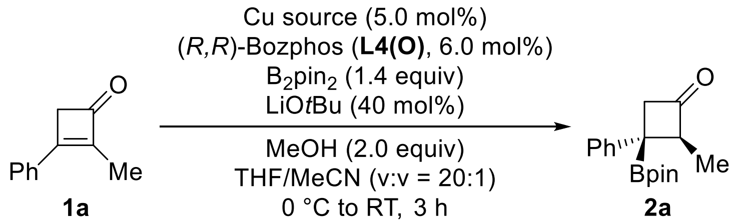

Entry	Cu source	Conversion	Yield (%)[a]	d.r.[b]	ee (%)[c]
1	CuCl	100%	>95	91:9	95
2	CuBr	100%	>95	92:8	84

All reactions were performed on a 0.10 mmol scale. [a] Determined by ^{1}H NMR spectroscopy of the crude reaction mixture by the addition of CH_2Br_2 as the internal standard. [b] Determined by the relative ^{1}H NMR peak height of the Me groups. [c] Determined by HPLC on chiral stationary phases after isolation of the major diastereomer.

4 Experimental Details for Enantioselective Conjugate Borylation of Cyclobutenones

4.1 General Procedures for Enantioselective Conjugate Borylation Cyclobutenones (GP1)

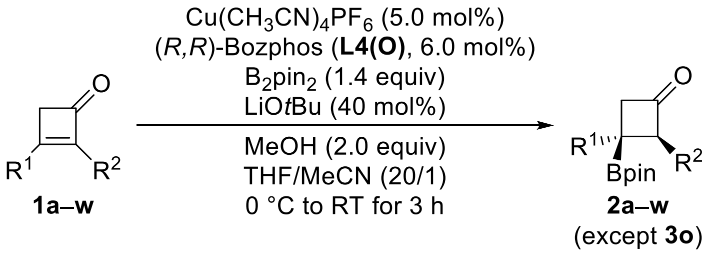

An oven-dried 10-mL Schlenk tube equipped with a magnetic stir bar was charged with $Cu(CH_3CN)_4PF_6$ (3.73 mg, 5.00 mol %), (*R*,*R*)-BozPhos (**L4(O)**, 3.87 mg, 6.00 mol %) and B_2pin_2 (71.1 mg, 0.280 mmol, 1.40 equiv). The tube was evacuated under high vacuum and backfilled with nitrogen gas (3 times). THF/MeCN (20/1, 1 mL) was added to the tube, and the resulting mixture was stirred under room temperature for 15 minutes. The mixture was then cooled to 0 °C, and LiO*t*Bu (2.2 M, 36.3 μL, 0.0800 mmol, 0.400 equiv) was added dropwise and then the mixture was stirred under 0 °C for 10 minutes. The corresponding cyclobutenones (**1**, 0.200 mmol, 1.00 equiv) were added under a static pressure of nitrogen gas, followed by dropwise addition of MeOH (16 μL, 2.00 equiv). The ice bath was subsequently removed and the reaction was stirred for 3 h at room temperature. After the indicated reaction time, the reaction mixture was filtered through a plug of silica and Celite, and the filter cake was washed with EtOAc (10 mL). The filtrate was concentrated under vaccuum and determined by ^{1}H NMR spectroscopy to obtain the NMR yield and diastereoselectivity with CH_2Br_2 (14.0 μL, 0.200 mmol, 1.0 equiv) as an internal standard. The diastereomeric ratio was determined by ^{1}H NMR spectroscopy of the crude reaction mixture by the relative peak height of the corresponding methyl protons which appear at 1.41 and 0.75 ppm for the major and minor diastereomers, respectively. For products **2s**, **2t**, **2u**, **2v** and **2w**, the diastereoselectivity was determined by the relative integration. The residue was purified by flash column chromatography on silica gel using the indicated mixture of cyclohexane and ethyl acetate.

4.2 General Procedures for Synthesis of Racemic Cyclobutylboronates (GP2)

Method A:

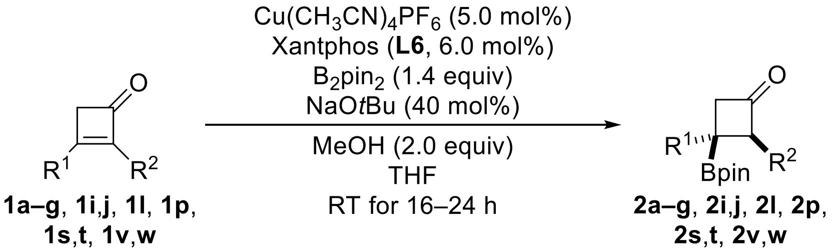

An oven-dried 10-mL Schlenk tube equipped with a magnetic stir bar was charged with $Cu(CH_3CN)_4PF_6$ (1.86 mg, 5.00 mol %), Xantphos (**L6**, 3.47 mg, 6.00 mol %), Na*Ot*Bu (1.92 mg, 0.200 equiv) and B_2pin_2 (35.5 mg, 0.140 mmol, 1.40 equiv). The tube was evacuated under high vacuum and backfilled with nitrogen gas (3 times). THF (1 mL) was added to the tube, and the resulting mixture was stirred under room temperature for 15 minutes. The corresponding cyclobutenones (**1a–g**, **1i**,**j**, **1l**, **1p**, **1s**,**t**, **1v**,**w**, 0.100 mmol, 1.00 equiv) were added under a static pressure of nitrogen gas, followed by dropwise addition of MeOH (8 µL, 2.00 equiv). The reaction was stirred for overnight at room temperature. The reaction mixture was concentrated under vaccuum and the residue was purified by flash column chromatography on silica gel using the indicated mixture of cyclohexane and ethyl acetate.

Method B:

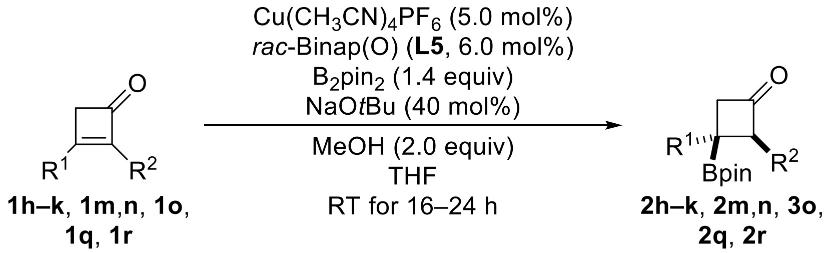

An oven-dried 10-mL Schlenk tube equipped with a magnetic stir bar was charged with $Cu(CH_3CN)_4PF_6$ (1.86 mg, 5.00 mol %), *rac*-Binap(O) (**L5**, 3.75 mg, 6.00 mol %), Na*Ot*Bu (1.92 mg, 0.200 equiv) and B_2pin_2 (35.5 mg, 0.140 mmol, 1.40 equiv). The tube was evacuated under high vacuum and backfilled with nitrogen gas (3 times). THF (1 mL) was added to the tube, and the resulting mixture was stirred under room temperature for 15 minutes. The corresponding cyclobutenones (**1h–k**, **1m**,**n**, **1o**, **1q**, **1r**, 0.100 mmol, 1.00 equiv) were added under a static pressure of nitrogen gas, followed by dropwise addition of MeOH (8 µL, 2.00 equiv). The reaction was stirred for overnight at room temperature. The reaction mixture was concentrated under vaccuum and the residue was purified by flash column chromatography on silica gel using the indicated mixture of cyclohexane and ethyl acetate.

4.3 2.00 mmol Scale Reaction

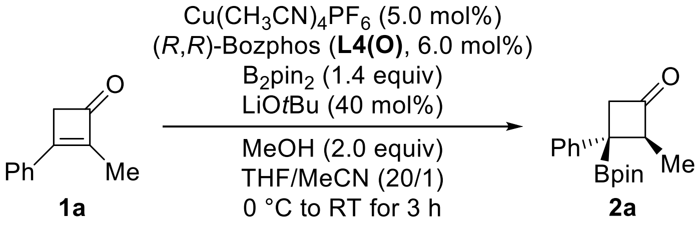

An oven-dried 100-mL Schlenk tube equipped with a magnetic stir bar was charged with $Cu(CH_3CN)_4PF_6$ (37.3 mg, 5.00 mol%), (*R*,*R*)-BozPhos (38.7 mg, 6.00 mol %) and B_2pin_2 (711 mg, 2.80 mmol, 1.40 equiv). The tube was evacuated under high vacuum and backfilled with nitrogen gas (3 times). THF/MeCN (20/1, 20 mL) was added to the tube, and the resulting mixture was stirred under room temperature for 15 minutes. The mixture was then cooled to 0 °C, and LiO*t*Bu (2.2 M, 363 μL, 0.800 mmol, 0.400 equiv.) was added dropwise and then the mixture was stirred under 0 °C for 10 minutes. Cyclobutenone **1a** (316 mg, 2.00 mmol, 1.00 equiv) were added under a static pressure of nitrogen gas, followed by dropwise addition of MeOH (160 μL, 2.00 equiv). The ice bath was subsequently removed and the reaction was stirred for 3 h at room temperature. After the indicated reaction time, the reaction mixture was filtered through a short plug of silica (fritted funnel with ~2 inches silica), and the filter cake was washed with EtOAc (15 mL). The filtrate was concentrated under vaccuum and determined by ^1H NMR spectroscopy to obtain diastereoselectivity with CH_2Br_2 as an internal standard. The diastereomeric ratio was determined by ^1H NMR spectroscopy of the crude reaction mixture by the relative peak height of the corresponding methyl protons which appear at 1.41 and 0.75 ppm for the major and minor diastereomers, respectively. The enantiomeric excess of **2a** was determined by HPLC analysis on a chiral stationary phase (*Daicel* Chiralcel IC column, column temperature 20°C, solvent *n*-heptane:*i*-PrOH = 99:1, flow rate 0.5 mL/min): t_R = 27.1 min (major), t_R = 25.2 min (minor). The residue was purified by flash column chromatography on silica gel using cyclohexane:ethyl acetate = 20:1 as the eluent to afford **2a** as a white solid (0.418 g, 73% yield, d.r. = 94:6, 98% ee).

4.4 Characterization Data of Cyclobutylboronates (2a–2n, 2p–2w, 3o, 6b)

4.4.1 (2*S*,3*R*)-2-Methyl-3-phenyl-3-(4,4,5,5-tetramethyl-1,3,2-dioxaborolan-2-yl)cyclobutan-1-one (2a)

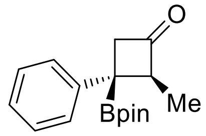

2a
$C_{17}H_{23}BO_3$
M = 286.1780 g/mol

Prepared from 2-methyl-3-phenylcyclobut-2-en-1-one (**1a**, 31.6 mg, 0.200 mmol,1.00 equiv) according to **GP1**. The residue was purified by flash column chromatography on silica gel using cyclohexane:ethyl acetate = 10:1 as the eluent to afford **2a** as a white solid (48.7 mg, 85% yield, d.r. = 95:5). **R$_f$** = 0.32 (cyclohexane:ethyl acetate = 10:1). **IR** (ATR): $\tilde{\nu}$ = 2977, 2929, 1778, 1354, 1322, 1140, 963, 857, 700 cm^{-1}. **^{1}H NMR** (400 MHz, $CDCl_3$, 298 K) δ = 7.35–7.29 (m, 2H), 7.24–7.17 (m, 3H), 3.58 (qt, *J* = 7.4, 2.1, 2.4 Hz, 1H), 3.52 (dd, *J* = 16.6, 2.2 Hz, 1H), 3.23 (dd, *J* = 16.6, 2.0 Hz, 1H), 1.42 (d, *J* = 7.6 Hz, 3H), 1.19 (s, 6H), 1.16 (s, 6H) ppm. **^{13}C NMR** (101 MHz, $CDCl_3$, 298 K) δ = 209.1, 147.2, 128.3, 126.1, 125.6, 84.2, 64.2, 53.5, 24.8, 24.7, 13.1 ppm [Note: the carbon atom attached to the boron atom was not detected due to quadrupole broadening caused by the ^{11}B nucleus]. **^{11}B NMR** (161 MHz, $CDCl_3$, 298 K) δ = 33.1 ppm. **HRMS** (APCI) exact mass for $[M+H]^+$ $C_{17}H_{24}BO_3^+$: calculated 286.1740, found 287.1816. Optical rotation: $[\alpha]_D^{20}$ = –66.5 (c 1.0, $CHCl_3$, 98% ee). The enantiomeric excess of **2a** was determined by HPLC analysis on a chiral stationary phase (*Daicel* Chiralcel IC column, column temperature 20°C, solvent *n*-heptane:*i*-PrOH = 99:1, flow rate 0.5 mL/min): t_R = 27.1 min (major), t_R = 25.2 min (minor).

4.4.2 (2*S*,3*R*)-2-Methyl-3-(4,4,5,5-tetramethyl-1,3,2-dioxaborolan-2-yl)-3-(p-tolyl)cyclobutan-1-one (2b)

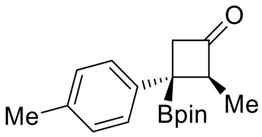

2b
$C_{18}H_{25}BO_3$
M = 300.2050 g/mol

Prepared from 2-methyl-3-(*p*-tolyl)cyclobut-2-en-1-one (**1b**, 34.4 mg, 0.200 mmol, 1.00 equiv.) according to **GP1**. The residue was purified by flash column chromatography on silica gel using cyclohexane:ethyl acetate = 10:1 as the eluent to afford **2b** as a white solid (39.6 mg, 66%

yield, d.r. = 94:6). **R**$_f$ = 0.31 (cyclohexane:ethyl acetate = 10:1). **IR** (ATR): $\tilde{\nu}$ = 2978, 2925, 1775, 1451, 1355, 1323, 1140, 857, 815, 755 cm^{-1}. **^{1}H NMR** (500 MHz, $CDCl_3$, 298 K): δ = 7.16–7.08 (m, 4H), 3.59–3.52 (qt, *J* = 7.5, 2.2, 2.2 Hz, 1H), 3.49 (dd, *J* = 16.3, 2.2 Hz, 1H), 3.19 (dd, *J* = 16.6, 2.0 Hz, 1H), 2.33 (s, 3H), 1.40 (d, *J* = 7.3 Hz, 3H), 1.19 (s, 6H), 1.16 (s, 6H) ppm. **^{13}C NMR** (126 MHz, $CDCl_3$, 298 K) δ = 209.2, 144.2, 135.0, 129.0, 126.0, 84.2, 64.2, 53.6, 24.8, 24.7, 21.0, 13.0 ppm [Note: the carbon atom attached to the boron atom was not detected due to quadrupole broadening caused by the ^{11}B nucleus]. **^{11}B NMR** (161 MHz, $CDCl_3$, 298 K) δ = 33.1 ppm. **HRMS** (APCI) exact mass for $[M+H]^+$ $C_{18}H_{26}BO_3^+$: calculated 301.1970, found 301.1971. Optical rotation: $[\alpha]_D^{20}$ = –77.4 (c 1.0, $CHCl_3$, 99% ee).

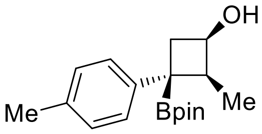

6b
$C_{18}H_{27}BO_3$
M = 302.22 g/mol

(1*R*,2*S*,3*R*)-2-Methyl-3-(4,4,5,5-tetramethyl-1,3,2-dioxaborolan-2-yl)-3-(*p*-tolyl)cyclobutan-1-ol (6b): Prepared from the product **2b** according to the reported procedure for determination of enantioselectivity of **2b**.[S2a] **^{1}H NMR** (500 MHz, $CDCl_3$, 298 K): δ = 7.09–7.06 (m, 2H), 7.02–6.98 (m, 2H), 4.26–4.19 (m, 1H), 3.12 (dd, *J* = 9.6 Hz, 1H), 2.83–2.76 (m, 1H), 2.49 (dd, *J* = 12.1, 6.6 Hz, 1H), 2.41 (dd, *J* = 12.4, 2.1 Hz, 1H), 2.31 (s, 3H), 1.28 (d, *J* = 7.33 Hz, 3H), 1.23 (s, 6H), 1.21 (s, 6H) ppm. **^{13}C NMR** (126 MHz, $CDCl_3$, 298 K) δ = 145.6, 134.0, 128.7, 125.5, 84.1, 68.7, 46.0, 39.2, 24.76, 24.66, 21.0, 12.9 ppm [Note: the carbon atom attached to the boron atom was not detected due to quadrupole broadening caused by the ^{11}B nucleus]. **^{11}B NMR** (161 MHz, $CDCl_3$, 298 K) δ = 33.6 ppm. The NMR spectroscopic data are in accordance with those reported.[S2a] The enantiomeric excess of **6b** was determined by HPLC analysis on a chiral stationary phase (*Daicel* Chiralcel IC column, column temperature 20°C, solvent *n*-heptane:*i*-PrOH = 95:5, flow rate 0.7 mL/min): t_R = 11.2 min (major), t_R = 10.2 min (minor).

4.4.3 (2*S*,3*R*)-2-Methyl-3-(4,4,5,5-tetramethyl-1,3,2-dioxaborolan-2-yl)-3-(*m*-tolyl)cyclobutan-1-one (2c)

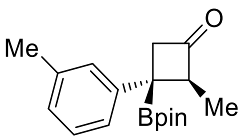

2c
$C_{18}H_{25}BO_3$
M = 300.2050 g/mol

Prepared from 2-methyl-3-(*m*-tolyl)cyclobut-2-en-1-one (**1c**, 34.4 mg, 0.200 mmol, 1.00 equiv) according to **GP1**. The residue was purified by flash column chromatography on silica gel using using cyclohexane:ethyl acetate = 10:1 as the eluent to afford **2c** as a white solid (35.4 mg, 59% yield, d.r. = 93:7). $\mathbf{R_f}$ = 0.31 (cyclohexane:ethyl acetate = 10:1). **IR** (ATR): $\tilde{\nu}$ = 2977, 2928, 2025, 1776, 1604, 1353, 1323, 1140, 1093, 757 cm^{-1}. **^{1}H NMR** (400 MHz, $CDCl_3$, 298 K) δ = 7.24–7.18 (m, 1H), 7.04–6.99 (m, 3H), 3.58 (qt, *J* = 7.4, 2.2, 2.2 Hz, 1H), 3.50 (dd, *J* = 16.6, 2.3 Hz, 1H), 3.20 (dd, *J* = 16.6, 2.0 Hz, 1H), 2.36 (s, 3H), 1.41 (d, *J* = 7.4 Hz, 3H), 1.19 (s, 6H), 1.16 (s, 6H) ppm. **^{13}C NMR** (126 MHz, $CDCl_3$, 298 K) δ = 208.2, 146.1, 136.8, 127.2, 125.7, 125.3, 122.2, 83.1, 62.9, 52.6, 23.7, 23.6, 20.5, 12.0 ppm [Note: the carbon atom attached to the boron atom was not detected due to quadrupole broadening caused by the ^{11}B nucleus]. **^{11}B NMR** (161 MHz, $CDCl_3$, 298 K) δ = 33.1 ppm. **HRMS** (APCI) exact mass for $[M+H]^+$ $C_{18}H_{26}BO_3^+$: calculated 301.1970, found 301.1970. Optical rotation: $[\alpha]_D^{20}$ = –66.7 (c 1.0, $CHCl_3$, 97% ee). The enantiomeric excess of **2c** was determined by HPLC analysis on a chiral stationary phase (*Daicel* Chiralcel IC column, column temperature 20°C, solvent *n*-heptane:*i*-PrOH = 99:1, flow rate 0.5 mL/min): t_R = 24.7 min (major), t_R = 23.6 min (minor).

4.4.4 (2*S*,3*R*)-2-Methyl-3-(4,4,5,5-tetramethyl-1,3,2-dioxaborolan-2-yl)-3-(*o*-tolyl)cyclobutan-1-one (2d)

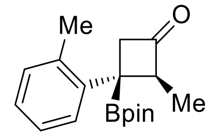

2d
$C_{18}H_{25}BO_3$
M = 300.2050 g/mol

Prepared from 2-methyl-3-(*o*-tolyl)cyclobut-2-en-1-one (**1**d, 34.4 mg, 0.200 mmol, 1.00 equiv) according to **GP1**. The residue was purified by flash column chromatography on silica gel using cyclohexane: ethyl acetate = 10:1 as the eluent to afford **2d** as a white solid (49.2 mg, 82% yield, d.r. > 95:5). **M.p.** = 85–86°C (cyclohexane). $\mathbf{R_f}$ = 0.21 (cyclohexane:ethyl acetate = 10:1). **IR** (ATR): $\tilde{\nu}$ = 2978, 2928, 1778, 1351, 1323, 1140, 857, 760, 727 cm^{-1}. **^{1}H NMR** (500 MHz, $CDCl_3$, 298 K) δ = 7.23–7.11 (m, 4H), 3.73 (q, *J* = 7.3 Hz, 1H), 3.54 (dd, *J* = 16.1, 1.9 Hz, 1H), 3.01 (dd, *J* = 16.0, 1.8 Hz, 1H), 2.23 (s, 3H), 1.42 (d, *J* = 7.32 Hz, 3H), 1.22 (s, 6H), 1.20 (s, 6H) ppm. **^{13}C NMR** (126 MHz, $CDCl_3$, 298 K) δ = 208.8, 145.1, 135.6, 130.7, 126.0, 125.96, 125.6, 84.3, 61.8, 54.5, 24.98, 24.96, 20.6, 12.4 ppm [Note: the carbon atom attached to the boron atom was not detected due to quadrupole broadening caused by the ^{11}B nucleus]. **^{11}B NMR** (161 MHz, $CDCl_3$, 298 K) δ = 33.4 ppm. **HRMS** (APCI) exact mass for $[M+H]^+$ $C_{18}H_{26}BO_3^+$: calculated 301.1970, found 301.1967. Optical rotation: $[\alpha]_D^{20}$ = –125.0 (c 1.1, $CHCl_3$, 94% ee). The enantiomeric excess of **2d** was determined by HPLC analysis on a chiral stationary phase

(*Daicel* Chiralcel IC column, column temperature 20°C, solvent *n*-heptane:*i*-PrOH = 95:5, flow rate 0.7 mL/min): t_R = 13.2 min (major), t_R = 11.3 min (minor).

4.4.5 (2*S*,3*R*)-3-(4-(*tert*-Butyl)phenyl)-2-methyl-3-(4,4,5,5-tetramethyl-1,3,2-dioxaborolan-2-yl)cyclobutan-1-one (2e)

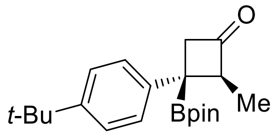

2e
$C_{21}H_{31}BO_3$
M = 342.2860 g/mol

Prepared from 3-(4-(*tert*-butyl)phenyl)-2-methylcyclobut-2-en-1-one (**1e**, 42.9 mg, 0.200 mmol, 1.00 equiv) according to **GP1**. The residue was purified by flash column chromatography on silica gel using cyclohexane:ethyl acetate = 10:1 as the eluent to afford **2e** as a white solid (42.4 mg, 62% yield, d.r. = 92:8). **R_f** = 0.35 (cyclohexane:ethyl acetate = 10:1). **IR** (ATR): $\tilde{\nu}$ = 2964, 2868, 1779, 1355, 1323, 1270, 1141, 857, 828 cm^{-1}. **^{1}H NMR** (500 MHz, $CDCl_3$, 298 K) δ = 7.34 (d, *J* = 8.4 Hz, 2H), 7.15 (d, *J* = 8.4 Hz, 2H), 3.57 (qt, *J* = 7.4, 2.3, 2.0 Hz, 1H), 3.50 (dd, *J* = 16.6, 2.1 Hz, 1H), 3.21 (dd, *J* = 16.6, 2.0 Hz, 1H), 1.40 (d, *J* = 7.5 Hz, 3H), 1.32 (s, 9H), 1.20 (s, 6H), 1.17 (s, 6H) ppm. **^{13}C NMR** (126 MHz, $CDCl_3$, 298 K) δ = 209.3, 148.2, 143.9, 125.7, 125.2, 84.1, 64.1, 53.6, 34.3, 31.4, 24.8, 24.7, 13.0 ppm [Note: the carbon atom attached to the boron atom was not detected due to quadrupole broadening caused by the ^{11}B nucleus]. **^{11}B NMR** (161 MHz, $CDCl_3$, 298 K) δ = 33.6 ppm. **HRMS** (APCI) exact mass for $[M+H]^+$ $C_{21}H_{32}BO_3^+$: calculated 343.2439, found 343.2441. Optical rotation: $[\alpha]_D^{20}$ = –62.7 (c 1.1, $CHCl_3$, 97% ee). The enantiomeric excess of **2e** was determined by HPLC analysis on a chiral stationary phase (*Daicel* Chiralcel OD-H column, column temperature 20°C, solvent *n*-heptane:*i*-PrOH = 99:1, flow rate 0.5 mL/min): t_R = 12.4 min (major), t_R = 15.2 min (minor).

4.4.6 (2*S*,3*R*)-3-(4-Methoxyphenyl)-2-methyl-3-(4,4,5,5-tetramethyl-1,3,2-dioxaborolan-2-yl)cyclobutan-1-one (2f)

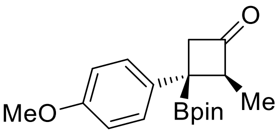

2f
$C_{18}H_{25}BO_4$
M = 316.2040

Prepared from 3-(4-methoxyphenyl)-2-methylcyclobut-2-en-1-one (**1f**, 37.6 mg, 0.200 mmol, 1.00 equiv) according to **GP1**. The residue was purified by flash column chromatography on silica gel using cyclo-hexane:ethyl acetate = 10:1 as the eluent to afford **2f** as a white solid

(33.5 mg, 53% yield, d.r. = 89:11). **M.p.** = 44–45°C (cyclohexane). **R$_f$** = 0.17 (cyclohexane:ethyl acetate = 10:1). **IR** (ATR): $\tilde{\nu}$ = 2977, 2930, 1776, 1510, 1355, 1322, 1247, 1140, 1035, 857, 829 cm^{-1}. **^{1}H NMR** (500 MHz, $CDCl_3$, 298 K) δ = 7.16–7.12 (m, 2H), 6.88–6.84 (m, 2H), 3.80 (s, 3H), 3.56–3.45 (m, 2H), 3.18 (dd, *J* = 16.5, 2.1 Hz, 1H), 1.39 (d, *J* = 7.4 Hz, 3H), 1.18 (s, 6H), 1.16 (s, 6H) ppm. **^{13}C NMR** (126 MHz, $CDCl_3$, 298 K) δ = 209.2, 157.5, 139.2, 127.1, 113.8, 84.1, 64.3, 55.2, 53.5, 24.75, 24.67, 13.0 ppm [Note: the carbon atom attached to the boron atom was not detected due to quadrupole broadening caused by the ^{11}B nucleus]. **^{11}B NMR** (160 MHz, $CDCl_3$, 298 K) δ = 33.4 ppm. **HRMS** (APCI) exact mass for $[M+O+H]^+$ $C_{18}H_{26}BO_5^+$: calculated 333.1868, found 333.1870. Optical rotation: $[\alpha]_D^{20}$ = –71.8 (c 1.1, $CHCl_3$, 98% ee). The enantiomeric excess of **2f** was determined by HPLC analysis on a chiral stationary phase (*Daicel* Chiralcel IC column, column temperature 20°C, solvent *n*-heptane:*i*-PrOH = 95:5, flow rate 0.7 mL/min): t_R = 18.5 min (major), t_R = 16.1 min (minor).

4.4.7 (2*S*,3*R*)-3-(3-Methoxyphenyl)-2-methyl-3-(4,4,5,5-tetramethyl-1,3,2-dioxaborolan-2-yl)cyclobutan-1-one (2g)

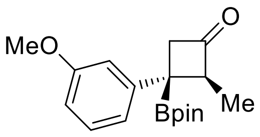

2g
$C_{18}H_{25}BO_4$
M = 316.2040

Prepared from 3-(3-methoxyphenyl)-2-methylcyclobut-2-en-1-one (**1g**, 37.6 mg, 0.200 mmol, 1.00 equiv) according to **GP1**. The residue was purified by flash column chromatography on silica gel using cyclohexane:ethyl acetate = 10:1 as the eluent to afford **2g** as a white solid (39.2 mg, 62% yield, d.r. = 92:8). **M.p.** = 53–54°C (cyclohexane). **R$_f$** = 0.18 (cyclohexane:ethyl acetate = 10:1). **IR** (ATR): $\tilde{\nu}$ = 2977, 2930, 1777, 1601, 1581, 1353, 1140, 1050, 858, 756 cm^{-1}. **^{1}H NMR** (500 MHz, $CDCl_3$, 298 K) δ = 7.26–7.22 (m, 1H), 6.83–6.79 (m, 1H), 6.77–6.72 (m, 2H), 3.81 (s, 3H), 3.58 (qt, *J* = 7.4, 2.2, 2.1 Hz, 1H), 3.49 (dd, *J* = 16.3, 2.3 Hz, 1H), 3.21 (dd, *J* = 16.5, 2.1 Hz, 1H), 1.40 (d, *J* = 7.5 Hz, 3H), 1.19 (s, 6H), 1.17 (s, 6H) ppm. **^{13}C NMR** (126 MHz, $CDCl_3$, 298 K) δ = 208.9, 159.5, 148.9, 129.3, 118.7, 112.6, 110.4, 84.2, 64.0, 55.2, 53.5, 24.75, 24.67, 13.0 ppm [Note: the carbon atom attached to the boron atom was not detected due to quadrupole broadening caused by the ^{11}B nucleus]. **^{11}B NMR** (160 MHz, $CDCl_3$, 298 K) δ = 33.4 ppm. **HRMS** (APCI) exact mass for $[M+H]^+$ $C_{18}H_{26}BO_4^+$: calculated 317.1919, found 317.1918. Optical rotation: $[\alpha]_D^{20}$ = –73.8 (c 0.54, $CHCl_3$, 96% ee). The enantiomeric excess of **2g** was determined by HPLC analysis on a chiral stationary phase (*Daicel* Chiralcel IC column, column temperature 20°C, solvent *n*-heptane:*i*-PrOH = 95:5, flow rate 0.7 mL/min): t_R = 16.4 min (major), t_R = 14.6 min (minor).

4.4.8 (2*S*,3*R*)-3-(4-Fluorophenyl)-2-methyl-3-(4,4,5,5-tetramethyl-1,3,2-dioxaborolan-2-yl)cyclobutan-1-one (2h)

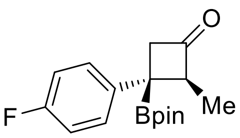

2h
$C_{17}H_{22}BFO_3$
M = 304.17 g/mol

Prepared from 3-(4-fluorophenyl)-2-methylcyclobut-2-en-1-one (**1h**, 35.2 mg, 0.200 mmol, 1.00 equiv) according to **GP1**. The residue was purified by flash column chromatography on silica gel using cyclohexane:ethyl acetate = 10:1 as the eluent to afford **2h** as a white solid (37.7 mg, 62% yield, d.r. = 95:5). **M.p.** = 84–85°C (cyclohexane). **R$_f$** = 0.25 (cyclohexane:ethyl acetate = 10:1). **IR** (ATR): $\tilde{\nu}$ = 2978, 2930, 1778, 1508, 1356, 1325, 1230, 1140, 857, 833 cm^{-1}. **^{1}H NMR** (500 MHz, $CDCl_3$, 298 K) δ = 7.19–7.14 (m, 2H), 7.03–6.97 (m, 2H), 3.57–3.46 (m, 2H), 3.18 (dd, *J* = 14.5, 2.0 Hz, 1H), 1.40 (d, *J* = 7.3 Hz, 3H), 1.19 (s, 6H), 1.16 (s, 6H) ppm. **^{13}C NMR** (126 MHz, $CDCl_3$, 298 K) δ = 208.4, 161.0 (d, *J* = 243.7 Hz), 142.8 (d, *J* = 3.5 Hz), 127.5 (d, *J* = 8.2 Hz), 115.1 (d, *J* = 21.0 Hz), 84.3, 64.4, 53.5, 24.72, 24.69, 12.9 ppm [Note: the carbon atom attached to the boron atom was not detected due to quadrupole broadening caused by the ^{11}B nucleus]. **^{11}B NMR** (160 MHz, $CDCl_3$, 298 K) δ = 33.0 ppm. **^{19}F NMR** (471 MHz, $CDCl_3$, 298 K) δ = –117.9 ppm. **HRMS** (APCI) exact mass for $[M+H]^+$ $C_{17}H_{23}BFO_3^+$: calculated 305.1719, found 305.1721. Optical rotation: $[\alpha]_D^{20}$ = –71.7 (c 1.1, $CHCl_3$, 99% ee). The enantiomeric excess of **2h** was determined by HPLC analysis on a chiral stationary phase (*Daicel* Chiralcel IC column, column temperature 20°C, solvent *n*-heptane:*i*-PrOH = 99:1, flow rate 0.5 mL/min): t_R = 31.5 min (major), t_R = 26.5 min (minor).

4.4.9 (2*S*,3*R*)-3-(4-Chlorophenyl)-2-methyl-3-(4,4,5,5-tetramethyl-1,3,2-dioxaborolan-2-yl)cyclobutan-1-one (2i)

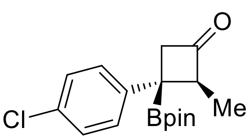

2i
$C_{17}H_{22}BClO_3$
M = 320.62 g/mol

Prepared from 3-(4-chlorophenyl)-2-methylcyclobut-2-en-1-one (**1i**, 38.4 mg, 0.200 mmol, 1.00 equiv.) according to **GP1**. The residue was purified by flash column chromatography on silica gel using cyclohexane:ethyl acetate = 10:1 as the eluent to afford **2i** as a white solid (64.1 mg, 66% yield, d.r. > 95:5). **R$_f$** = 0.29 (cyclohexane:ethyl acetate = 10:1). **IR** (ATR): $\tilde{\nu}$ = 2978, 2929,

1780, 1491, 1354, 1326, 1140, 1092, 856, 827 cm^{-1}. **^{1}H NMR** (400 MHz, $CDCl_3$, 298 K) δ = 7.30–7.26 (m, 2H), 7.16–7.12 (m, 2H), 3.56–3.47 (m, 2H), 3.18 (dd, *J* = 16.4, 2.3 Hz, 1H), 1.40 (d, *J* = 7.0 Hz, 3H), 1.19 (s, 6H), 1.16 (s, 6H) ppm. **^{13}C NMR** (101 MHz, $CDCl_3$, 298 K) δ = 208.2, 145.7, 131.4, 128.4, 127.5, 84.4, 64.4, 53.4, 24.77, 24.72, 13.0 ppm [Note: the carbon atom attached to the boron atom was not detected due to quadrupole broadening caused by the ^{11}B nucleus]. **^{11}B NMR** (160 MHz, $CDCl_3$, 298 K) δ = 33.1 ppm. **HRMS** (APCI) exact mass for $[M+H]^+$ $C_{17}H_{23}BClO_3^+$: calculated 321.1423, found 321.1428. Optical rotation: $[\alpha]_D^{20}$ = –56.9 (c 1.0, $CHCl_3$, 97% ee). The enantiomeric excess of **2i** was determined by HPLC analysis on a chiral stationary phase (*Daicel* Chiralcel IC column, column temperature 20°C, solvent *n*-heptane:*i*-PrOH = 99:1, flow rate 0.5 mL/min): t_R = 34.9 min (major), t_R = 28.7 min (minor).

4.4.10 (2*S*,3*R*)-3-(4-Bromophenyl)-2-methyl-3-(4,4,5,5-tetramethyl-1,3,2-dioxaborolan-2-yl)cyclobutan-1-one (2j)

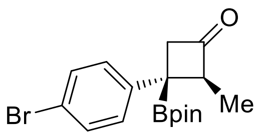

2j
$C_{17}H_{22}BBrO_3$
M = 365.07 g/mol

Prepared from 3-(4-bromophenyl)-2-methylcyclobut-2-en-1-one (**1j**, 47.2 mg, 0.200 mmol, 1.00 equiv.) according to **GP1**. The residue was purified by flash column chromatography on silica gel using cyclohexane:ethyl acetate = 10:1 as the eluent to afford **2j** as a white solid (56.2 mg, 77% yield, d.r. > 95:5). **R$_f$** = 0.29 (cyclohexane:ethyl acetate = 10:1). **IR** (ATR): $\tilde{\nu}$ = 698, 731, 771, 810, 1050, 1108, 1250, 1426, 1777, 2925, 2953 cm^{-1}. **^{1}H NMR** (500 MHz, $CDCl_3$, 298 K) δ = 7.45–7.42 (m, 2H), 7.10–7.07 (m, 2H), 3.55–3.46 (m, 2H), 3.18 (dd, *J* = 16.5, 2.2 Hz, 1H), 1.40 (d, *J* = 7.4 Hz, 3H), 1.19 (s, 6H), 1.16 (s, 6H) ppm. **^{13}C NMR** (101 MHz, $CDCl_3$, 298 K) δ = 208.2, 146.3, 131.4, 128.0, 119.4, 84.4, 64.4, 53.4, 24.8, 24.7, 13.0 ppm [Note: the carbon atom attached to the boron atom was not detected due to quadrupole broadening caused by the ^{11}B nucleus]. **^{11}B NMR** (160 MHz, $CDCl_3$, 298 K) δ = 32.7 ppm. **HRMS** (APCI) exact mass for $[M+H]^+$ $C_{17}H_{23}BBrO_3^+$: calculated 365.0918, found 365.0926. Optical rotation: $[\alpha]_D^{20}$ = –62.6 (c 1.0, $CHCl_3$, 96% ee). The enantiomeric excess of **2j** was determined by HPLC analysis on a chiral stationary phase (*Daicel* Chiralcel IC column, column temperature 20°C, solvent *n*-heptane:*i*-PrOH = 99:1, flow rate 0.5 mL/min): t_R = 36.8 min (major), t_R = 30.5 min (minor).

4.4.11 (2*S*,3*R*)-3-(3-Fluorophenyl)-2-methyl-3-(4,4,5,5-tetramethyl-1,3,2-dioxaborolan-2-yl)cyclobutan-1-one (2k)

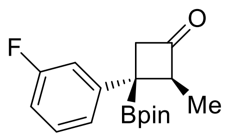

2k
$C_{17}H_{22}BFO_3$
M = 304.1684 g/mol

Prepared from 3-(3-fluorophenyl)-2-methylcyclobut-2-en-1-one (**1k**, 35.2 mg, 0.200 mmol, 1.00 equiv) according to **GP1**. The residue was purified by flash column chromatography on silica gel using cyclohexane:ethyl acetate = 10:1 as the eluent to afford **2k** as a white solid (56.0 mg, 92% yield, d.r. > 95:5). **R$_f$** = 0.19 (cyclohexane:ethyl acetate = 10:1). **IR** (ATR): $\tilde{\nu}$ = 2979, 2930, 1777, 1373, 1353, 1327, 1140, 1125, 850, 756 cm^{-1}. **^{1}H NMR** (400 MHz, $CDCl_3$, 298 K) δ = 7.31–7.24 (m, 1H), 7.00–6.96 (m, 1H), 6.94–6.86 (m, 2H), 3.60–3.53 (m, 1H), 3.50 (dd, *J* = 14.3, 2.2 Hz, 1H), 3.20 (dd, *J* = 14.5, 2.0 Hz, 1H), 1.40 (d, *J* = 7.3 Hz, 3H), 1.19 (s, 6H), 1.17 (s, 6H) ppm. **^{13}C NMR** (101 MHz, $CDCl_3$, 298 K) δ = 208.3, 162.8 (d, *J* = 245.4 Hz), 150.0 (d, *J* = 7.5 Hz), 129.7 (d, *J* = 8.5 Hz), 121.8 (d, *J* = 2.8 Hz), 113.3 (d, *J* = 21.7 Hz), 112.5 (d, *J* = 21.7 Hz), 84.4, 83.5, 64.3, 53.4, 24.76, 24.72, 13.0 ppm [Note: the carbon atom attached to the boron atom was not detected due to quadrupole broadening caused by the ^{11}B nucleus]. **^{11}B NMR** (160 MHz, $CDCl_3$, 298 K) δ = 32.9 ppm. **^{19}F NMR** (471 MHz, $CDCl_3$, 298 K) δ = –113.3 ppm. **HRMS** (APCI) exact mass for $[M+H]^+$ $C_{17}H_{23}BFO_3^+$: calculated 305.1719, found 305.1725. Optical rotation: $[\alpha]_D^{20}$ = –52.3 (c 1.0, $CHCl_3$, 97% ee). The enantiomeric excess of **2k** was determined by HPLC analysis on a chiral stationary phase (*Daicel* Chiralcel OD-H column, column temperature 20°C, solvent *n*-heptane:*i*-PrOH = 99:1, flow rate 0.5 mL/min): t_R = 14.5 min (major), t_R = 17.8 min (minor).

4.4.12 (2*S*,3*R*)-2-Methyl-3-(4,4,5,5-tetramethyl-1,3,2-dioxaborolan-2-yl)-3-(4-(trifluoromethyl)phenyl)cyclobutan-1-one (2l)

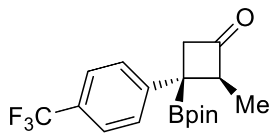

2l
$C_{18}H_{22}BF_3O_3$
M = 354.18 g/mol

Prepared from 2-methyl-3-(4-(trifluoromethyl)phenyl)cyclobut-2-en-1-one (**1l**, 45.2 mg, 0.200 mmol, 1.00 equiv) according to **GP1**. The residue was purified by flash column chromatography on silica gel using cyclohexane:ethyl acetate = 10:1 as the eluent to afford **2l** as a colorless oil

(45.3 mg, 64% yield, single diastereomer). **R**$_f$ = 0.26 (cyclohexane:ethyl acetate = 10:1). **IR** (ATR): $\tilde{\nu}$ = 2979, 2932, 1781, 1354, 1326, 1164, 1115, 1069, 1017, 857 cm^{-1}. **^{1}H NMR** (500 MHz, $CDCl_3$, 298 K) δ = 7.57 (d, *J* = 8.2 Hz, 2H), 7.32 (d, *J* = 8.1 Hz, 2H), 3.60–3.51 (m, 2H), 3.22 (dd, *J* = 16.2, 1.7 Hz, 1H), 1.43 (d, *J* = 7.4 Hz, 3H), 1.20 (s, 6H), 1.17 (s, 6H) ppm. **^{13}C NMR** (126 MHz, $CDCl_3$, 298 K) δ = 207.7, 151.4, 127.9 (q, *J* = 32.7 Hz), 126.5, 125.3 (q, *J* = 3.8 Hz), 84.5, 64.4, 53.3, 24.74, 24.70, 13.0 ppm [Note: the carbon atom attached to the boron atom was not detected due to quadrupole broadening caused by the ^{11}B nucleus]. **^{11}B NMR** (160 MHz, $CDCl_3$, 298 K) δ = 33.0 ppm. **^{19}F NMR** (470 MHz, $CDCl_3$, 298 K) δ = –62.3 ppm. **HRMS** (APCI) exact mass for $[M+H]^+$ $C_{18}H_{23}BF_3O_3^+$: calculated 355.1687, found 355.1691. Optical rotation: $[\alpha]_D^{20}$ = –55.0 (c 1.1, $CHCl_3$, 95% ee). The enantiomeric excess of **2l** was determined by HPLC analysis on a chiral stationary phase (*Daicel* Chiralcel IC column, column temperature 20°C, solvent *n*-heptane:*i*-PrOH = 99:1, flow rate 0.5 mL/min): t_R = 22.4 min (major), t_R = 20.7 min (minor).

4.4.13 Ethyl 4-((1*R*,2*S*)-2-methyl-3-oxo-1-(4,4,5,5-tetramethyl-1,3,2-dioxaborolan-2-yl)cyclobutyl)benzoate (2m)

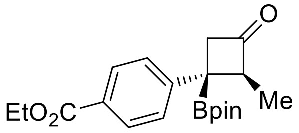

2m
$C_{20}H_{27}BO_5$
M = 358.24 g/mol

Prepared from ethyl 4-(2-methyl-3-oxocyclobut-1-en-1-yl)benzoate (**1m**, 71.6 mg, 0.200 mmol, 1.00 equiv) according to **GP1**. The residue was purified by flash column chromatography on silica gel using cyclohexane:ethyl acetate = 10:1 as the eluent to afford **2m** as a yellow oil (50.9 mg, 71% yield, d.r. > 95:5). **R**$_f$ = 0.34 (cyclohexane:ethyl acetate = 5:1). **IR** (ATR): $\tilde{\nu}$ = 2978, 2930, 1780, 1715, 1352, 1274, 1140, 1105, 1022, 858 cm^{-1}. **^{1}H NMR** (500 MHz, $CDCl_3$, 298 K) δ = 8.02 (d, *J* = 8.4 Hz, 2H), 7.29 (d, *J* = 8.4 Hz, 2H), 4.39 (q, *J* = 7.14 Hz, 2H), 3.65–3.53 (m, 2H), 3.26 (dd, *J* = 16.5, 1.9 Hz, 1H), 1.45 (d, *J* = 7.4 Hz, 3H), 1.41 (t, *J* = 7.2 Hz, 3H), 1.21 (s, 6H), 1.17 (s, 6H) ppm. **^{13}C NMR** (126 MHz, $CDCl_3$, 298 K) δ = 208.0, 166.5, 152.6, 129.6, 127.9, 126.1, 84.4, 64.3, 60.8, 53.3, 24.71, 24.67, 14.4, 13.0 ppm [Note: the carbon atom attached to the boron atom was not detected due to quadrupole broadening caused by the ^{11}B nucleus]. **^{11}B NMR** (160 MHz, $CDCl_3$, 298 K) δ = 32.9 ppm. **HRMS** (APCI) exact mass for $[M+H]^+$ $C_{20}H_{27}BO_5^+$: calculated 359.2024, found 359.2018. Optical rotation: $[\alpha]_D^{20}$ = –71.8 (c 1.1, $CHCl_3$, 97% ee). The enantiomeric excess of **2m** was determined by HPLC analysis on a chiral stationary phase (*Daicel* Chiralcel IC column, column temperature 20°C, solvent *n*-heptane:*i*-PrOH = 90:10, flow rate 0.5 mL/min): t_R = 38.7 min (major), t_R = 36.4 min (minor).

4.4.14 4-((1*R*,2*S*)-2-Methyl-3-oxo-1-(4,4,5,5-tetramethyl-1,3,2-dioxaborolan-2-yl)cyclobutyl)benzonitrile (2n)

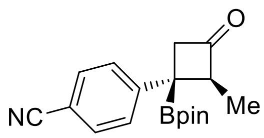

2n
$C_{18}H_{22}BNO_3$
M = 311.19 g/mol

Prepared from 4-(2-methyl-3-oxocyclobut-1-en-1-yl)benzonitrile (**1n**, 36.6 mg, 0.200 mmol, 1.00 equiv) according to **GP1**. The residue was purified by flash column chromatography on silica gel using cyclohexane:ethyl acetate = 10:1 as the eluent to afford **2n** as a yellow oil (62.2 mg, 59% yield, d.r. = 92:8). **R$_f$** = 0.35 (cyclohexane:ethyl acetate = 5:1). **IR** (ATR): $\tilde{\nu}$ = 2978, 2930, 2226, 1780, 1605, 1352, 1330, 1140, 856, 832 cm^{-1}. **^{1}H NMR** (500 MHz, $CDCl_3$, 298 K) δ = 7.61 (d, *J* = 8.4 Hz, 2H), 7.31 (d, *J* = 8.4 Hz, 2H), 3.58–3.50 (m, 2H), 3.21 (dd, *J* = 16.6, 2.1 Hz, 1H), 1.42 (d, *J* = 7.5 Hz, 3H), 1.19 (s, 6H), 1.16 (s, 6H) ppm. **^{13}C NMR** (126 MHz, $CDCl_3$, 298 K) δ = 207.1, 152.9, 132.2, 127.0, 119.0, 109.5, 84.7, 64.5, 53.1, 24.73, 24.71, 13.0 ppm [Note: the carbon atom attached to the boron atom was not detected due to quadrupole broadening caused by the ^{11}B nucleus]. **^{11}B NMR** (160 MHz, $CDCl_3$, 298 K) δ = 32.8 ppm. **HRMS** (APCI) exact mass for $[M+H]^+$ $C_{18}H_{23}BNO_3^+$: calculated 312.1766, found 312.1763. Optical rotation: $[\alpha]_D^{20}$ = –88.1 (c 1.1, $CHCl_3$, 96% ee). The enantiomeric excess of **2n** was determined by HPLC analysis on a chiral stationary phase (*Daicel* Chiralcel IC column, column temperature 20°C, solvent *n*-heptane:*i*-PrOH = 90:10, flow rate 0.8 mL/min): t_R = 47.6 min (major), t_R = 39.8 min (minor).

4.4.15 (2*S*,3*R*)-2-Methyl-3-(4-nitrophenyl)cyclobutan-1-one (3o)

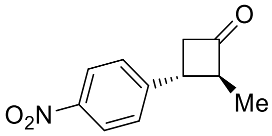

3o
$C_{11}H_{11}NO_3$
M = 205.21 g/mol

Prepared from 2-methyl-3-(4-nitrophenyl)cyclobut-2-en-1-one (**1o**, 40.6 mg, 0.200 mmol, 1.00 equiv) according to **GP1**. The residue was purified by flash column chromatography on silica gel using cyclohexane:ethyl acetate = 5:1 as the eluent to afford **3o** as a white solid (25.9 mg, 63% yield, d.r. = 88:12). **R$_f$** = 0.22 (cyclohexane:ethyl acetate = 5:1). **IR** (ATR): $\tilde{\nu}$ = 2966, 1779, 1600, 1516, 1345, 1111, 851, 740, 697 cm^{-1}. **^{1}H NMR** (500 MHz, $CDCl_3$, 298 K) δ = 8.22 (d, *J* = 8.7 Hz, 2H), 7.47 (d, *J* = 8.6 Hz, 2H), 3.46–3.37 (m, 2H), 3.32–3.25 (m, 2H), 1.33 (d, *J* = 7.2

Hz, 3H) ppm. **^{13}C NMR** (126 MHz, $CDCl_3$, 298 K) δ = 207.1, 150.4, 146.9, 127.4, 124.0, 63.2, 51.5, 37.8, 13.3 ppm. **HRMS** (APCI) exact mass for $[M+H]^+$ $C_{11}H_{12}NO_3^+$: calculated 206.0812, found 206.0814. Optical rotation: $[\alpha]_D^{20}$ = –52.3 (c 1.1, $CHCl_3$, 67% ee). The enantiomeric excess of **3o** was determined by HPLC analysis on a chiral stationary phase (*Daicel* Chiralcel IC column, column temperature 20°C, solvent *n*-heptane:*i*-PrOH = 90:10, flow rate 0.6 mL/min): t_R = 72.4 min (major), t_R = 63.5 min (minor).

4.4.16 (2*S*,3*R*)-3-([1,1'-Biphenyl]-4-yl)-2-methyl-3-(4,4,5,5-tetramethyl-1,3,2-dioxaborolan-2-yl)cyclobutan-1-one (2p)

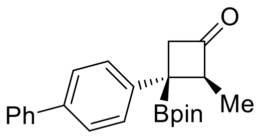

2p
$C_{23}H_{27}BO_3$
M = 362.28 g/mol

Prepared from 3-([1,1'-biphenyl]-4-yl)-2-methylcyclobut-2-en-1-one (**1p**, 46.9 mg, 0.200 mmol, 1.00 equiv) according to **GP1**. The residue was purified by flash column chromatography on silica gel using cyclohexane:ethyl acetate = 10:1 as the eluent to afford **2p** as a white solid (44.2 mg, 61% yield, d.r. = 94:6). **R_f** = 0.13 (cyclohexane:ethyl acetate = 10:1). **IR** (ATR): $\tilde{\nu}$ = 2974, 2924, 1771, 1346, 1323, 1136, 1094, 854, 827, 762, 732, 696 cm^{-1}. **^{1}H NMR** (400 MHz, $CDCl_3$, 298 K) δ = 7.64–7.54 (m, 4H), 7.47–7.41 (m, 2H), 7.37–7.28 (m, 3H), 3.62 (qt, *J* = 7.4, 7.2, 2.1 Hz, 1H), 3.56 (dd, *J* = 16.6, 2.2 Hz, 1H), 3.27 (dd, *J* = 16.8, 2.0 Hz, 1H), 1.45 (d, *J* = 7.5 Hz, 3H), 1.22 (s, 6H), 1.19 (s, 6H) ppm. **^{13}C NMR** (101 MHz, $CDCl_3$, 298 K) δ = 208.9, 146.3, 140.9, 138.4, 128.8, 127.13, 127.06, 126.99, 126.6, 84.3, 64.3, 53.6, 24.8, 24.7, 13.1 ppm [Note: the carbon atom attached to the boron atom was not detected due to quadrupole broadening caused by the ^{11}B nucleus]. **^{11}B NMR** (160 MHz, $CDCl_3$, 298 K) δ = 33.2 ppm. **HRMS** (APCI) exact mass for $[M+H]^+$ $C_{23}H_{28}BO_3^+$: calculated 363.2126, found 363.2130. Optical rotation: $[\alpha]_D^{20}$ = –67.7 (c 1.1, $CHCl_3$, >85% ee). The enantiomeric excess of **2p** was determined by HPLC analysis on a chiral stationary phase (*Daicel* Chiralcel OD-H column, column temperature 20°C, solvent *n*-heptane:*i*-PrOH = 99:1, flow rate 0.3 mL/min): t_R = 55.8 min (major), t_R = 60.9 min (minor) with no baseline separation.

4.4.17 (2*S*,3*R*)-2-Methyl-3-(4,4,5,5-tetramethyl-1,3,2-dioxaborolan-2-yl)-3-(thiophen-3-yl)cyclobutan-1-one (2q)

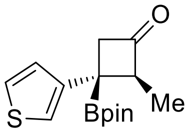

2q
$C_{15}H_{21}BO_3S$
M = 292.20 g/mol

Prepared from 2-methyl-3-(thiophen-3-yl)cyclobut-2-en-1-one (**1q**, 32.8 mg, 0.200 mmol, 1.00 equiv) according to **GP1**. The residue was purified by flash column chromatography on silica gel using cyclohexane:ethyl acetate = 10:1 as the eluent to afford **2q** as a yellow oil (38.6 mg, 66% yield, d.r. = 86:14). **M.p.** = 78–79°C (cyclohexane). $\mathbf{R_f}$ = 0.13 (cyclohexane:ethyl acetate = 10:1). **IR** (ATR): $\tilde{\nu}$ = 2977, 2924, 1780, 1371, 1353, 1322, 1141, 852, 777 cm^{-1}. ^{1}**H NMR** (500 MHz, $CDCl_3$, 298 K) δ = 7.31–7.27 (m, 1H), 7.03 (d, *J* = 4.0 Hz, 2H), 3.54–3.45 (m, 2H), 3.22 (dd, *J* = 16.6, 2.2 Hz, 1H), 1.33 (d, *J* = 7.3 Hz, 3H), 1.23 (s, 6H), 1.21 (s, 6H) ppm. ^{13}**C NMR** (126 MHz, $CDCl_3$, 298 K) δ = 209.3, 147.1, 127.0, 125.8, 118.6, 84.3, 65.2, 53.8, 24.8, 12.5 ppm [Note: the carbon atom attached to the boron atom was not detected due to quadrupole broadening caused by the ^{11}B nucleus]. ^{11}**B NMR** (160 MHz, $CDCl_3$, 298 K) δ = 33.1 ppm. **HRMS** (APCI) exact mass for $[M+H]^+$ $C_{15}H_{22}BO_3S^+$: calculated 293.1377, found 293.1379. Optical rotation: $[\alpha]_D^{20}$ = –55.6 (c 0.35, $CHCl_3$, >99% ee). The enantiomeric excess of **2q** was determined by HPLC analysis on a chiral stationary phase (*Daicel* Chiralcel IB column, column temperature 20°C, solvent *n*-heptane:*i*-PrOH = 99:1, flow rate 0.5 mL/min): t_R = 12.0 min (major), t_R = 12.5 min (minor).

4.4.18 (2*S*,3*R*)-3-(1*H*-Indol-5-yl)-2-methyl-3-(4,4,5,5-tetramethyl-1,3,2-dioxaborolan-2-yl)cyclobutan-1-one (2r)

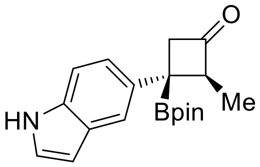

2r
$C_{19}H_{24}BNO_3$
M = 325.22 g/mol

Prepared from 3-(1*H*-indol-5-yl)-2-methylcyclobut-2-en-1-one (**1r**, 39.4 mg, 0.200 mmol, 1.00 equiv) according to **GP1**. The residue was purified by flash column chromatography on silica gel using cyclohexane:ethyl acetate = 1:2 as the eluent to afford **2r** as a white solid (46.8 mg, 72% yield, d.r. = 91:9). **M.p.** = 200–201°C (cyclohexane). $\mathbf{R_f}$ = 0.37 (cyclohexane:ethyl acetate = 1:2). **IR** (ATR): $\tilde{\nu}$ = 3369, 2976, 2929, 2870, 1768, 1354, 1316, 1141, 1096, 730 cm^{-1}. ^{1}**H**

NMR (500 MHz, $CDCl_3$, 298 K) δ = 8.21 (s, 1H), 7.47 (s, 1H), 7.33 (d, *J* = 8.4 Hz, 1H), 7.20–7.16 (m, 1H), 7.07 (dd, *J* = 8.5, 1.5 Hz, 1H), 6.53–6.49 (m, 1H), 3.69–3.62 (m, 1H), 3.57 (dd, *J* = 16.7, 2.1 Hz, 1H), 3.29 (dd, *J* = 16.5, 2.0 Hz, 1H), 1.47 (d, *J* = 7.5 Hz, 3H), 1.19 (s, 6H), 1.16 (s, 6H) ppm. **^{13}C NMR** (126 MHz, $CDCl_3$, 298 K) δ = 209.9, 138.6, 134.1, 127.9, 124.5, 121.1, 117.3, 111.0, 102.4, 84.0, 64.3, 54.0, 24.80, 24.66, 13.1 ppm [Note: the carbon atom attached to the boron atom was not detected due to quadrupole broadening caused by the ^{11}B nucleus]. **^{11}B NMR** (160 MHz, $CDCl_3$, 298 K) δ = 33.8 ppm. **HRMS** (APCI) exact mass for $[M+H]^+$ $C_{19}H_{25}BNO_3^+$: calculated 326.1922, found 326.1924. Optical rotation: $[\alpha]_D^{20}$ = +220.8 (c 1.0, $CHCl_3$, 94% ee). The enantiomeric excess of **2r** was determined by HPLC analysis on a chiral stationary phase (*Daicel* Chiralcel IC column, column temperature 20°C, solvent *n*-heptane:*i*-PrOH = 95:5, flow rate 0.8 mL/min): t_R = 35.2 min (major), t_R = 29.3 min (minor).

4.4.19 (2*S*,3*R*)-2-Ethyl-3-phenyl-3-(4,4,5,5-tetramethyl-1,3,2-dioxaborolan-2-yl)cyclobutan-1-one (2s)

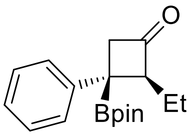

2s
$C_{18}H_{25}BO_3$
M = 300.21 g/mol

Prepared from 2-ethyl-3-phenylcyclobut-2-en-1-one (**1s**, 34.4 mg, 0.200 mmol, 1.00 equiv) according to **GP1**. The diastereomeric ratio was determined by relative ^{1}HNMR peak height of the CH_2 groups which appear at 3.17 and 3.35 ppm for the major and minor diastereomers, respectively. The residue was purified by flash column chromatography on silica gel using cyclohexane:ethyl acetate = 10:1 as the eluent to afford **2s** as a white solid (40.2 mg, 67% yield, d.r. = 90:10). **M.p.** = 40–41°C (cyclohexane). **R_f** = 0.31 (cyclohexane:ethyl acetate = 10:1). **IR** (ATR): $\tilde{\nu}$ = 2977, 2924, 1775, 1353, 1140, 1100, 964, 860, 760, 700 cm^{-1}. **^{1}H NMR** (500 MHz, $CDCl_3$, 298 K) δ = 7.35–7.29 (m, 2H), 7.25–7.17 (m, 3H), 3.53 (dd, *J* = 16.8, 2.4 Hz, 1H), 3.48–3.42 (m, 1H), 3.17 (dd, *J* = 16.7, 2.6 Hz, 1H), 1.98–1.88 (m, 1H), 1.86–1.76 (m, 1H), 1.19 (s, 6H), 1.16 (s, 6H), 1.16 (t, *J* = 7.5 Hz, 3H) ppm. **^{13}C NMR** (126 MHz, $CDCl_3$, 298 K) δ = 209.3, 147.2, 128.3, 126.3, 125.4, 84.2, 71.0, 53.8, 24.69, 24.67, 22.4, 13.0 ppm [Note: the carbon atom attached to the boron atom was not detected due to quadrupole broadening caused by the ^{11}B nucleus]. **^{11}B NMR** (160 MHz, $CDCl_3$, 298 K) δ = 33.2 ppm. **HRMS** (APCI) exact mass for $[M+H]^+$ $C_{18}H_{26}BO_3^+$: calculated 301.1970, found 301.1967. Optical rotation: $[\alpha]_D^{20}$ = –64.6 (c 1.0, $CHCl_3$, 92% ee). The enantiomeric excess of **2s** was determined by HPLC analysis on a chiral stationary phase (*Daicel* Chiralcel IC column, column temperature 20°C,

solvent *n*-heptane:*i*-PrOH = 95:5, flow rate 0.7 mL/min): t_R = 9.2 min (major), t_R = 8.5 min (minor).

4.4.20 (2*S*,3*R*)-2-Benzyl-3-phenyl-3-(4,4,5,5-tetramethyl-1,3,2-dioxaborolan-2-yl)cyclobutan-1-one (2t)

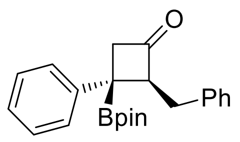

2t
$C_{23}H_{27}BO_3$
M = 362.28 g/mol

Prepared from 2-benzyl-3-phenylcyclobut-2-en-1-one (**1t**, 46.8 mg, 0.200 mmol, 1.00 equiv) according to **GP1**. The diastereomeric ratio was determined by relative ^{1}HNMR peak height of the CH proton which appear at 3.77 and 4.10 ppm for the major and minor diastereomers, respectively. The residue was purified by flash column chromatography on silica gel using cyclohexane:ethyl acetate = 10:1 as the eluent to afford **2t** as a yellow oil (44.2 mg, 61% yield, d.r. = 94:6). **R_f** = 0.29 (cyclohexane:ethyl acetate = 10:1). **IR** (ATR): $\tilde{\nu}$ = 2978, 2926, 1777, 1494, 1452, 1355, 1324, 1139, 699 cm^{-1}. **^{1}H NMR** (500 MHz, $CDCl_3$, 298 K) δ = 7.39–7.36 (m, 2H), 7.34–7.30 (m, 2H), 7.28–7.21 (m, 3H), 7.16 (tt, *J* = 7.3, 1.9, 1.2 Hz, 1H), 7.08–7.05 (m, 2H), 3.81–3.74 (m, 1H), 3.52 (dd, *J* = 16.4, 2.1 Hz, 1H), 3.27 (dd, *J* = 14.4, 8.4 Hz, 1H), 3.22 (dd, *J* = 16.4, 1.9 Hz, 1H), 3.11 (dd, *J* = 14.3, 5.7 HZ, 1H), 1.25 (s, 6H), 1.23 (s, 6H) ppm. **^{13}C NMR** (126 MHz, $CDCl_3$, 298 K) δ = 207.0, 146.7, 140.0, 129.1, 128.5, 128.3, 126.3, 125.6, 84.3, 70.5, 53.6, 34.8, 24.78, 24.73 ppm [Note: the carbon atom attached to the boron atom was not detected due to quadrupole broadening caused by the ^{11}B nucleus]. **^{11}B NMR** (160 MHz, $CDCl_3$, 298 K) δ = 33.3 ppm. **HRMS** (APCI) exact mass for $[M+H]^+$ $C_{23}H_{28}BO_3^+$: calculated 363.2126, found 363.2126. Optical rotation: $[\alpha]_D^{20}$ = –29.2 (c 0.74, $CHCl_3$, 98% ee). The enantiomeric excess of **2t** was determined by HPLC analysis on a chiral stationary phase (*Daicel* Chiralcel IB column, column temperature 20°C, solvent *n*-heptane:*i*-PrOH = 99:1, flow rate 0.5 mL/min): t_R = 13.8 min (major), t_R = 12.3 min (minor).

4.4.21 (2*S*,3*S*)-3-Phenyl-3-(4,4,5,5-tetramethyl-1,3,2-dioxaborolan-2-yl)-2-(trimethylsilyl)cyclobutan-1-one (2u)

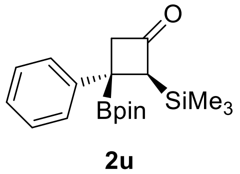

2u
$C_{19}H_{29}BO_3Si$
M = 344.33 g/mol

Prepared from 3-phenyl-2-(trimethylsilyl)cyclobut-2-en-1-one (**1u**, 43.2 mg, 0.200 mmol, 1.00 equiv) according to **GP1**. The residue was purified by flash column chromatography on silica gel using cyclohexane:ethyl acetate = 10:1 as the eluent to afford **2u** as a white solid (21.3 mg, 31% yield). $\mathbf{R_f}$ = 0.16 (cyclohexane:ethyl acetate = 10:1). **IR** (ATR): $\tilde{\nu}$ = 2979, 1758, 1373, 1344, 1319, 1249, 1139, 843, 757, 701, 667 cm^{-1}. **^{1}H NMR** (400 MHz, $CDCl_3$, 298 K) δ = 7.33–7.28 (m, 2H), 7.25–7.21 (m, 2H), 7.19–7.14 (m, 1H), 3.60–3.53 (m, 1H), 3.40–3.33 (m, 2H), 1.15 (s, 6H), 1.10 (s, 6H), 0.26 (s, 9H) ppm. **^{13}C NMR** (101 MHz, $CDCl_3$, 298 K) δ = 211.0, 148.1, 129.5, 128.1, 126.7, 85.4, 66.2, 57.0, 26.1, 25.8, 0.0 ppm [Note: the carbon atom attached to the boron atom was not detected due to quadrupole broadening caused by the ^{11}B nucleus]. **^{11}B NMR** (160 MHz, $CDCl_3$, 298 K) δ = 32.8 ppm. **^{29}Si{^{1}H} DEPT NMR** (99 MHz, $CDCl_3$, 298 K) δ = 0.12 ppm. **HRMS** (APCI) exact mass for $[M+H]^+$ $C_{19}H_{30}BO_3Si^+$: calculated 345.2052, found 345.2050. Optical rotation: $[\alpha]_D^{20}$ = +36.6 (c 1.0, $CHCl_3$, 72% ee). The enantiomeric excess of **2u** was determined by HPLC analysis on a chiral stationary phase (*Daicel* Chiralcel IC column, column temperature 20°C, solvent *n*-heptane:*i*-PrOH = 99:1, flow rate 0.5 mL/min): t_R = 23.3 min (major), t_R = 21.1 min (minor).

4.4.22 (2*S*,3*R*)-2,3-Dipropyl-3-(4,4,5,5-tetramethyl-1,3,2-dioxaborolan-2-yl)cyclobutan-1-one (2v)

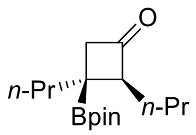

2v
$C_{16}H_{29}BO_3$
M = 280.22 g/mol

Prepared from 2,3-dipropylcyclobut-2-en-1-one (**1v**, 30.4 mg, 0.200 mmol, 1.00 equiv.) according to **GP1**. The diastereomeric ratio was determined by relative ^{1}HNMR peak height of the corresponding CH_2 protons of cyclobutyl skeleton which appear at 2.54 and 2.41 ppm for the major and minor diastereomers, respectively. The residue was purified by flash column chromatography on silica gel using cyclohexane:ethyl acetate = 10:1 as the eluent to afford **2v** as a yellow oil (38.1 mg, 68% yield, d.r. = 67:33). $\mathbf{R_f}$ = 0.36 (cyclohexane:ethyl acetate = 10:1). **IR** (ATR): $\tilde{\nu}$ = 2958, 2928, 2872, 1774, 1464, 1382, 1317, 1141, 853 cm^{-1}. **^{1}H NMR** (500 MHz, $CDCl_3$, 298 K) δ = 3.09 (dd, *J* = 16.9, 2.5 Hz, 1H), 2.90–2.85 (m, 1H), 2.56 (dd, *J* = 17.0, 2.8 Hz, 1H), 1.97–1.89 (m, 1H), 1.67–1.59 (m, 1H), 1.46–1.31 (m, 6H), 1.25 (s, 6H), 1.24 (s, 6H), 0.93 (t, *J* = 7.3 Hz, 3H), 0.89 (t, *J* = 7.3 Hz, 3H) ppm. **^{13}C NMR** (126 MHz, $CDCl_3$, 298 K) δ = 210.0, 83.7, 69.3, 52.1, 43.2, 30.4, 25.1, 24.9, 21.0, 14.6, 13.9 ppm [Note: the carbon atom attached to the boron atom was not detected due to quadrupole broadening caused by the ^{11}B nucleus]. **^{11}B NMR** (160 MHz, $CDCl_3$, 298 K) δ = 33.9 ppm. **HRMS** (APCI) exact mass for

[M+H]$^+$ $C_{16}H_{30}BO_3^+$: calculated 281.2283, found 281.2279. Optical rotation: $[\alpha]_D^{20}$ = –27.9 (c 0.32, $CHCl_3$, 83% ee). The enantiomeric excess of **2v** was determined by HPLC analysis on a chiral stationary phase (*Daicel* Chiralcel IC column, column temperature 20°C, solvent *n*-heptane:*i*-PrOH = 99:1, flow rate 0.5 mL/min): t_R = 16.6 min (major), t_R = 15.9 min (minor).

4.4.23 (2*S*,3*R*)-2,3-Diphenethyl-3-(4,4,5,5-tetramethyl-1,3,2-dioxaborolan-2-yl)cyclobutan-1-one (2w)

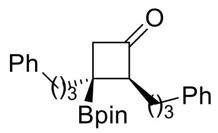

2w
$C_{28}H_{37}BO_3$
M = 432.41 g/mol

Prepared from 2,3-bis(3-phenylpropyl)cyclobut-2-en-1-one (**1w**, 60.8 mg, 0.200 mmol, 1.00 equiv) according to **GP1**. The diastereomeric ratio was determined by relative ^{1}HNMR peak height of the corresponding CH_2 protons of cyclobutyl skeleton which appear at 2.54 and 2.44 ppm for the major and minor diastereomers, respectively. The residue was purified by flash column chromatography on silica gel using cyclohexane:ethyl acetate = 10:1 as the eluent to afford **2w** as a yellow oil (54.5 mg, 63% yield, d.r. = 83:17). **R$_f$** = 0.47 (cyclohexane:ethyl acetate = 5:1). **IR** (ATR): $\tilde{\nu}$ = 2977, 2927, 2855, 1771, 1495, 1453, 1382, 1316, 1140, 856 cm^{-1}. **^{1}H NMR** (400 MHz, $CDCl_3$, 298 K) δ = 7.30–7.24 (m, 4H), 7.21–7.13 (m, 6H), 3.08 (dd, *J* = 16.9, 2.4 Hz, 1H), 2.91–2.83 (m, 1H), 2.71–2.50 (m, 5H), 2.05–1.94 (m, 1H), 1.77–1.51 (m, 7H), 1.22 (s, 6H), 1.21 (s, 6H) ppm. **^{13}C NMR** (101 MHz, $CDCl_3$, 298 K) δ = 210.2, 142.3, 142.1, 128.5, 128.34, 128.31, 125.8, 83.9, 69.4, 52.1, 40.4, 36.3, 35.9, 29.7, 29.6, 28.1, 25.2, 24.8 ppm [Note: the carbon atom attached to the boron atom was not detected due to quadrupole broadening caused by the ^{11}B nucleus]. **^{11}B NMR** (160 MHz, $CDCl_3$, 298 K) δ = 34.0 ppm. **HRMS** (APCI) exact mass for [M+H]$^+$ $C_{28}H_{38}BO_3^+$: calculated 433.2909, found 433.2902. Optical rotation: $[\alpha]_D^{20}$ = –12.0 (c 0.42, $CHCl_3$, 97% ee). The enantiomeric excess of **2w** was determined by HPLC analysis on a chiral stationary phase (*Daicel* Chiralcel IC column, column temperature 20°C, solvent *n*-heptane:*i*-PrOH = 99:1, flow rate 0.5 mL/min): t_R = 42.8 min (major), t_R = 35.4 min (minor).

5 Experimental Details for the 1,6-Borylation of a *para*-Quinone Methide

5.1 General Procedures for Enantioselective Borylation *para*-Quinone Methide (GP3)

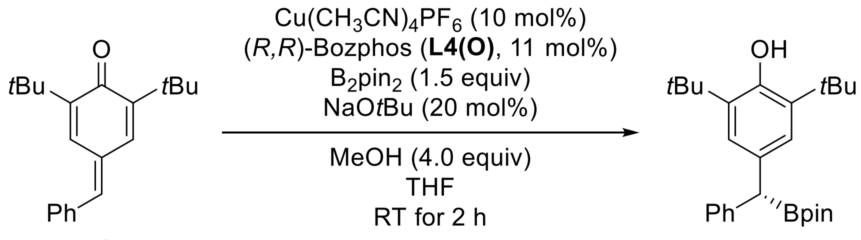

An oven-dried 10-mL Schlenk tube equipped with a magnetic stir bar was charged with $Cu(CH_3CN)_4PF_6$ (3.73 mg, 5.00 mol %), (*R*,*R*)-BozPhos (3.54 mg, 6.00 mol %) and B_2pin_2 (38.1 mg, 0.150 mmol, 1.50 equiv). The tube was evacuated under high vacuum and backfilled with nitrogen gas (3 times). THF (1 mL) was added to the tube, and the resulting mixture was stirred under room temperature for 30 minutes. NaO*t*Bu (1.92 mg, 0.0200 mmol, 0.200 equiv) in THF (0.2 ml) was added dropwise and then the mixture was stirred for 15 minutes. 4-benzylidene-2,6-di-*tert*-butylcyclohexa-2,5-dien-1-ol (**4**, 29.6 mg, 0.100 mmol, 1.00 equiv) were added under a static pressure of nitrogen gas, followed by dropwise addition of MeOH (16 µL, 4.00 equiv). Then, the reaction was stirred for 2 h at room temperature. After the indicated reaction time, the reaction mixture was filtered through a plug of silica and Celite, and the filter cake was washed with EtOAc (10 mL). The filtrate was concentrated under vaccuum and the residue was purified by flash column chromatography on silica gel using the indicated mixture of cyclohexane and ethyl acetate.

5.2 (*R*)-2,6-Di-tert-butyl-4-(phenyl(4,4,5,5-tetramethyl-1,3,2-dioxaborolan-2-yl)methyl)phenol (5)

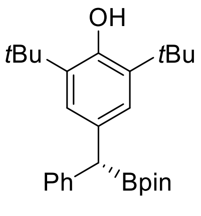

5

$C_{27}H_{39}BO_3$

M = 422.30 g/mol

Prepared from 4-benzylidene-2,6-di-tert-butylcyclohexa-2,5-dien-1-ol[S4] (**4**, 29.6 mg, 0.100 mmol,1.00 equiv) according to **GP3**. The residue was purified by flash column chromatography on silica gel using cyclohexane:ethyl acetate = 10:1 as the eluent to afford **5** as a yellow oil (35.9 mg, 85% yield). **^{1}H NMR** (500 MHz, $CDCl_3$, 298 K) δ = 7.32–7.26 (m, 4H), 7.20–7.16 (m, 1H), 7.12 (s, 2H), 5.04 (s, 1H), 3.82 (s, 1H), 1.45 (s, 18H), 1.28 (d, *J* = 5.3 Hz, 12H) ppm. **^{13}C NMR** (126 MHz, $CDCl_3$, 298 K) δ = 151.8, 143.1, 135.6, 132.1, 128.9, 128.2, 126.0, 125.2,

83.5, 34.4, 30.4, 24.7, 24.6 ppm [Note: the carbon atom attached to the boron atom was not detected due to quadrupole broadening caused by the ^{11}B nucleus]. ^{11}**B NMR** (160 MHz, $CDCl_3$, 298 K) δ = 32.8 ppm. The NMR spectroscopic data are in accordance with those reported.[S4] Optical rotation: $[\alpha]_D^{20}$ = –15.6 (c 1.6, $CHCl_3$, 78% ee). The enantiomeric excess of **5** was determined by HPLC analysis on a chiral stationary phase (*Daicel* Chiralcel OD-H column, column temperature 20°C, solvent *n*-heptane:*i*-PrOH = 99.8:0.2, flow rate 0.3 mL/min): t_R = 17.3 min (major), t_R = 18.7 min (minor).

6 HPLC Traces

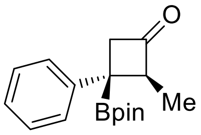

2a

(2*S*,3*R*)-2-Methyl-3-phenyl-3-(4,4,5,5-tetramethyl-1,3,2-dioxaborolan-2-yl)cyclobutan-1-one (**2a**): The enantiomeric excess of **2a** was determined by HPLC analysis on a chiral stationary phase (*Daicel* Chiralcel IC column, column temperature 20°C, solvent *n*-heptane:*i*-PrOH = 99:1, flow rate 0.5 mL/min): t_R = 26.0 min (major), t_R = 24.2 min (minor).

rac-**2a**

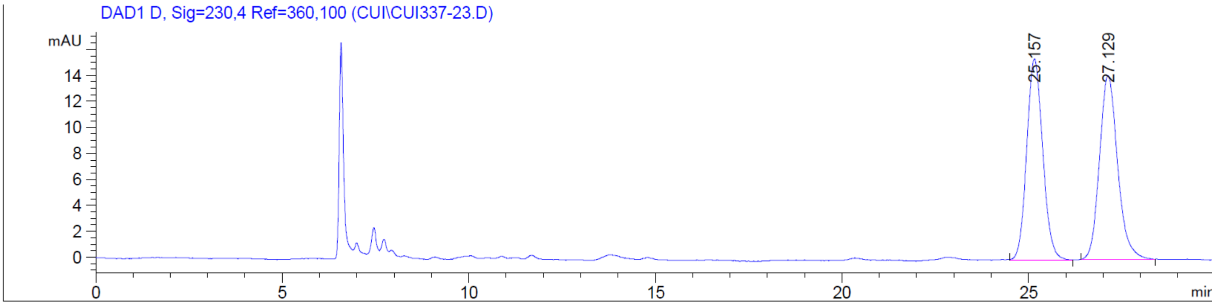

Signal 4: DAD1 D, Sig=230,4 Ref=360,100

Peak #	RetTime [min]	Type	Width [min]	Area [mAU*s]	Height [mAU]	Area %
1	25.157	BB	0.4584	461.86182	15.46794	49.0907
2	27.129	BB	0.5069	478.97101	14.14935	50.9093

(2*S*,3*R*)-**2a**

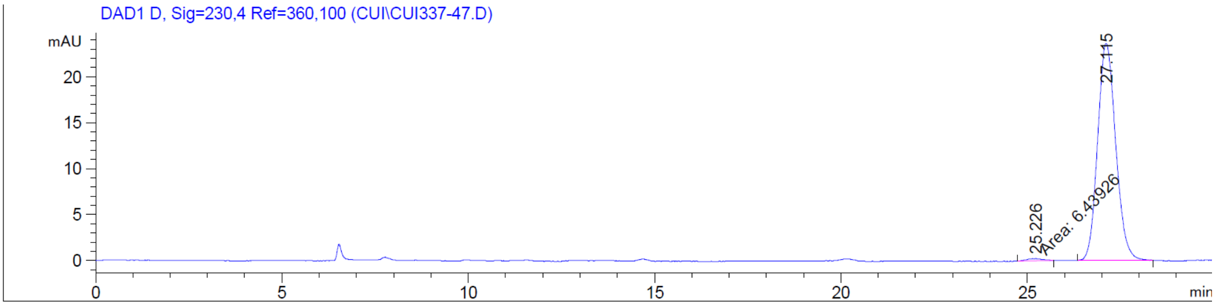

Signal 4: DAD1 D, Sig=230,4 Ref=360,100

Peak #	RetTime [min]	Type	Width [min]	Area [mAU*s]	Height [mAU]	Area %
1	25.226	MM	0.4783	6.43926	2.24376e-1	0.8009
2	27.115	BB	0.5179	797.55383	23.62089	99.1991

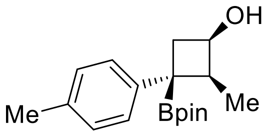

6b

(1*R*,2*S*,3*R*)-2-Methyl-3-(4,4,5,5-tetramethyl-1,3,2-dioxaborolan-2-yl)-3-(*p*-tolyl)cyclobutan-1-ol (6b): The enantiomeric excess of **6b** was determined by HPLC analysis on a chiral stationary phase (*Daicel* Chiralcel IC column, column temperature 20°C, solvent *n*-heptane:*i*-PrOH = 95:5, flow rate 0.7 mL/min): t_R = 11.2 min (major), t_R = 10.2 min (minor).

rac-**6b**

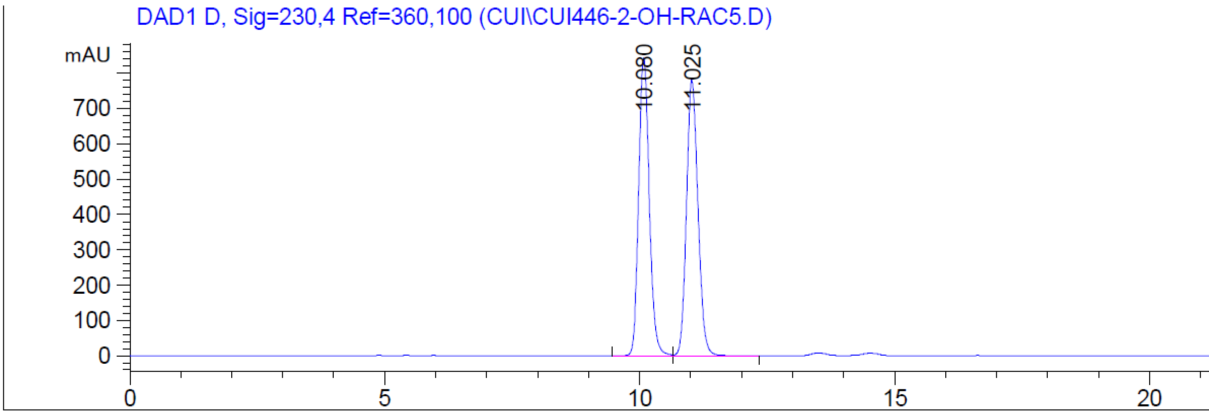

Signal 4: DAD1 D, Sig=230,4 Ref=360,100

Peak #	RetTime [min]	Type	Width [min]	Area [mAU*s]	Height [mAU]	Area %
1	10.080	BV	0.2228	1.21973e4	842.97601	49.8903
2	11.025	VB	0.2434	1.22510e4	779.28284	50.1097

(1*R*,2*S*,3*R*)-**6b**

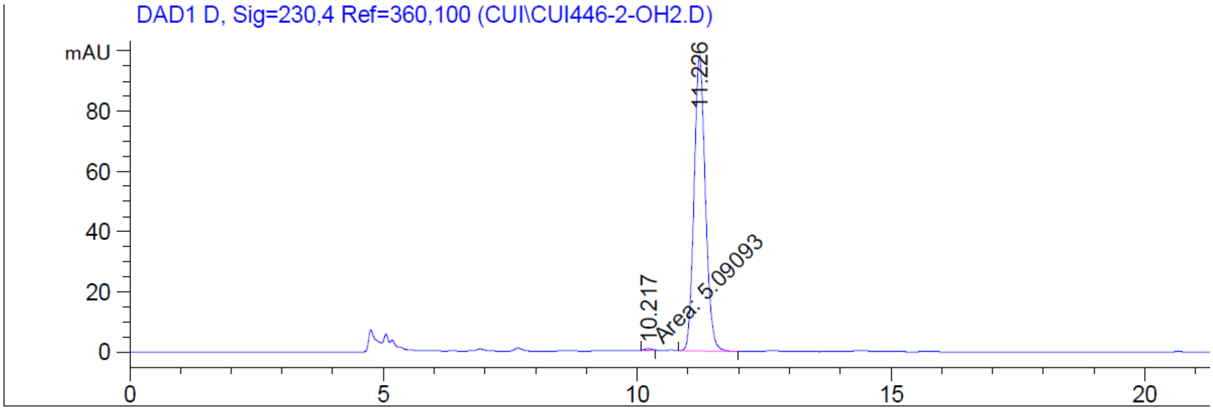

Signal 4: DAD1 D, Sig=230,4 Ref=360,100

Peak #	RetTime [min]	Type	Width [min]	Area [mAU*s]	Height [mAU]	Area %
1	10.217	MM	0.1615	5.09093	5.25432e-1	0.3341
2	11.226	BB	0.2387	1518.73035	98.07388	99.6659

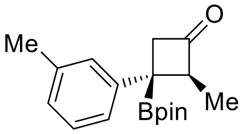

2c

(2*S*,3*R*)-2-Methyl-3-(4,4,5,5-tetramethyl-1,3,2-dioxaborolan-2-yl)-3-(*m*-tolyl)cyclobutan-1-one (2c): The enantiomeric excess of **2c** was determined by HPLC analysis on a chiral stationary phase (*Daicel* Chiralcel IC column, column temperature 20°C, solvent *n*-heptane:*i*-PrOH = 99:1, flow rate 0.5 mL/min): t_R = 24.7 min (major), t_R = 23.6 min (minor).

rac-**2c**

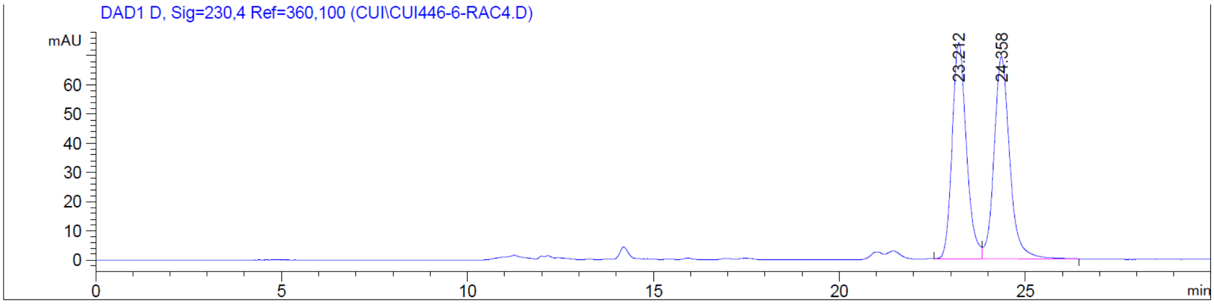

Signal 4: DAD1 D, Sig=230,4 Ref=360,100

Peak #	RetTime [min]	Type	Width [min]	Area [mAU*s]	Height [mAU]	Area %
1	23.212	BV	0.4090	1970.98889	73.93523	49.3009
2	24.358	VB	0.4454	2026.89124	69.25558	50.6991

(2*S*,3*R*)-**2c**

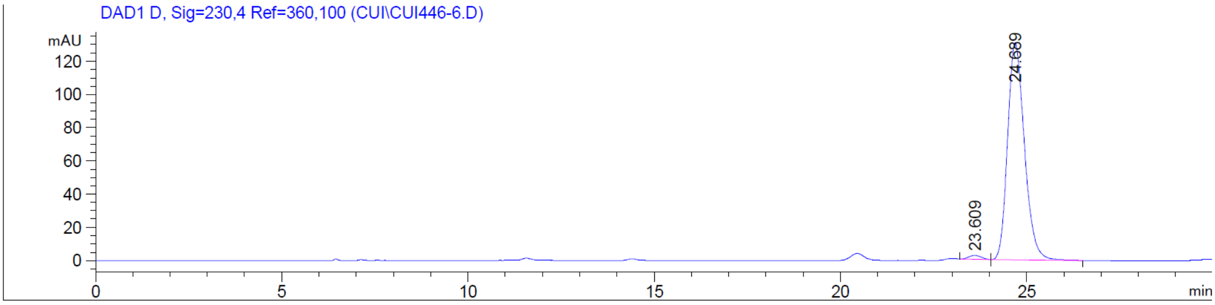

Signal 4: DAD1 D, Sig=230,4 Ref=360,100

Peak #	RetTime [min]	Type	Width [min]	Area [mAU*s]	Height [mAU]	Area %
1	23.609	BB	0.3281	54.66976	2.34381	1.3163
2	24.689	BB	0.4818	4098.47314	130.70654	98.6837

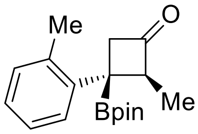

2d

(2*S*,3*R*)-2-Methyl-3-(4,4,5,5-tetramethyl-1,3,2-dioxaborolan-2-yl)-3-(*o*-tolyl)cyclobutan-1-one (2d): The enantiomeric excess of **2d** was determined by HPLC analysis on a chiral stationary phase (*Daicel* Chiralcel IC column, column temperature 20°C, solvent *n*-heptane:*i*-PrOH = 95:5, flow rate 0.7 mL/min): t_R = 13.2 min (major), t_R = 11.3 min (minor).

rac-**2d**

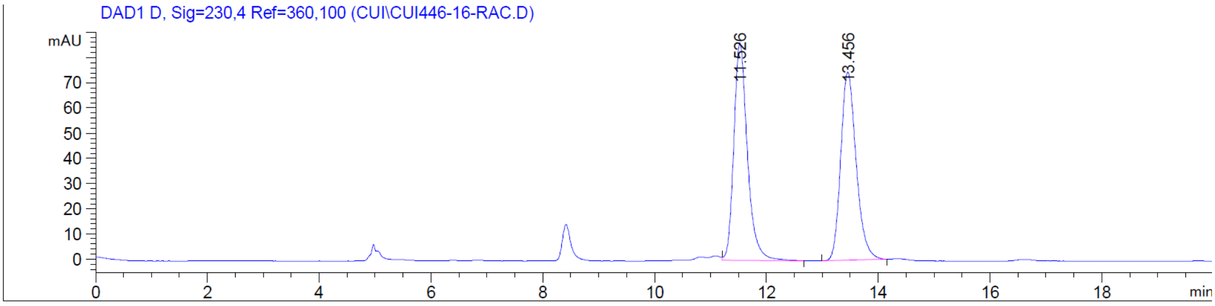

Signal 4: DAD1 D, Sig=230,4 Ref=360,100

Peak #	RetTime [min]	Type	Width [min]	Area [mAU*s]	Height [mAU]	Area %
1	11.526	VB	0.2574	1463.16577	86.47226	50.4861
2	13.456	BB	0.2940	1434.98792	74.75412	49.5139

(2*S*,3*R*)-**2d**

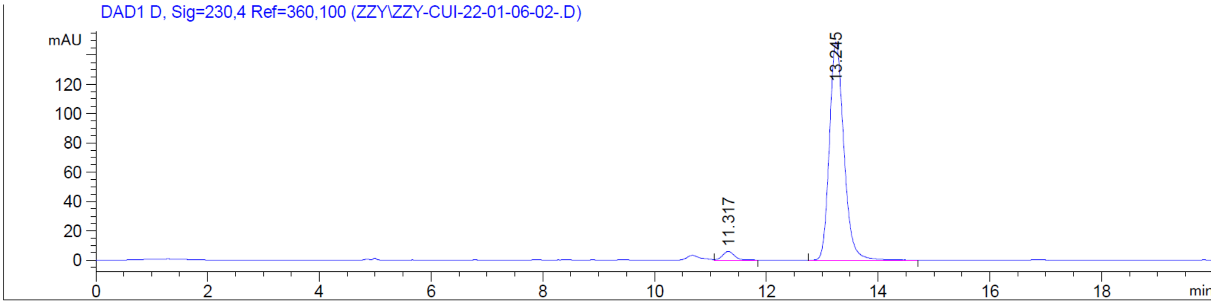

Signal 4: DAD1 D, Sig=230,4 Ref=360,100

Peak #	RetTime [min]	Type	Width [min]	Area [mAU*s]	Height [mAU]	Area %
1	11.317	VB	0.2203	85.75911	5.94591	3.1145
2	13.245	BB	0.2750	2667.74341	148.90494	96.8855

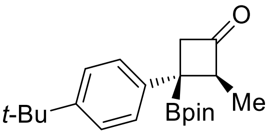

2e

(2*S*,3*R*)-3-(4-(*tert*-Butyl)phenyl)-2-methyl-3-(4,4,5,5-tetramethyl-1,3,2-dioxaborolan-2-yl)cyclobutan-1-one (2e): The enantiomeric excess of **2e** was determined by HPLC analysis on a chiral stationary phase (*Daicel* Chiralcel OD-H column, column temperature 20°C, solvent *n*-heptane:*i*-PrOH = 99:1, flow rate 0.5 mL/min): t_R = 12.4 min (major), t_R = 15.2 min (minor).

rac-**2e**

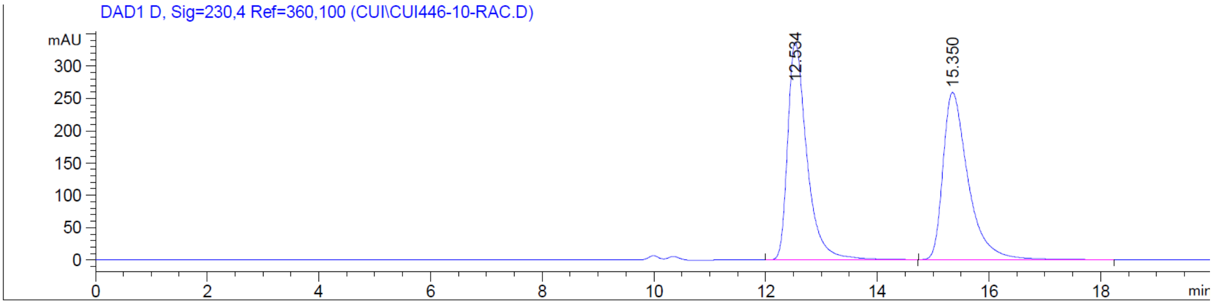

Signal 4: DAD1 D, Sig=230,4 Ref=360,100

Peak #	RetTime [min]	Type	Width [min]	Area [mAU*s]	Height [mAU]	Area %
1	12.534	BB	0.3605	8053.42139	337.32089	49.9006
2	15.350	BB	0.4701	8085.50635	259.08917	50.0994

(2*S*,3*R*)-**2e**

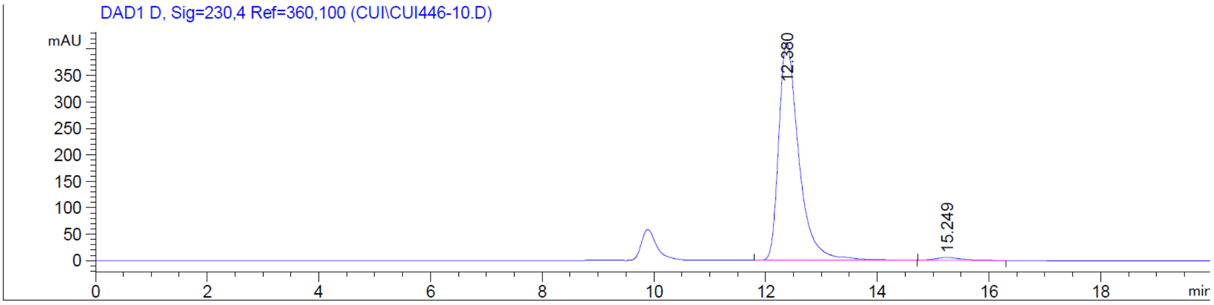

Signal 4: DAD1 D, Sig=230,4 Ref=360,100

Peak #	RetTime [min]	Type	Width [min]	Area [mAU*s]	Height [mAU]	Area %
1	12.380	BB	0.3774	1.02766e4	411.37555	98.4994
2	15.249	BB	0.4318	156.55995	5.22176	1.5006

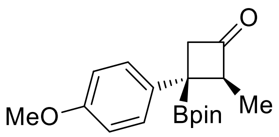

2f

(2*S*,3*R*)-3-(4-Methoxyphenyl)-2-methyl-3-(4,4,5,5-tetramethyl-1,3,2-dioxaborolan-2-yl)cyclobutan-1-one (2f): The enantiomeric excess of **2f** was determined by HPLC analysis on a chiral stationary phase (*Daicel* Chiralcel IC column, column temperature 20°C, solvent *n*-heptane:*i*-PrOH = 95:5, flow rate 0.7 mL/min): t_R = 18.5 min (major), t_R = 16.1 min (minor).

rac-**2f**

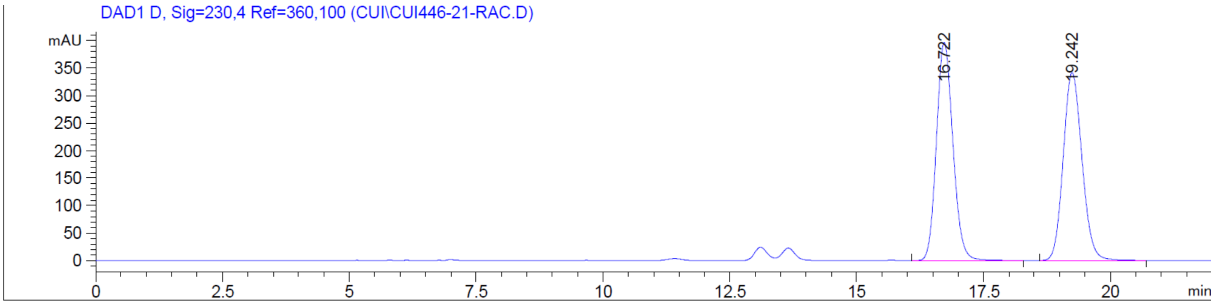

Signal 4: DAD1 D, Sig=230,4 Ref=360,100

Peak #	RetTime [min]	Type	Width [min]	Area [mAU*s]	Height [mAU]	Area %
1	16.722	BB	0.3488	8935.12207	396.68396	50.4819
2	19.242	BB	0.3992	8764.52051	341.72827	49.5181

(2*S*,3*R*)-**2f**

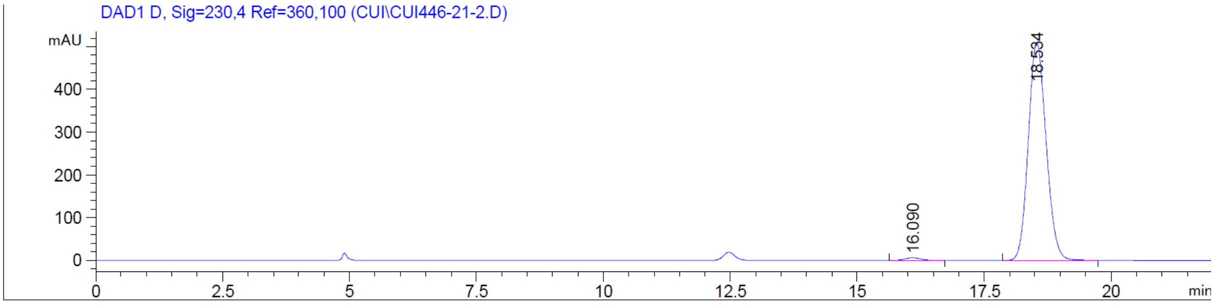

Signal 4: DAD1 D, Sig=230,4 Ref=360,100

Peak #	RetTime [min]	Type	Width [min]	Area [mAU*s]	Height [mAU]	Area %
1	16.090	BB	0.3285	129.76785	6.24392	1.0292
2	18.534	BB	0.3812	1.24793e4	510.42203	98.9708

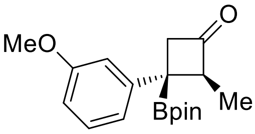

2g

(2*S*,3*R*)-3-(3-Methoxyphenyl)-2-methyl-3-(4,4,5,5-tetramethyl-1,3,2-dioxaborolan-2-yl)cyclobutan-1-one (2g): The enantiomeric excess of **2g** was determined by HPLC analysis on a chiral stationary phase (*Daicel* Chiralcel IC column, column temperature 20°C, solvent *n*-heptane:*i*-PrOH = 95:5, flow rate 0.7 mL/min): t_R = 16.4 min (major), t_R = 14.6 min (minor).

rac-**2g**

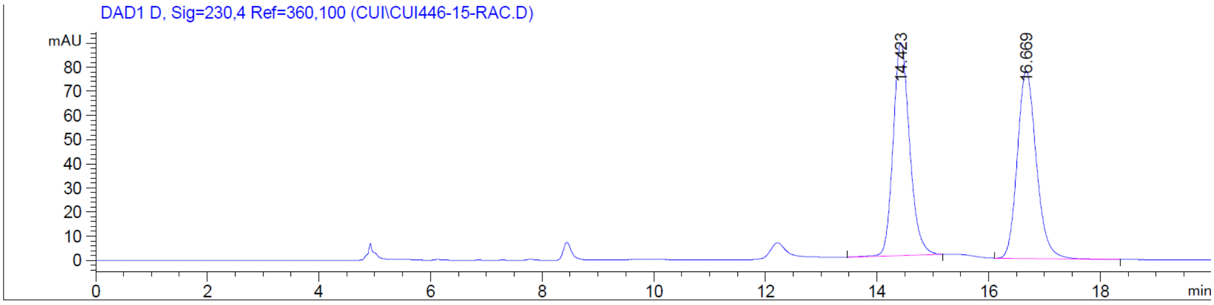

Signal 4: DAD1 D, Sig=230,4 Ref=360,100

Peak #	RetTime [min]	Type	Width [min]	Area [mAU*s]	Height [mAU]	Area %
1	14.423	BB	0.3132	1807.19739	88.15385	49.6018
2	16.669	BB	0.3636	1836.21411	77.74442	50.3982

(2*S*,3*R*)-**2g**

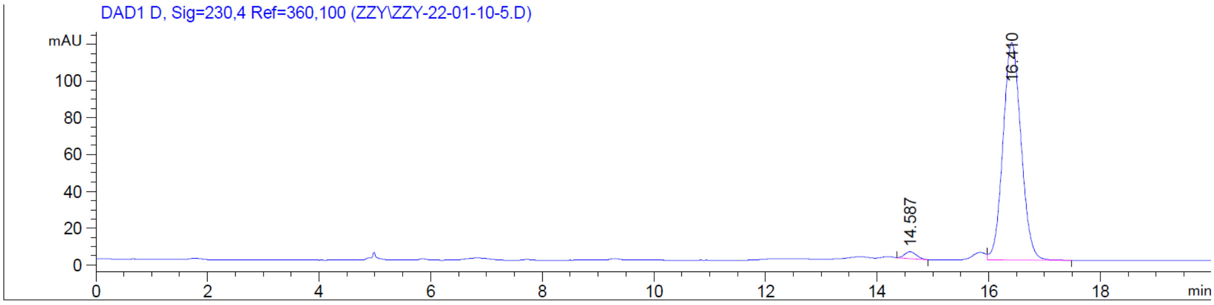

Signal 4: DAD1 D, Sig=230,4 Ref=360,100

Peak #	RetTime [min]	Type	Width [min]	Area [mAU*s]	Height [mAU]	Area %
1	14.587	BB	0.2257	52.17590	3.63011	1.9279
2	16.410	VB	0.3481	2654.13916	118.15330	98.0721

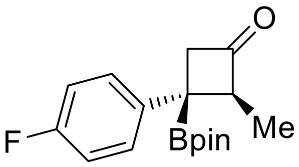

2h

(2*S*,3*R*)-3-(4-Fluorophenyl)-2-methyl-3-(4,4,5,5-tetramethyl-1,3,2-dioxaborolan-2-yl)cyclobutan-1-one (2h): The enantiomeric excess of **2h** was determined by HPLC analysis on a chiral stationary phase (*Daicel* Chiralcel IC column, column temperature 20°C, solvent *n*-heptane:*i*-PrOH = 99:1, flow rate 0.5 mL/min): t_R = 31.5 min (major), t_R = 26.5 min (minor).

rac-**2h**

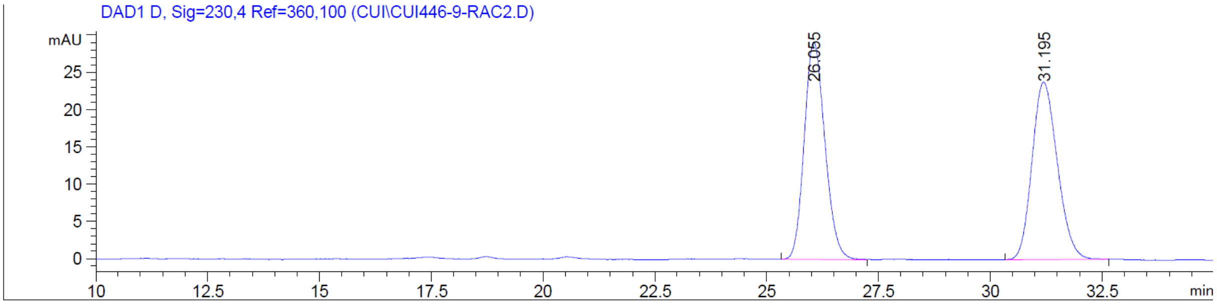

Signal 4: DAD1 D, Sig=230,4 Ref=360,100

Peak #	RetTime [min]	Type	Width [min]	Area [mAU*s]	Height [mAU]	Area %
1	26.055	BB	0.5031	938.80579	29.19608	49.9778
2	31.195	BB	0.6019	939.63965	23.83469	50.0222

(2*S*,3*R*)-**2h**

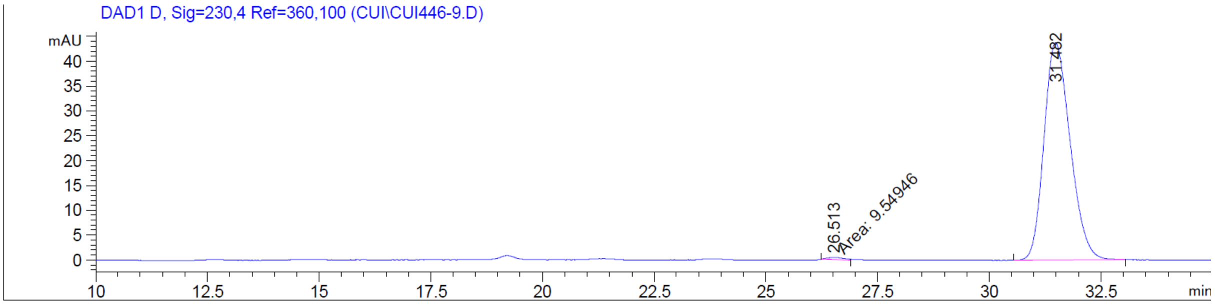

Signal 4: DAD1 D, Sig=230,4 Ref=360,100

Peak #	RetTime [min]	Type	Width [min]	Area [mAU*s]	Height [mAU]	Area %
1	26.513	MM	0.4095	9.54946	3.88703e-1	0.5325
2	31.482	BB	0.6240	1783.64673	43.72118	99.4675

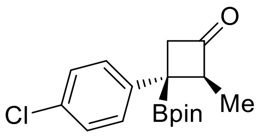

2i

(2*S*,3*R*)-3-(4-Chlorophenyl)-2-methyl-3-(4,4,5,5-tetramethyl-1,3,2-dioxaborolan-2-yl)cyclobutan-1-one (2i): The enantiomeric excess of **2i** was determined by HPLC analysis on a chiral stationary phase (*Daicel* Chiralcel IC column, column temperature 20°C, solvent *n*-heptane:*i*-PrOH = 99:1, flow rate 0.5 mL/min): t_R = 34.9 min (major), t_R = 28.7 min (minor).

rac-**2i**

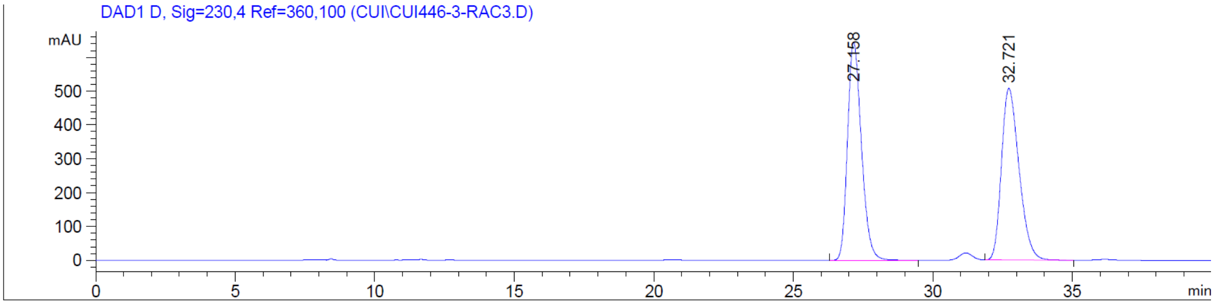

Signal 4: DAD1 D, Sig=230,4 Ref=360,100

Peak #	RetTime [min]	Type	Width [min]	Area [mAU*s]	Height [mAU]	Area %
1	27.158	BB	0.5312	2.22895e4	644.91461	50.0763
2	32.721	BB	0.6769	2.22216e4	507.44296	49.9237

(2*S*,3*R*)-**2i**

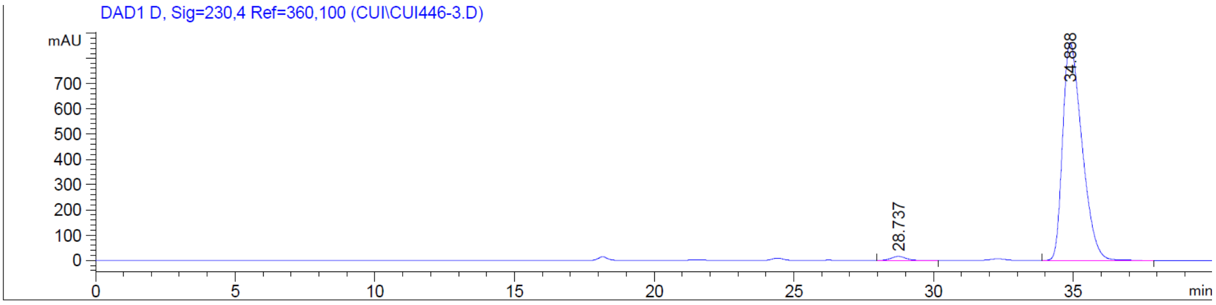

Signal 4: DAD1 D, Sig=230,4 Ref=360,100

Peak #	RetTime [min]	Type	Width [min]	Area [mAU*s]	Height [mAU]	Area %
1	28.737	BB	0.5500	549.91974	15.34839	1.3131
2	34.888	BB	0.7339	4.13300e4	861.21997	98.6869

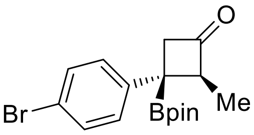

2j

(2*S*,3*R*)-3-(4-Bromophenyl)-2-methyl-3-(4,4,5,5-tetramethyl-1,3,2-dioxaborolan-2-yl)cyclobutan-1-one (2j): The enantiomeric excess of **2j** was determined by HPLC analysis on a chiral stationary phase (*Daicel* Chiralcel IC column, column temperature 20°C, solvent *n*-heptane:*i*-PrOH = 99:1, flow rate 0.5 mL/min): t_R = 36.8 min (major), t_R = 30.5 min (minor).

rac-**2j**

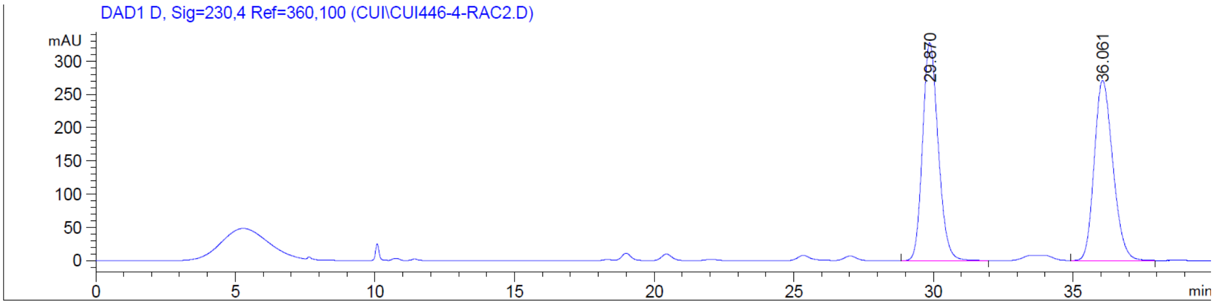

Signal 4: DAD1 D, Sig=230,4 Ref=360,100

Peak #	RetTime [min]	Type	Width [min]	Area [mAU*s]	Height [mAU]	Area %
1	29.870	BB	0.6005	1.27018e4	327.48004	50.3490
2	36.061	BB	0.7179	1.25257e4	270.67657	49.6510

(2*S*,3*R*)-**2j**

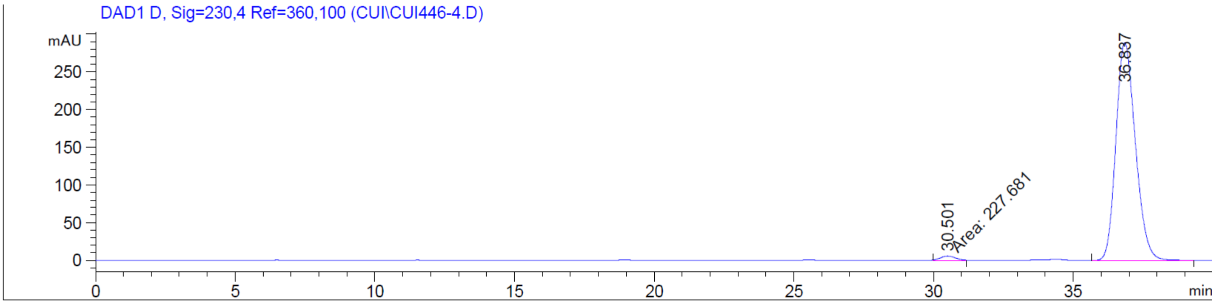

Signal 4: DAD1 D, Sig=230,4 Ref=360,100

Peak #	RetTime [min]	Type	Width [min]	Area [mAU*s]	Height [mAU]	Area %
1	30.501	MM	0.6371	227.68138	5.95641	1.6028
2	36.837	BB	0.7505	1.39775e4	288.86517	98.3972

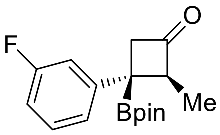

2k

(2*S*,3*R*)-3-(3-Fluorophenyl)-2-methyl-3-(4,4,5,5-tetramethyl-1,3,2-dioxaborolan-2-yl)cyclobutan-1-one (2k): The enantiomeric excess of **2k** was determined by HPLC analysis on a chiral stationary phase (*Daicel* Chiralcel OD-H column, column temperature 20°C, solvent *n*-heptane:*i*-PrOH = 99:1, flow rate 0.5 mL/min): t_R = 14.5 min (major), t_R = 17.8 min (minor).

rac-**2k**

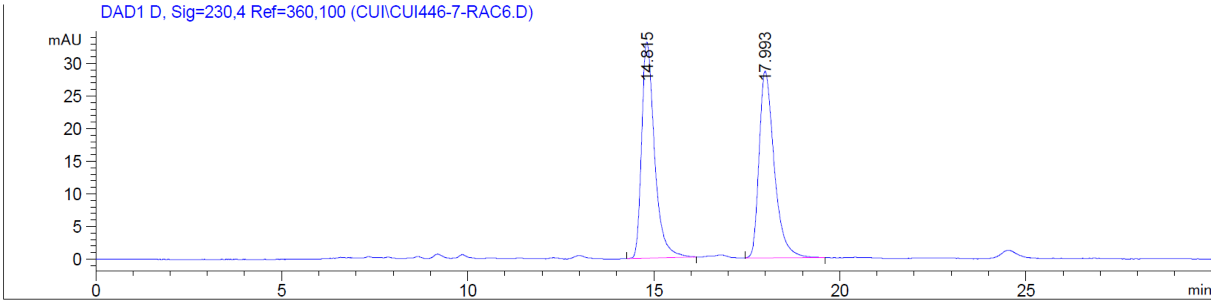


```
Signal 4: DAD1 D, Sig=230,4 Ref=360,100
```

Peak #	RetTime [min]	Type	Width [min]	Area [mAU*s]	Height [mAU]	Area %
1	14.815	BB	0.3632	798.33606	33.12640	49.6405
2	17.993	BB	0.4321	809.90051	28.62207	50.3595

(2*S*,3*R*)-**2k**

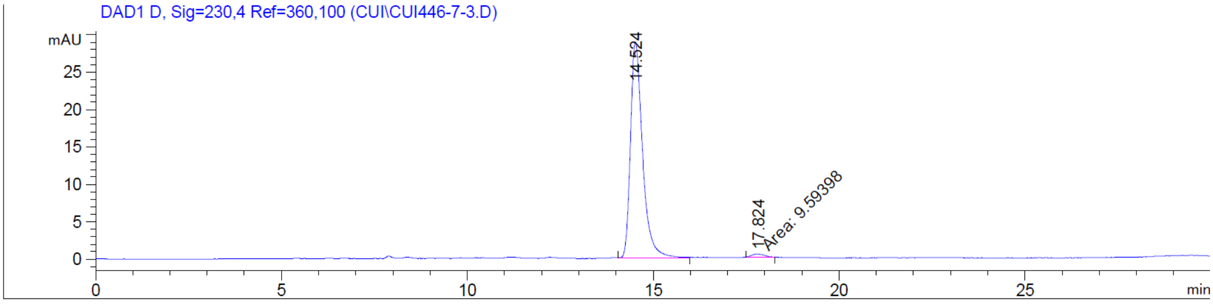


```
Signal 4: DAD1 D, Sig=230,4 Ref=360,100
```

Peak #	RetTime [min]	Type	Width [min]	Area [mAU*s]	Height [mAU]	Area %
1	14.524	BB	0.3400	648.21265	28.86552	98.5415
2	17.824	MM	0.3890	9.59398	4.11007e-1	1.4585

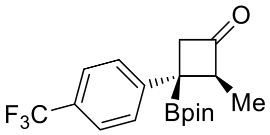

2l

(2*S*,3*R*)-2-Methyl-3-(4,4,5,5-tetramethyl-1,3,2-dioxaborolan-2-yl)-3-(4-(trifluoromethyl)phenyl)cyclobutan-1-one (2l): The enantiomeric excess of **2l** was determined by HPLC analysis on a chiral stationary phase (*Daicel* Chiralcel IC column, column

temperature 20°C, solvent *n*-heptane:*i*-PrOH = 99:1, flow rate 0.5 mL/min): t_R = 22.4 min (major), t_R = 20.7 min (minor).

rac-**2l**

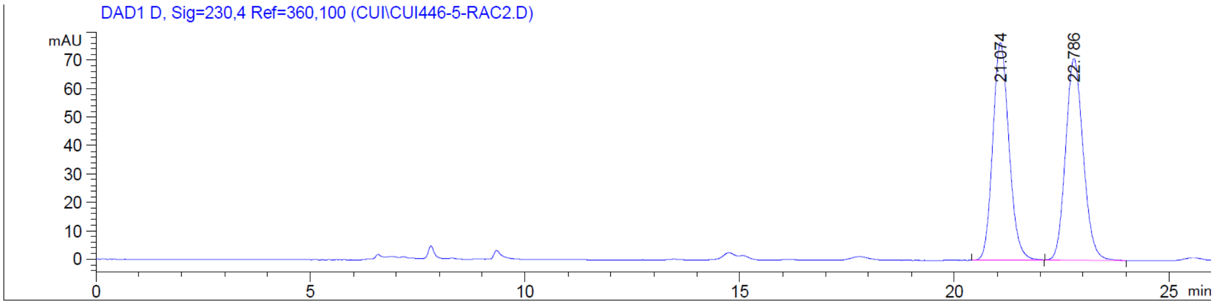

Signal 4: DAD1 D, Sig=230,4 Ref=360,100

Peak #	RetTime [min]	Type	Width [min]	Area [mAU*s]	Height [mAU]	Area %
1	21.074	BB	0.4069	2012.20935	76.49278	49.8153
2	22.786	BB	0.4395	2027.12817	70.91780	50.1847

(2*S*,3*R*)-**2l**

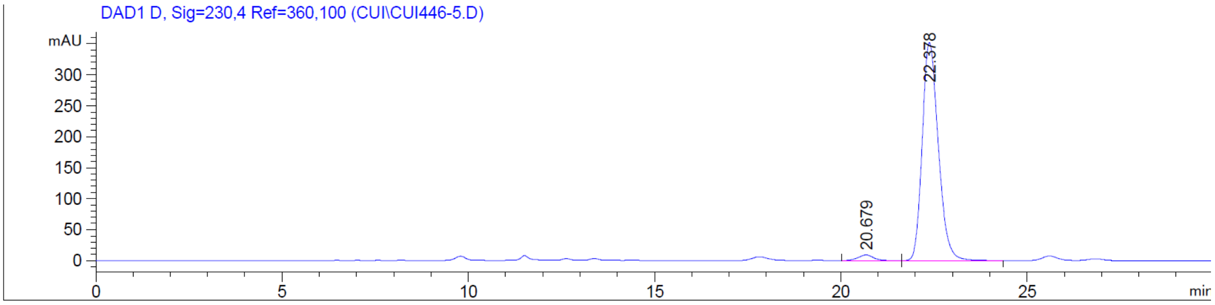

Signal 4: DAD1 D, Sig=230,4 Ref=360,100

Peak #	RetTime [min]	Type	Width [min]	Area [mAU*s]	Height [mAU]	Area %
1	20.679	BB	0.4293	254.31567	8.90104	2.3559
2	22.378	BB	0.4637	1.05407e4	351.66489	97.6441

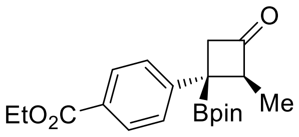

2m

Ethyl 4-((1*R*,2*S*)-2-methyl-3-oxo-1-(4,4,5,5-tetramethyl-1,3,2-dioxaborolan-2-yl)cyclobutyl)benzoate (2m): The enantiomeric excess of **2m** was determined by HPLC analysis on a chiral stationary phase (*Daicel* Chiralcel IC column, column temperature 20°C, solvent *n*-heptane:*i*-PrOH = 90:10, flow rate 0.5 mL/min): t_R = 38.7 min (major), t_R = 36.4 min (minor).

rac-**2m**

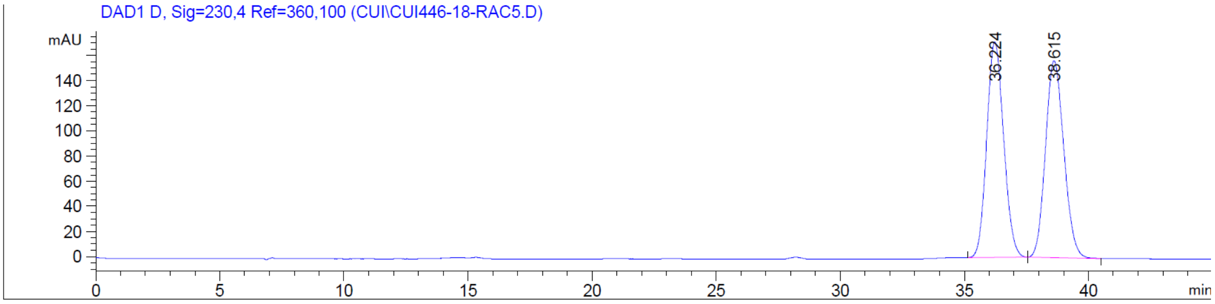

Signal 4: DAD1 D, Sig=230,4 Ref=360,100

Peak #	RetTime [min]	Type	Width [min]	Area [mAU*s]	Height [mAU]	Area %
1	36.224	BB	0.7370	8127.54541	171.48152	50.1420
2	38.615	BB	0.8054	8081.50830	156.77238	49.8580

(1*R*,2*S*)-**2m**

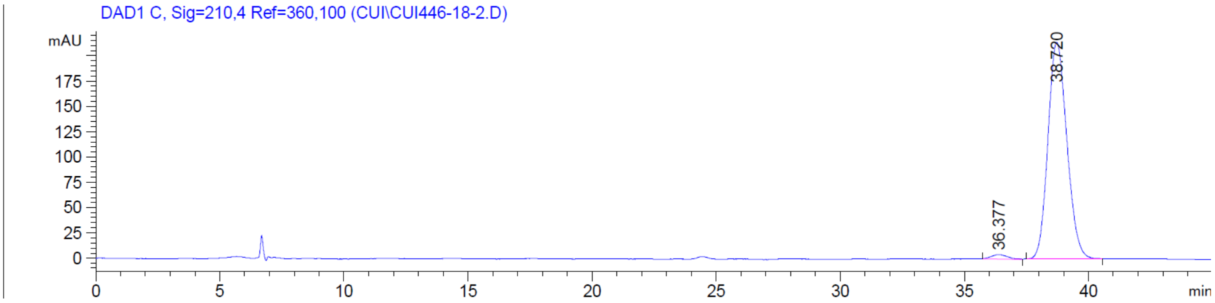

Signal 3: DAD1 C, Sig=210,4 Ref=360,100

Peak #	RetTime [min]	Type	Width [min]	Area [mAU*s]	Height [mAU]	Area %
1	36.377	BB	0.5080	177.30275	4.17026	1.5409
2	38.720	BB	0.8097	1.13292e4	213.97951	98.4591

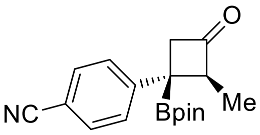

2n

4-((1*R*,2*S*)-2-Methyl-3-oxo-1-(4,4,5,5-tetramethyl-1,3,2-dioxaborolan-2-yl)cyclobutyl)benzonitrile (2n): The enantiomeric excess of **2n** was determined by HPLC analysis on a chiral stationary phase (*Daicel* Chiralcel IC column, column temperature 20°C, solvent *n*-heptane:*i*-PrOH = 90:10, flow rate 0.8 mL/min): t_R = 47.6 min (major), t_R = 39.8 min (minor).

rac-**2n**

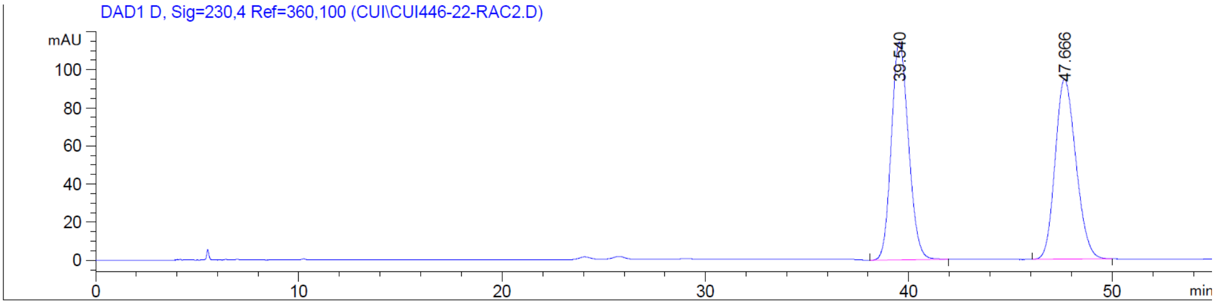

Signal 4: DAD1 D, Sig=230,4 Ref=360,100

Peak #	RetTime [min]	Type	Width [min]	Area [mAU*s]	Height [mAU]	Area %
1	39.540	BB	0.8979	6704.60498	114.37919	49.9246
2	47.666	BB	1.0757	6724.84619	94.55680	50.0754

(1*R*,2*S*)-**2n**

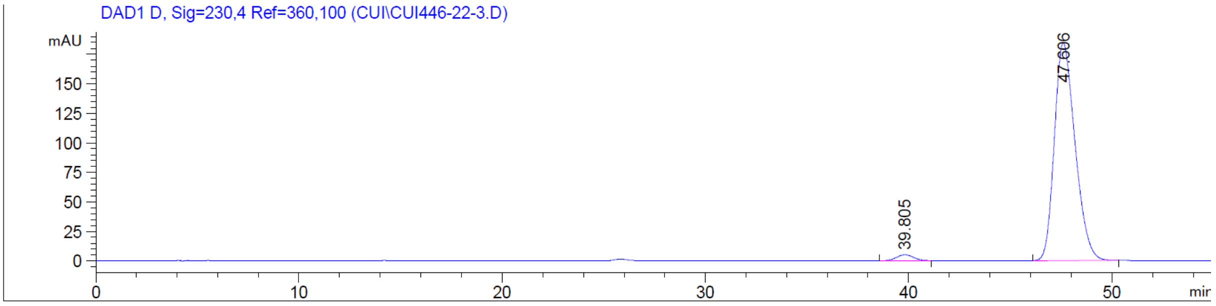

Signal 4: DAD1 D, Sig=230,4 Ref=360,100

Peak #	RetTime [min]	Type	Width [min]	Area [mAU*s]	Height [mAU]	Area %
1	39.805	BB	0.6989	293.20694	5.05368	2.1945
2	47.606	BB	1.0745	1.30678e4	184.47121	97.8055

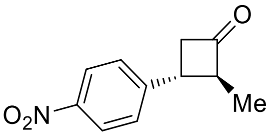

3o

$C_{11}H_{11}NO_3$

M = 205.21 g/mol

(2*S*,3*R*)-2-Methyl-3-(4-nitrophenyl)cyclobutan-1-one (3o): The enantiomeric excess of **3o** was determined by HPLC analysis on a chiral stationary phase (*Daicel* Chiralcel IC column, column temperature 20°C, solvent *n*-heptane:*i*-PrOH = 90:10, flow rate 0.6 mL/min): t_R = 72.4 min (major), t_R = 63.5 min (minor).

rac-**3o**

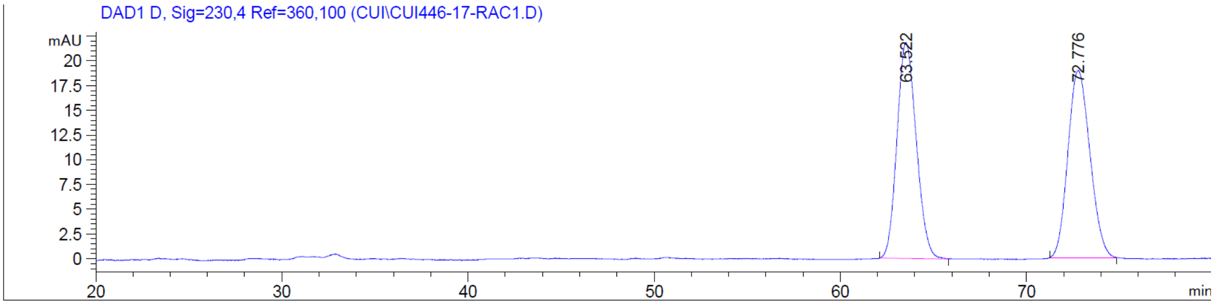

Signal 4: DAD1 D, Sig=230,4 Ref=360,100

Peak #	RetTime [min]	Type	Width [min]	Area [mAU*s]	Height [mAU]	Area %
1	63.522	BB	0.8757	1570.05286	21.81354	50.1546
2	72.776	BB	0.9834	1560.37207	18.93616	49.8454

(2*S*,3*R*)-**3o**

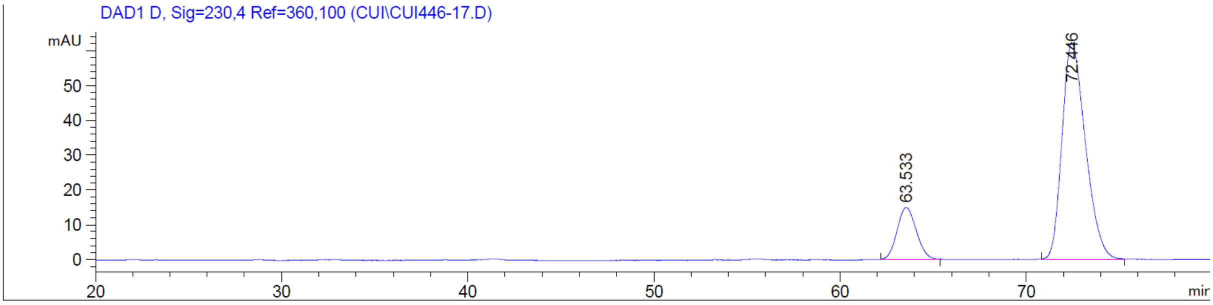

Signal 4: DAD1 D, Sig=230,4 Ref=360,100

Peak #	RetTime [min]	Type	Width [min]	Area [mAU*s]	Height [mAU]	Area %
1	63.533	BB	0.8630	1065.32007	14.88490	16.4495
2	72.446	BB	1.2196	5410.98584	62.15604	83.5505

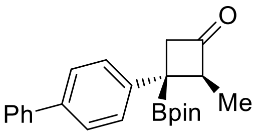

2p
$C_{23}H_{27}BO_3$
M = 362.28 g/mol

(2*S*,3*R*)-3-([1,1'-Biphenyl]-4-yl)-2-methyl-3-(4,4,5,5-tetramethyl-1,3,2-dioxaborolan-2-yl)cyclobutan-1-one (2p): The enantiomeric excess of **2p** was determined by HPLC analysis on a chiral stationary phase (*Daicel* Chiralcel OD-H column, column temperature 20°C, solvent *n*-heptane:*i*-PrOH = 99:1, flow rate 0.3 mL/min): t_R = 55.8 min (major), t_R = 60.9 min (minor).

rac-**2p**

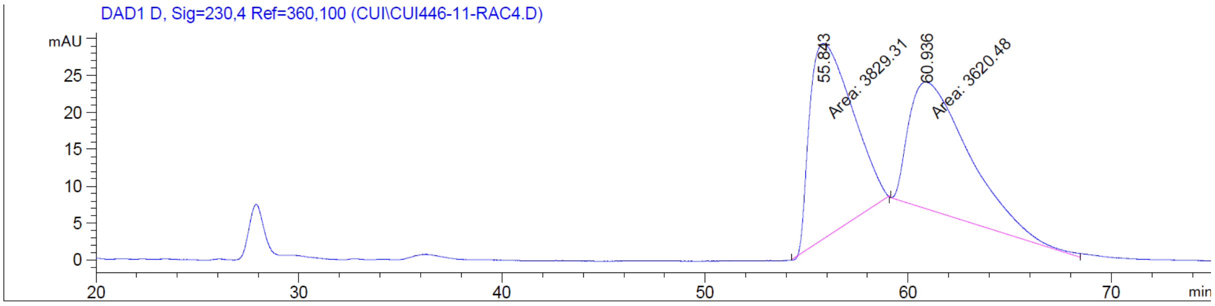

Signal 4: DAD1 D, Sig=230,4 Ref=360,100

Peak #	RetTime [min]	Type	Width [min]	Area [mAU*s]	Height [mAU]	Area %
1	55.843	MM	1.7068	3829.31201	26.46959	51.4016
2	60.936	MM	3.5070	3620.48193	17.20591	48.5984

(2*S*,3*R*)-**2p**

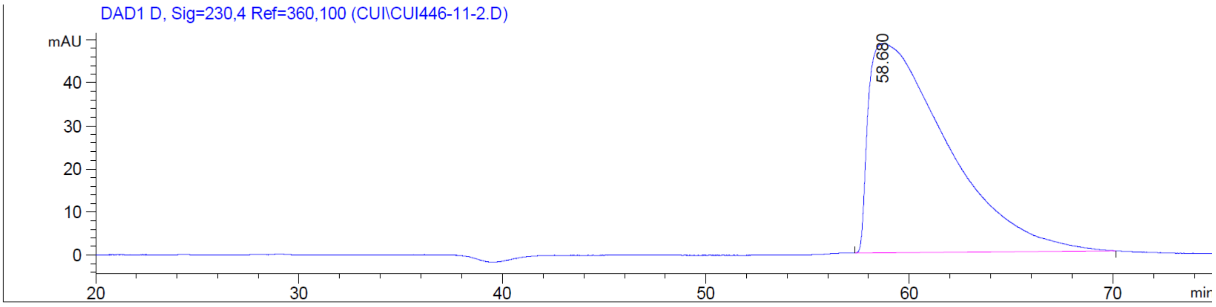

Signal 4: DAD1 D, Sig=230,4 Ref=360,100

Peak #	RetTime [min]	Type	Width [min]	Area [mAU*s]	Height [mAU]	Area %
1	58.680	BB	3.1837	1.31701e4	48.56632	100.0000

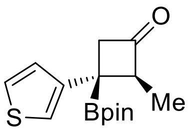

2q

(2*S*,3*R*)-2-Methyl-3-(4,4,5,5-tetramethyl-1,3,2-dioxaborolan-2-yl)-3-(thiophen-3-yl)cyclobutan-1-one (2q): The enantiomeric excess of **2q** was determined by HPLC analysis on a chiral stationary phase (*Daicel* Chiralcel IB column, column temperature 20°C, solvent *n*-heptane:*i*-PrOH = 99:1, flow rate 0.5 mL/min): t_R = 12.5 min (major), t_R = 12.0 min (minor).

rac-**2q**

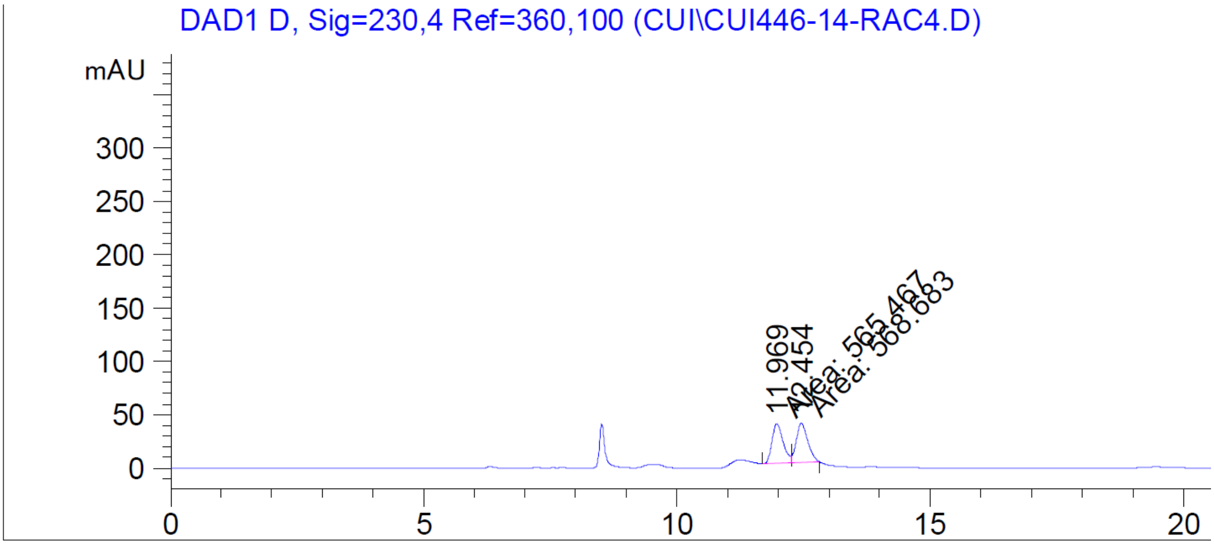

Signal 4: DAD1 D, Sig=230,4 Ref=360,100

Peak #	RetTime [min]	Type	Width [min]	Area [mAU*s]	Height [mAU]	Area %
1	11.969	MF	0.2547	565.46686	36.99973	49.8582
2	12.454	FM	0.2589	568.68335	36.61331	50.1418

(2*S*,3*R*)-**2q**

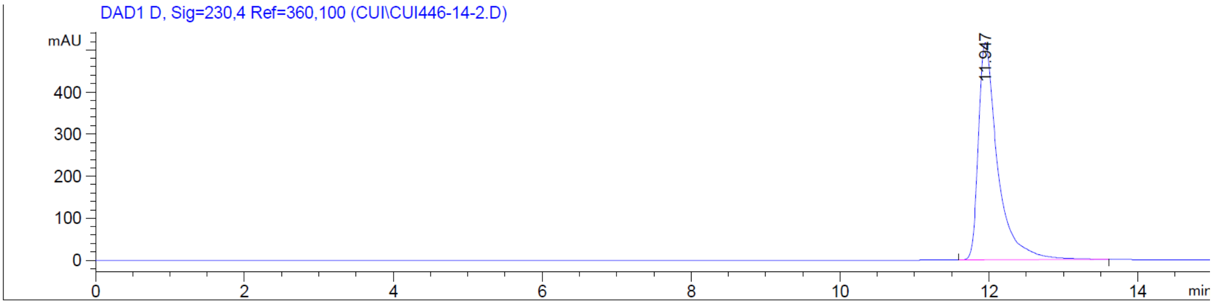

Signal 4: DAD1 D, Sig=230,4 Ref=360,100

Peak #	RetTime [min]	Type	Width [min]	Area [mAU*s]	Height [mAU]	Area %
1	11.947	BB	0.2608	9248.23633	516.77606	100.0000

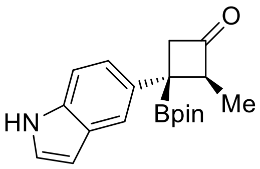

2r
$C_{19}H_{24}BNO_3$
M = 325.22 g/mol

(2*S*,3*R*)-3-(1H-Indol-5-yl)-2-methyl-3-(4,4,5,5-tetramethyl-1,3,2-dioxaborolan-2-yl)cyclobutan-1-one (2r): The enantiomeric excess of **2r** was determined by HPLC analysis on a chiral stationary phase (*Daicel* Chiralcel IC column, column temperature 20°C, solvent *n*-heptane:*i*-PrOH = 95:5, flow rate 0.8 mL/min): t_R = 35.2 min (major), t_R = 29.3 min (minor).

rac-**2r**

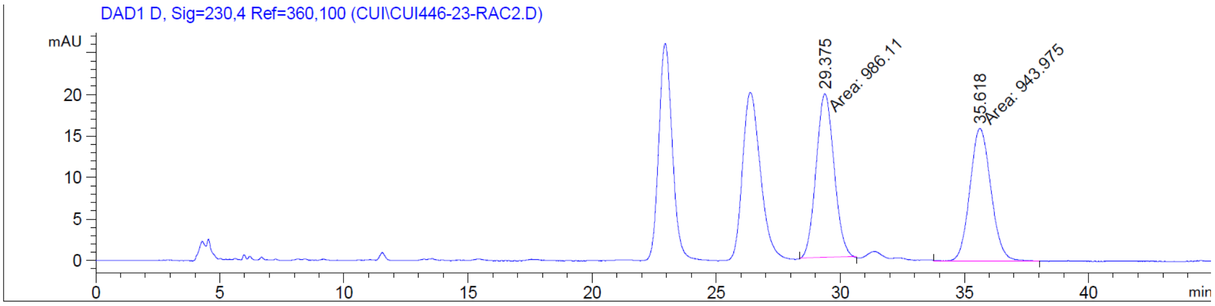

Signal 4: DAD1 D, Sig=230,4 Ref=360,100

Peak #	RetTime [min]	Type	Width [min]	Area [mAU*s]	Height [mAU]	Area %
1	29.375	MM	0.8353	986.10986	19.67601	51.0915
2	35.618	MM	0.9850	943.97522	15.97320	48.9085

(2*S*,3*R*)-**2r**

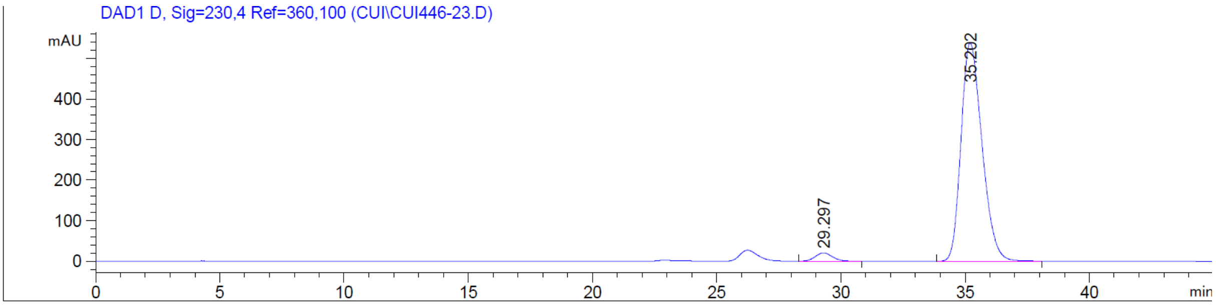

Signal 4: DAD1 D, Sig=230,4 Ref=360,100

Peak #	RetTime [min]	Type	Width [min]	Area [mAU*s]	Height [mAU]	Area %
1	29.297	BB	0.6850	970.38916	20.47309	2.9879
2	35.202	BB	0.9012	3.15064e4	537.99103	97.0121

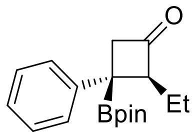

2s

$C_{18}H_{25}BO_3$

M = 300.21 g/mol

(2*S*,3*R*)-2-Ethyl-3-phenyl-3-(4,4,5,5-tetramethyl-1,3,2-dioxaborolan-2-yl)cyclobutan-1-one (2s): The enantiomeric excess of **2s** was determined by HPLC analysis on a chiral stationary phase (*Daicel* Chiralcel IC column, column temperature 20°C, solvent *n*-heptane:*i*-PrOH = 95:5, flow rate 0.7 mL/min): t_R = 9.2 min (major), t_R = 8.5 min (minor).

rac-**2s**

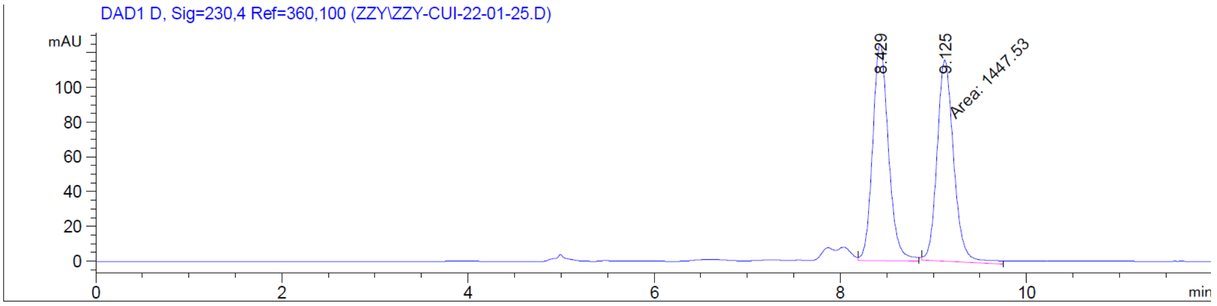

Signal 4: DAD1 D, Sig=230,4 Ref=360,100

Peak #	RetTime [min]	Type	Width [min]	Area [mAU*s]	Height [mAU]	Area %
1	8.429	VV	0.1801	1480.52344	125.09821	50.5635
2	9.125	MM	0.2082	1447.52588	115.88146	49.4365

(2*S*,3*R*)-**2s**

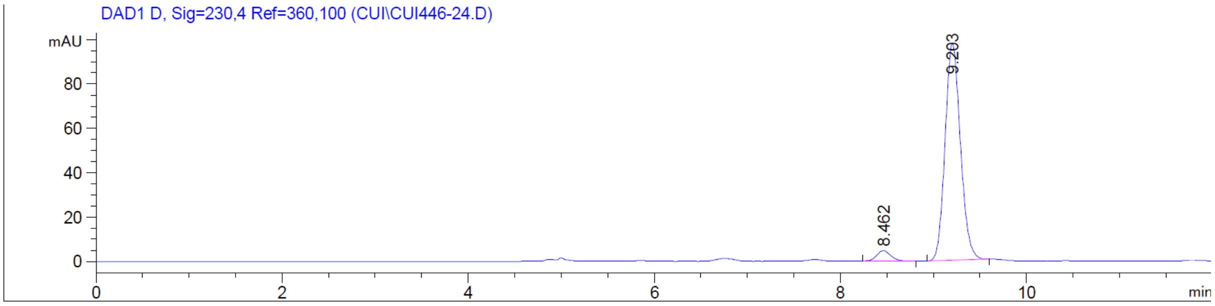

Signal 4: DAD1 D, Sig=230,4 Ref=360,100

Peak #	RetTime [min]	Type	Width [min]	Area [mAU*s]	Height [mAU]	Area %
1	8.462	BB	0.1634	49.06311	4.71359	4.1993
2	9.203	BB	0.1776	1119.30518	97.75955	95.8007

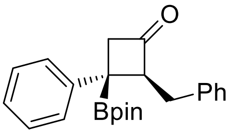

2t

(2*S*,3*R*)-2-Benzyl-3-phenyl-3-(4,4,5,5-tetramethyl-1,3,2-dioxaborolan-2-yl)cyclobutan-1-one (2t): The enantiomeric excess of **2t** was determined by HPLC analysis on a chiral stationary phase (*Daicel* Chiralcel IB column, column temperature 20°C, solvent *n*-heptane:*i*-PrOH = 99:1, flow rate 0.5 mL/min): t_R = 13.8 min (major), t_R = 12.3 min (minor).

rac-**2t**

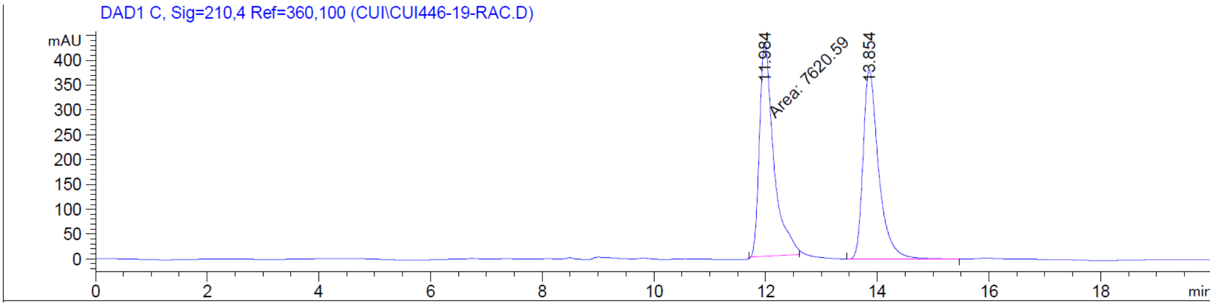

Signal 4: DAD1 D, Sig=230,4 Ref=360,100

Peak #	RetTime [min]	Type	Width [min]	Area [mAU*s]	Height [mAU]	Area %
1	11.984	BB	0.2904	1014.40948	50.05337	54.4089
2	13.855	BB	0.2892	850.00873	44.02662	45.5911

(2*S*,3*R*)-**2t**

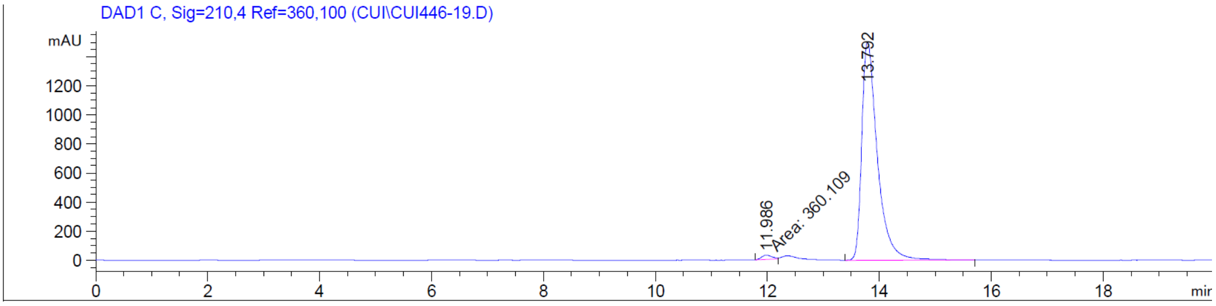

Signal 3: DAD1 C, Sig=210,4 Ref=360,100

Peak #	RetTime [min]	Type	Width [min]	Area [mAU*s]	Height [mAU]	Area %
1	11.986	MM	0.2085	360.10925	28.78420	1.2257
2	13.792	BB	0.2899	2.90196e4	1498.83582	98.7743

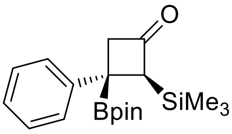

2u

(2*S*,3*S*)-3-Phenyl-3-(4,4,5,5-tetramethyl-1,3,2-dioxaborolan-2-yl)-2-(trimethylsilyl)cyclobutan-1-one (2u): The enantiomeric excess of **2u** was determined by HPLC analysis on a chiral stationary phase (*Daicel* Chiralcel IC column, column temperature 20°C, solvent *n*-heptane:*i*-PrOH = 99:1, flow rate 0.5 mL/min): t_R = 23.3 min (major), t_R = 21.1 min (minor).

rac-**2u**

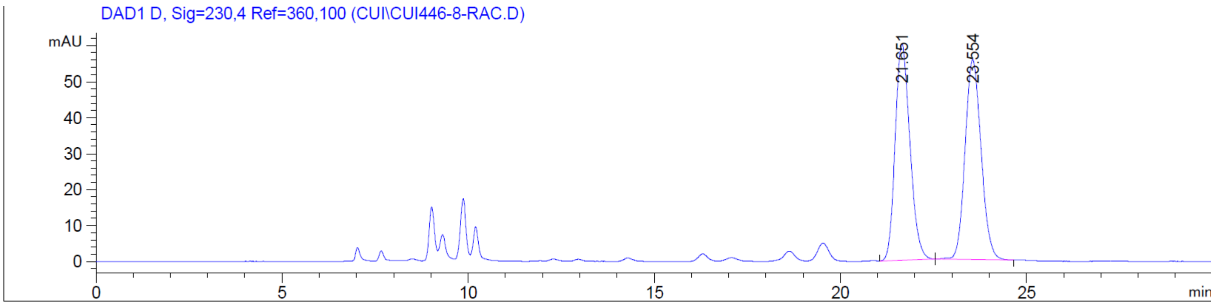

Signal 4: DAD1 D, Sig=230,4 Ref=360,100

Peak #	RetTime [min]	Type	Width [min]	Area [mAU*s]	Height [mAU]	Area %
1	21.651	BB	0.4250	1657.91345	60.26405	49.8342
2	23.554	BB	0.4656	1668.94458	55.69206	50.1658

(2*S*,3*S*)-**2u**

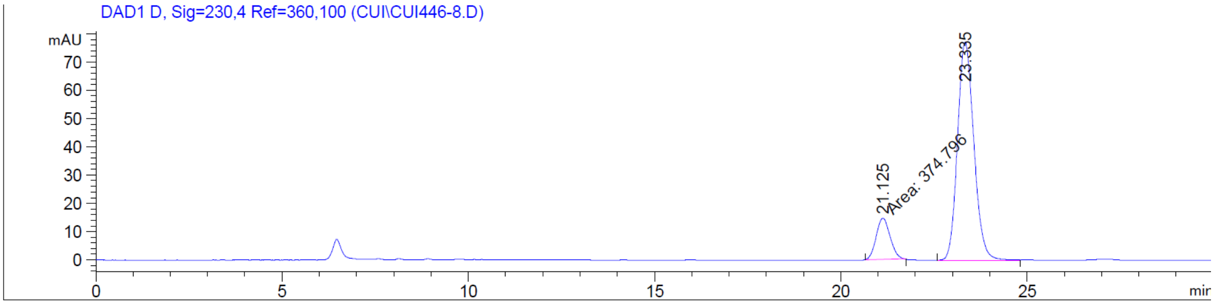

Signal 4: DAD1 D, Sig=230,4 Ref=360,100

Peak #	RetTime [min]	Type	Width [min]	Area [mAU*s]	Height [mAU]	Area %
1	21.125	MM	0.4300	374.79550	14.52641	13.7666
2	23.335	BB	0.4751	2347.70020	77.12996	86.2334

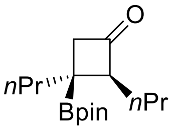

2v

$C_{16}H_{29}BO_3$

M = 280.22 g/mol

(2*S*,3*R*)-2,3-Dipropyl-3-(4,4,5,5-tetramethyl-1,3,2-dioxaborolan-2-yl)cyclobutan-1-one (2v): The enantiomeric excess of **2v** was determined by HPLC analysis on a chiral stationary phase (*Daicel* Chiralcel IC column, column temperature 20°C, solvent *n*-heptane:*i*-PrOH = 99:1, flow rate 0.5 mL/min): t_R = 16.6 min (major), t_R = 15.9 min (minor).

rac-**2v**

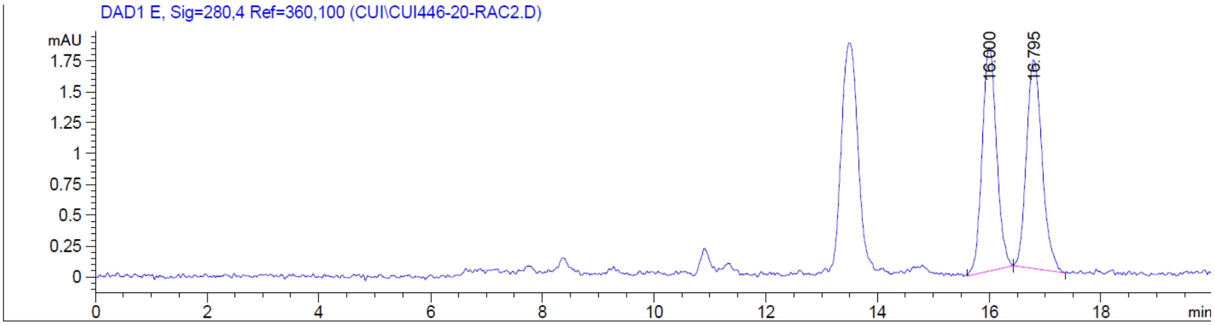

Signal 5: DAD1 E, Sig=280,4 Ref=360,100

Peak #	RetTime [min]	Type	Width [min]	Area [mAU*s]	Height [mAU]	Area %
1	16.000	BB	0.2556	32.51572	1.80982	49.6810
2	16.795	BB	0.2854	32.93324	1.68989	50.3190

(2*S*,3*R*)-**2v**

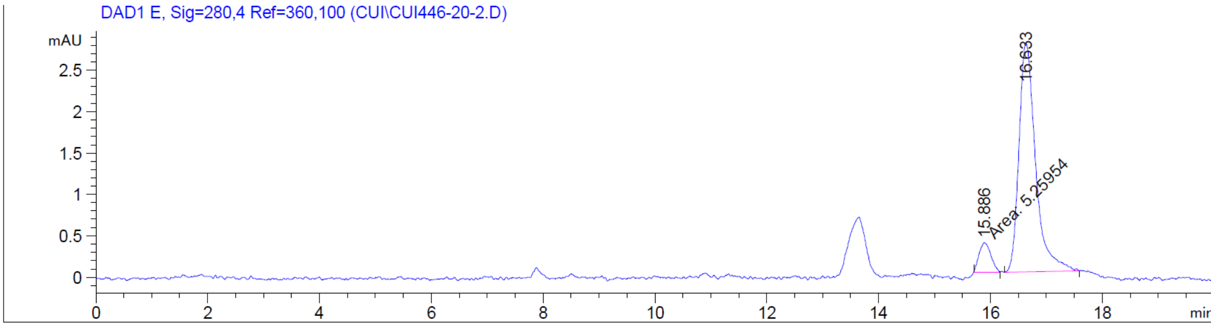

Signal 5: DAD1 E, Sig=280,4 Ref=360,100

Peak #	RetTime [min]	Type	Width [min]	Area [mAU*s]	Height [mAU]	Area %
1	15.886	MM	0.2434	5.25954	3.60158e-1	8.4248
2	16.633	BB	0.2975	57.16953	2.76177	91.5752

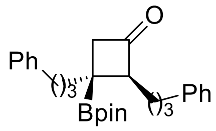

2w

(2*S*,3*R*)-2,3-Diphenethyl-3-(4,4,5,5-tetramethyl-1,3,2-dioxaborolan-2-yl)cyclobutan-1-one (2w): The enantiomeric excess of **2w** was determined by HPLC analysis on a chiral stationary phase (*Daicel* Chiralcel IC column, column temperature 20°C, solvent *n*-heptane:*i*-PrOH = 99:1, flow rate 0.5 mL/min): t_R = 42.8 min (major), t_R = 35.4 min (minor).

rac-**2w**

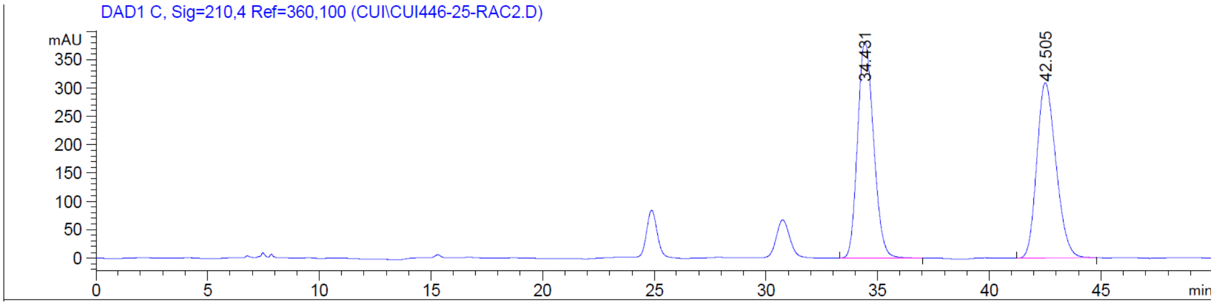

Signal 3: DAD1 C, Sig=210,4 Ref=360,100

Peak #	RetTime [min]	Type	Width [min]	Area [mAU*s]	Height [mAU]	Area %
1	34.431	BB	0.7709	1.90899e4	382.06894	50.7477
2	42.505	BB	0.9004	1.85274e4	309.47662	49.2523

(2*S*,3*R*)-**2w**

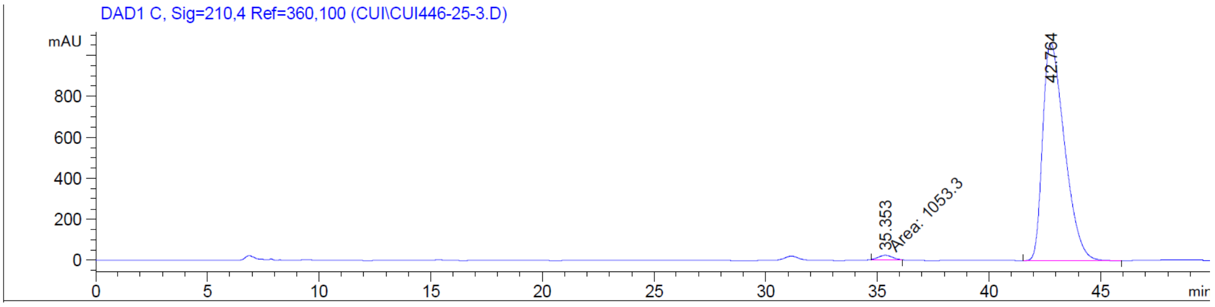

Signal 3: DAD1 C, Sig=210,4 Ref=360,100

Peak #	RetTime [min]	Type	Width [min]	Area [mAU*s]	Height [mAU]	Area %
1	35.353	MM	0.7568	1053.30029	23.19607	1.4333
2	42.764	BB	0.9759	7.24343e4	1061.78894	98.5667

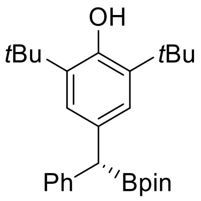

5

(*R*)-2,6-di-tert-Butyl-4-(phenyl(4,4,5,5-tetramethyl-1,3,2-dioxaborolan-2-yl)methyl)phenol (5): The enantiomeric excess of **5** was determined by HPLC analysis on a chiral stationary phase (*Daicel* Chiralcel OD-H column, column temperature 20°C, solvent *n*-heptane:*i*-PrOH = 99.8:0.2, flow rate 0.3 mL/min): t_R = 17.3 min (major), t_R = 18.7 min (minor).

rac-**5**

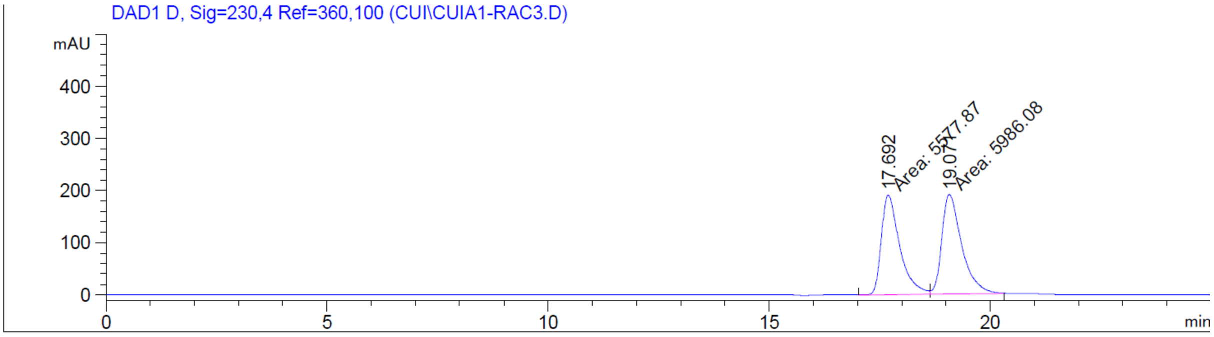

Signal 4: DAD1 D, Sig=230,4 Ref=360,100

Peak #	RetTime [min]	Type	Width [min]	Area [mAU*s]	Height [mAU]	Area %
1	17.692	MF	0.4866	5577.87158	191.04343	48.2350
2	19.071	FM	0.5216	5986.07715	191.26042	51.7650

(*R*)-**5**

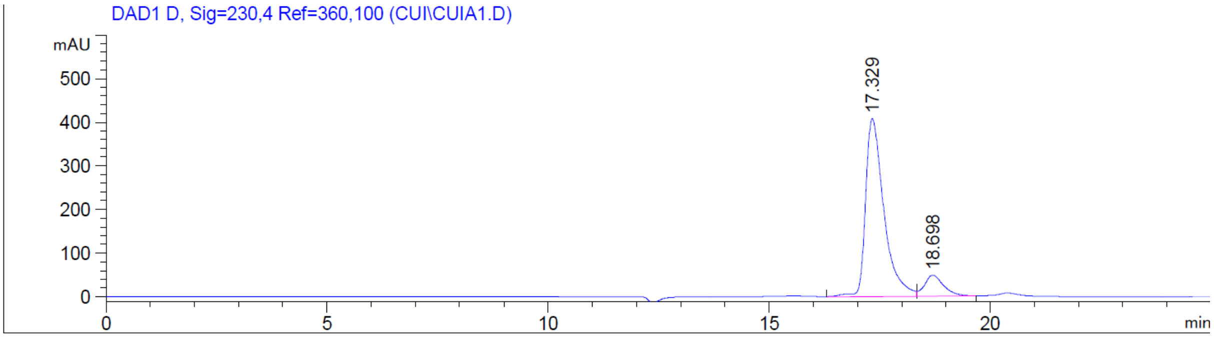

Signal 4: DAD1 D, Sig=230,4 Ref=360,100

Peak #	RetTime [min]	Type	Width [min]	Area [mAU*s]	Height [mAU]	Area %
1	17.329	BV	0.4317	1.17206e4	407.34790	89.1453
2	18.698	VB	0.4476	1427.14661	47.89550	10.8547

7 NMR Spectra

Figure S1. [1]**H NMR** spectrum (400 MHz, $CDCl_3$, 298 K) of cyclobutenone **1f**.

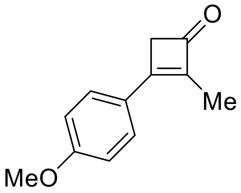

1f

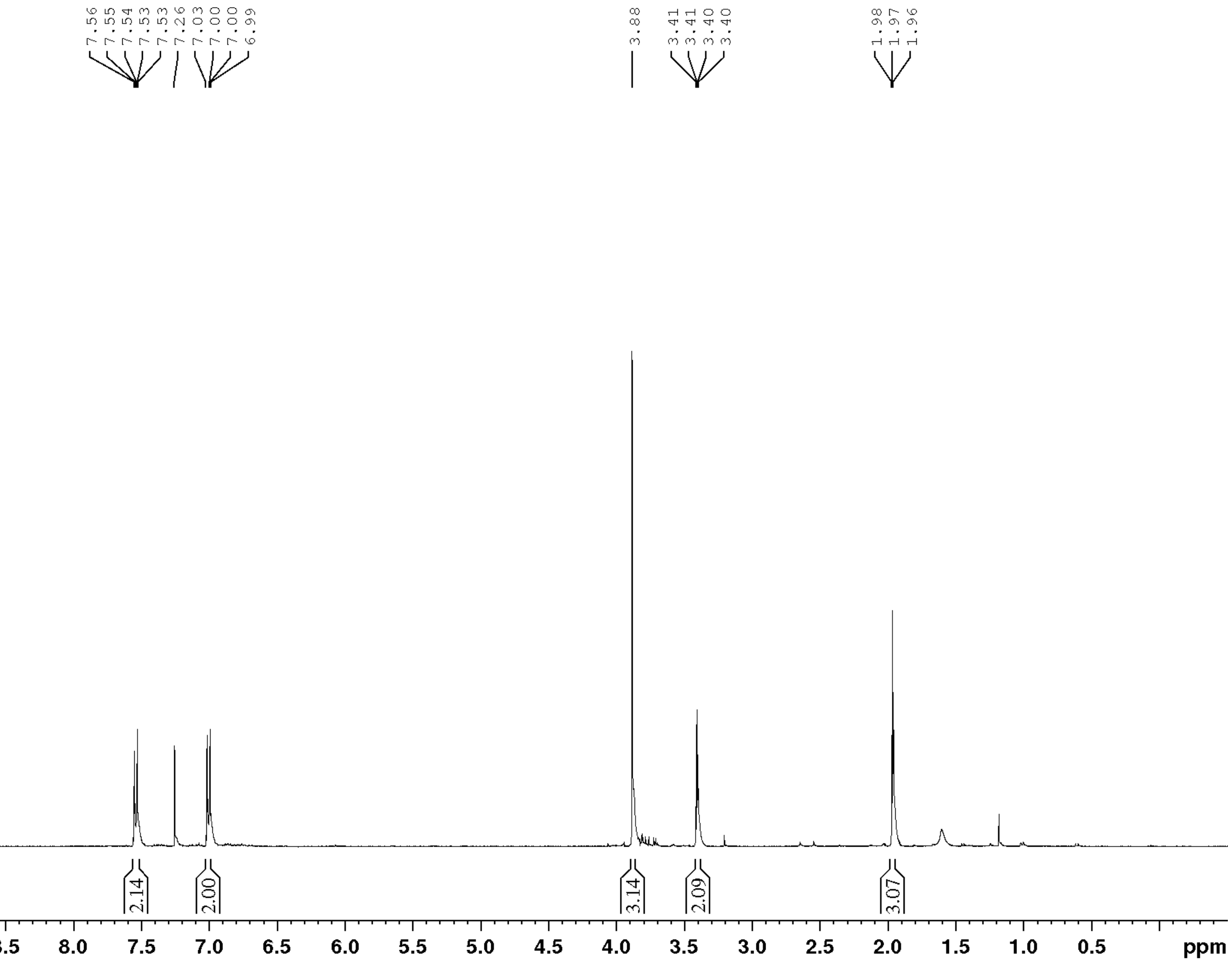

Figure S2. **$^{13}C\{^{1}H\}$ NMR** spectrum (101 MHz, $CDCl_3$, 298 K) of cyclobutenone **1f**.

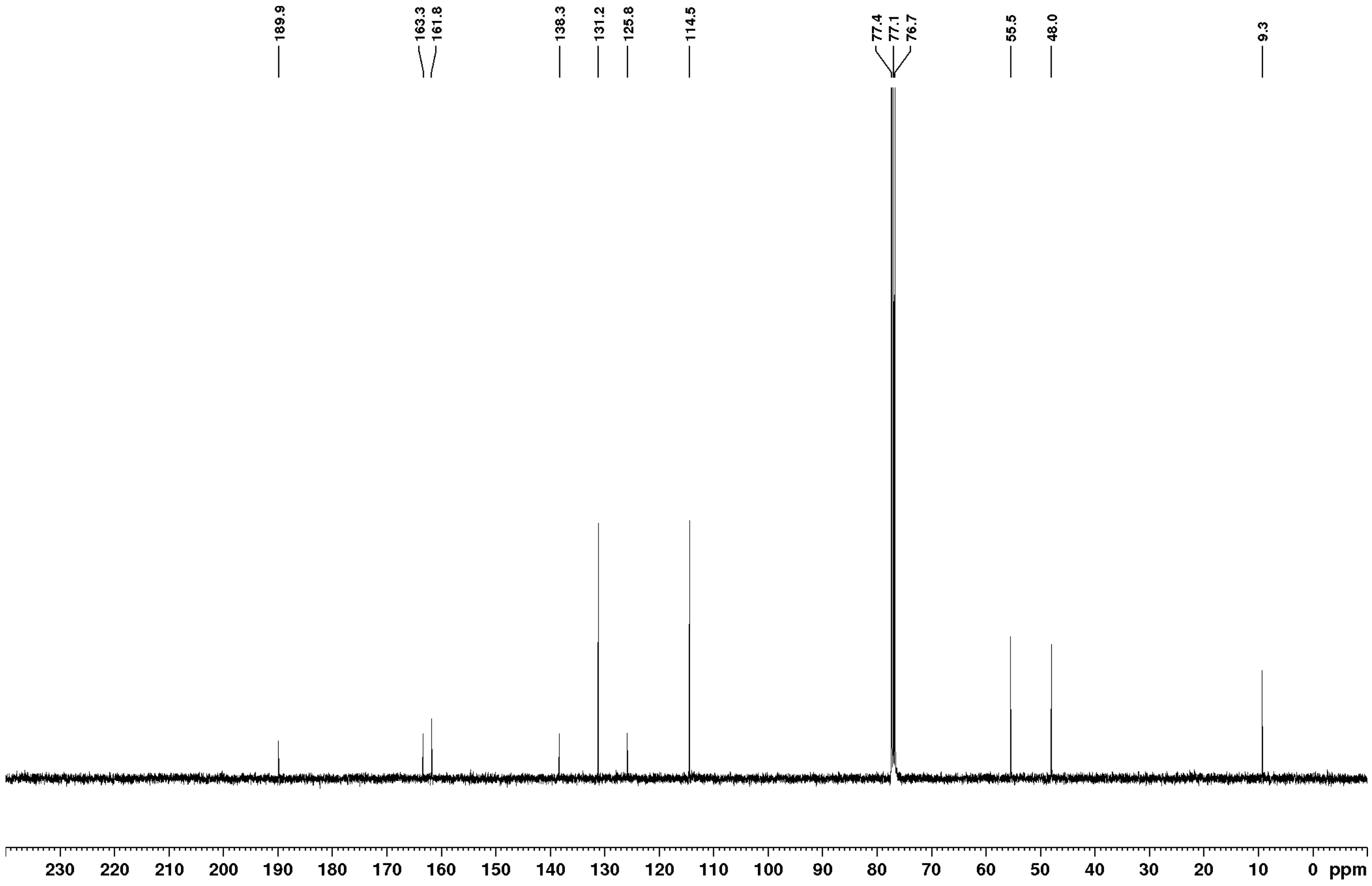

Figure S3. **^{1}H NMR** spectrum (500 MHz, $CDCl_3$, 298 K) of cyclobutenone **1h**.

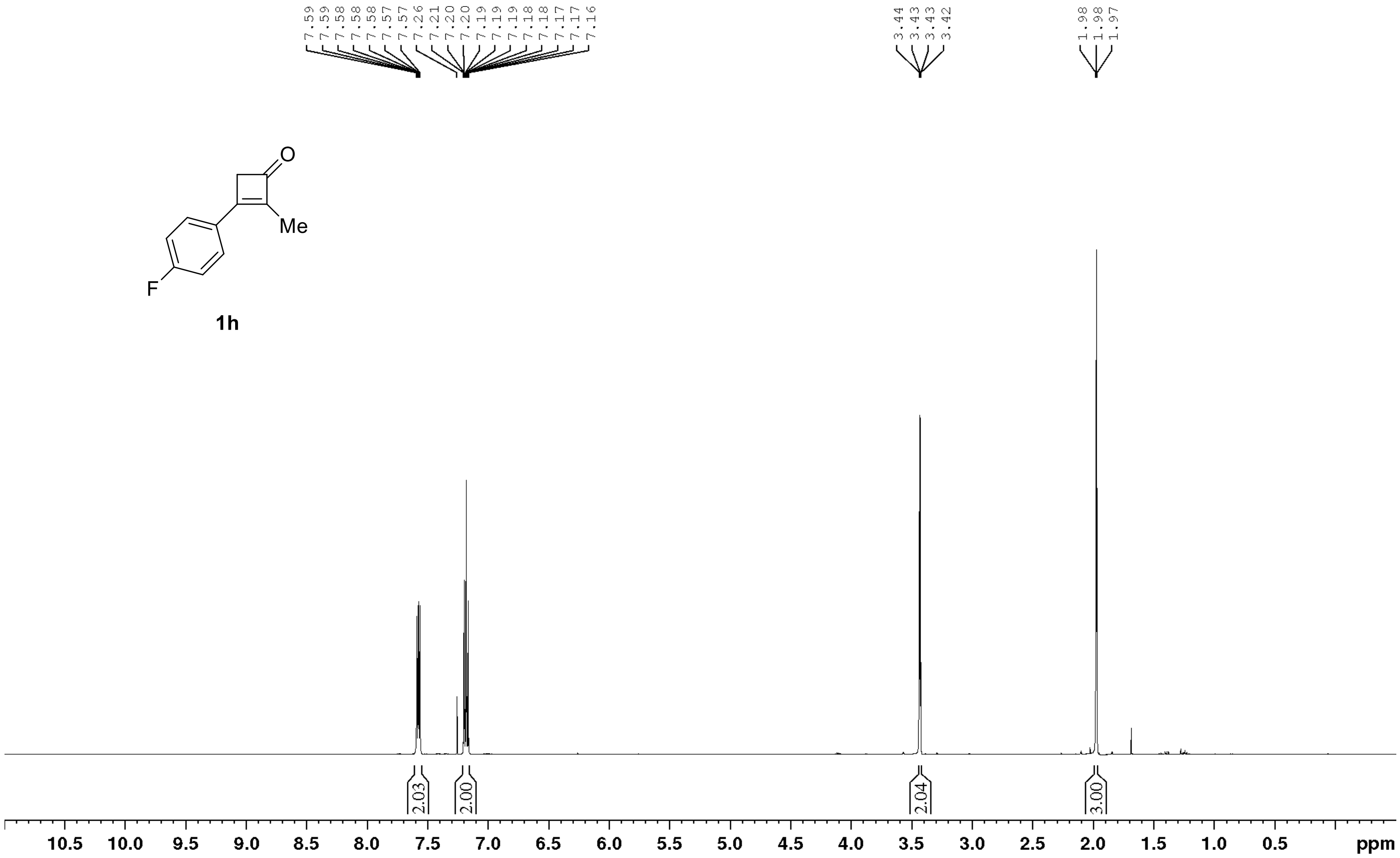

Figure S4. **$^{13}C\{^1H\}$ NMR** spectrum (126 MHz, $CDCl_3$, 298 K) of cyclobutenone **1h**.

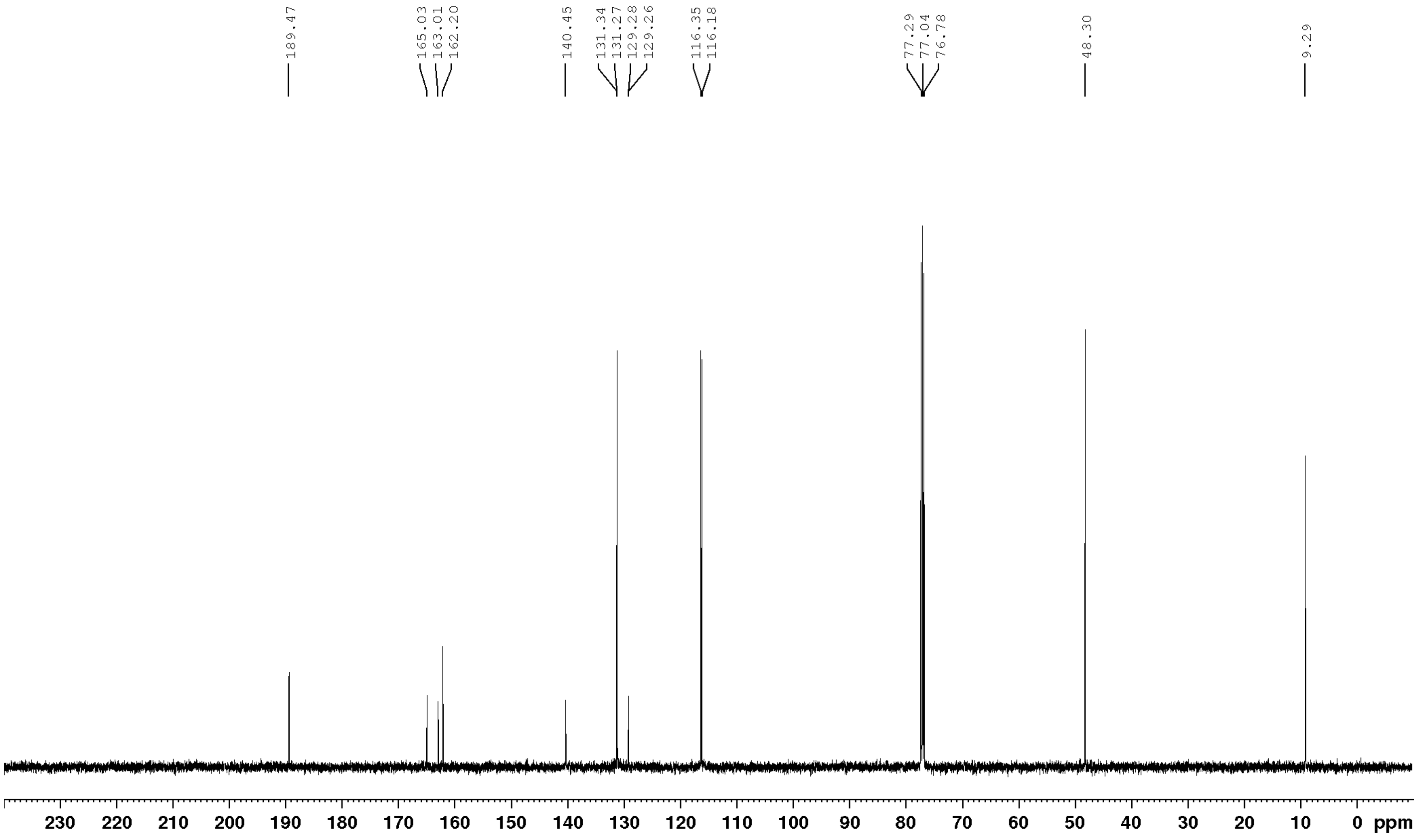

Figure S5. **^{19}F NMR** spectrum (471 MHz, $CDCl_3$, 298 K) of cyclobutenone **1h**.

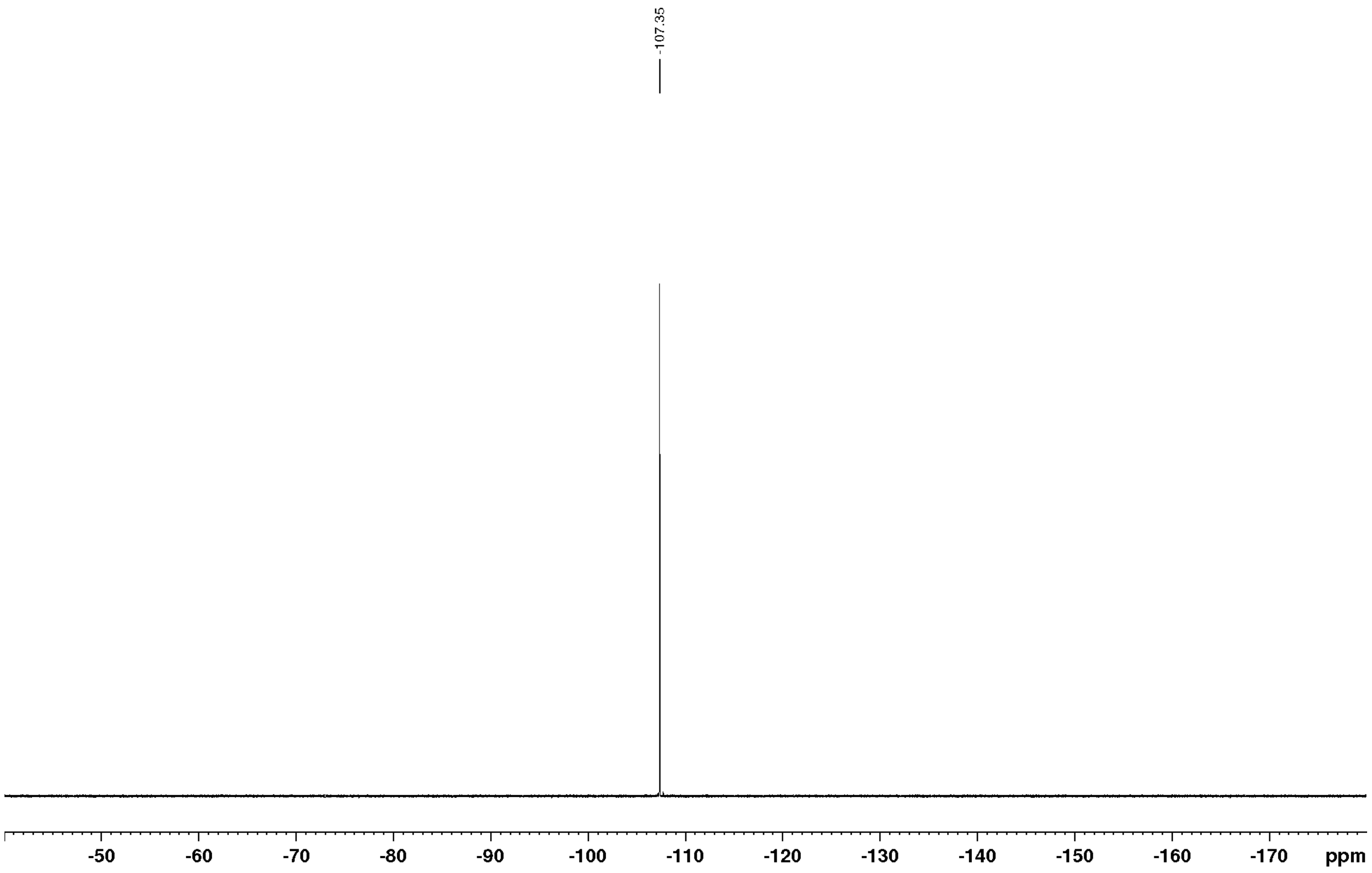

Figure S6. **^{1}H NMR** spectrum (500 MHz, $CDCl_3$, 298 K) of cyclobutenone **1m**.

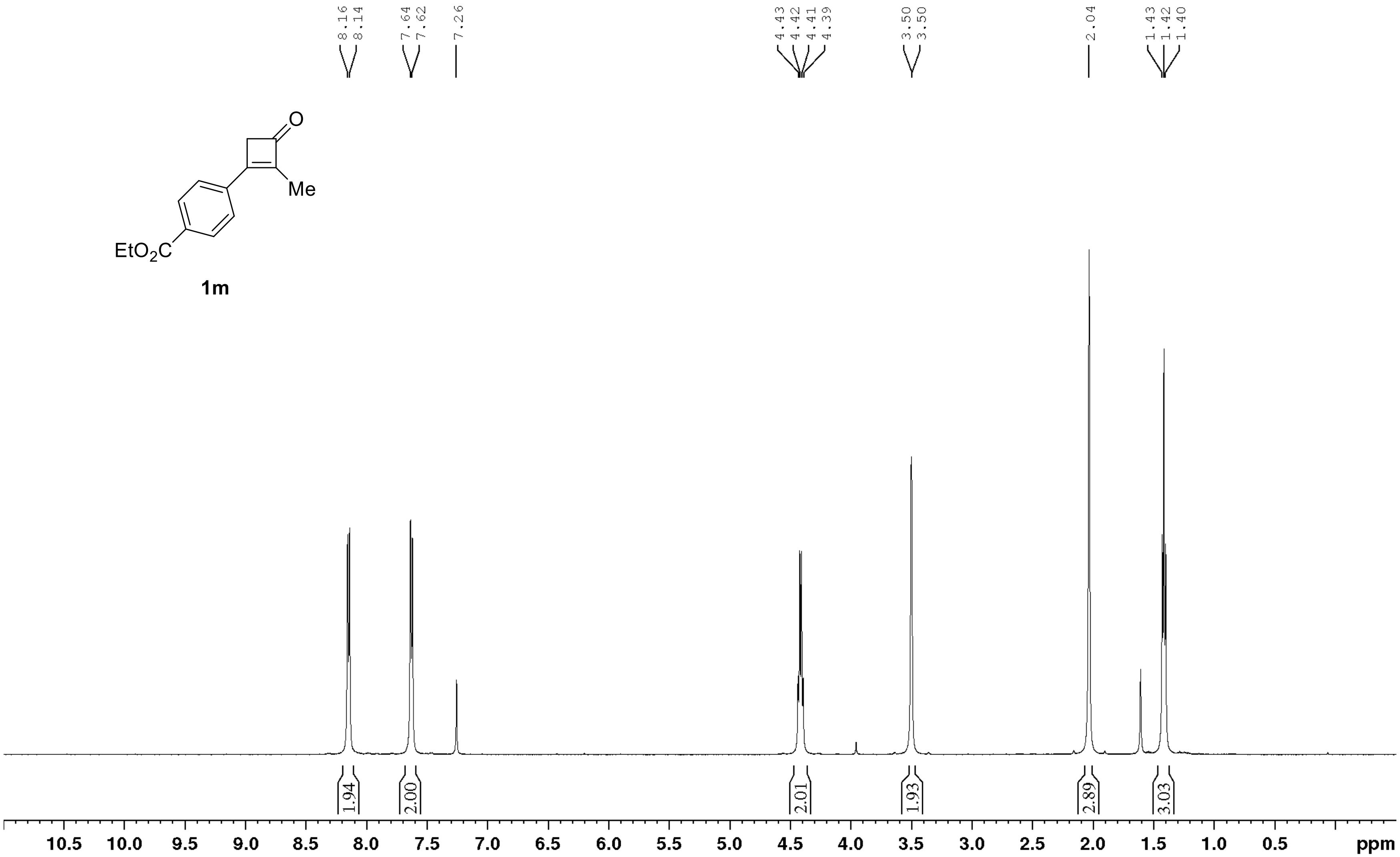

Figure S7. **$^{13}C\{^1H\}$ NMR** spectrum (126 MHz, $CDCl_3$, 298 K) of cyclobutenone **1m**.

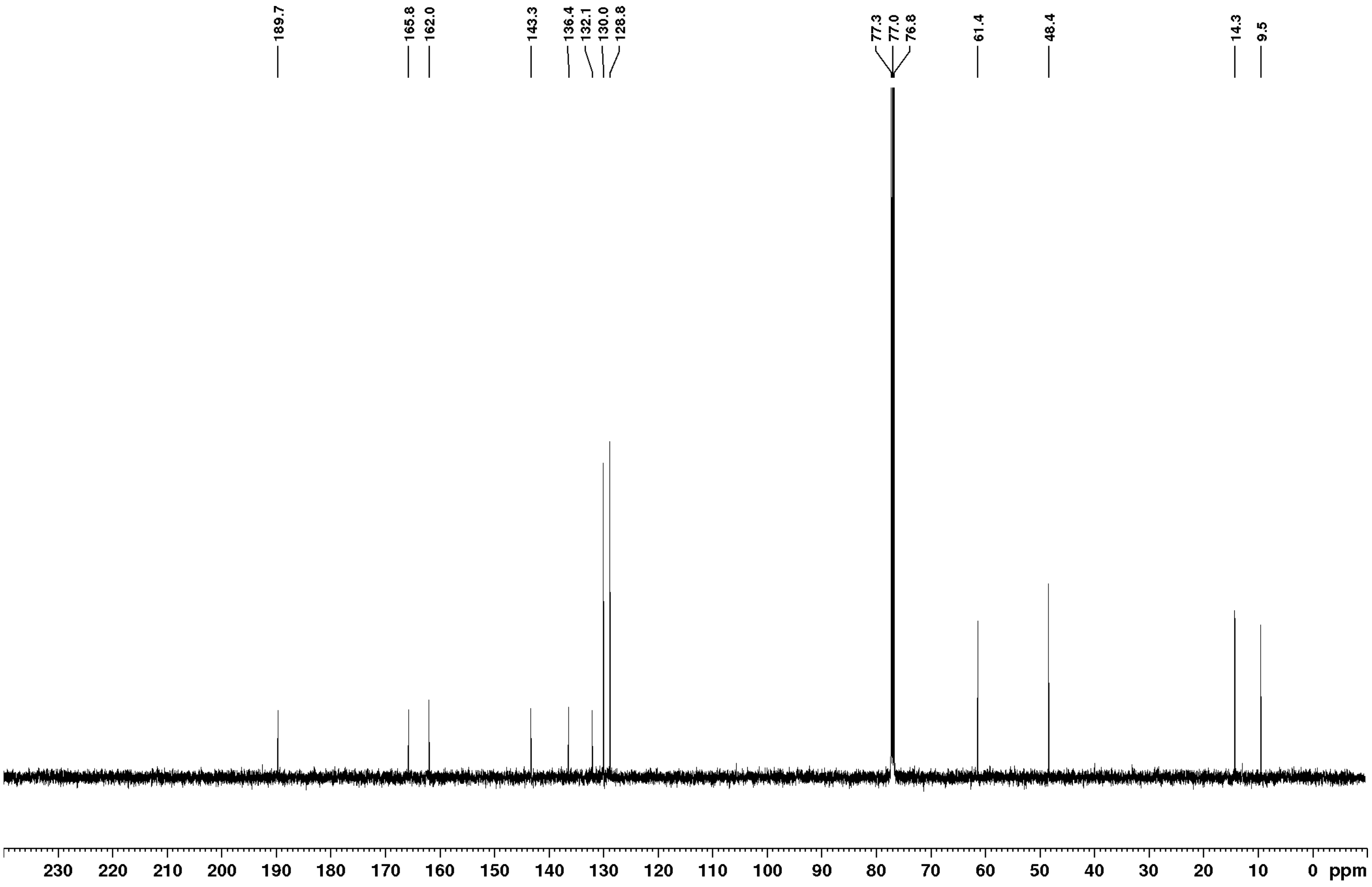

Figure S8. **^{1}H NMR** spectrum (500 MHz, $CDCl_3$, 298 K) of cyclobutenone **1q**.

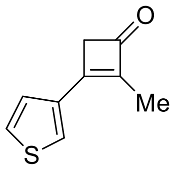

1q

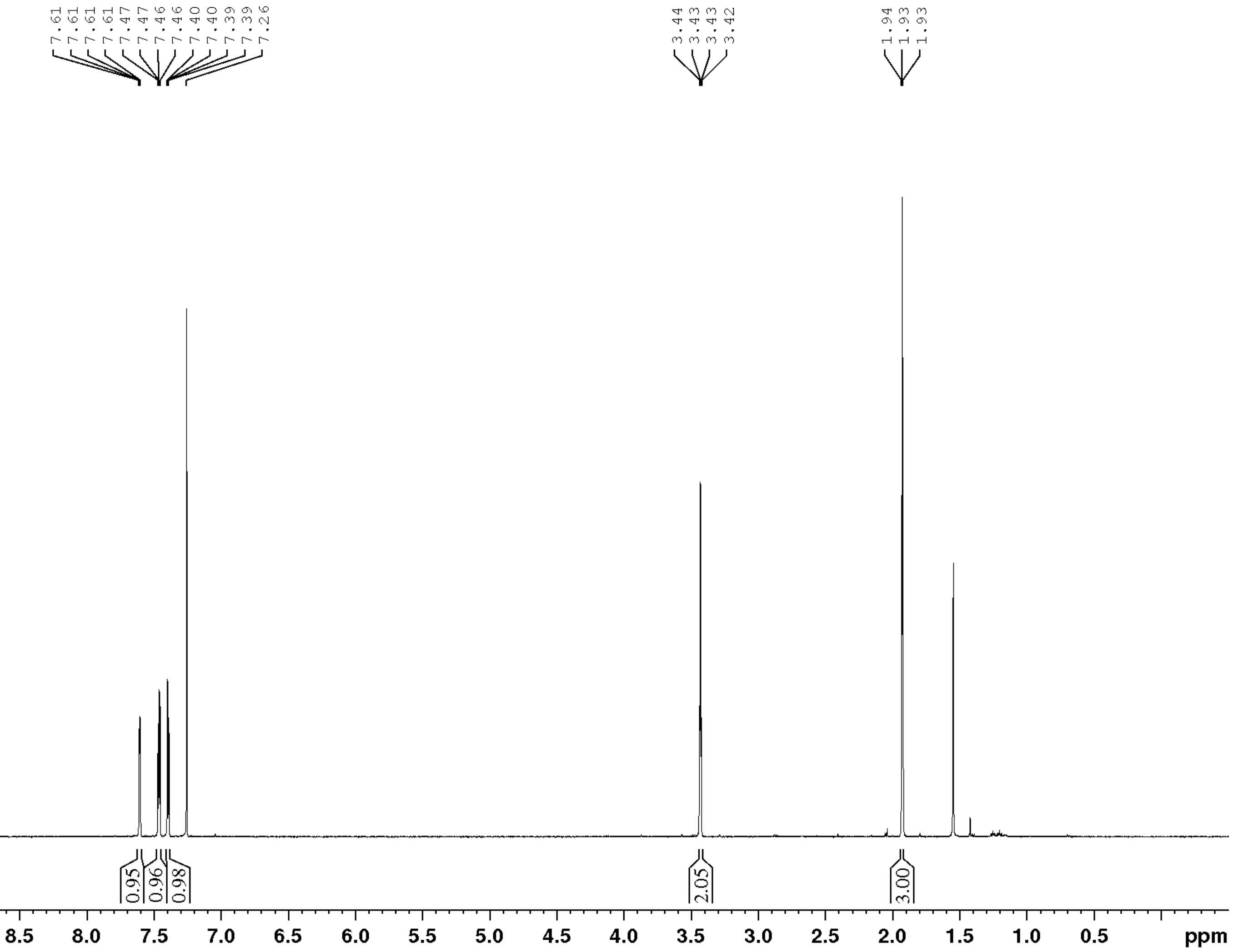

Figure S9. **$^{13}C\{^1H\}$ NMR** spectrum (126 MHz, $CDCl_3$, 298 K) of cyclobutenone **1q**.

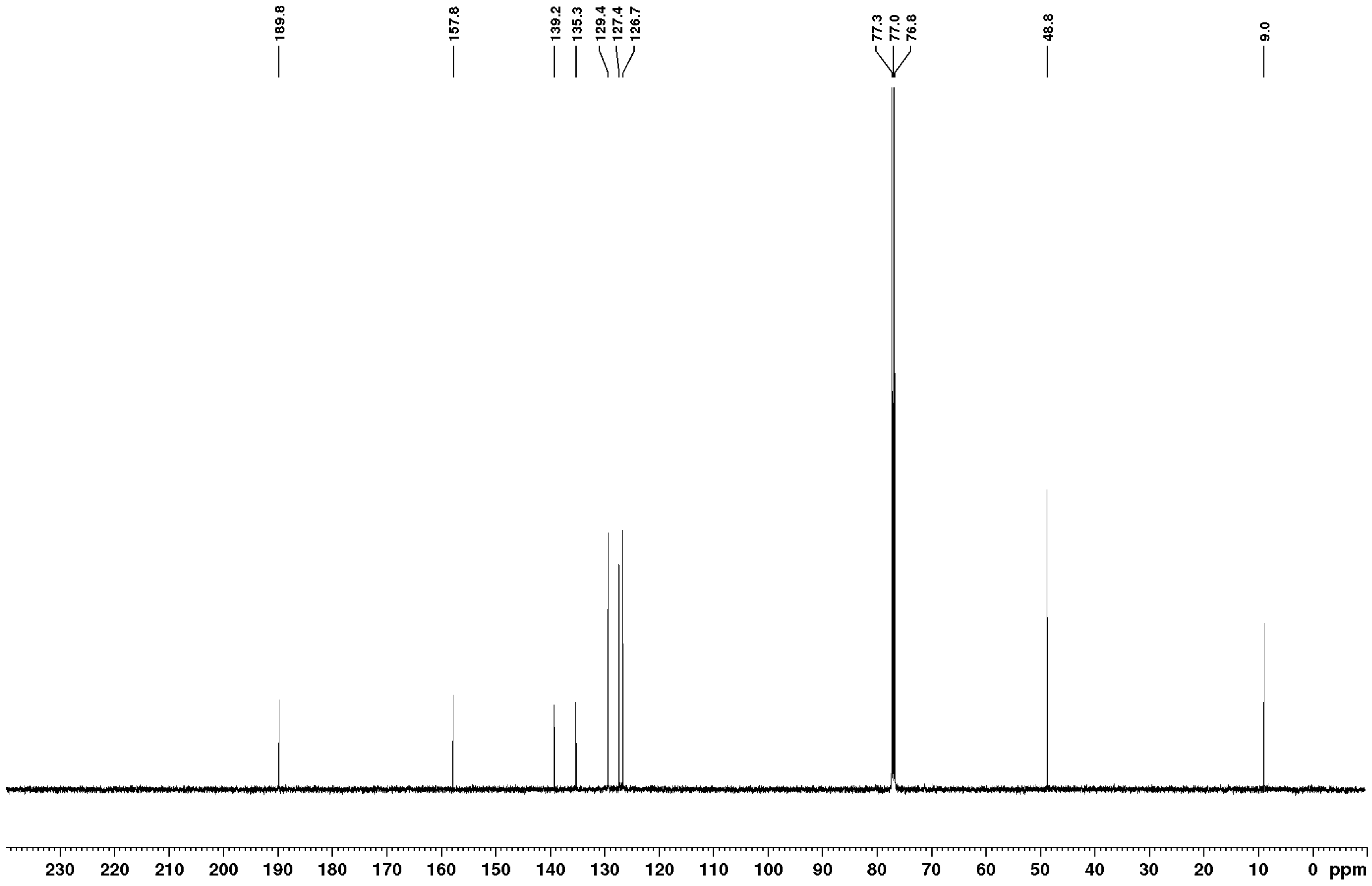

Figure S10. **^{1}H NMR** spectrum (500 MHz, $CDCl_3$, 298 K) of cyclobutenone **1r**.

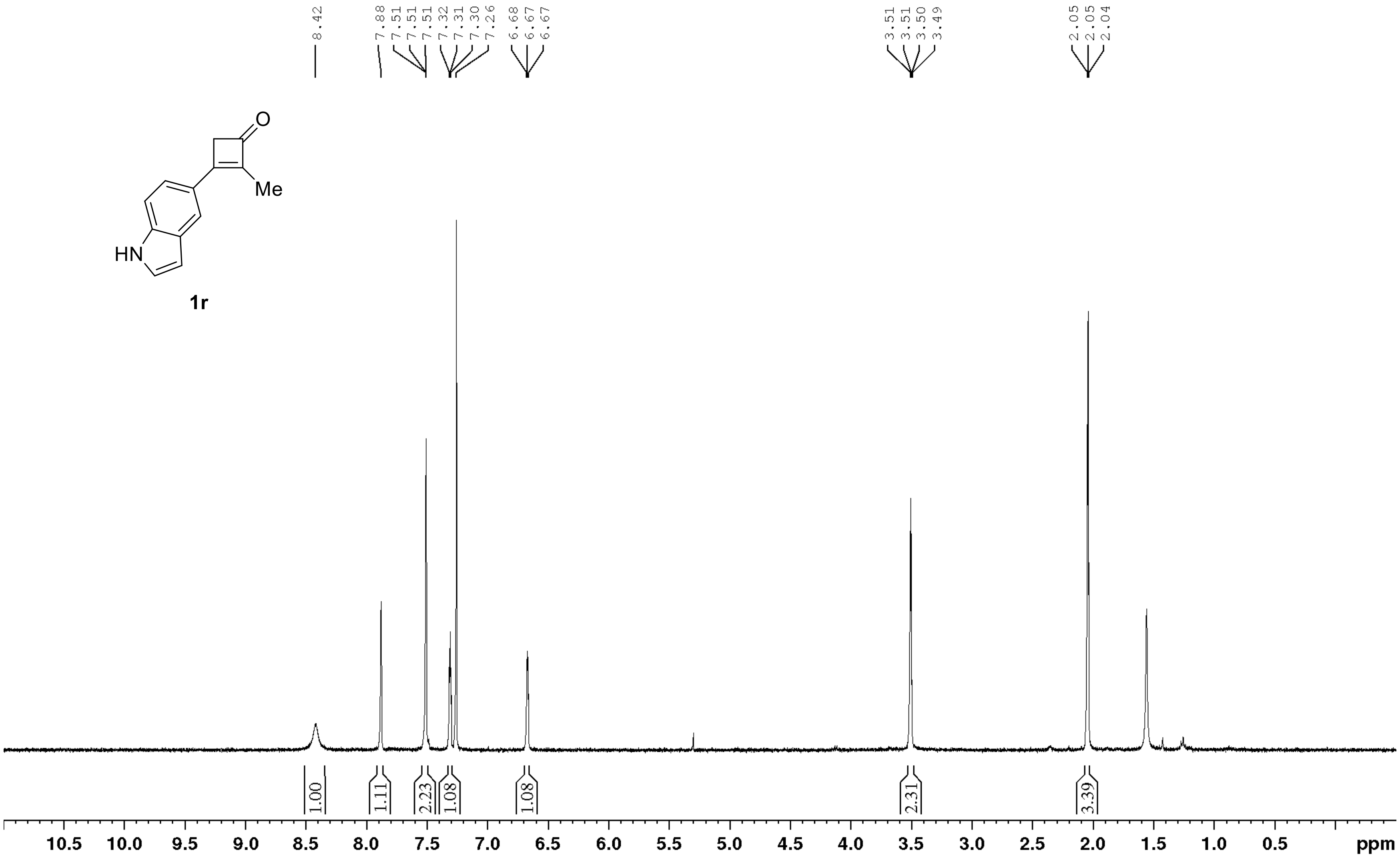

Figure S11. $^{13}C\{^{1}H\}$ **NMR** spectrum (101 MHz, $CDCl_3$, 298 K) of cyclobutenone **1r**.

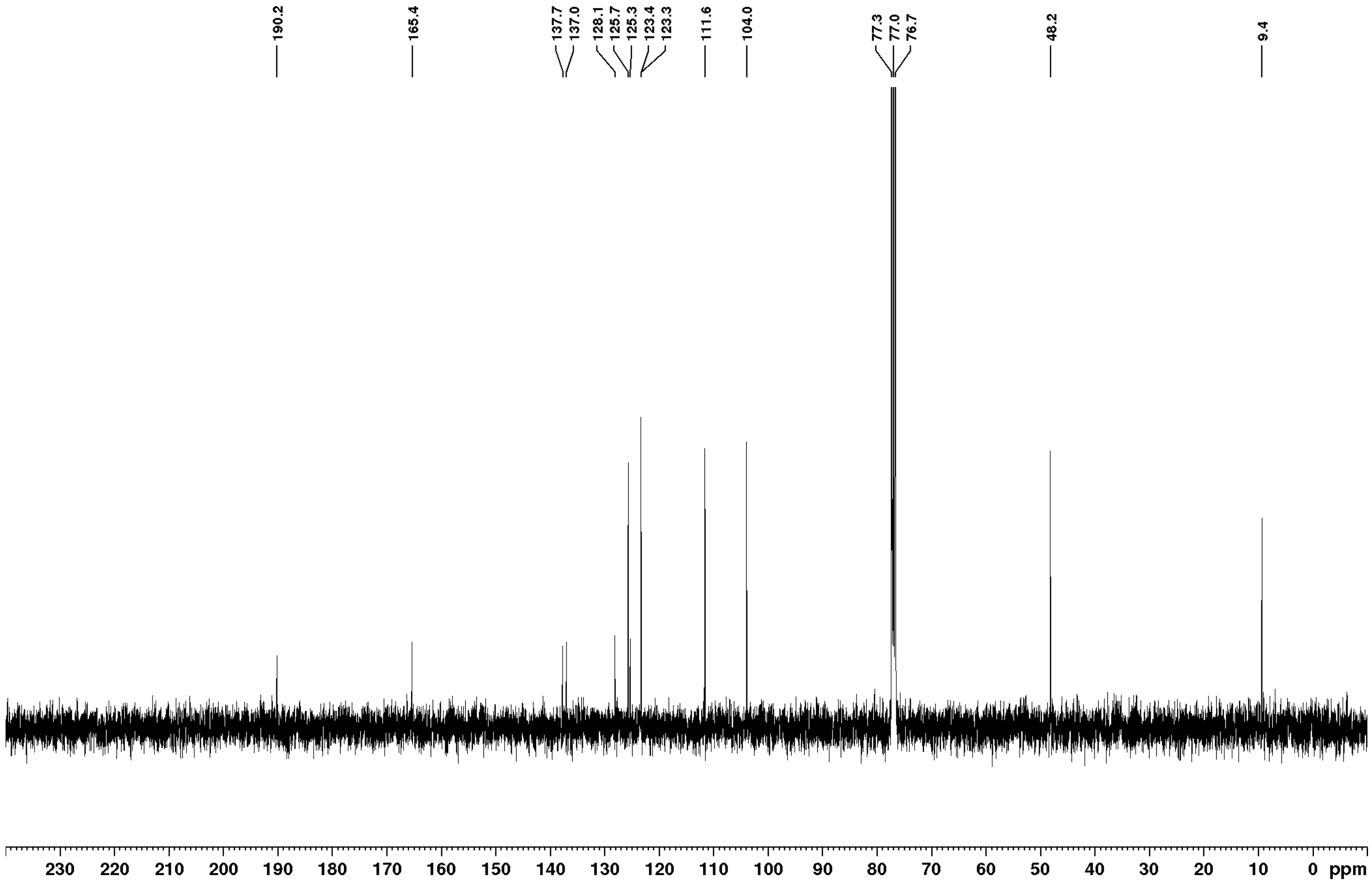

Figure S12. **^{1}H NMR** spectrum (400 MHz, $CDCl_3$, 298 K) of cyclobutylboronate **2a**.

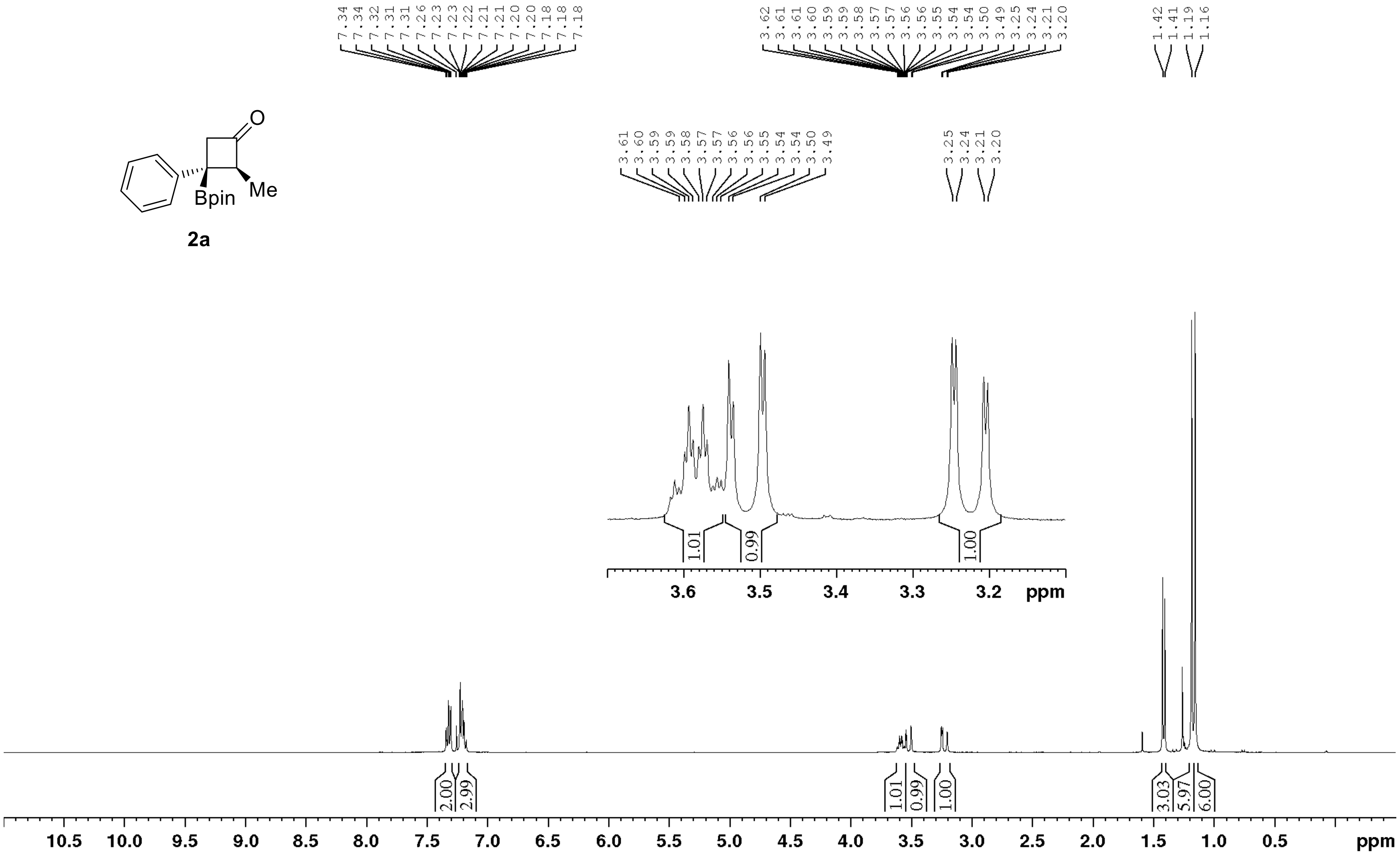

Figure S13. **$^{13}C\{^1H\}$ NMR** spectrum (126 MHz, $CDCl_3$, 298 K) of cyclobutylboronate **2a**.

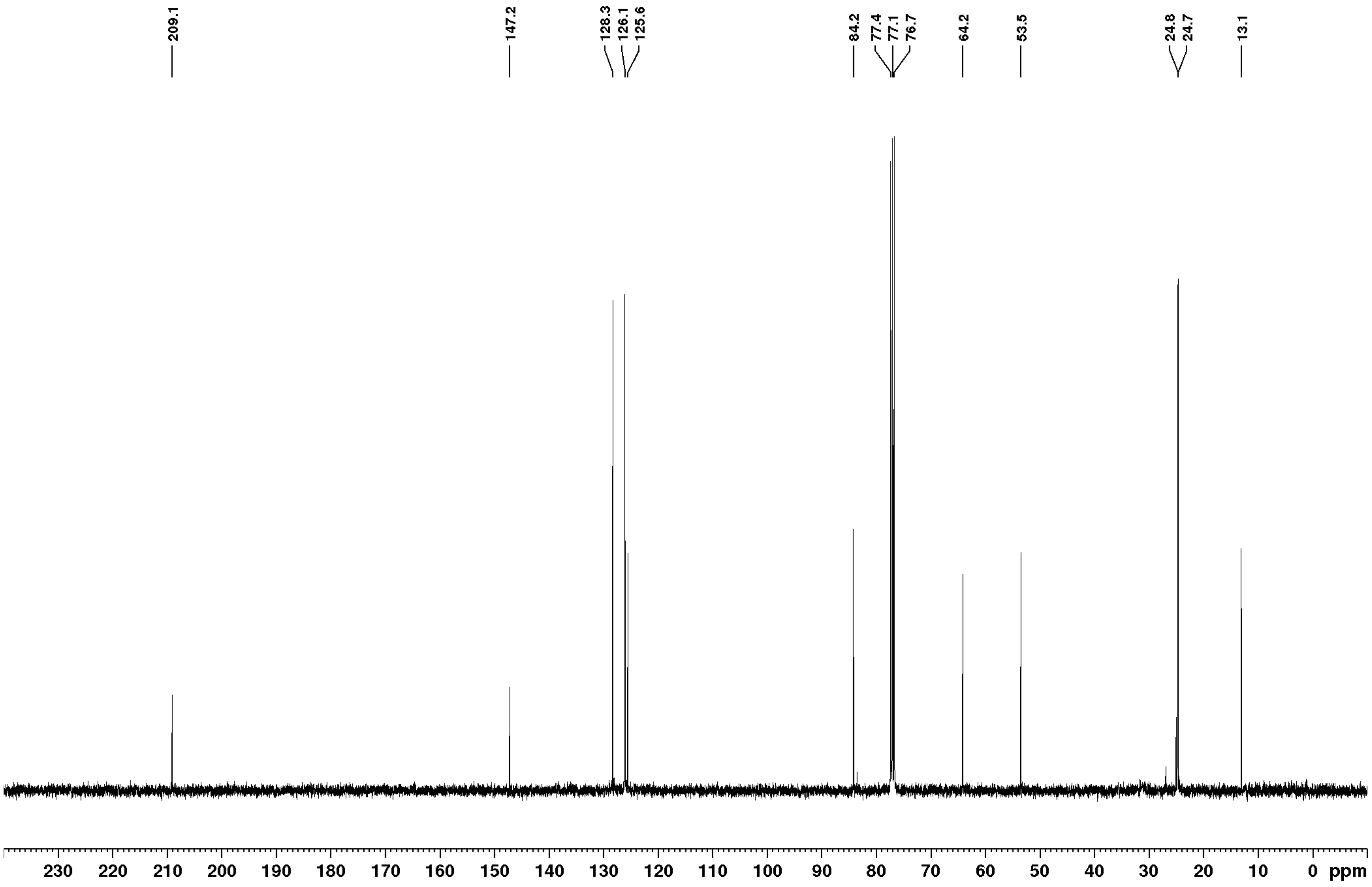

Figure S14. **^{1}H NMR** spectrum (400 MHz, $CDCl_3$, 298 K) of cyclobutylboronate **2b**.

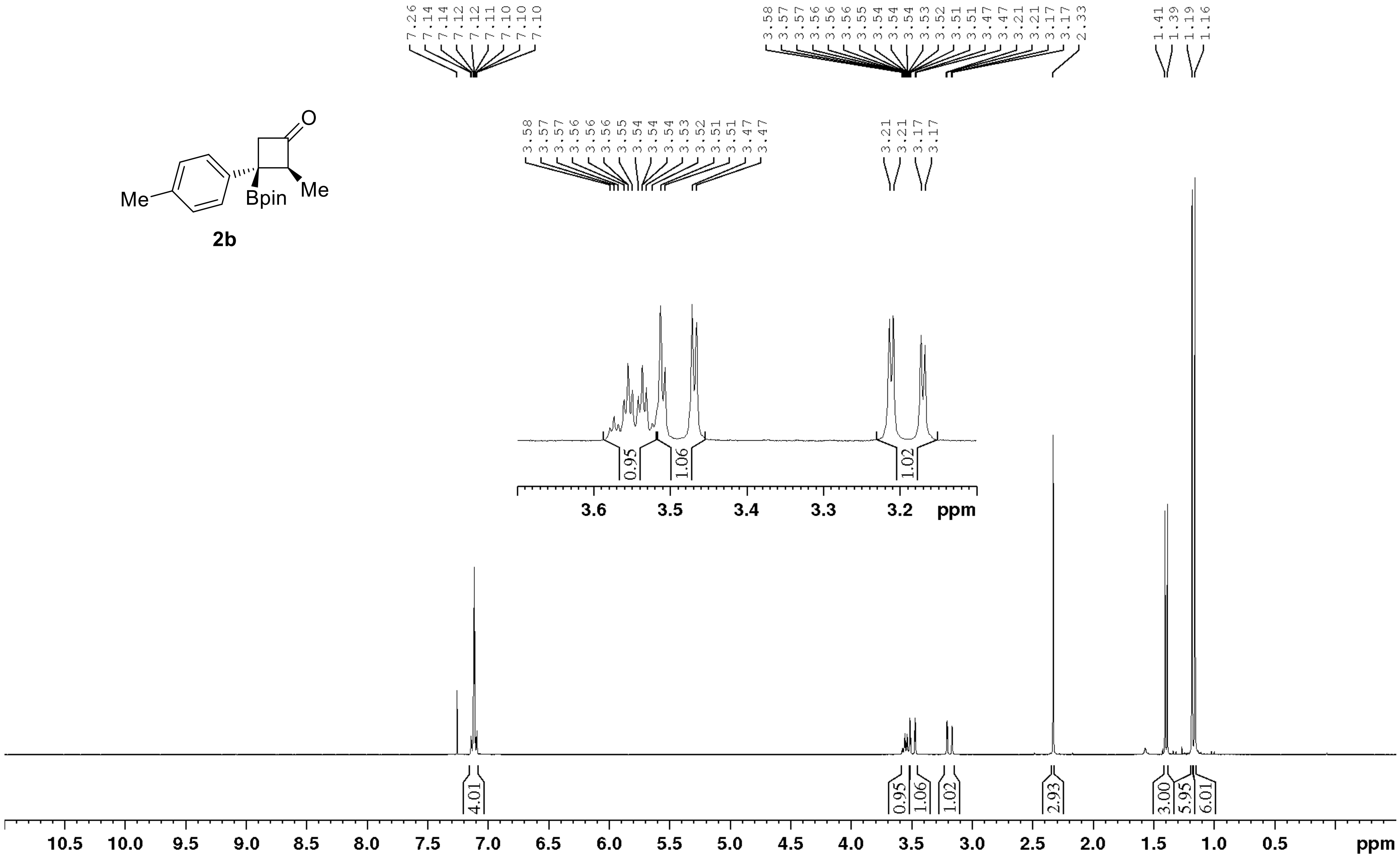

2b

Figure S15. $^{13}C\{^{1}H\}$ **NMR** spectrum (101 MHz, $CDCl_3$, 298 K) of cyclobutylboronate **2b**.

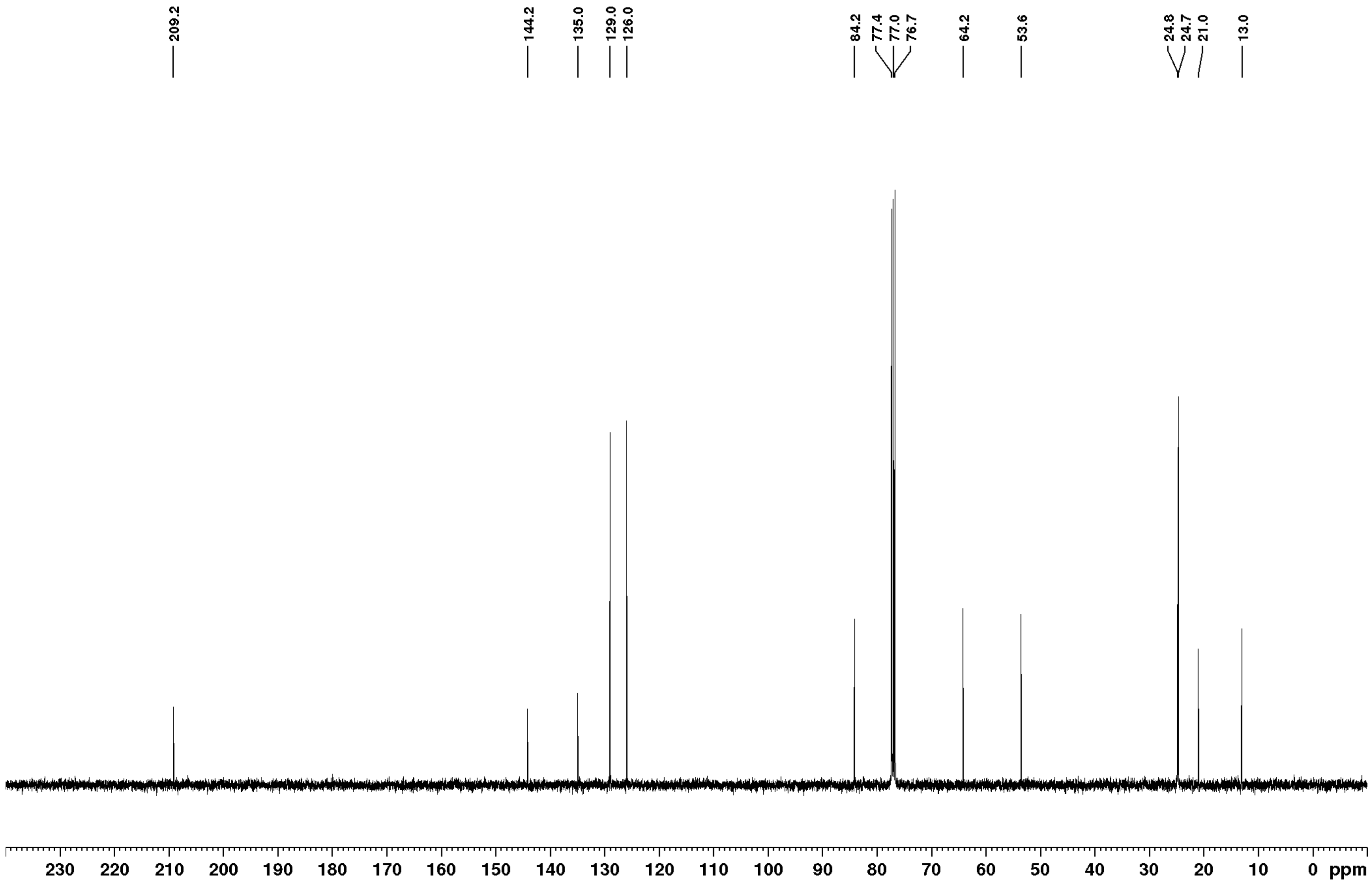

Figure S16. **^{1}H NMR** NMR spectrum (400 MHz, $CDCl_3$, 298 K) of cyclobutylboronate **2c**.

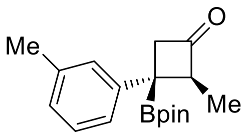

2c

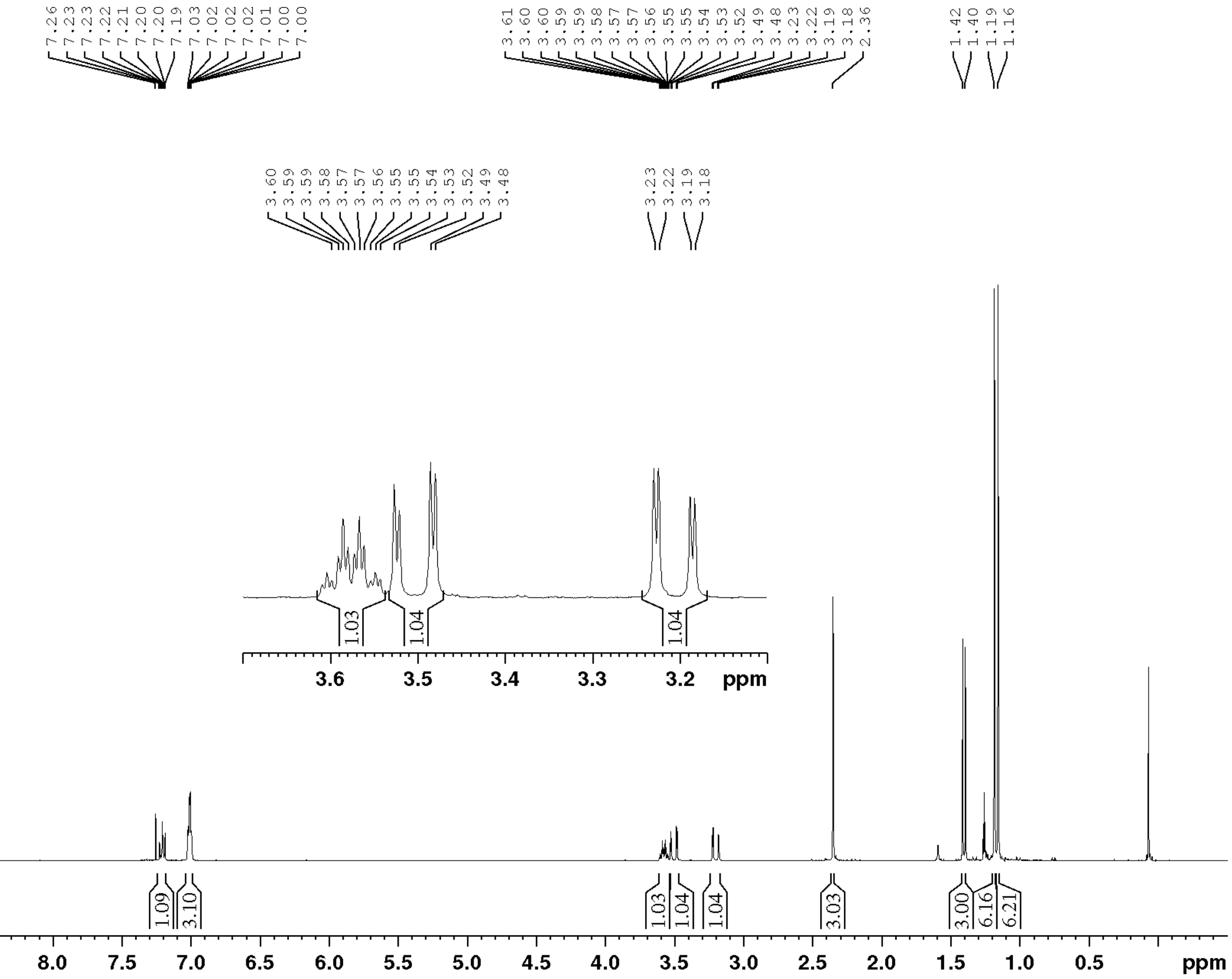

Figure S17. **$^{13}C\{^1H\}$ NMR** spectrum (101 MHz, $CDCl_3$, 298 K) of cyclobutylboronate **2c**.

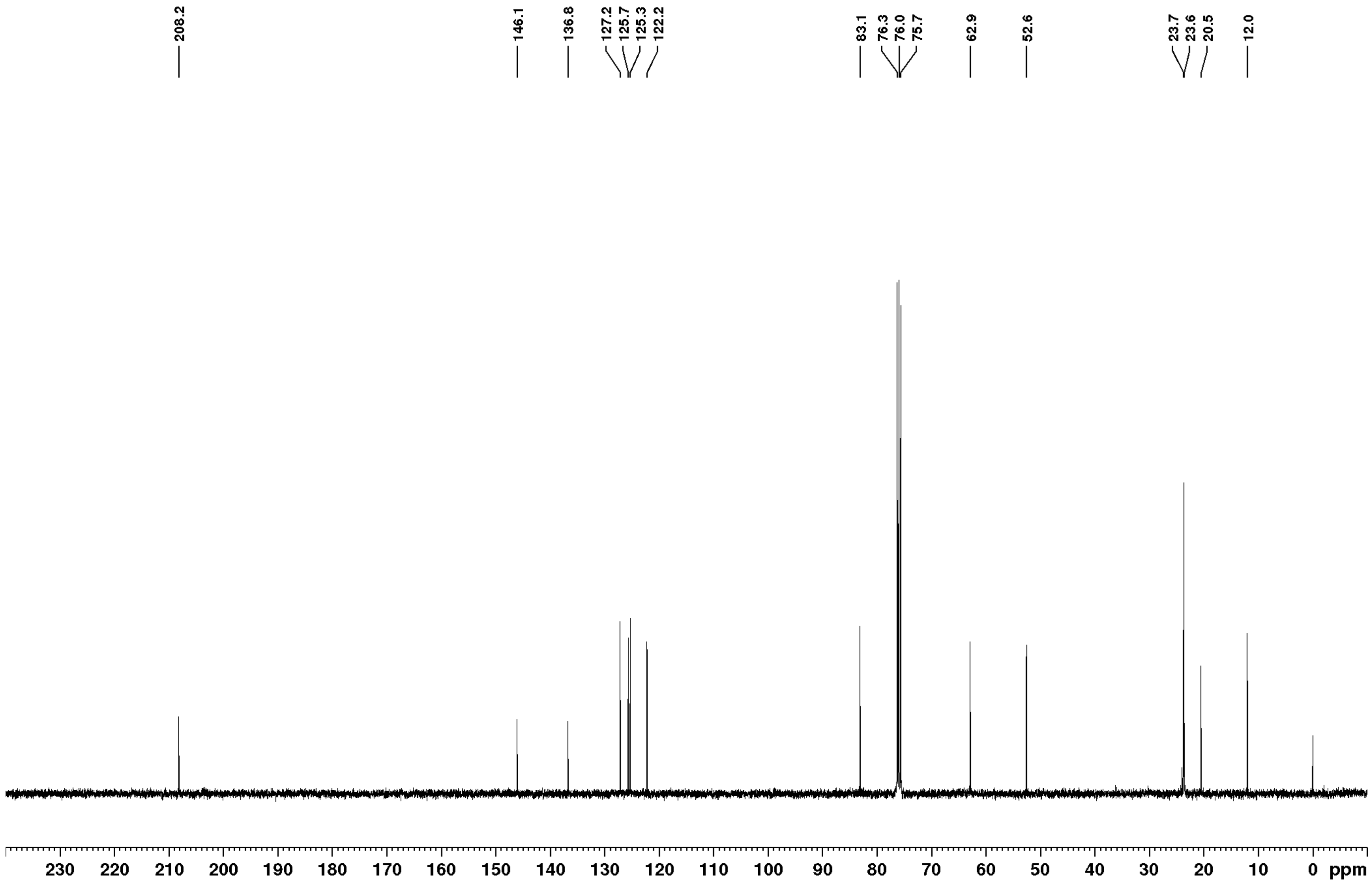

Figure S18. **^{1}H NMR** spectrum (500 MHz, $CDCl_3$, 298 K) of cyclobutylboronate **2d**.

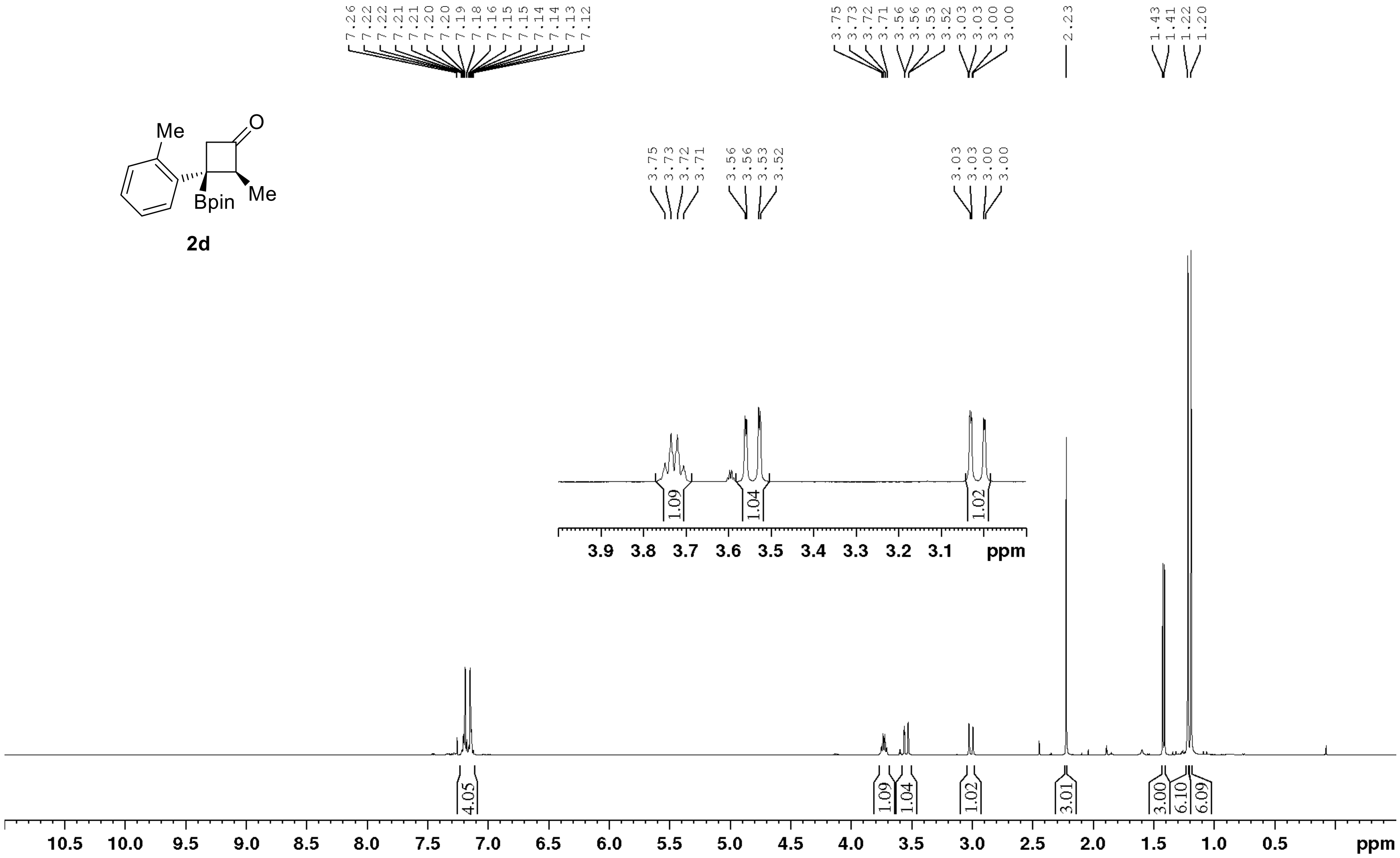

Figure S19. **$^{13}C\{^{1}H\}$ NMR** spectrum (126 MHz, $CDCl_3$, 298 K) of cyclobutylboronate **2d**.

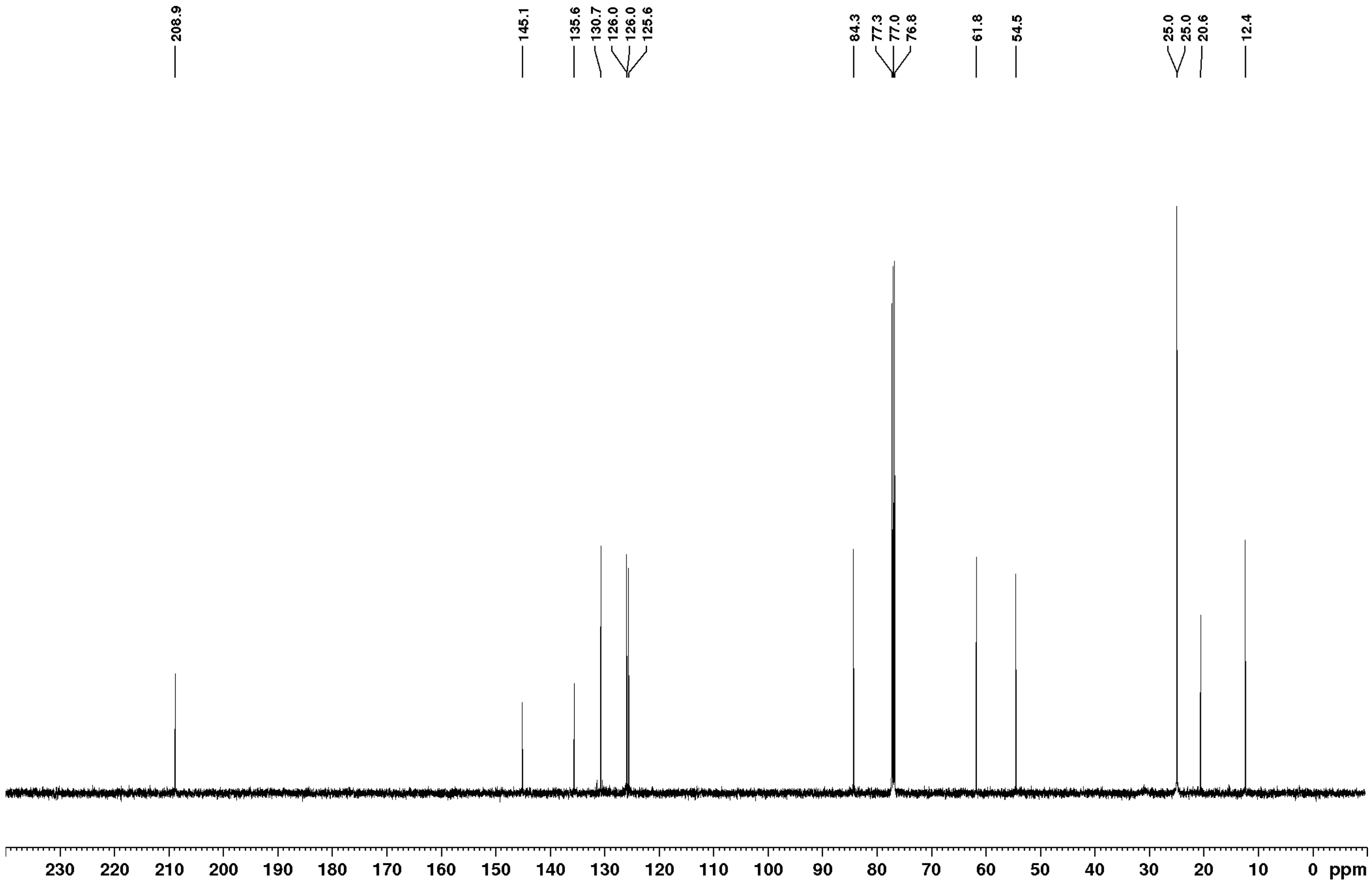

Figure S20. **^{1}H NMR** spectrum (500 MHz, $CDCl_3$, 298 K) of cyclobutylboronate **2e**.

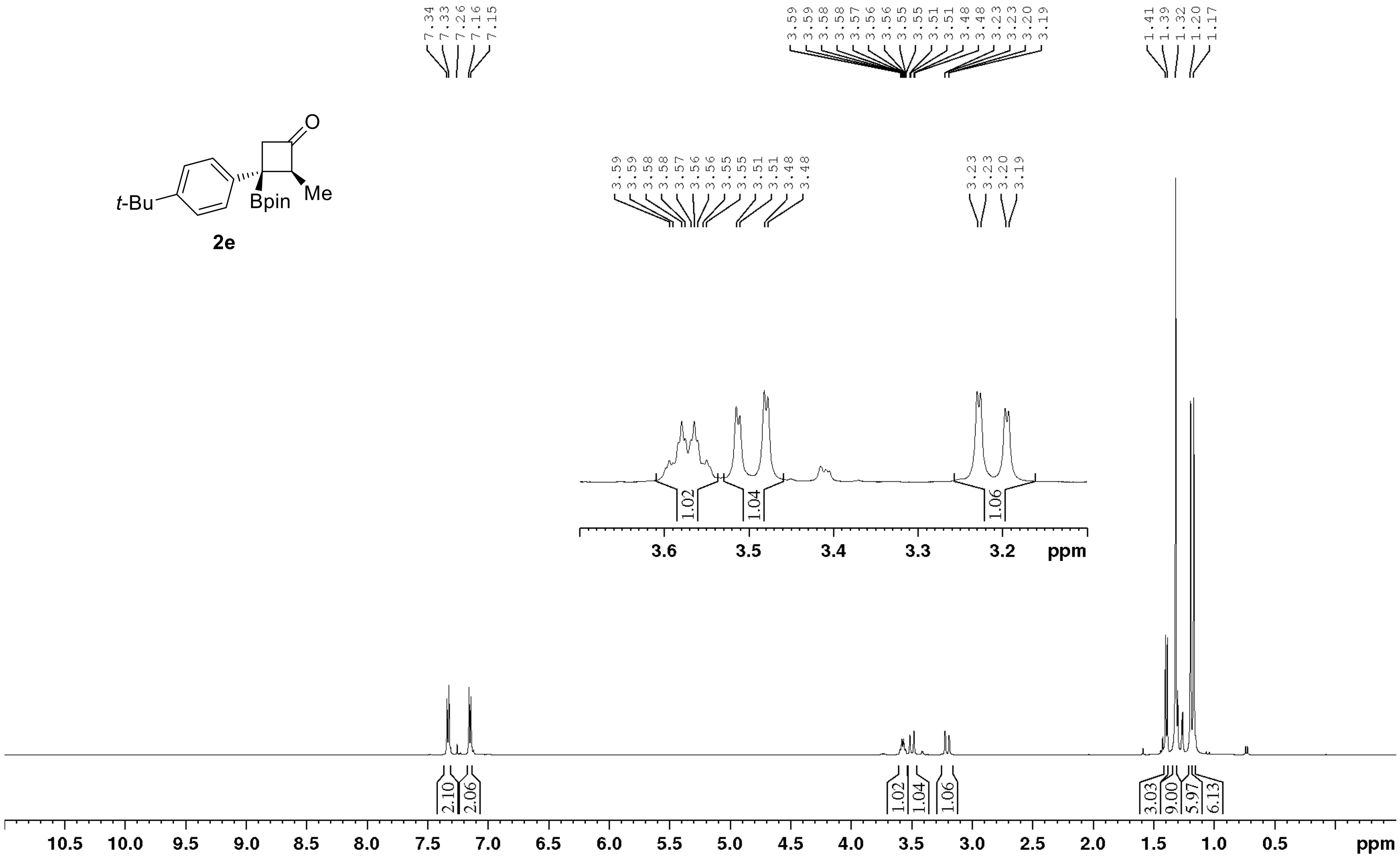

Figure S21. **$^{13}C\{^1H\}$ NMR** spectrum (126 MHz, $CDCl_3$, 298 K) of cyclobutylboronate **2e**.

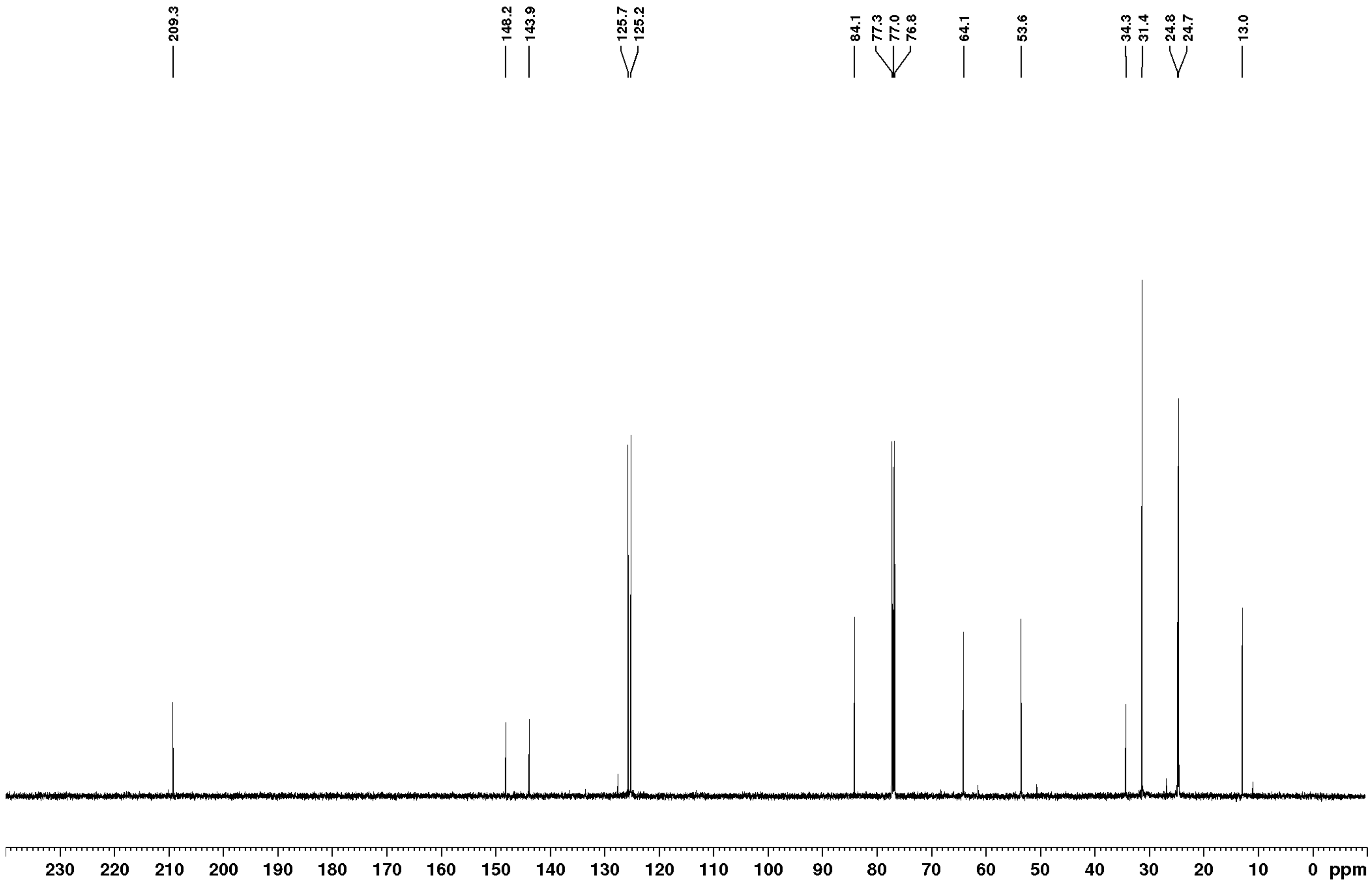

Figure S22. **^{1}H NMR** spectrum (500 MHz, $CDCl_3$, 298 K) of cyclobutylboronate **2f**.

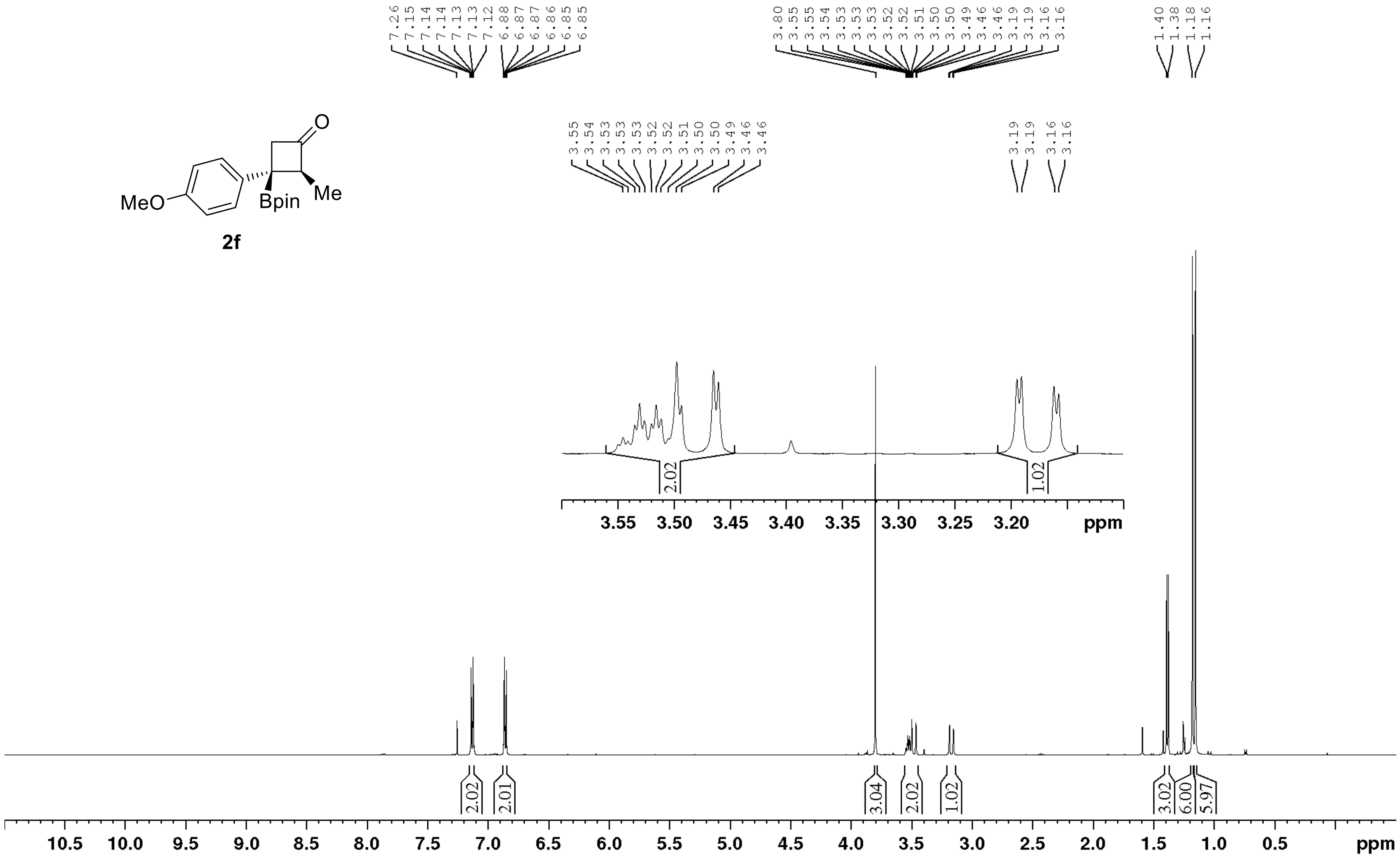

Figure S23. **$^{13}C\{^1H\}$ NMR** spectrum (126 MHz, $CDCl_3$, 298 K) of cyclobutylboronate **2f**.

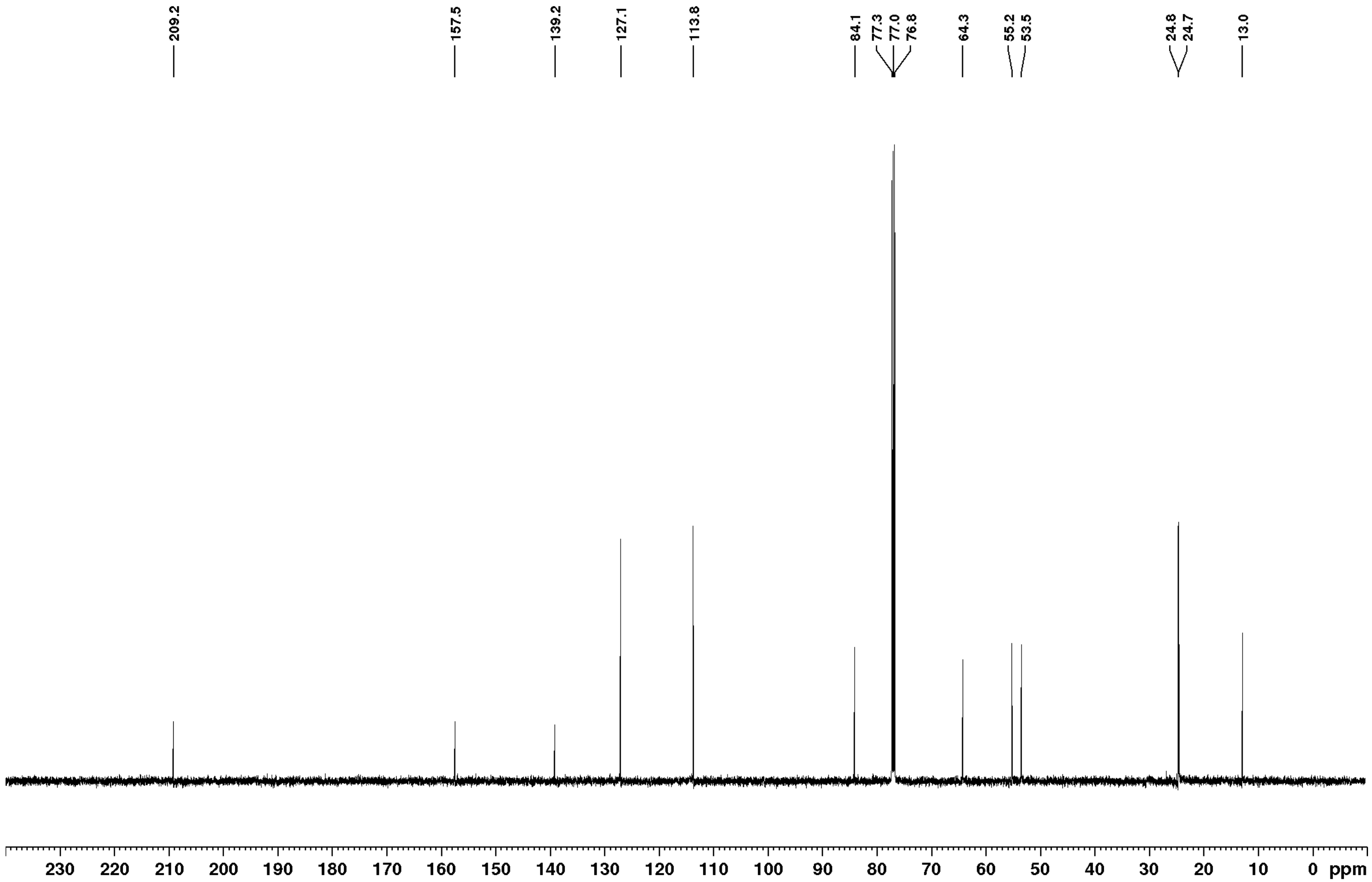

Figure S24. **^{1}H NMR** spectrum (500 MHz, $CDCl_3$, 298 K) of cyclobutylboronate **2g**.

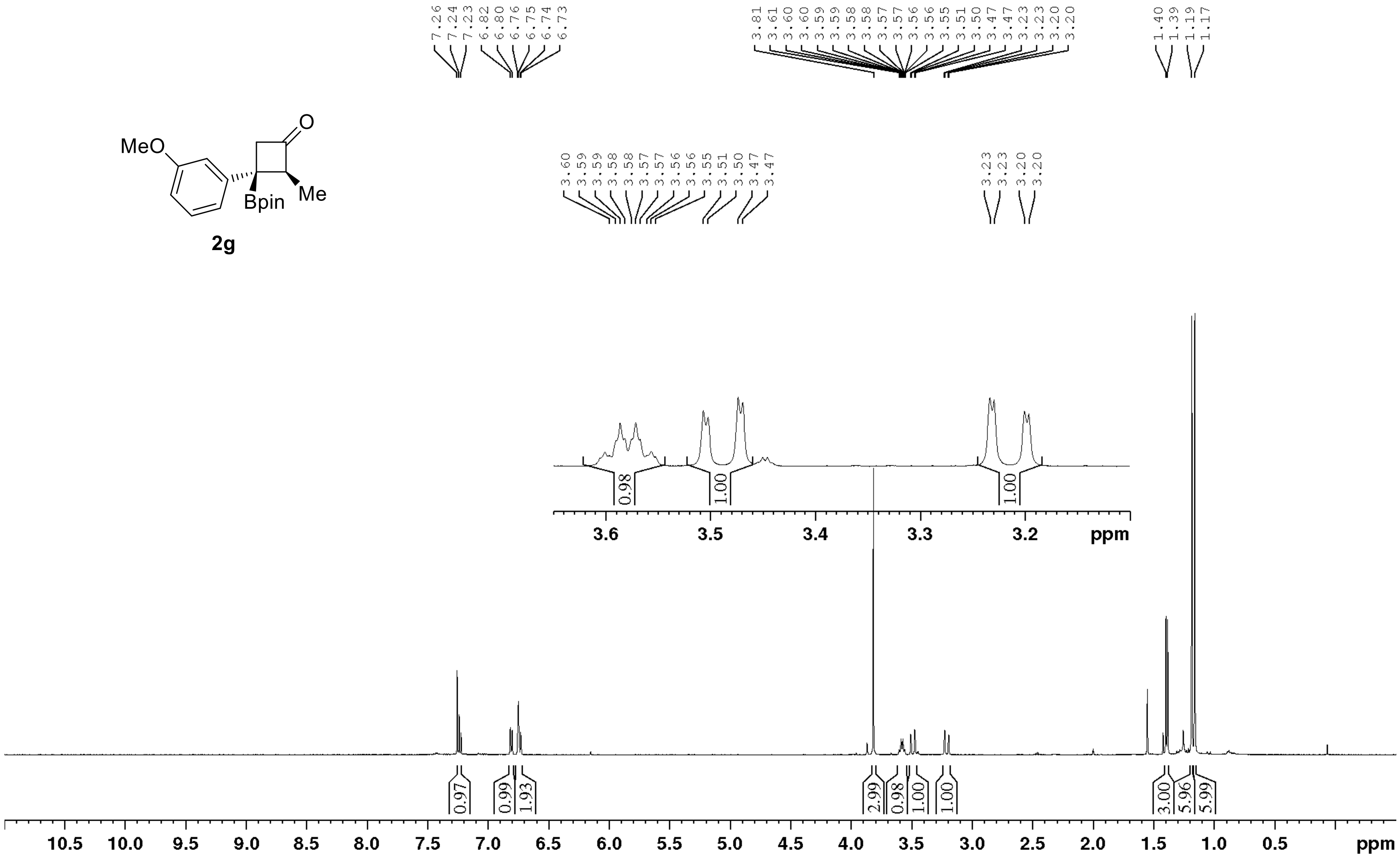

Figure S25. ^{13}**C{^{1}H}** NMR spectrum (126 MHz, $CDCl_3$, 298 K) of cyclobutylboronate **2g**.

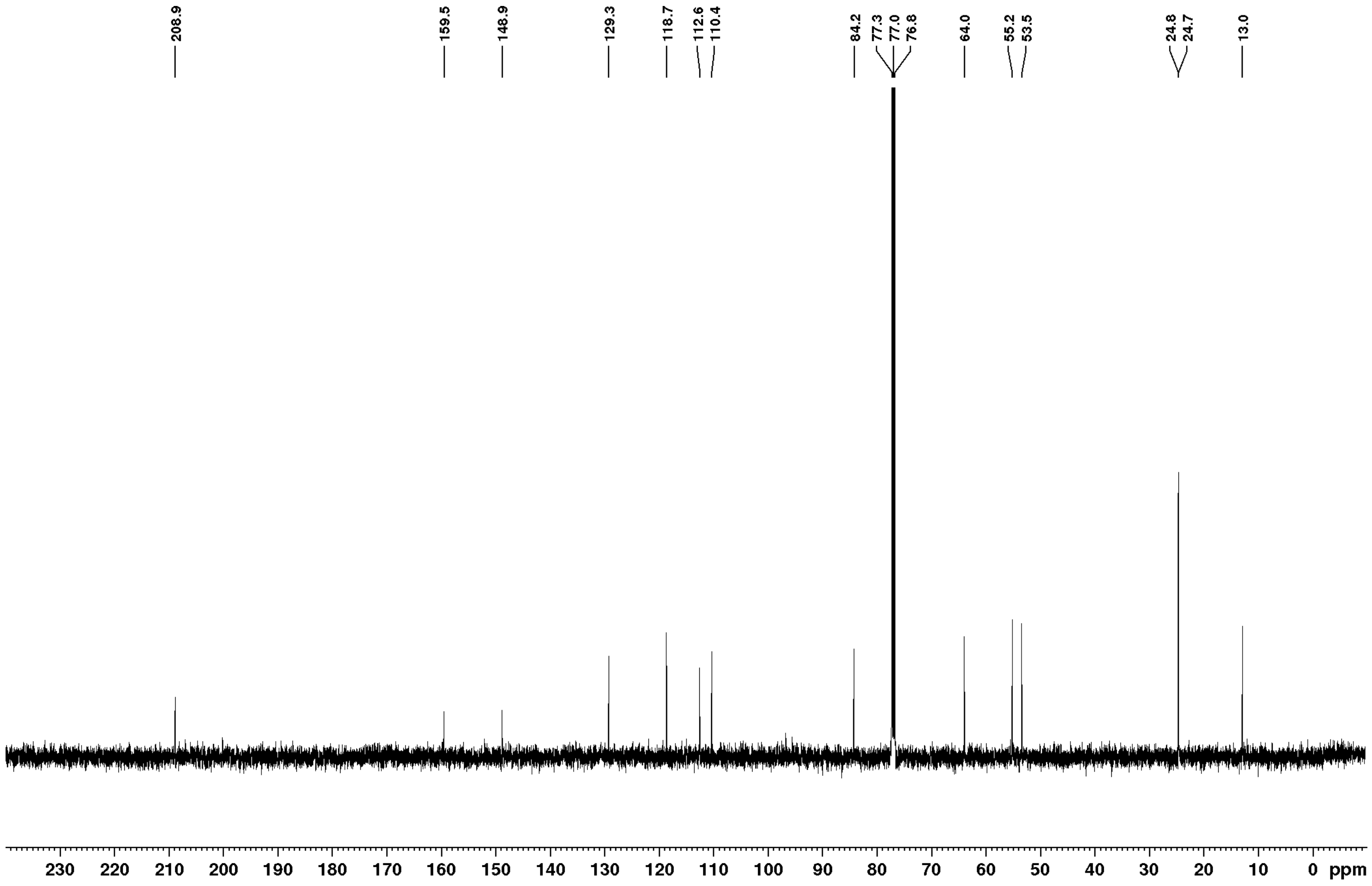

Figure S26. **^{1}H NMR** spectrum (500 MHz, $CDCl_3$, 298 K) of cyclobutylboronate **2h**.

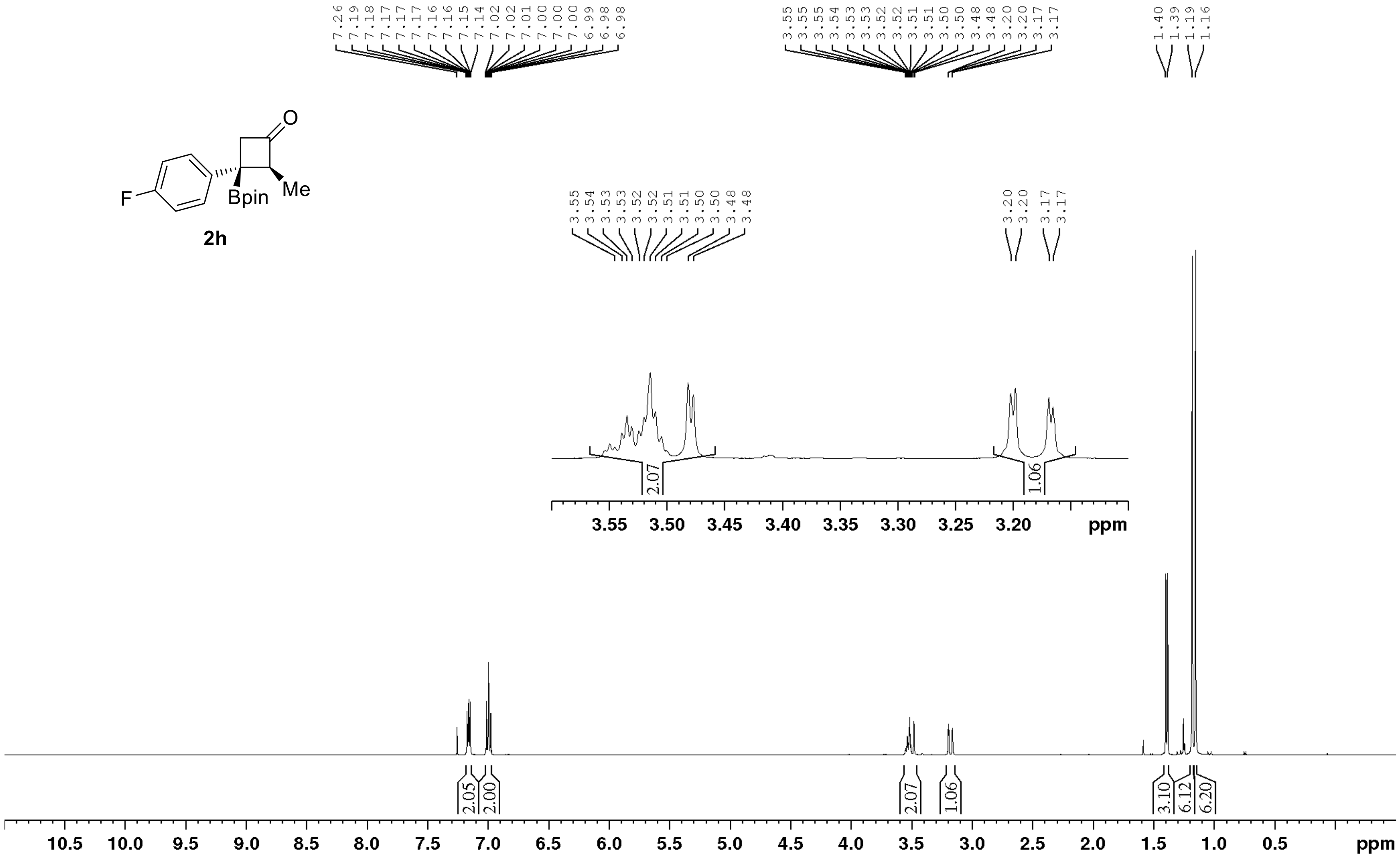

2h

Figure S27. **$^{13}C\{^{1}H\}$ NMR** spectrum (126 MHz, $CDCl_3$, 298 K) of cyclobutylboronate **2h**.

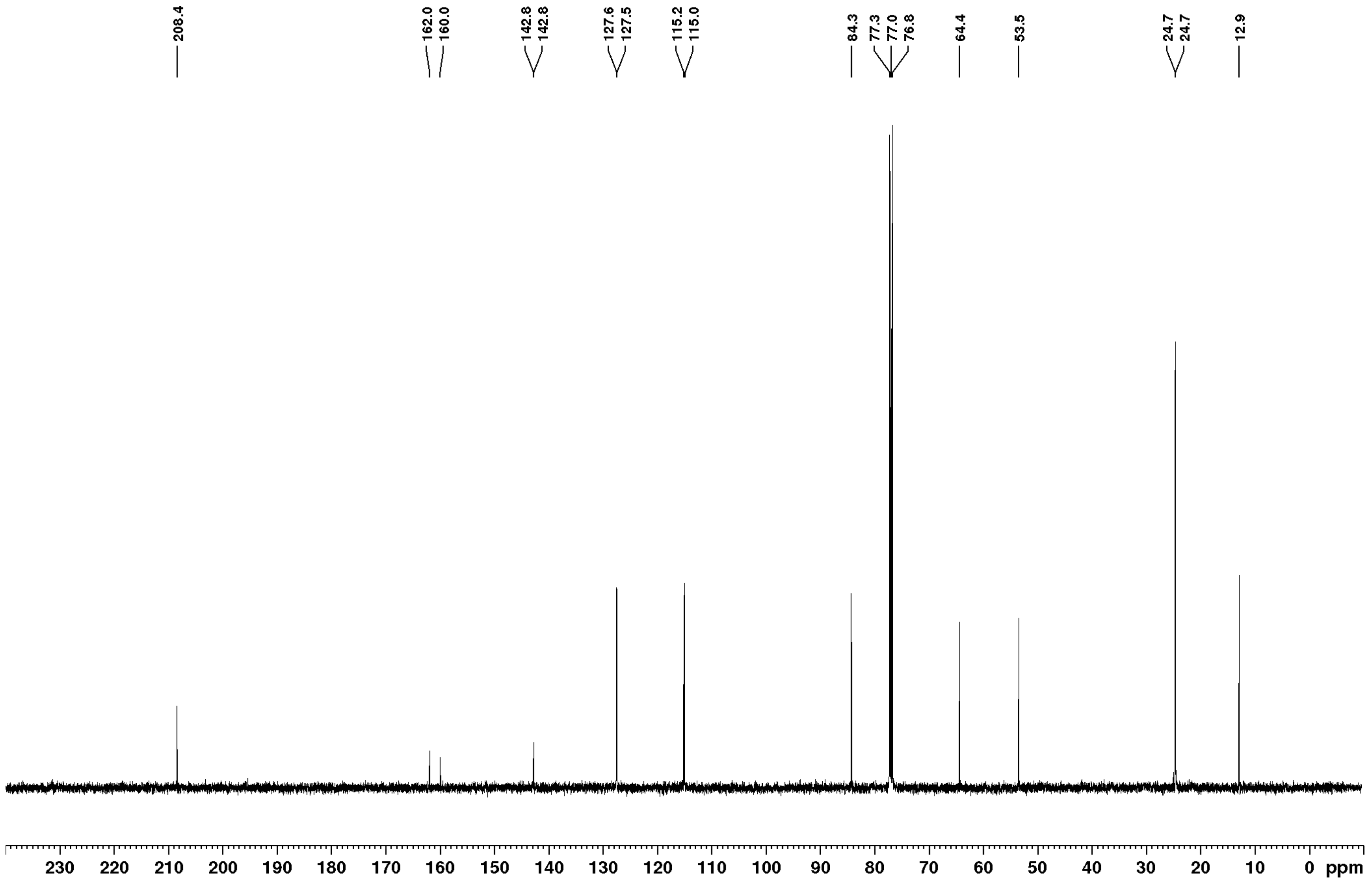

Figure S28. **^{19}F NMR** spectrum (471 MHz, $CDCl_3$, 298 K) of cyclobutylboronate **2h**.

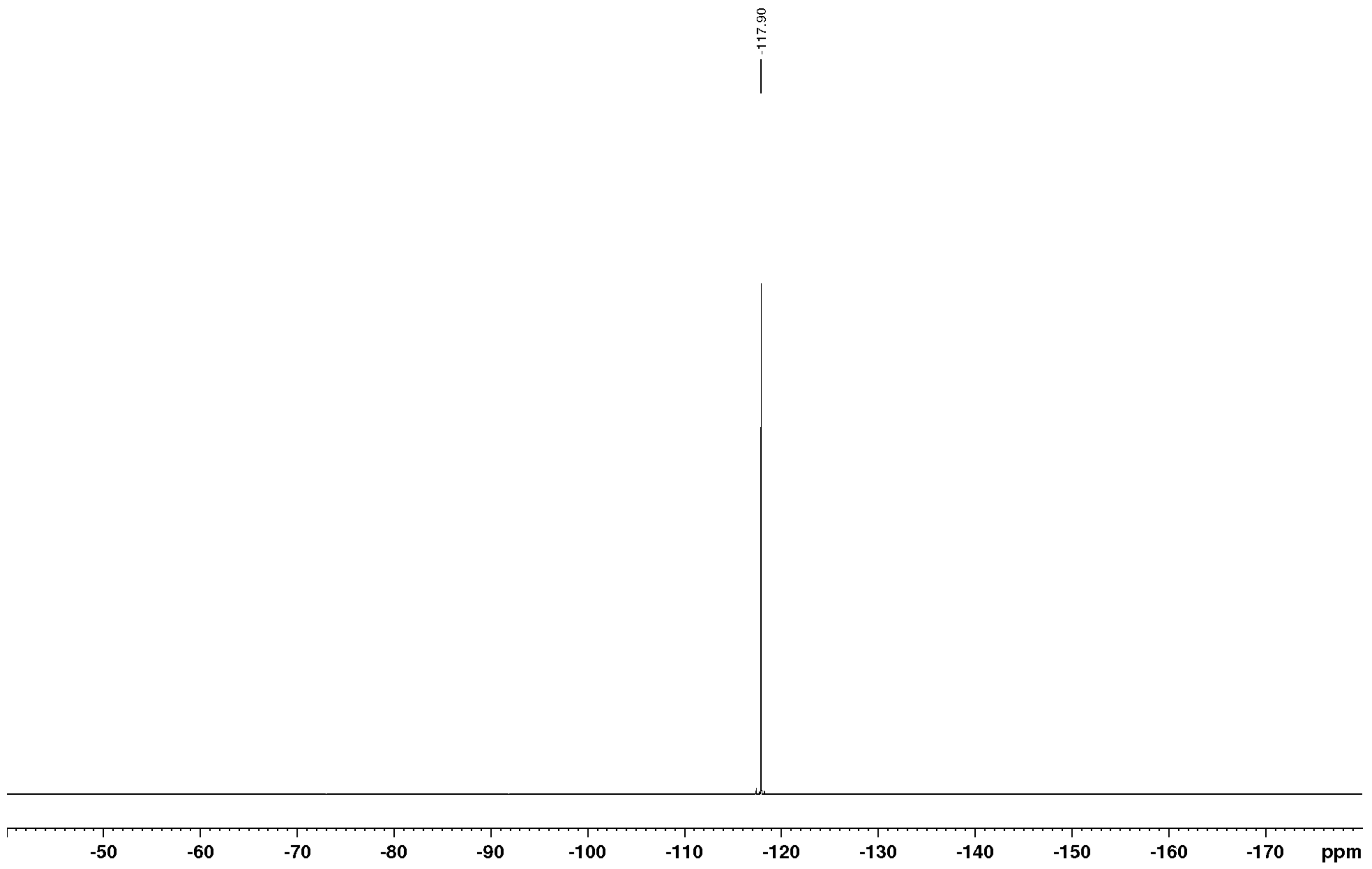

Figure S29. **^{1}H NMR** spectrum (400 MHz, $CDCl_3$, 298 K) of cyclobutylboronate **2i**.

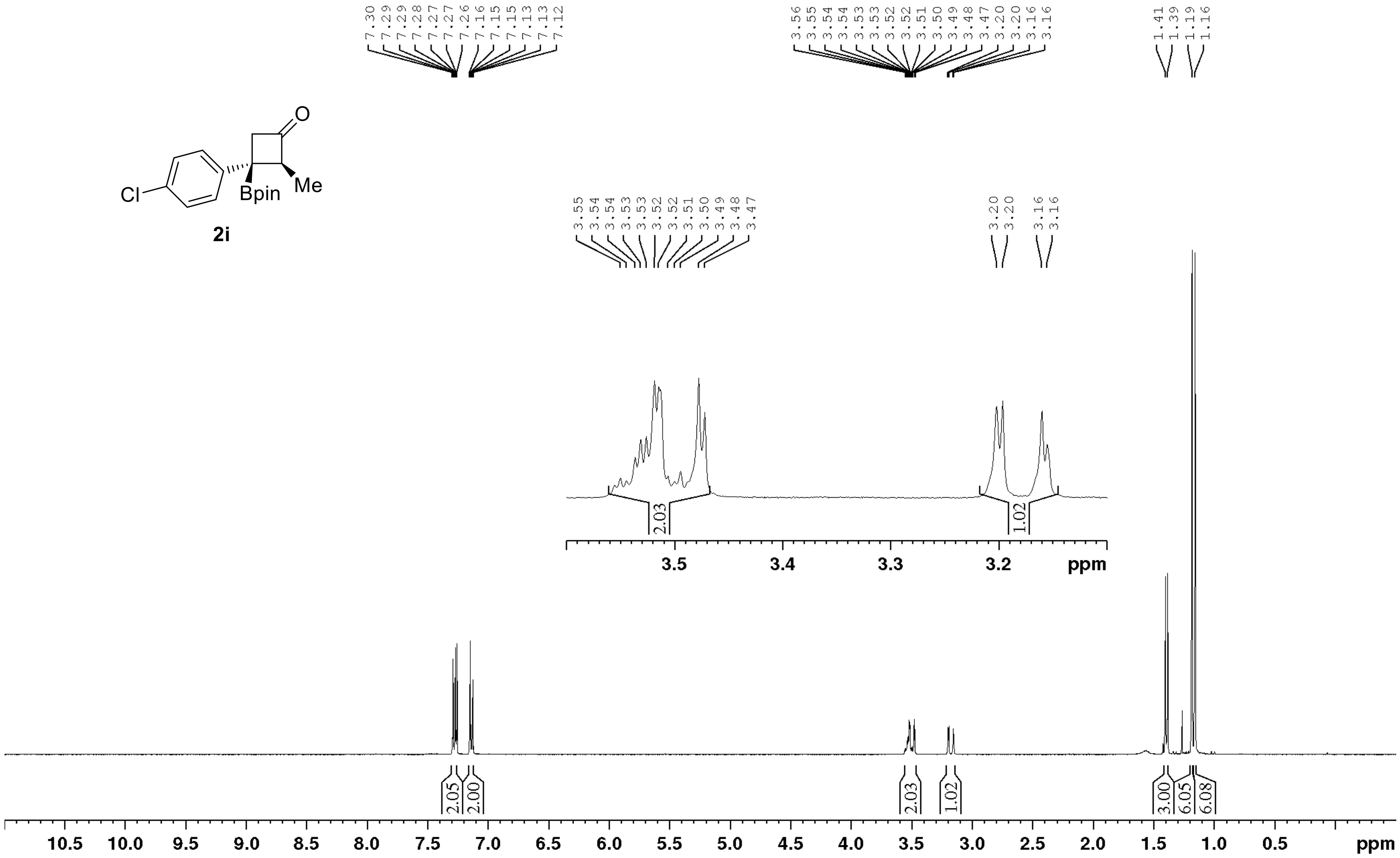

Figure S30. **^{13}C NMR** spectrum (101 MHz, $CDCl_3$, 298 K) of cyclobutylboronate **2i**.

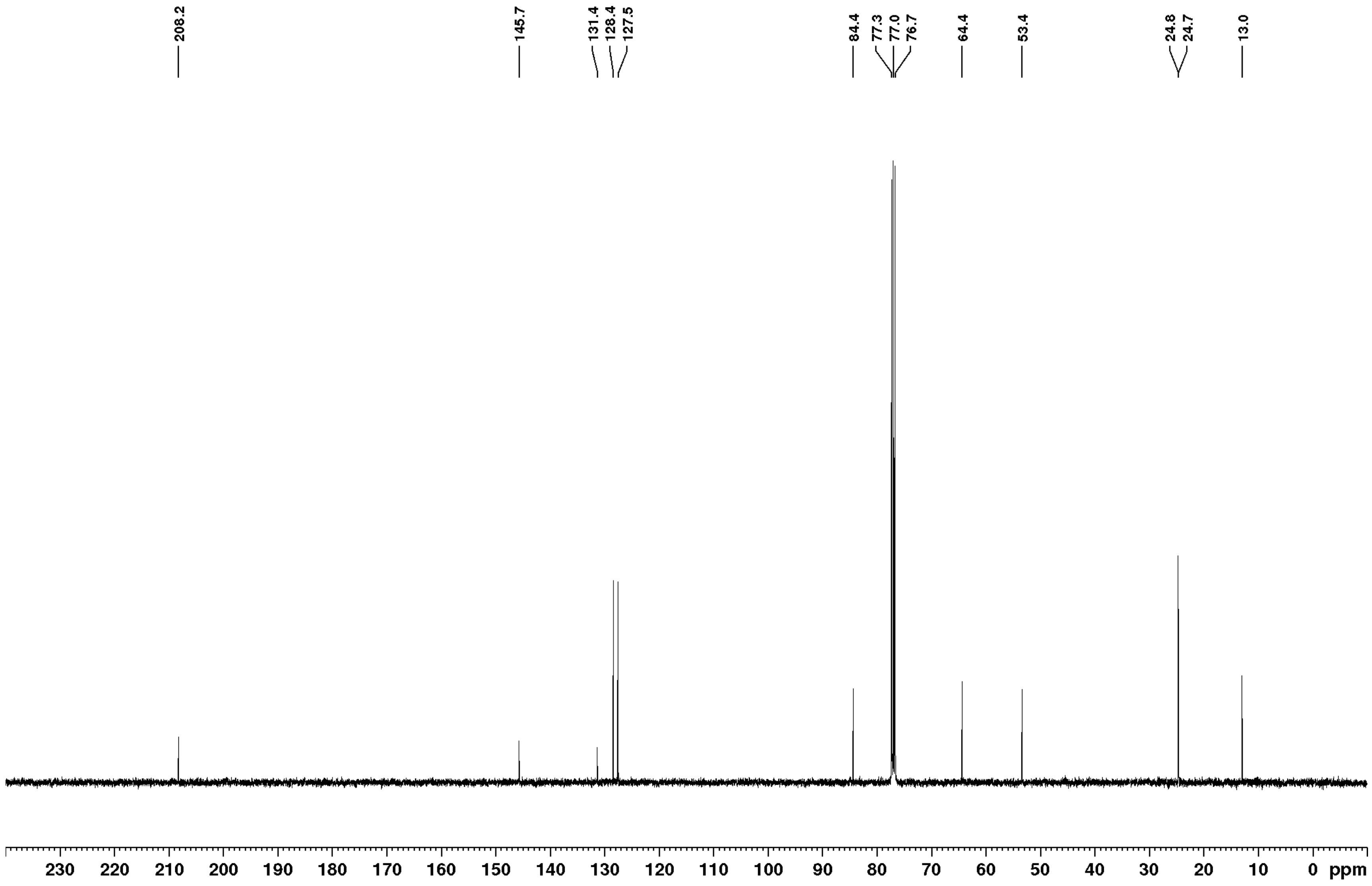

Figure S31. **^{1}H NMR** spectrum (500 MHz, $CDCl_3$, 298 K) of cyclobutylboronate **2j**.

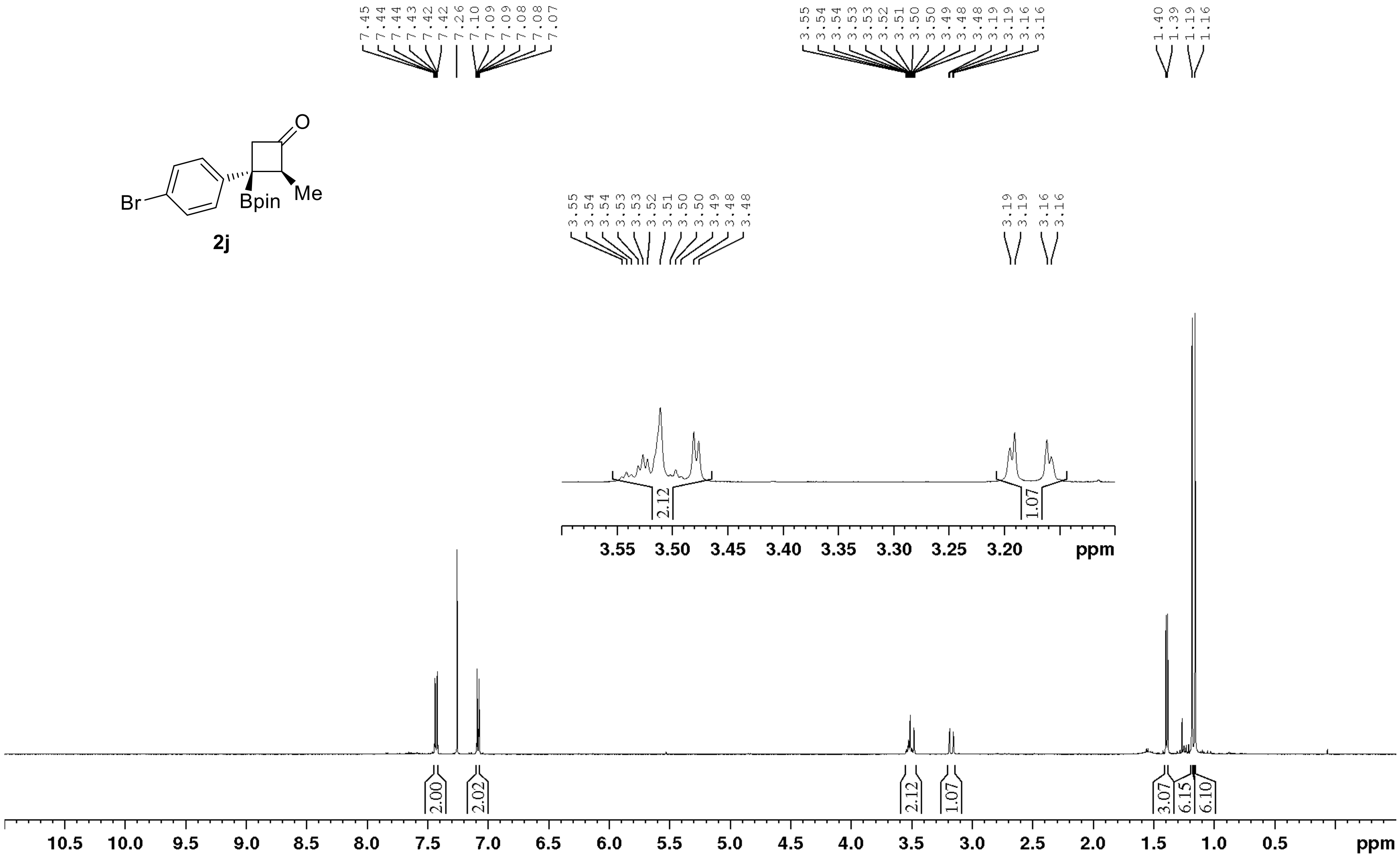

Figure S32. **$^{13}C\{^{1}H\}$ NMR** spectrum (101 MHz, $CDCl_3$, 298 K) of cyclobutylboronate **2j**.

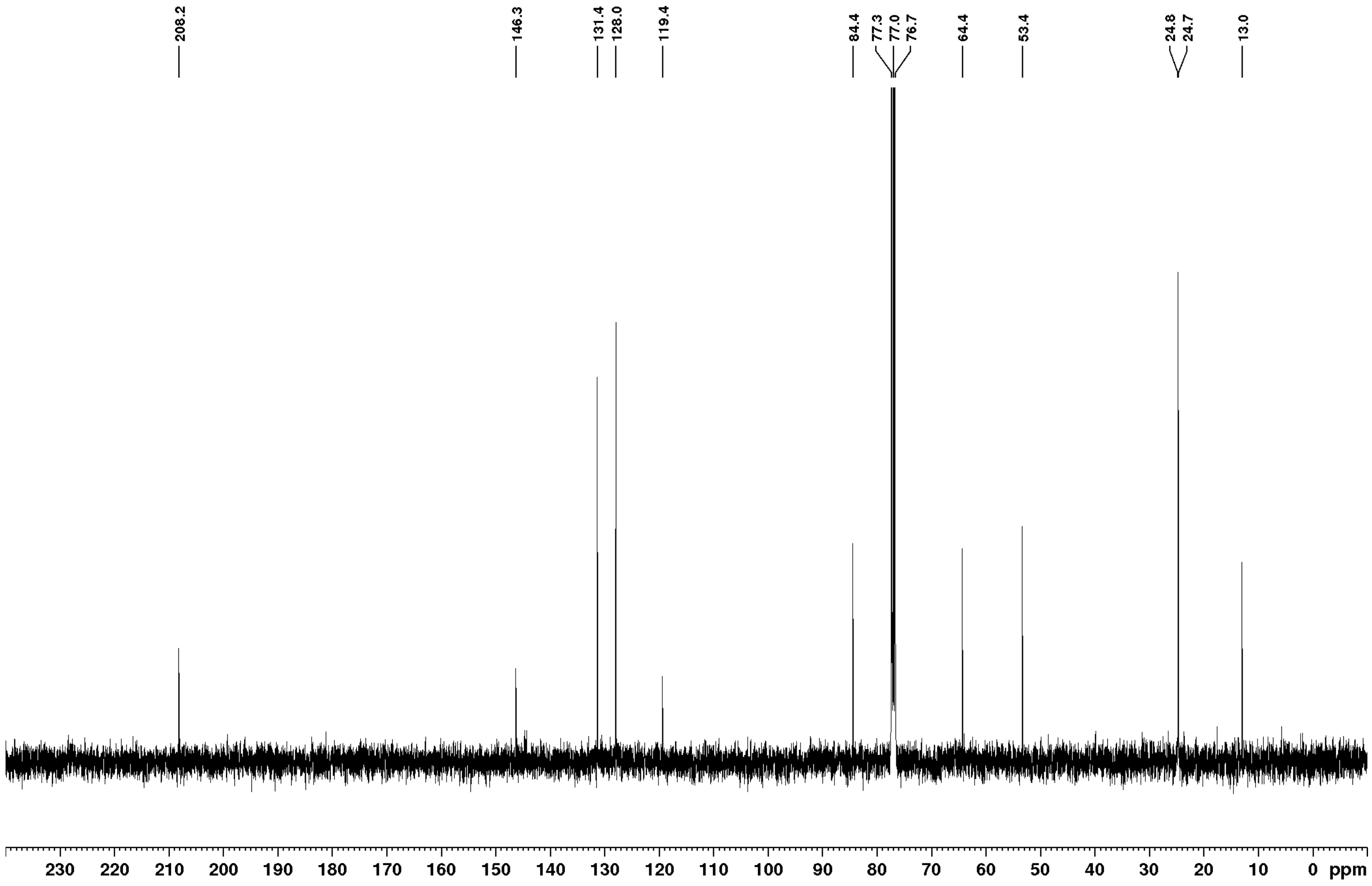

Figure S33. **^{1}H NMR** spectrum (500 MHz, $CDCl_3$, 298 K) of cyclobutylboronate **2k**.

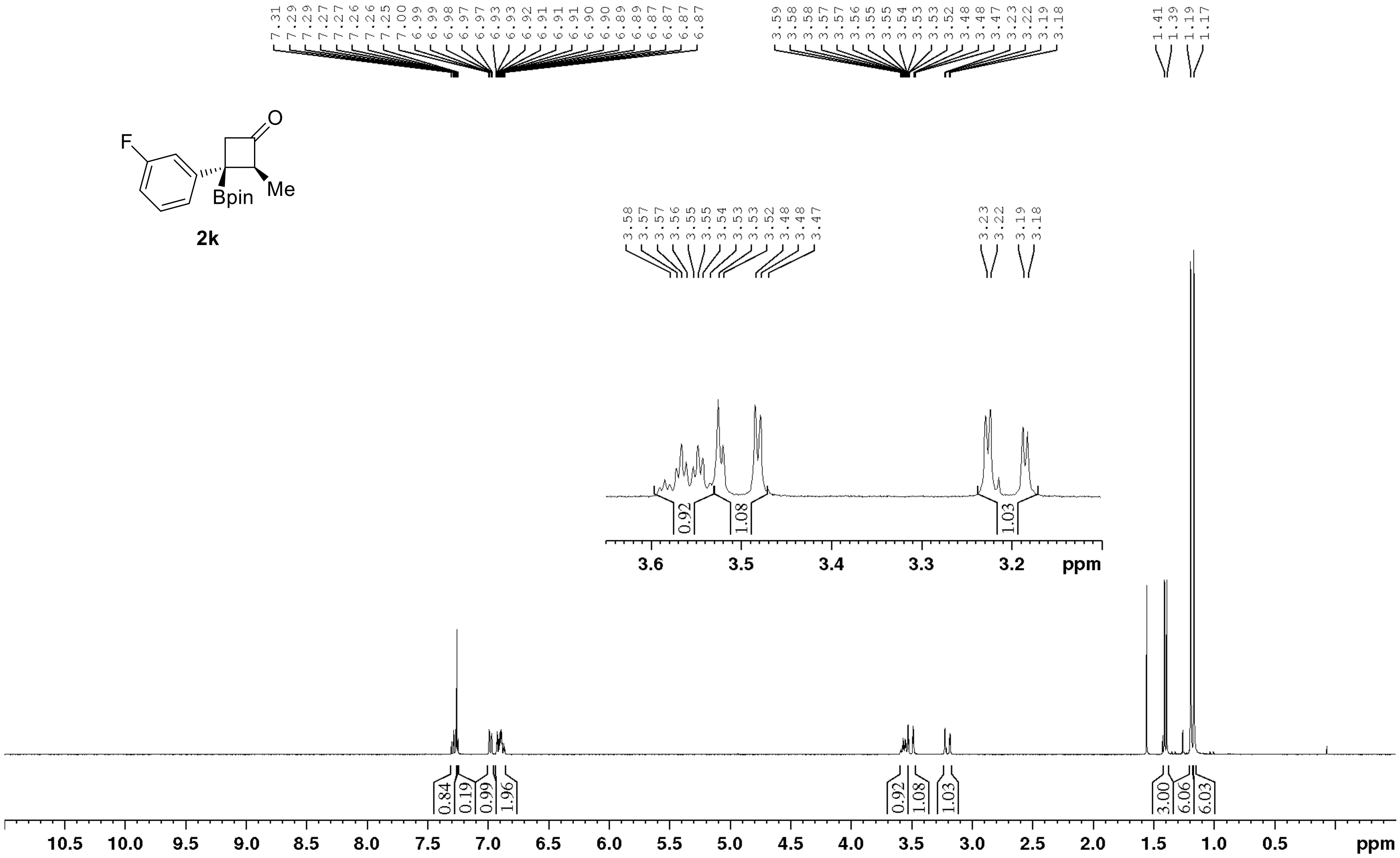

Figure S34. **$^{13}C\{^1H\}$ NMR** spectrum (101 MHz, $CDCl_3$, 298 K) of cyclobutylboronate **2k**.

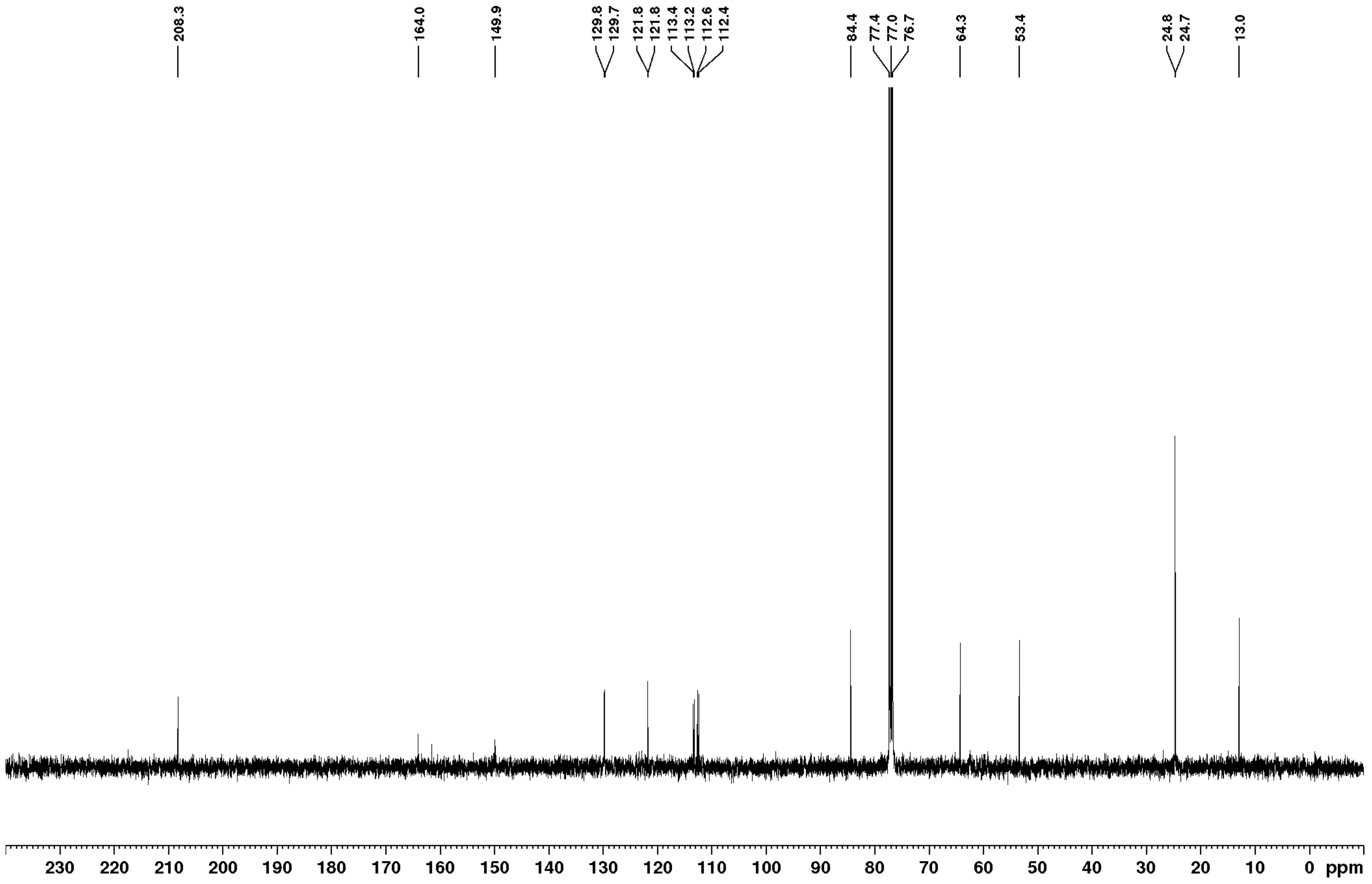

Figure S35. **^{19}F NMR** spectrum (471 MHz, $CDCl_3$, 298 K) of cyclobutylboronate **2k**.

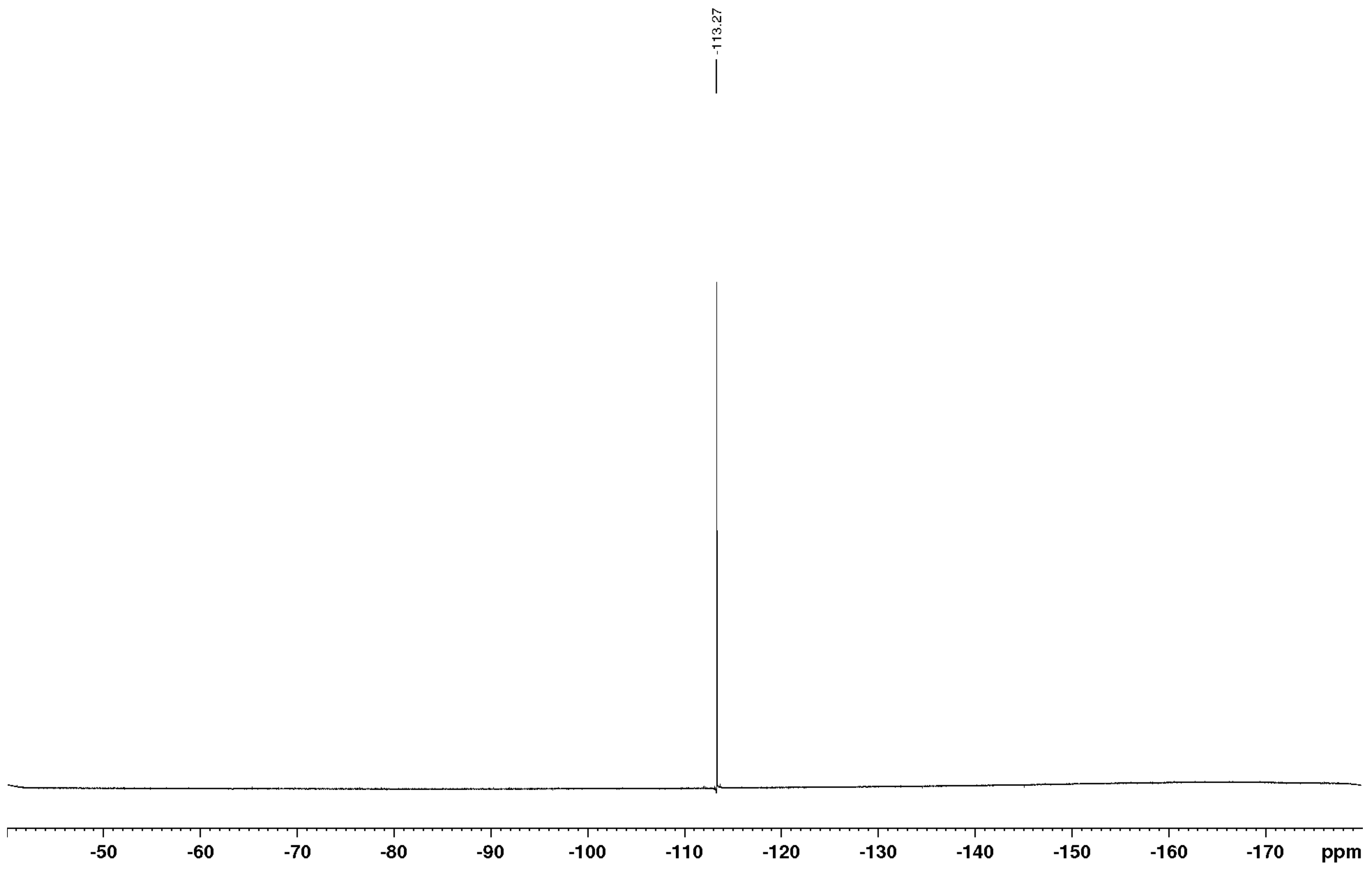

Figure S36. **^{1}H NMR** spectrum (500 MHz, $CDCl_3$, 298 K) of cyclobutylboronate **2l**.

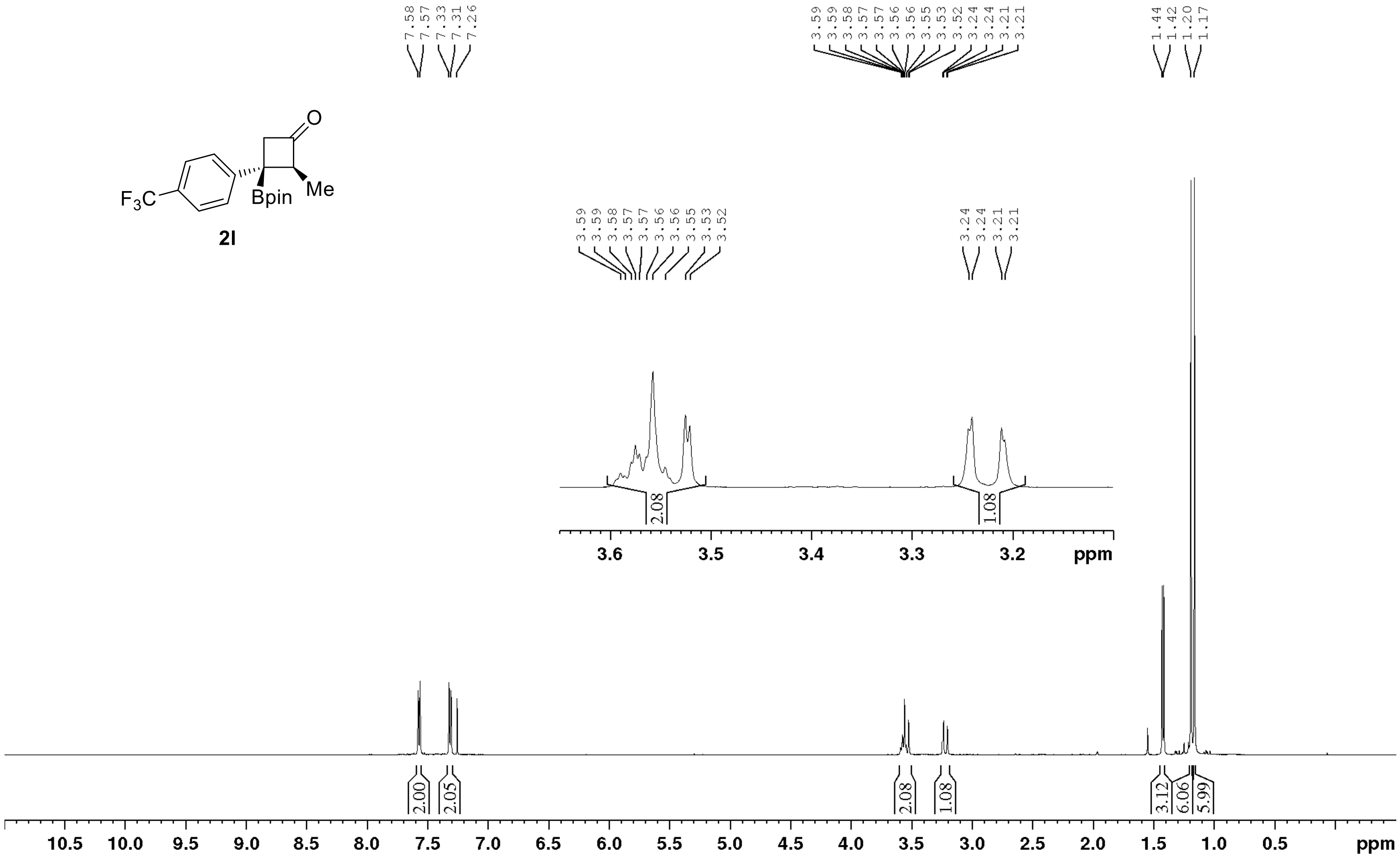

Figure S37. **$^{13}C\{^1H\}$ NMR** spectrum (126 MHz, $CDCl_3$, 298 K) of cyclobutylboronate **2l**.

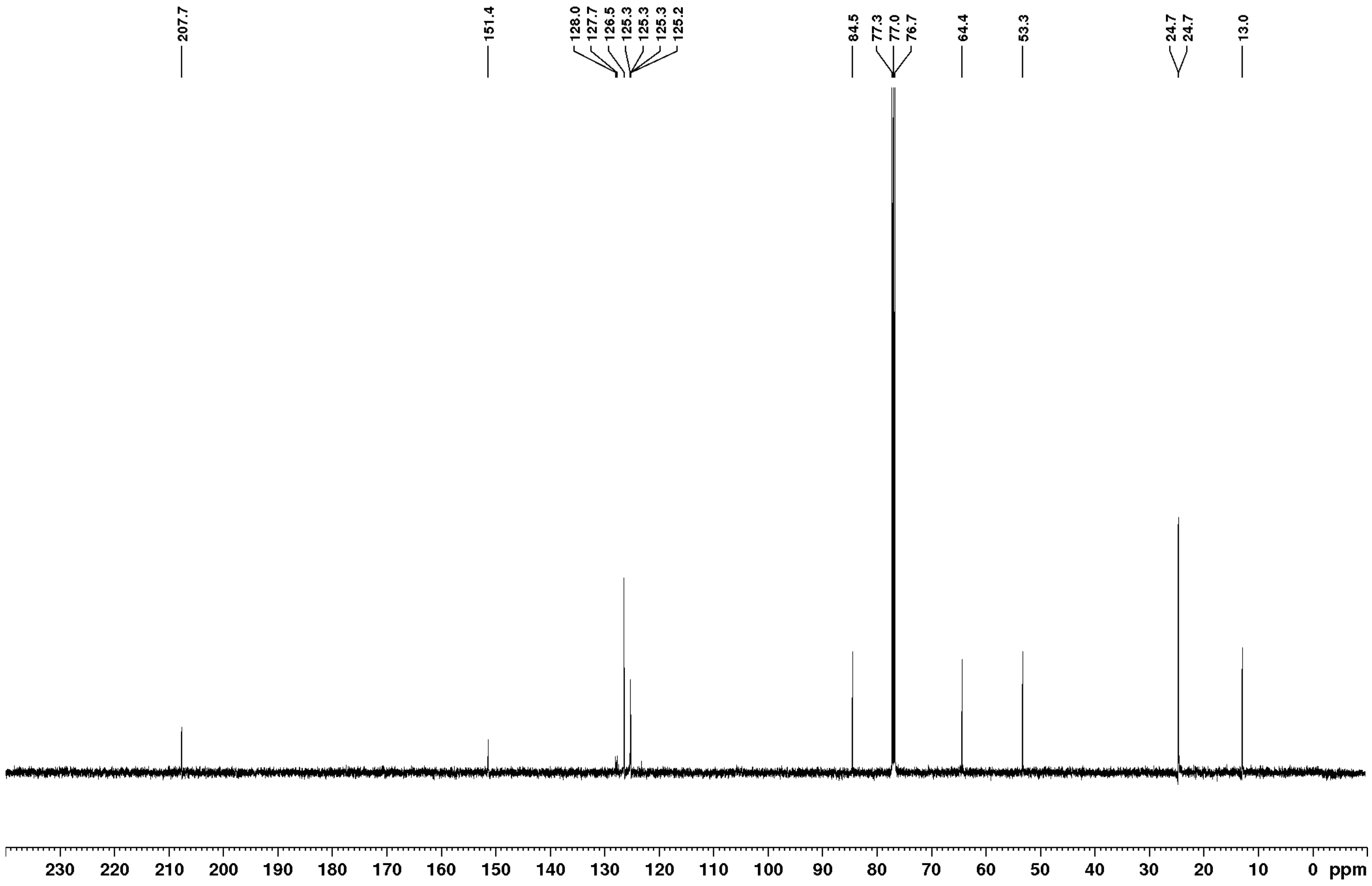

Figure S38. **^{19}F NMR** spectrum (471 MHz, $CDCl_3$, 298 K) of cyclobutylboronate **2l**.

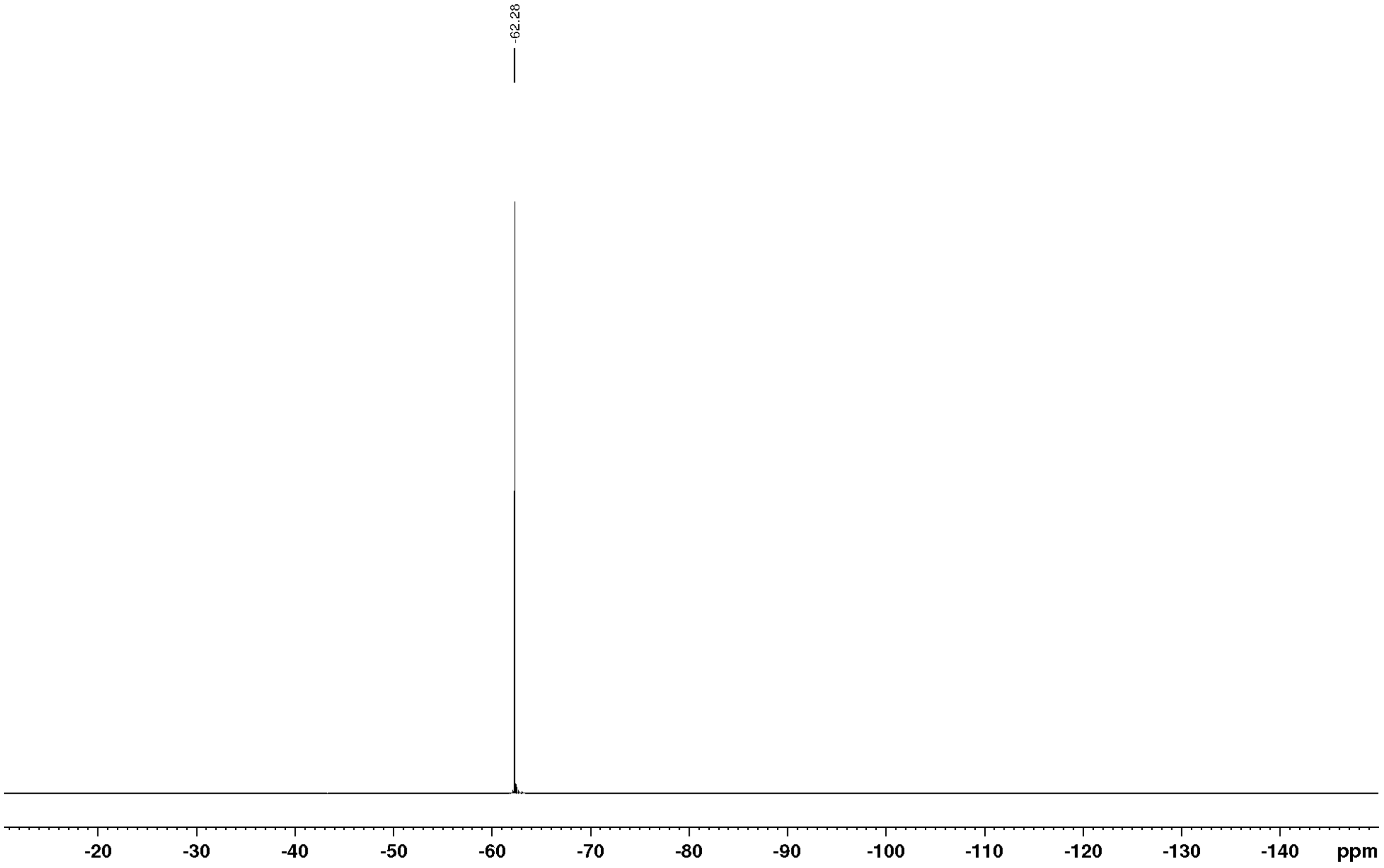

Figure S39. **^{1}H NMR** spectrum (500 MHz, $CDCl_3$, 298 K) of cyclobutylboronate **2m**.

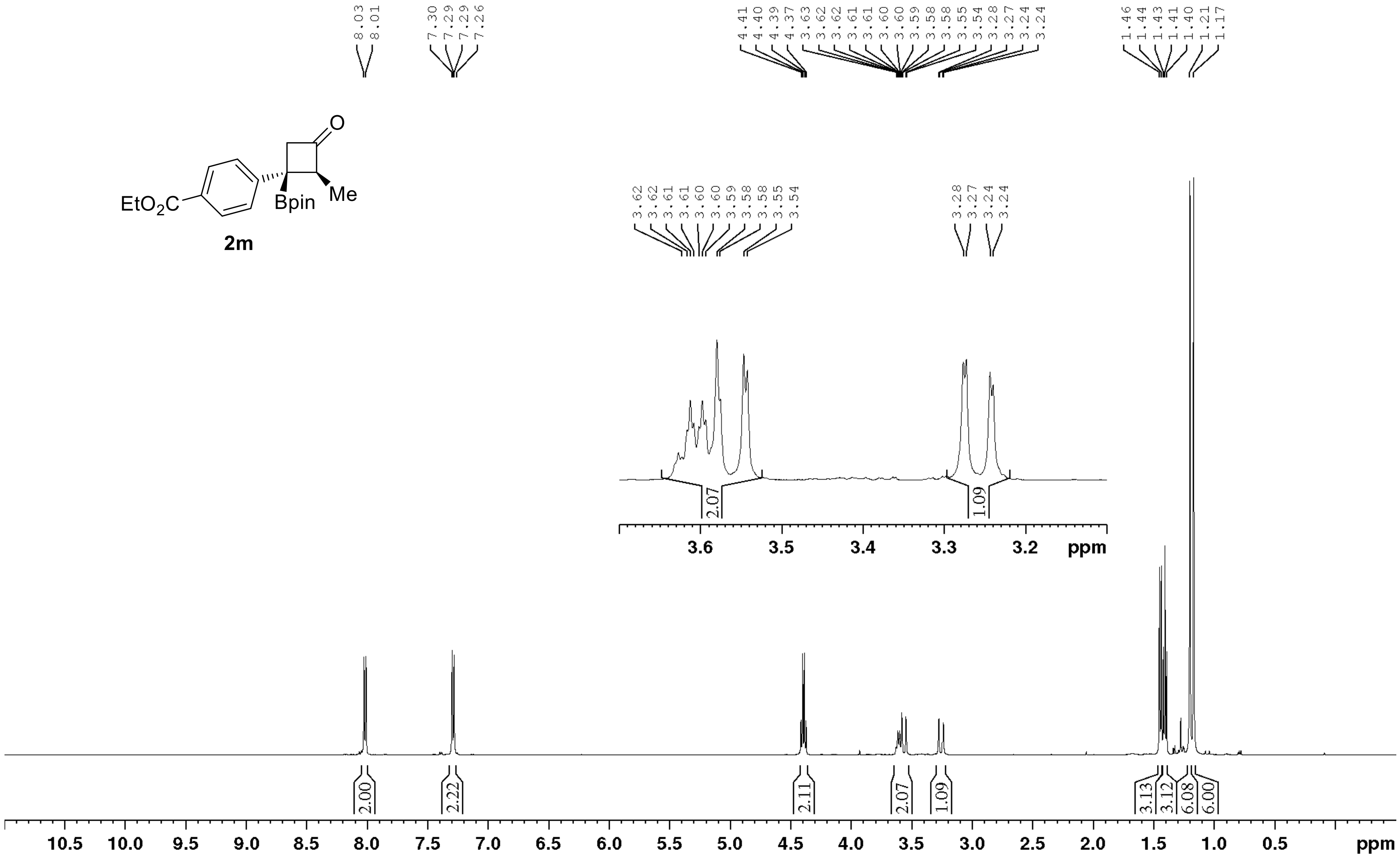

Figure S40. **$^{13}C\{^1H\}$ NMR** spectrum (126 MHz, $CDCl_3$, 298 K) of cyclobutylboronate **2m**.

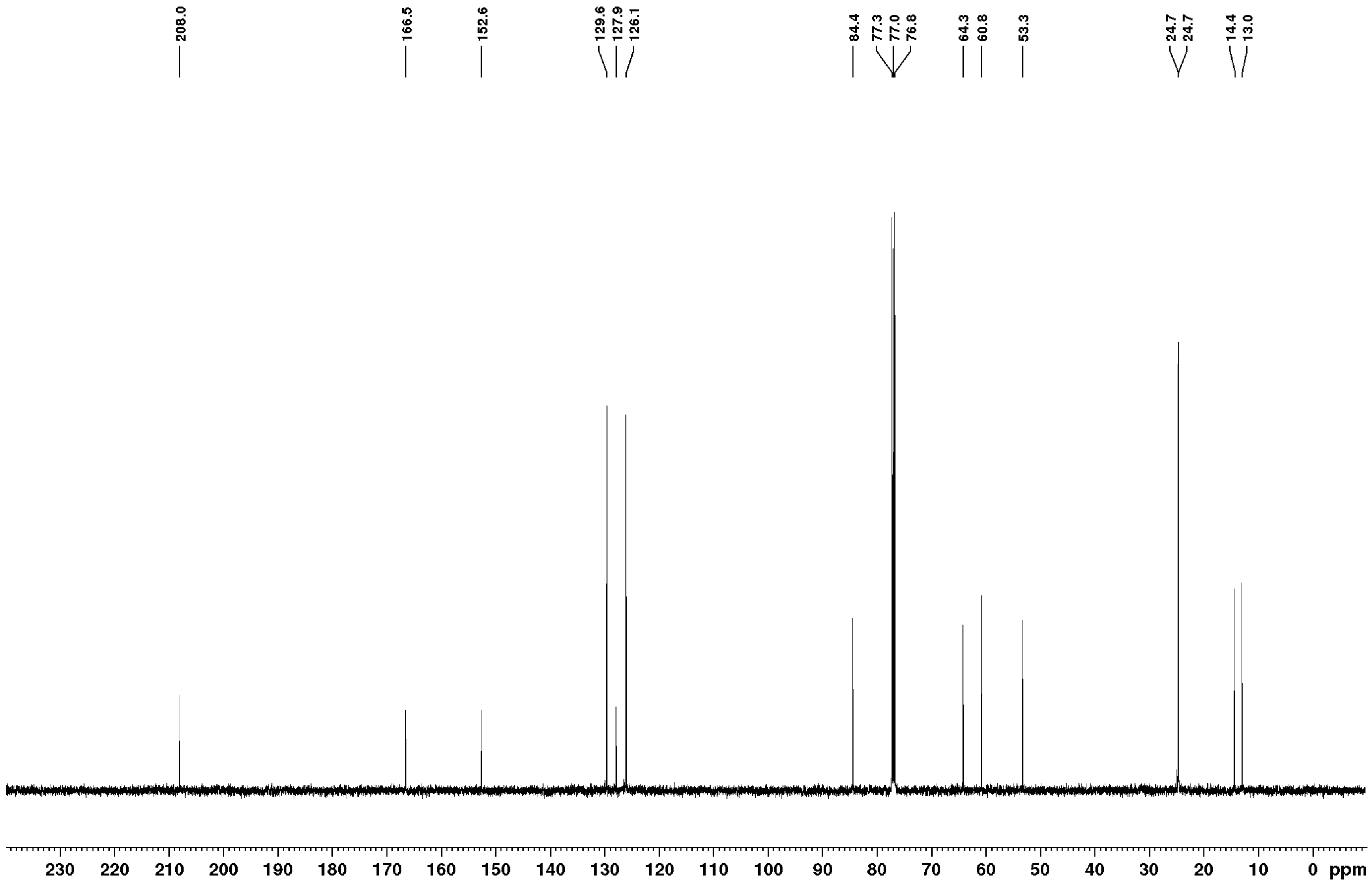

Figure S41. **^{1}H NMR** spectrum (500 MHz, $CDCl_3$, 298 K) of cyclobutylboronate **2n**.

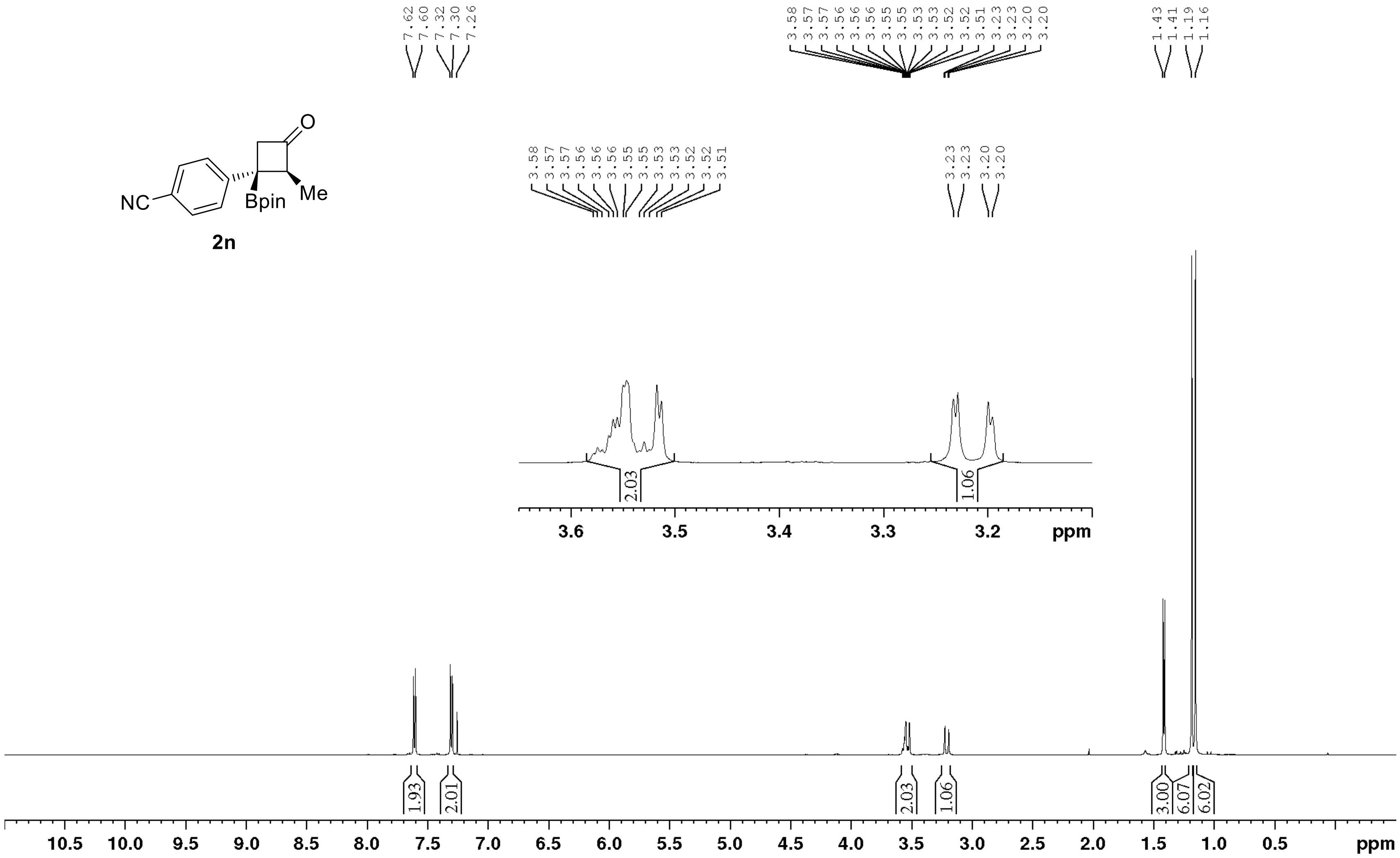

Figure S42. **$^{13}C\{^1H\}$ NMR** spectrum (126 MHz, $CDCl_3$, 298 K) of cyclobutylboronate **2n**.

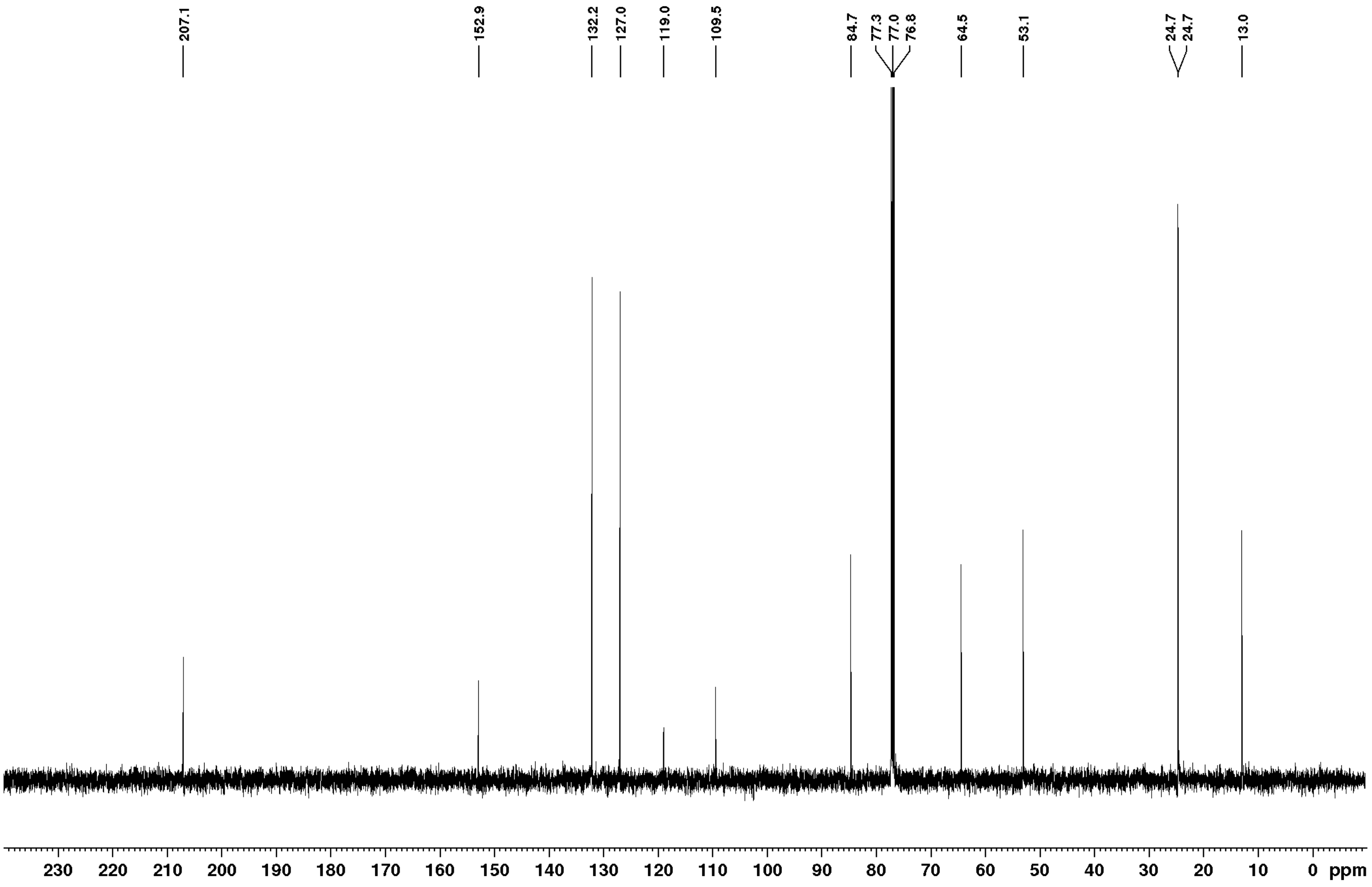

Figure S43. **^{1}H NMR** spectrum (500 MHz, $CDCl_3$, 298 K) of cyclobutylboronate **3o**.

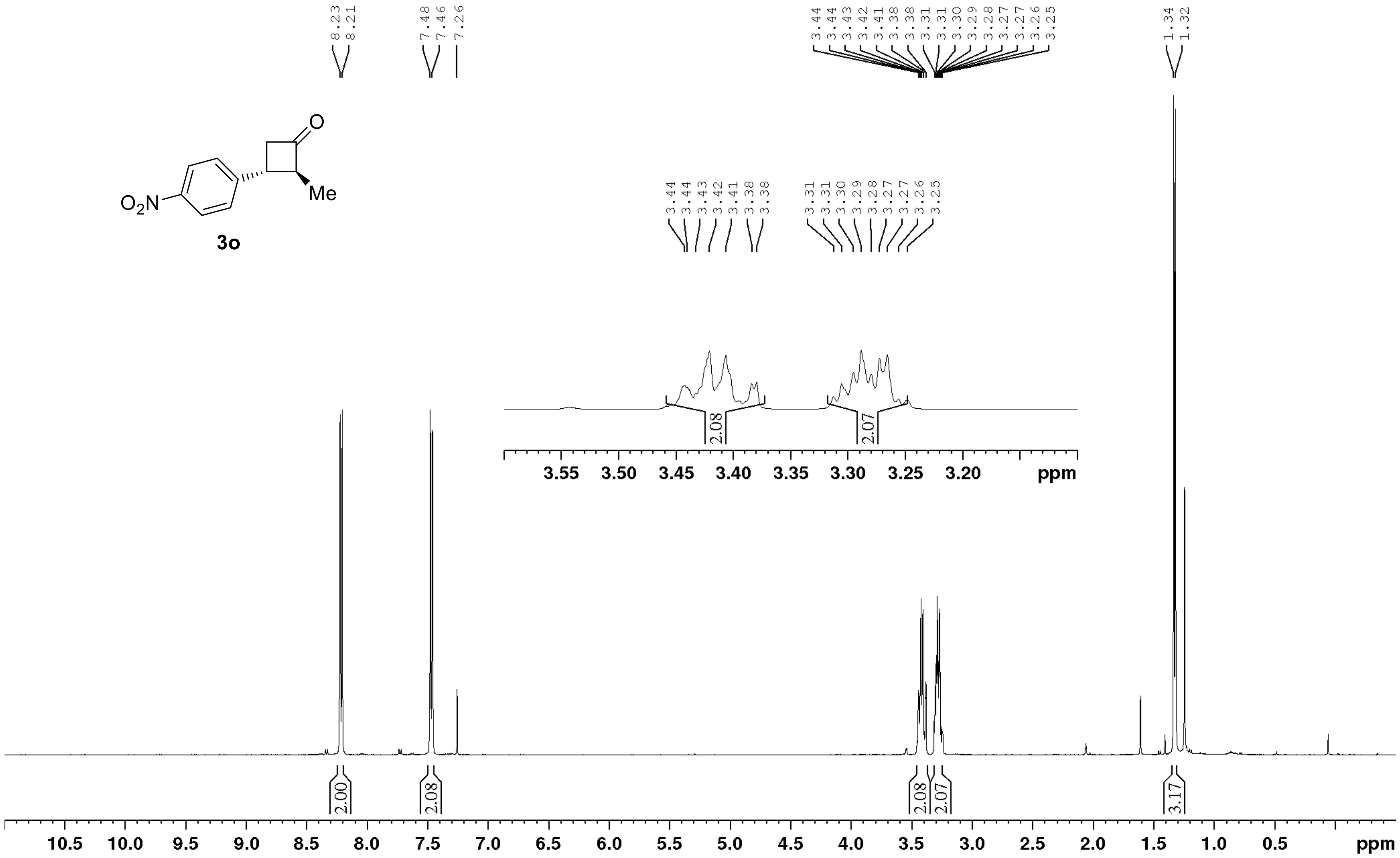

Figure S44. **^{13}C NMR** spectrum (126 MHz, $CDCl_3$, 298 K) of cyclobutylboronate **3o**.

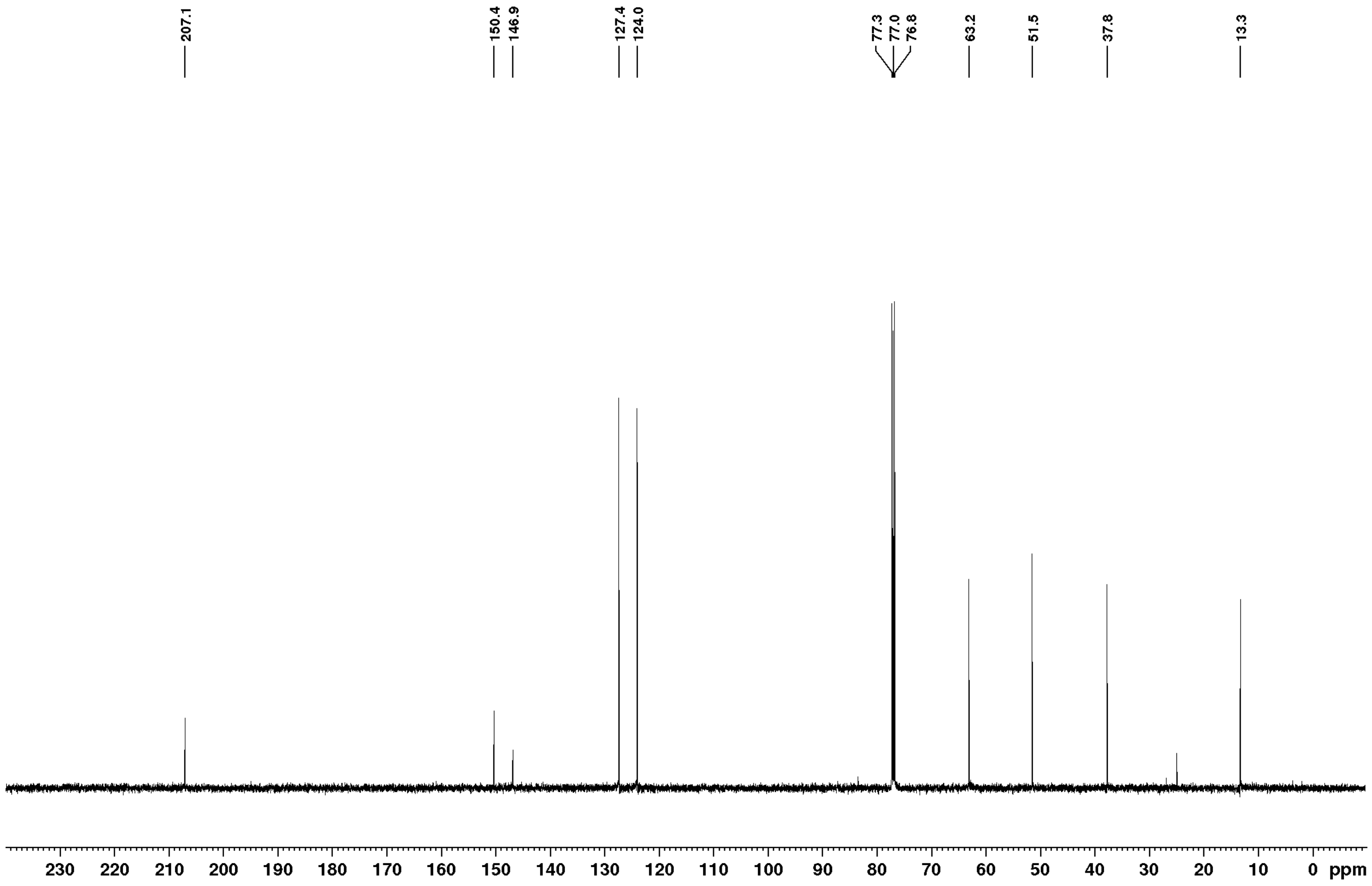

Figure S45. **^{1}H NMR** spectrum (400 MHz, $CDCl_3$, 298 K) of cyclobutylboronate **2p**.

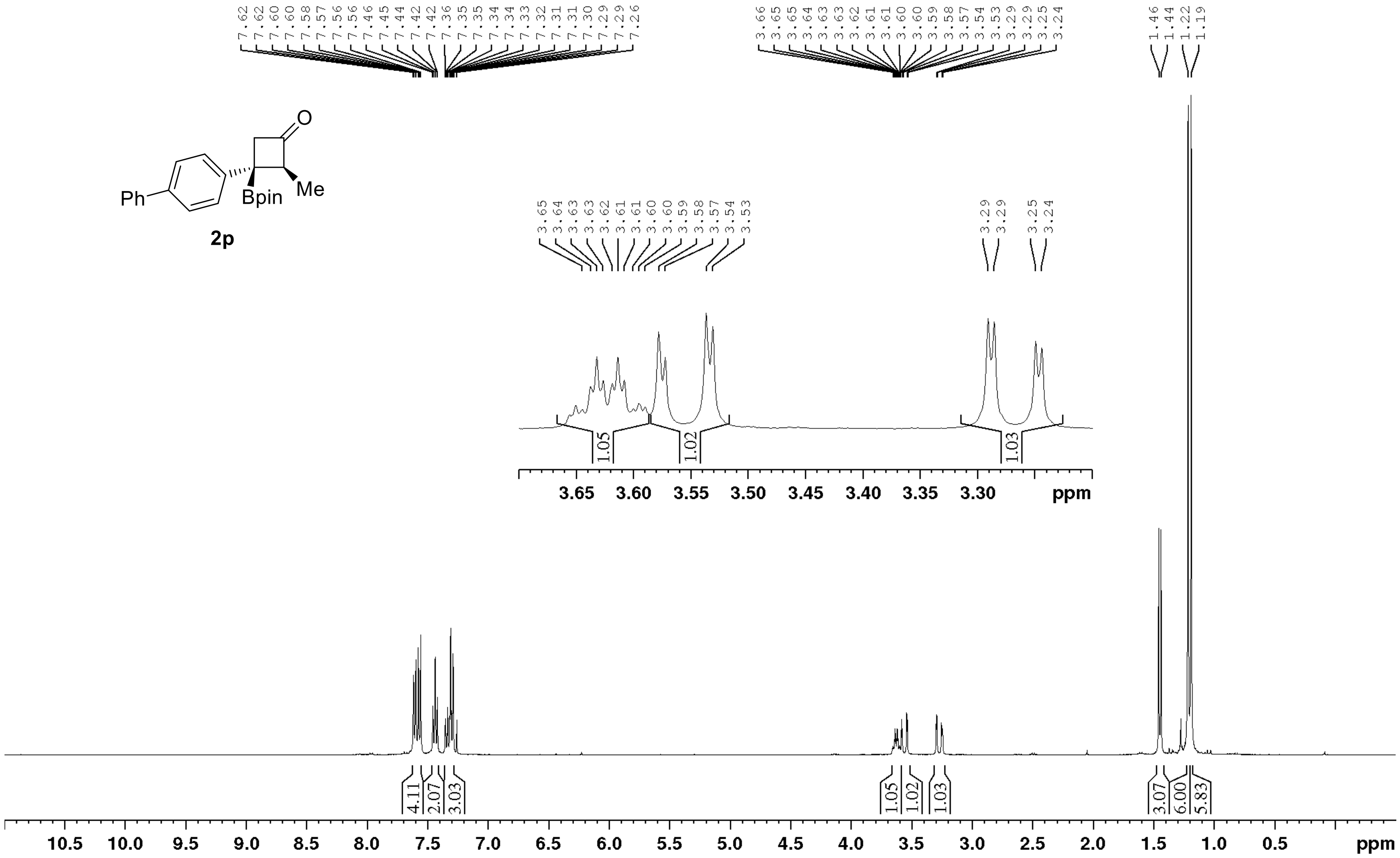

Figure S46. **$^{13}C\{^{1}H\}$ NMR** spectrum (101 MHz, $CDCl_3$, 298 K) of cyclobutylboronate **2p**.

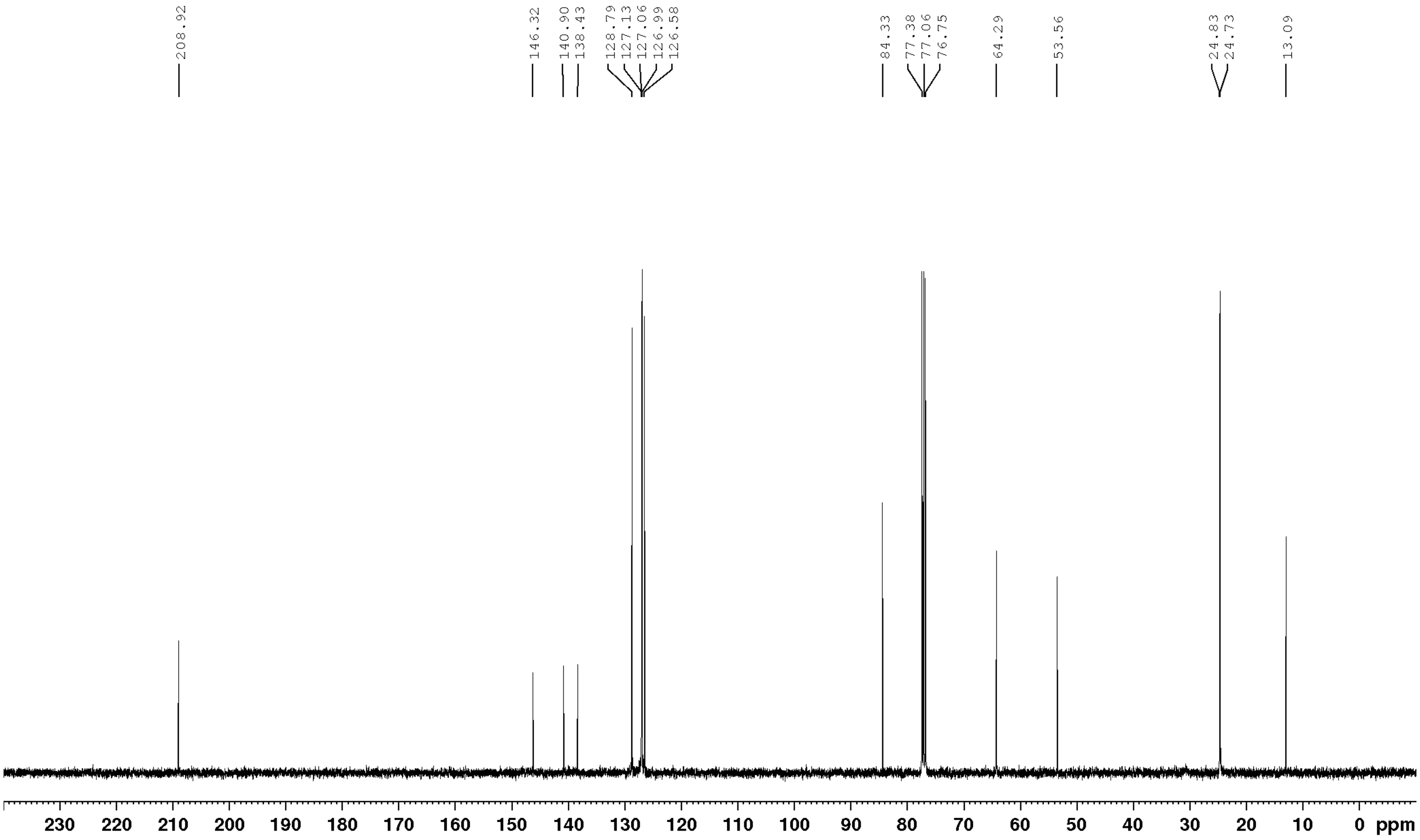

Figure S47. **^{1}H NMR** spectrum (500 MHz, $CDCl_3$, 298 K) of cyclobutylboronate **2q**.

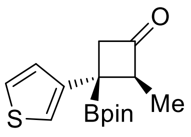

2q

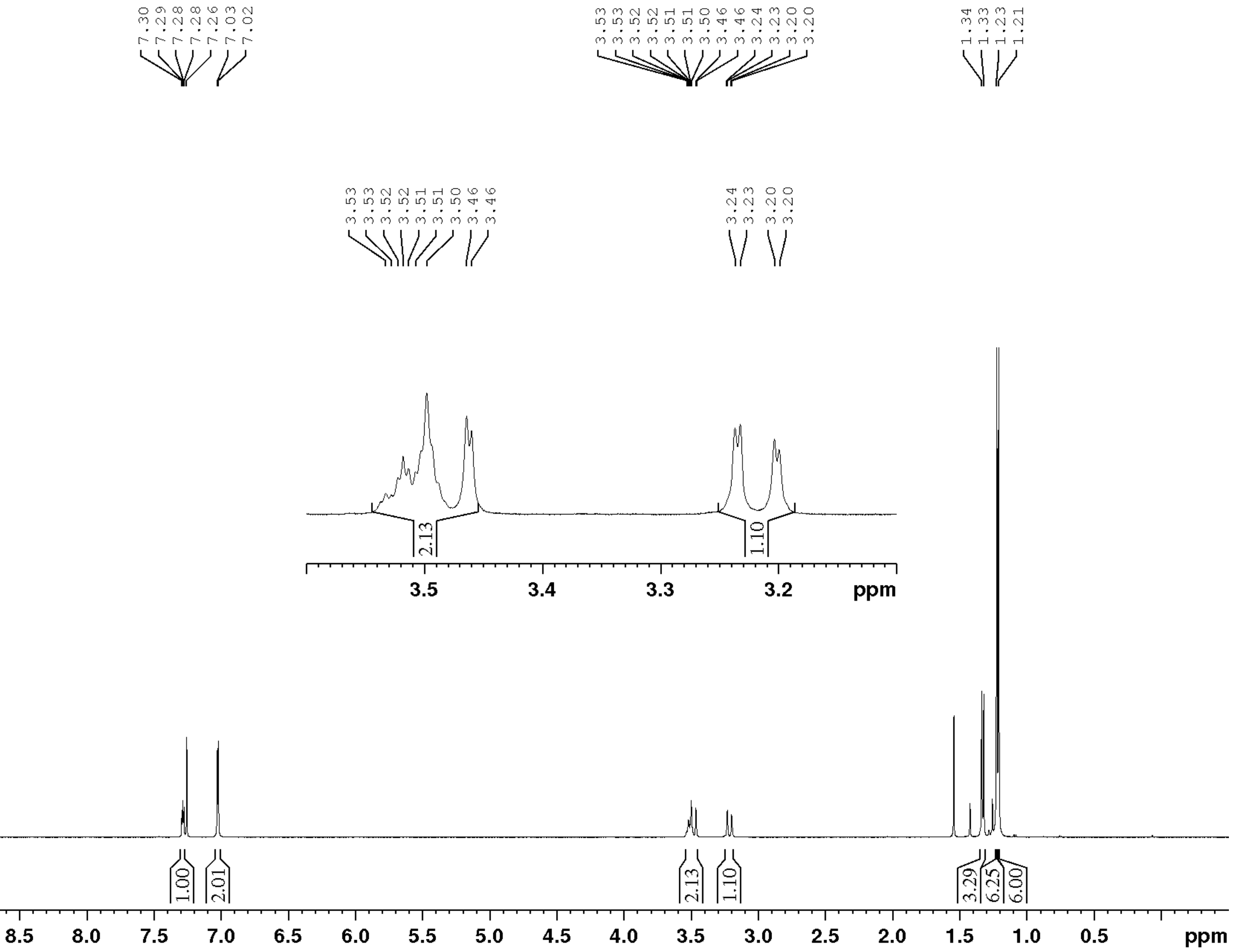

Figure S48. **$^{13}C\{^1H\}$ NMR** spectrum (126 MHz, $CDCl_3$, 298 K) of cyclobutylboronate **2q**.

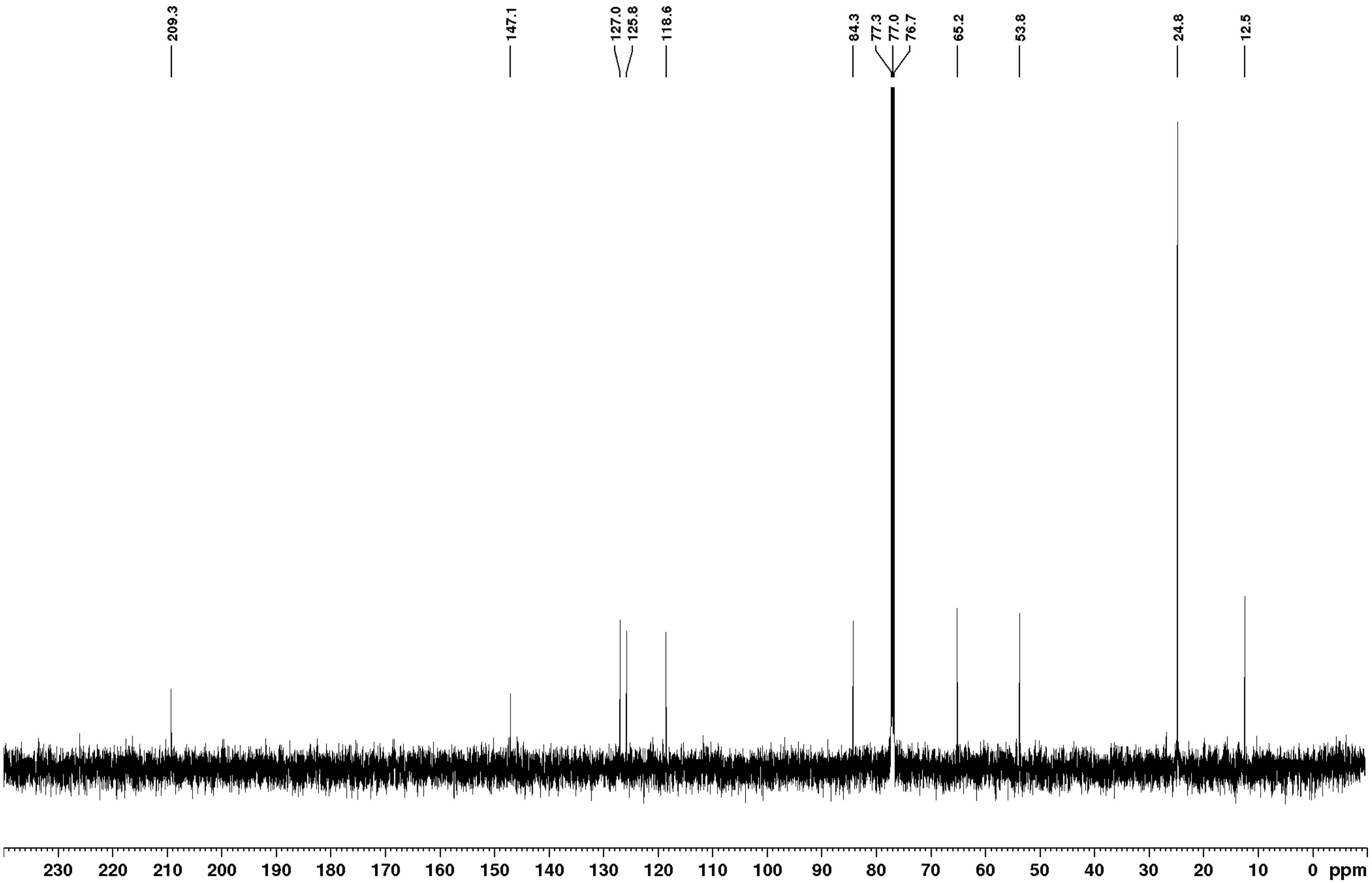

Figure S49. **^{1}H NMR** spectrum (126 MHz, $CDCl_3$, 298 K) of cyclobutylboronate **2r**.

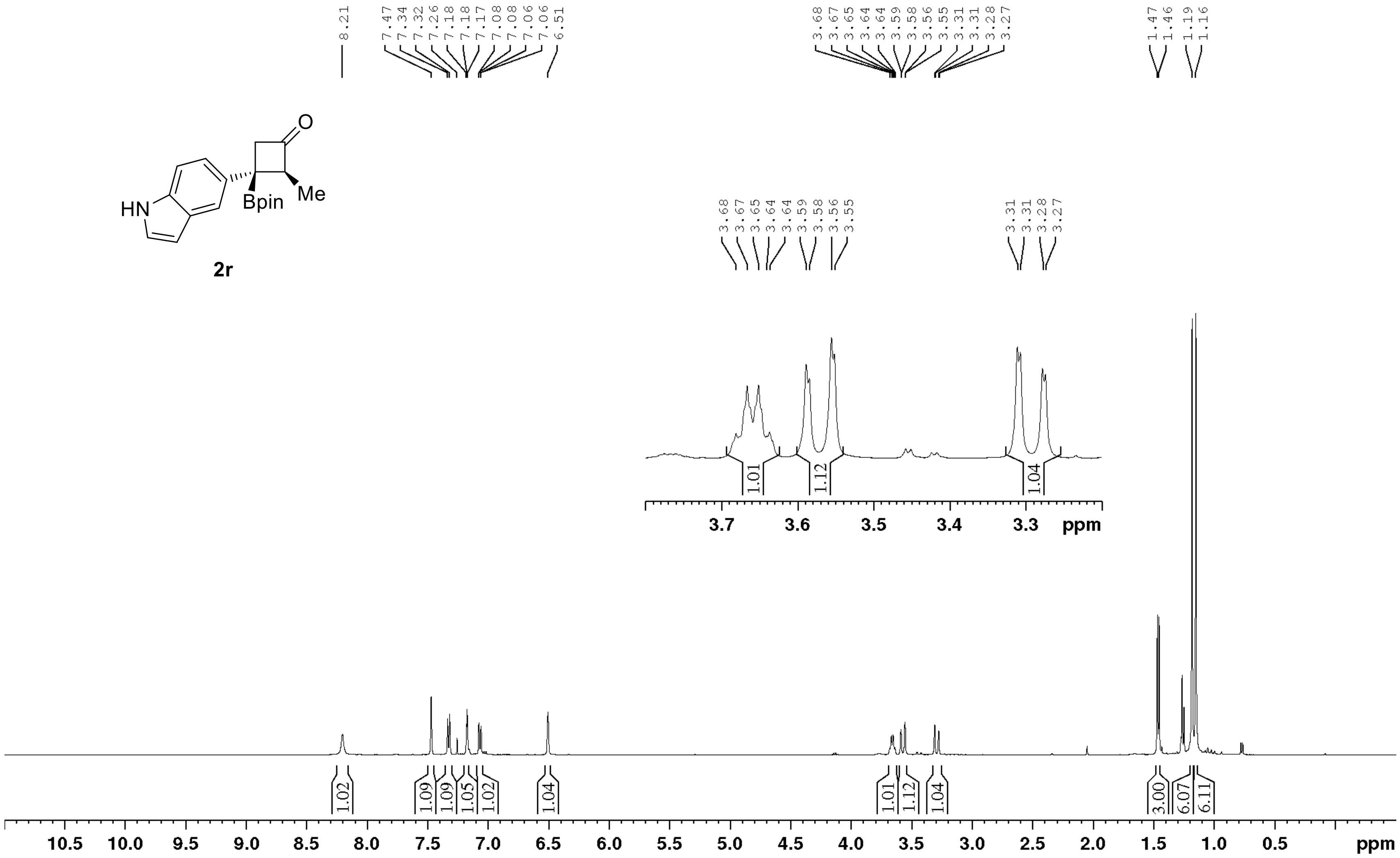

Figure S50. **$^{13}C\{^1H\}$ NMR** spectrum (126 MHz, $CDCl_3$, 298 K) of cyclobutylboronate **2r**.

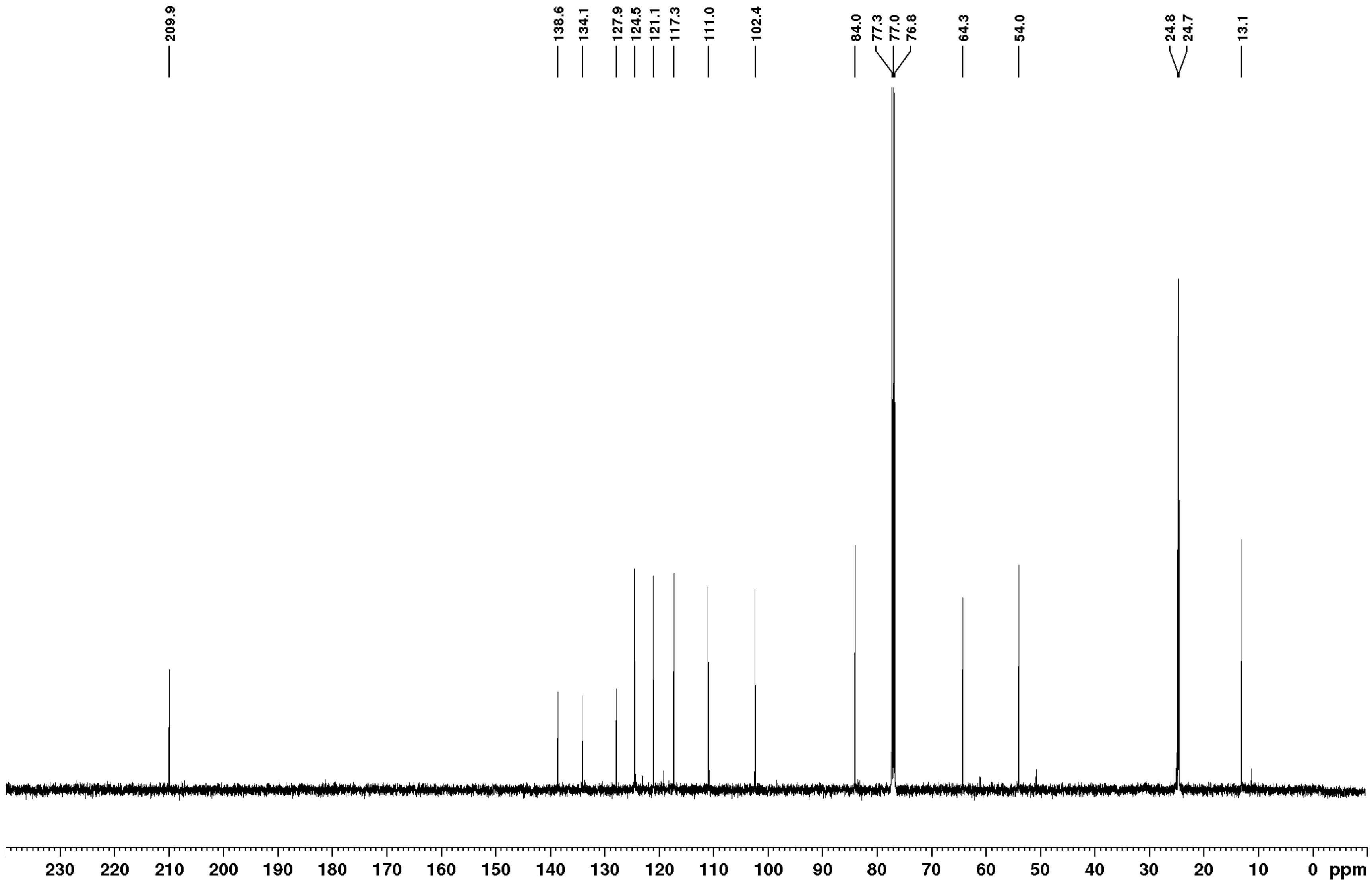

Figure S51. **^{1}H NMR** spectrum (500 MHz, $CDCl_3$, 298 K) of cyclobutylboronate **2s**.

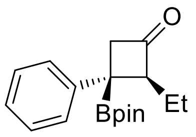

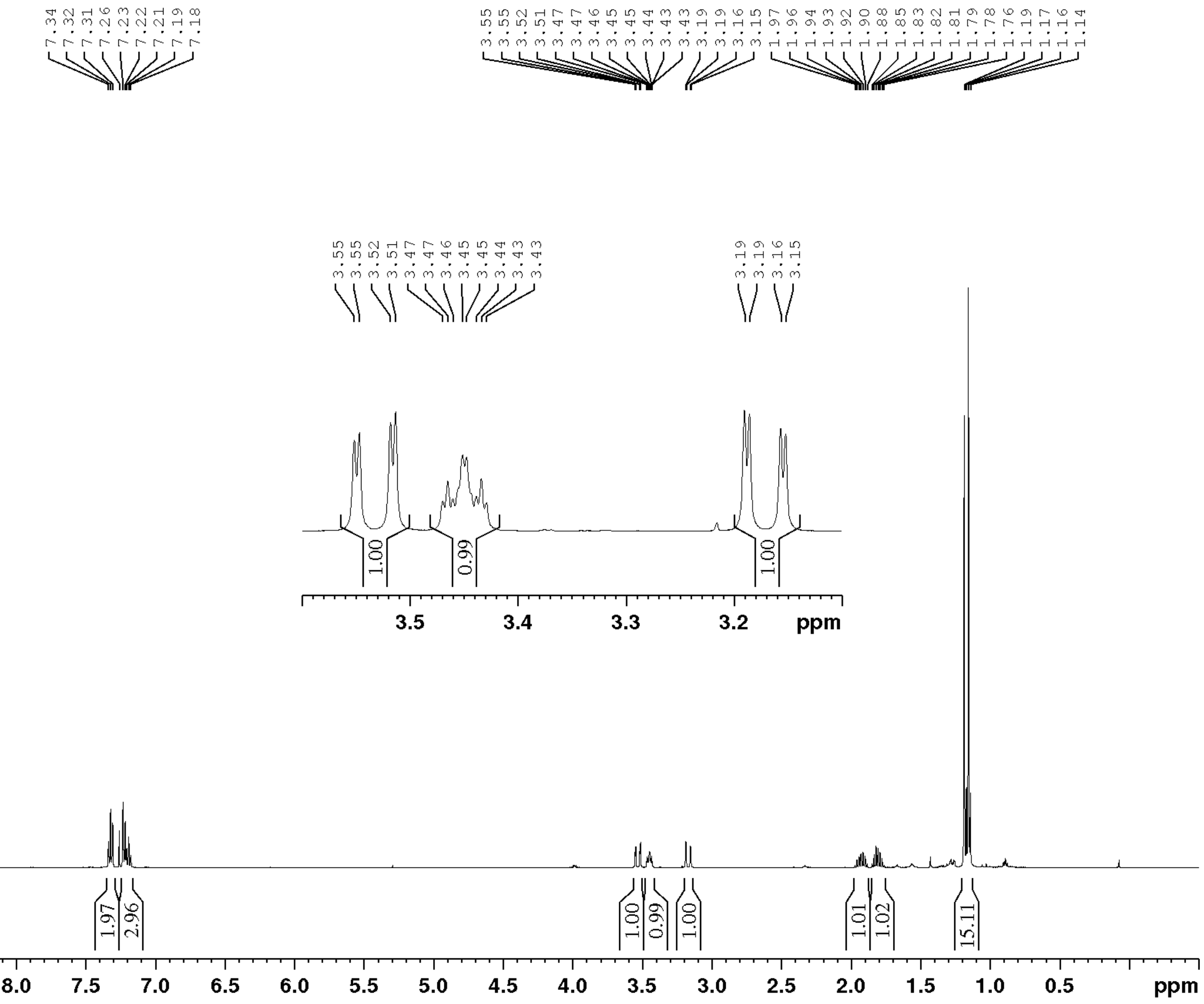

Figure S52. **$^{13}C\{^{1}H\}$ NMR** spectrum (126 MHz, $CDCl_3$, 298 K) of cyclobutylboronate **2s**.

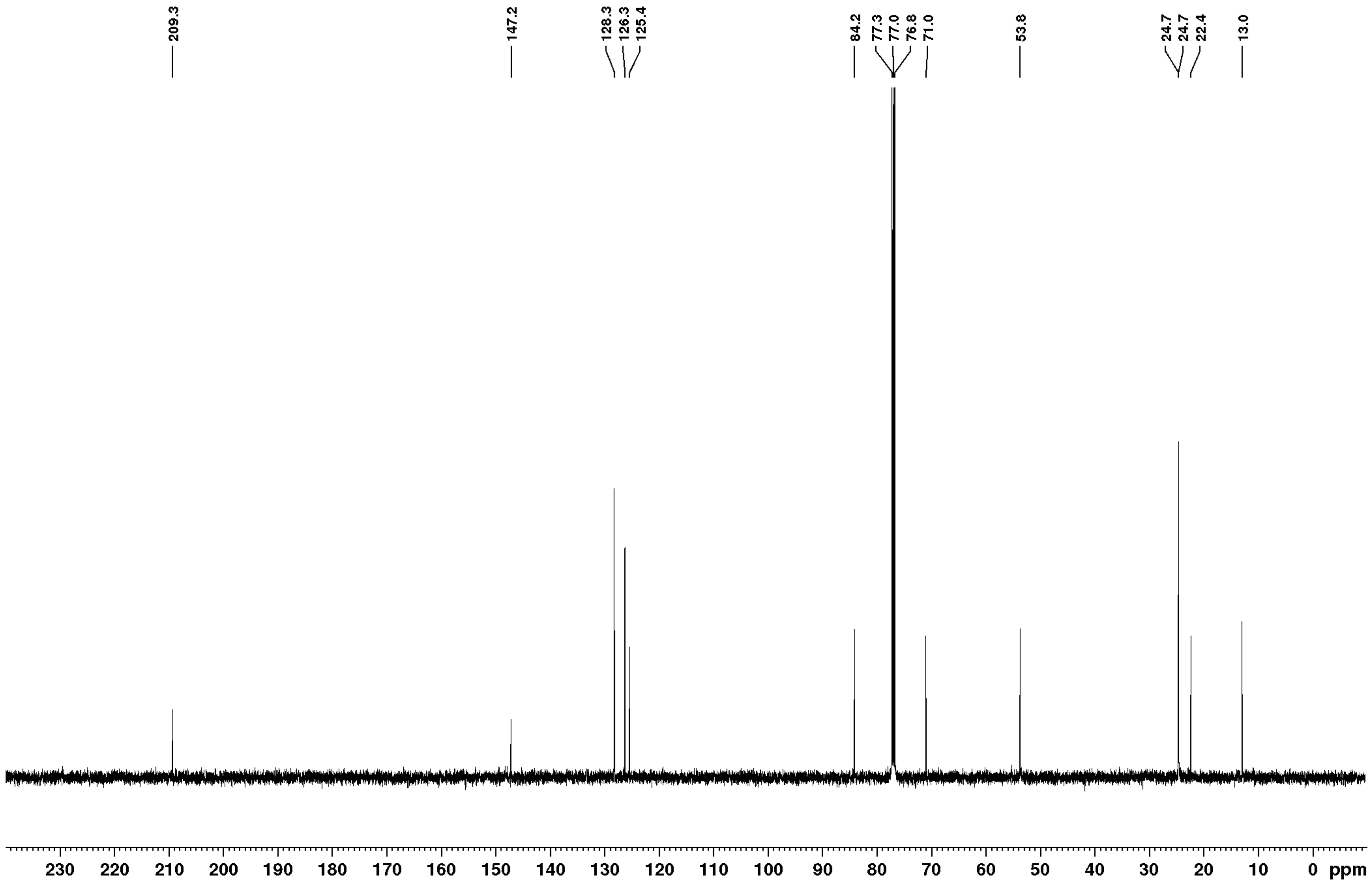

Figure S53. **^{1}H NMR** spectrum (500 MHz, $CDCl_3$, 298 K) of cyclobutylboronate **2t**.

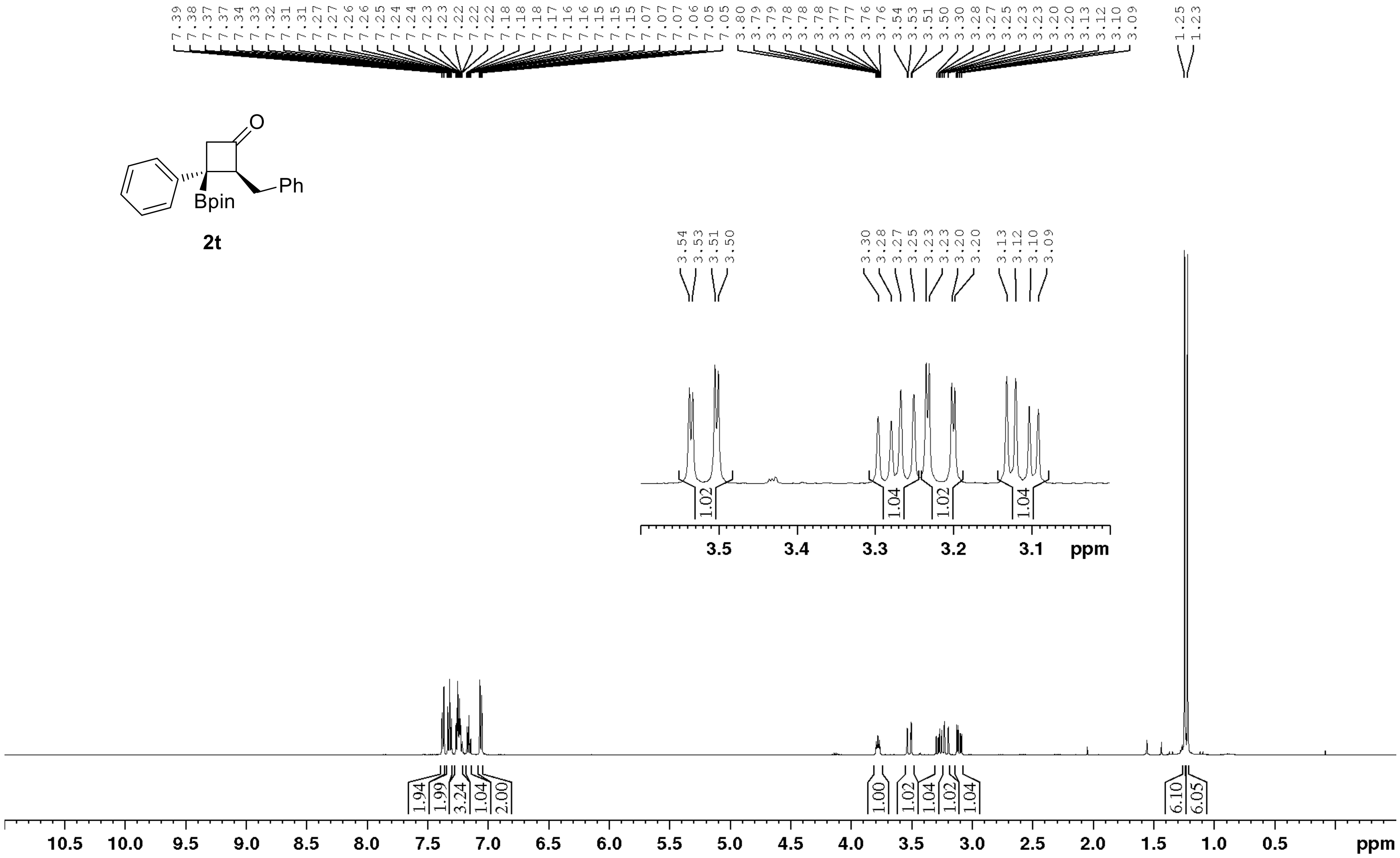

Figure S54. **$^{13}C\{^1H\}$ NMR** spectrum (126 MHz, $CDCl_3$, 298 K) of cyclobutylboronate **2t**.

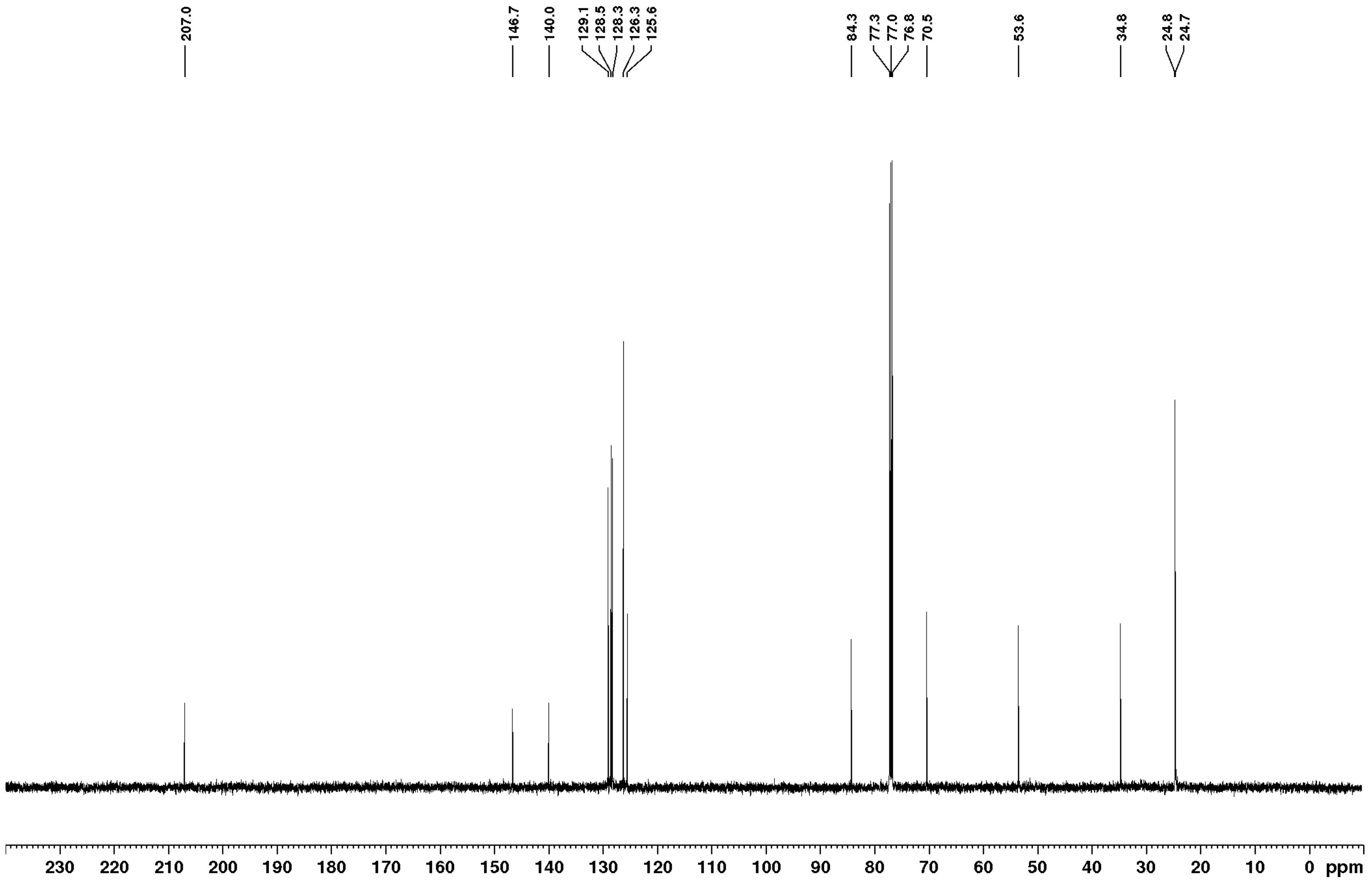

Figure S55. **^{1}H NMR** spectrum (400 MHz, $CDCl_3$, 298 K) of cyclobutylboronate **2u**.

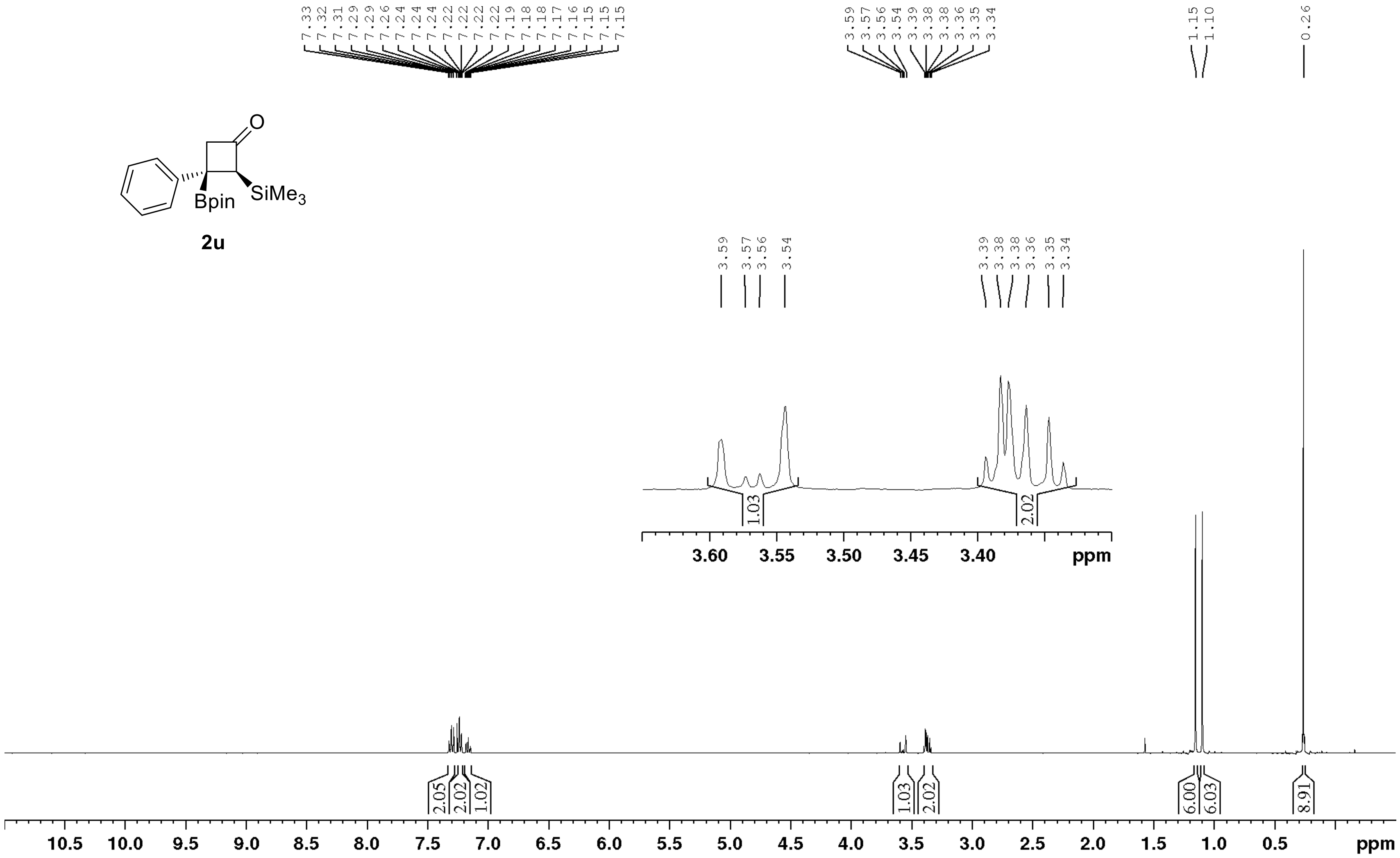

Figure S56. **$^{13}C\{^{1}H\}$ NMR** spectrum (101 MHz, $CDCl_3$, 298 K) of cyclobutylboronate **2u**.

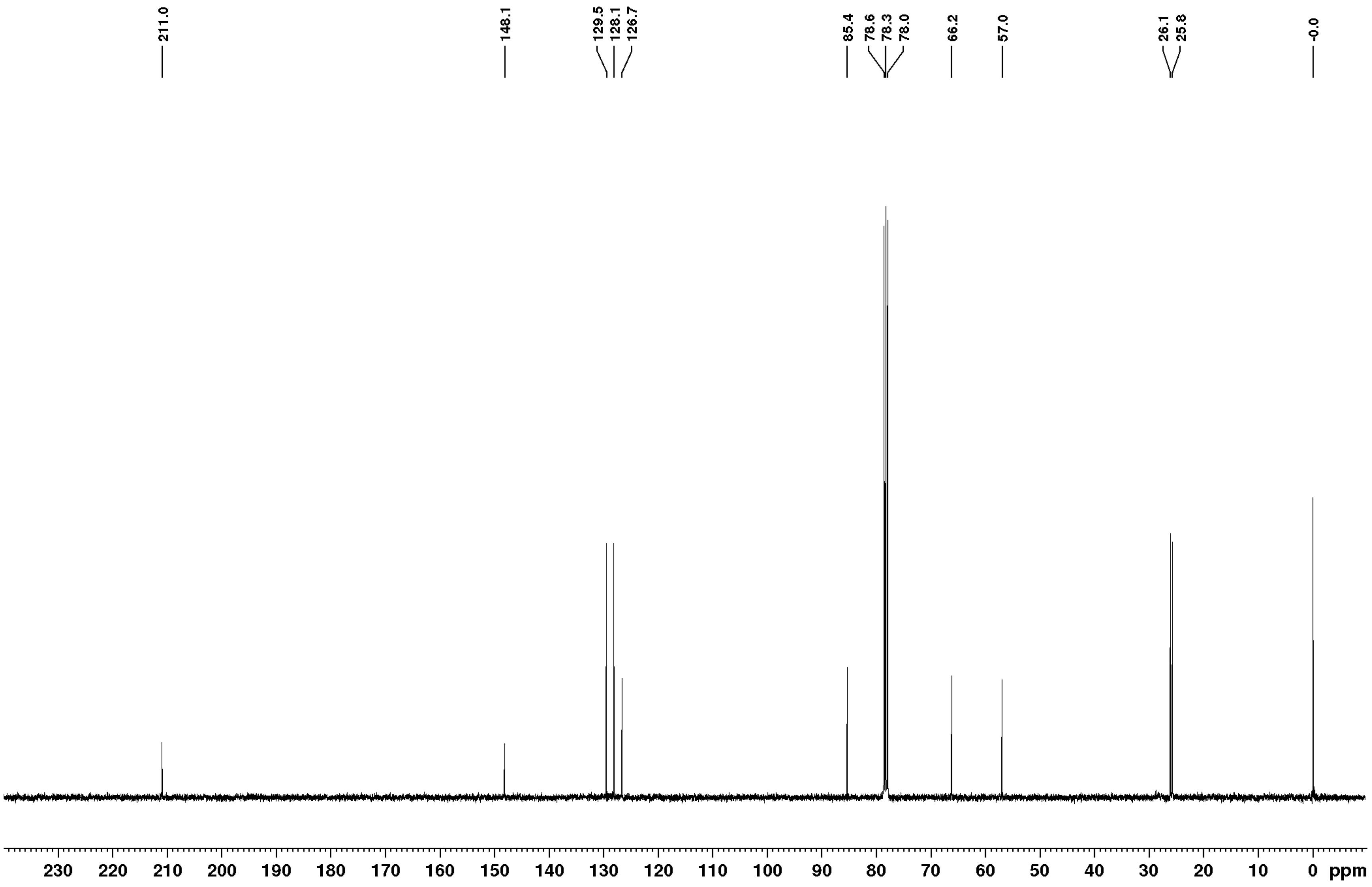

Figure S57. **^{29}Si{^{1}H} DEPT** NMR spectrum (99 MHz, $CDCl_3$, 298 K, optimized for *J* = 7 Hz) of cyclobutylboronate **2u**.

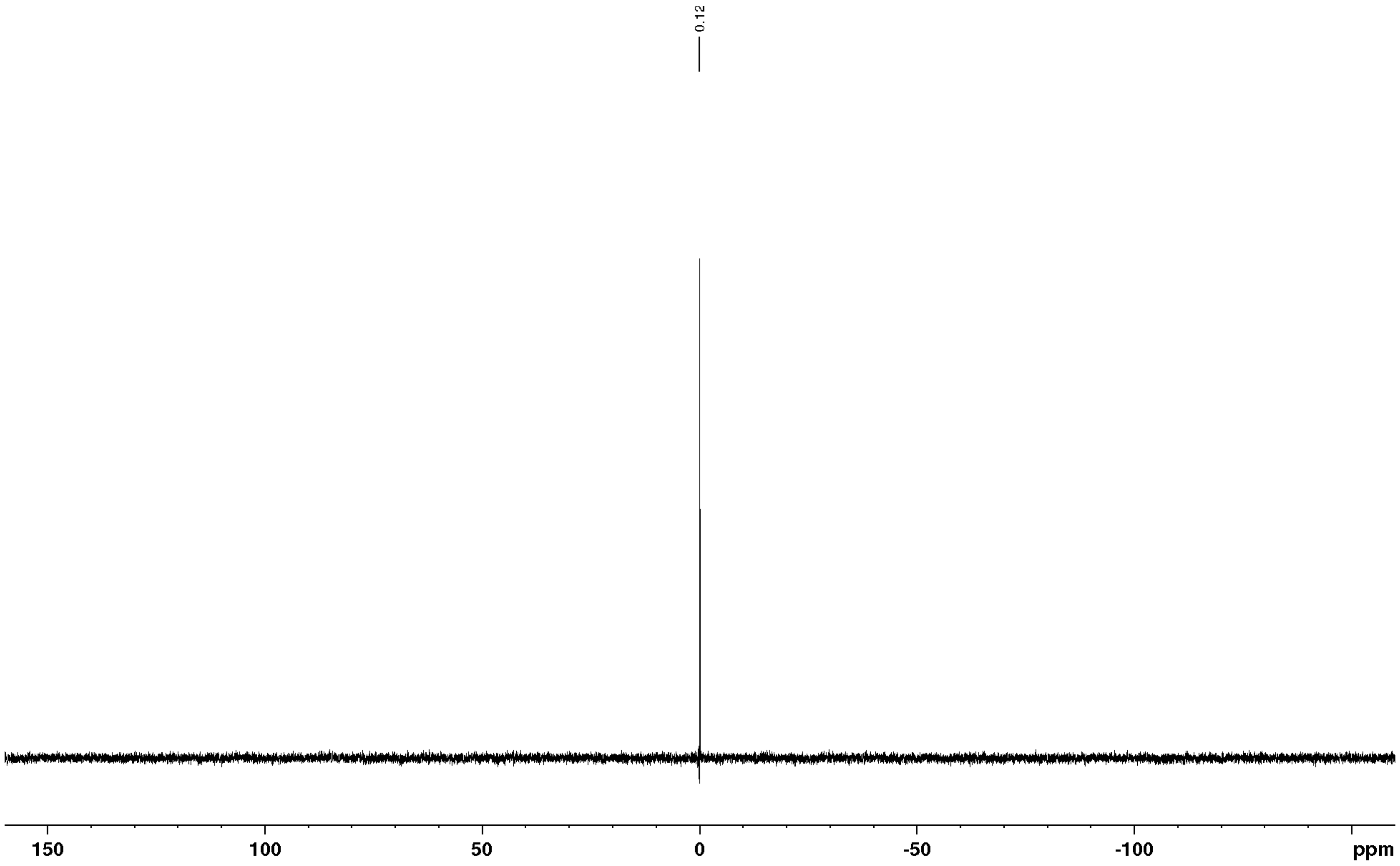

Figure S58. **^{1}H NMR** spectrum (500 MHz, $CDCl_3$, 298 K) of cyclobutylboronate **2v**.

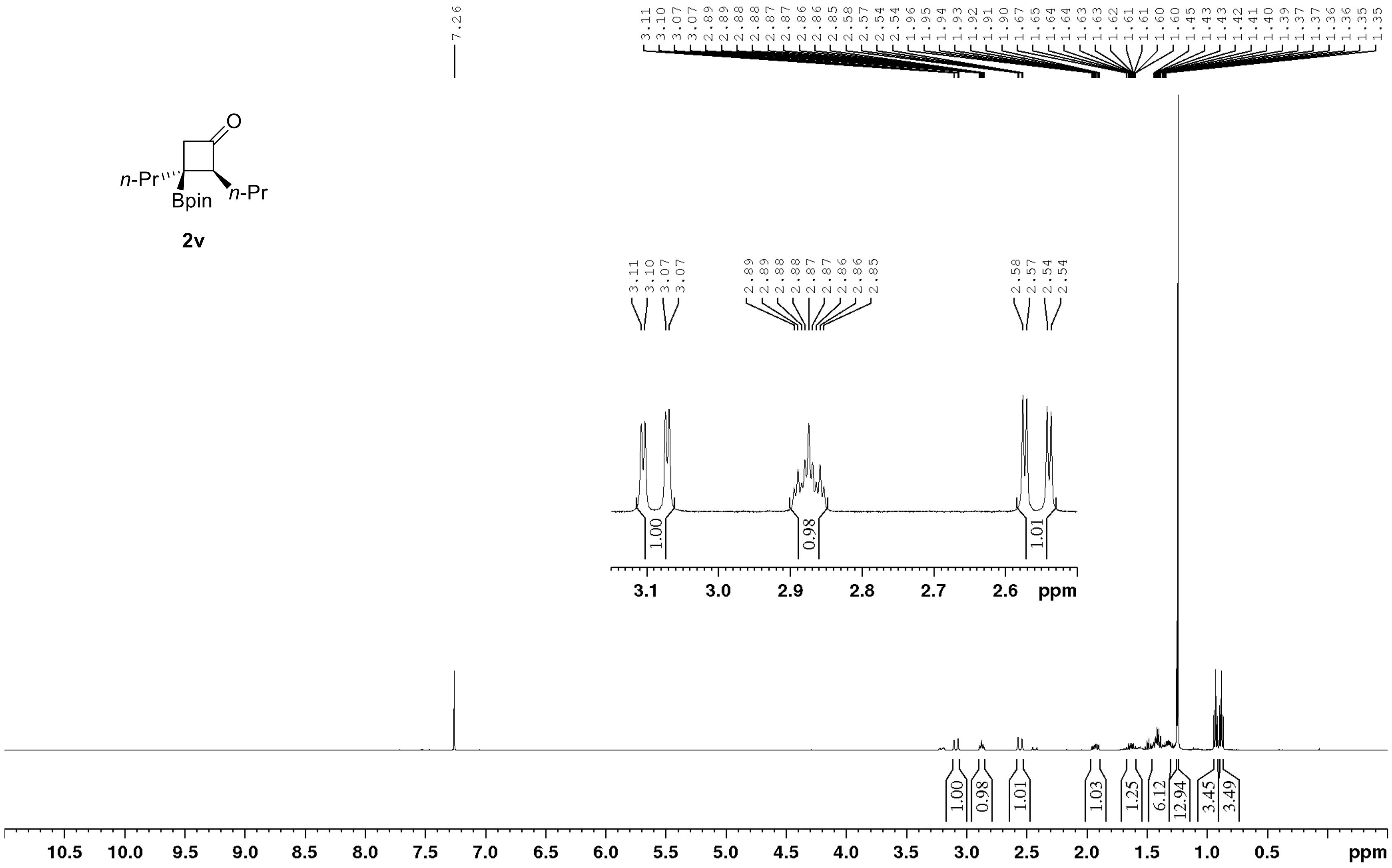

Figure S59. **$^{13}C\{^{1}H\}$ NMR** spectrum (126 MHz, $CDCl_3$, 298 K) of cyclobutylboronate **2v**.

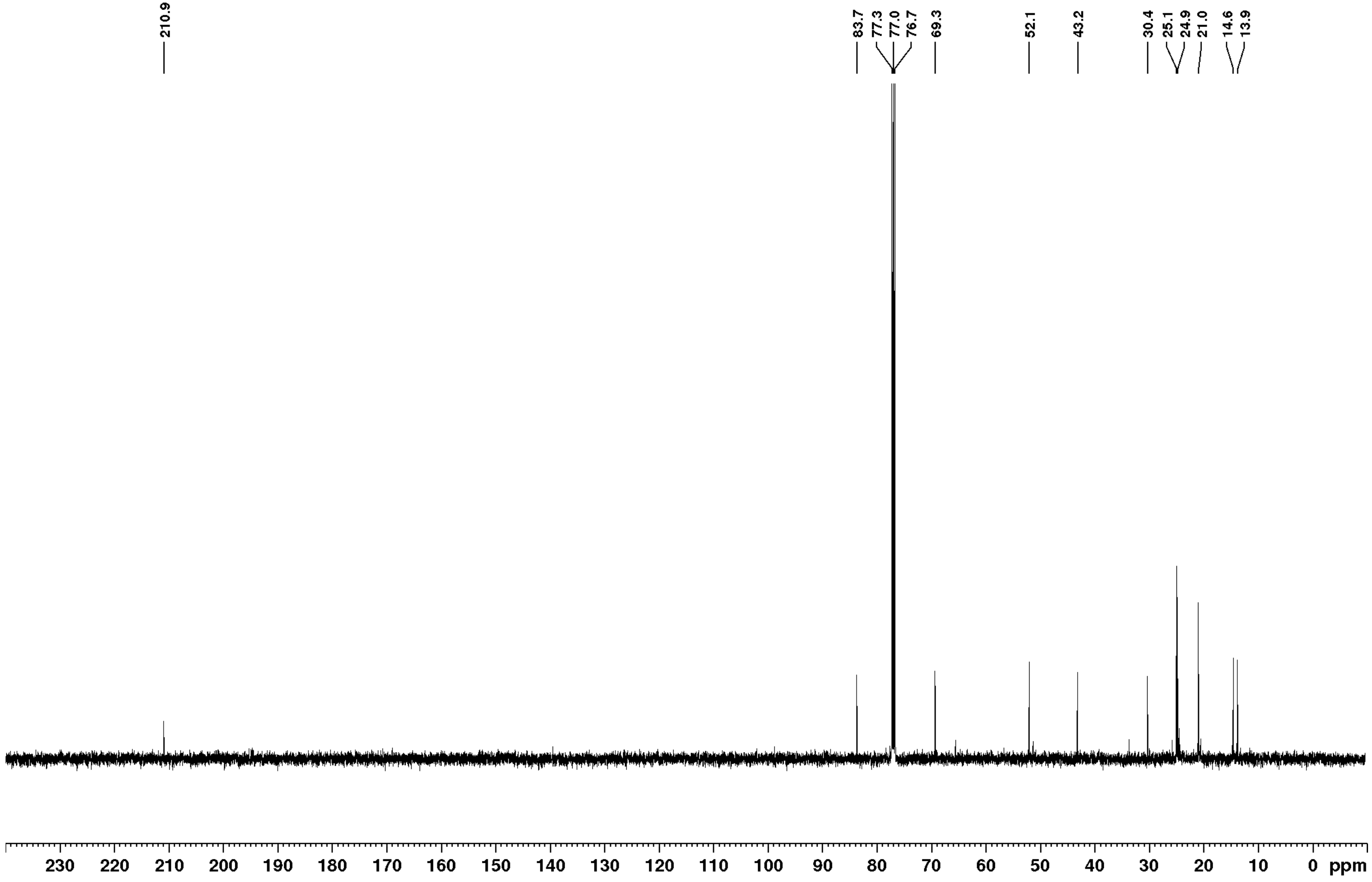

Figure S61. **^{1}H NMR** spectrum (400 MHz, $CDCl_3$, 298 K) of cyclobutylboronate **2w**.

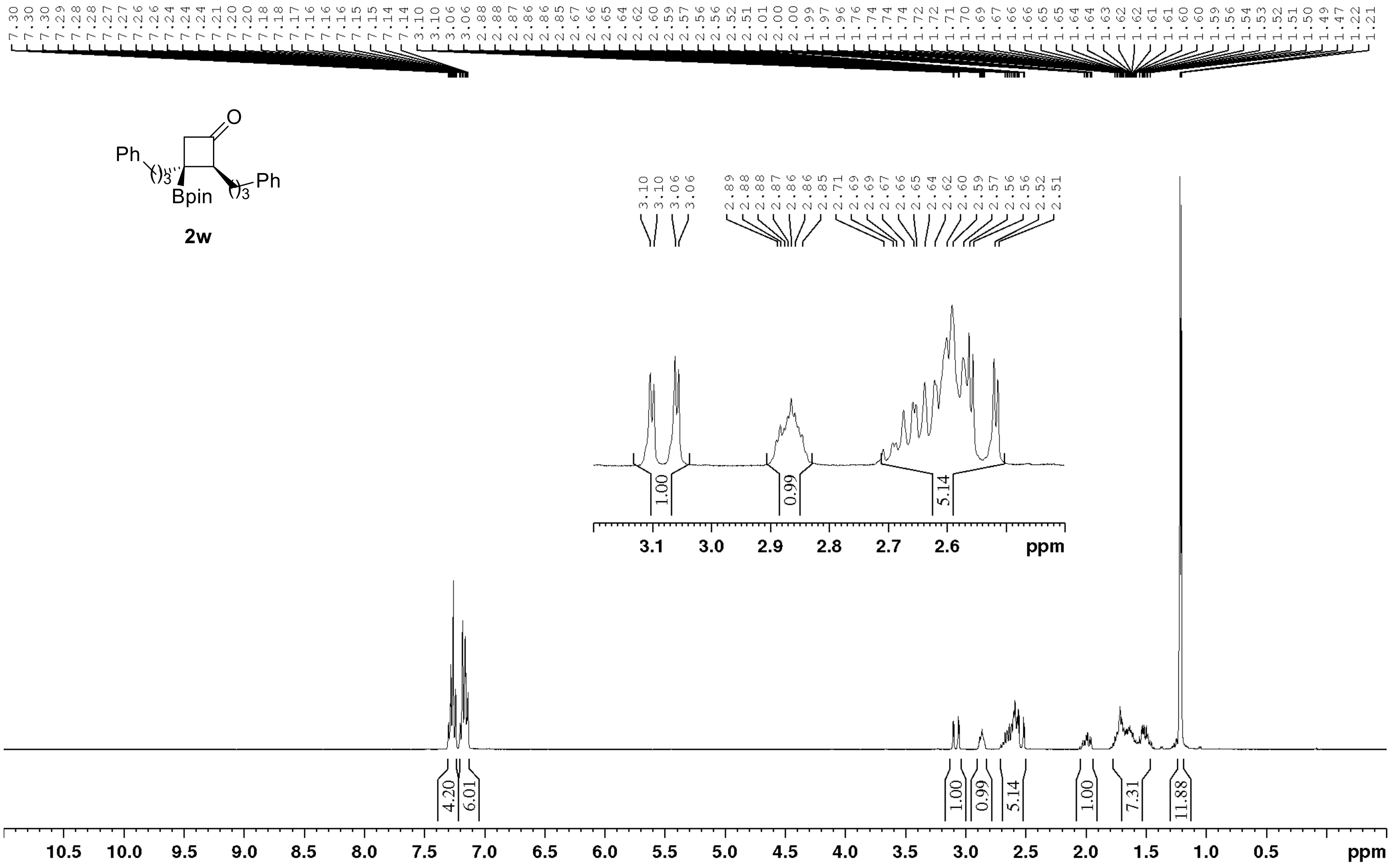

Figure S62. **$^{13}C\{^1H\}$ NMR** spectrum (101 MHz, $CDCl_3$, 298 K) of cyclobutylboronate **2w**.

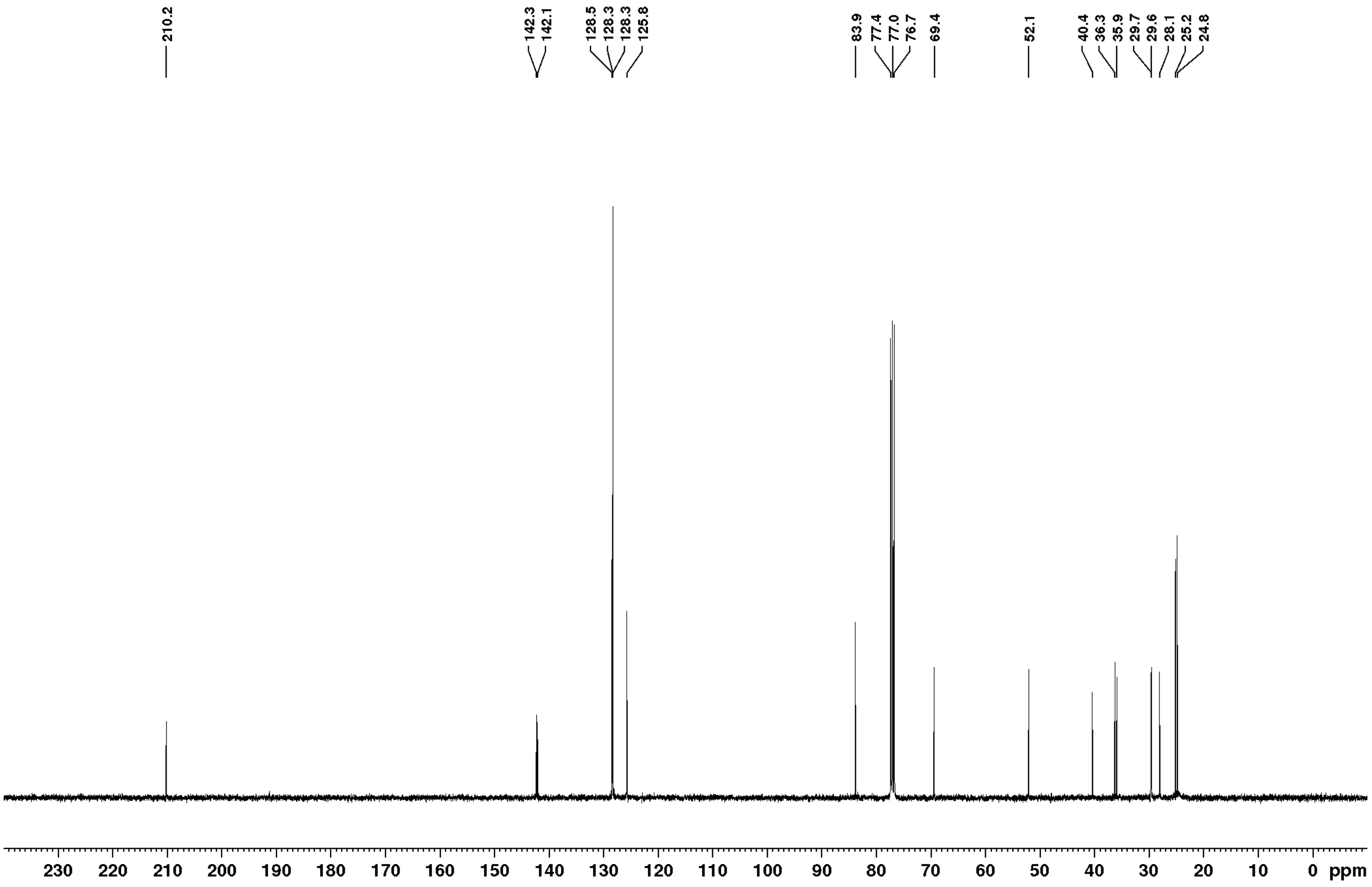

Figure S63. **^{1}H NMR** spectrum (500 MHz, $CDCl_3$, 298 K) of **5**.

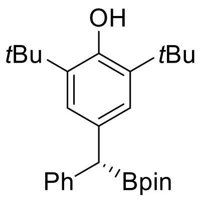

5

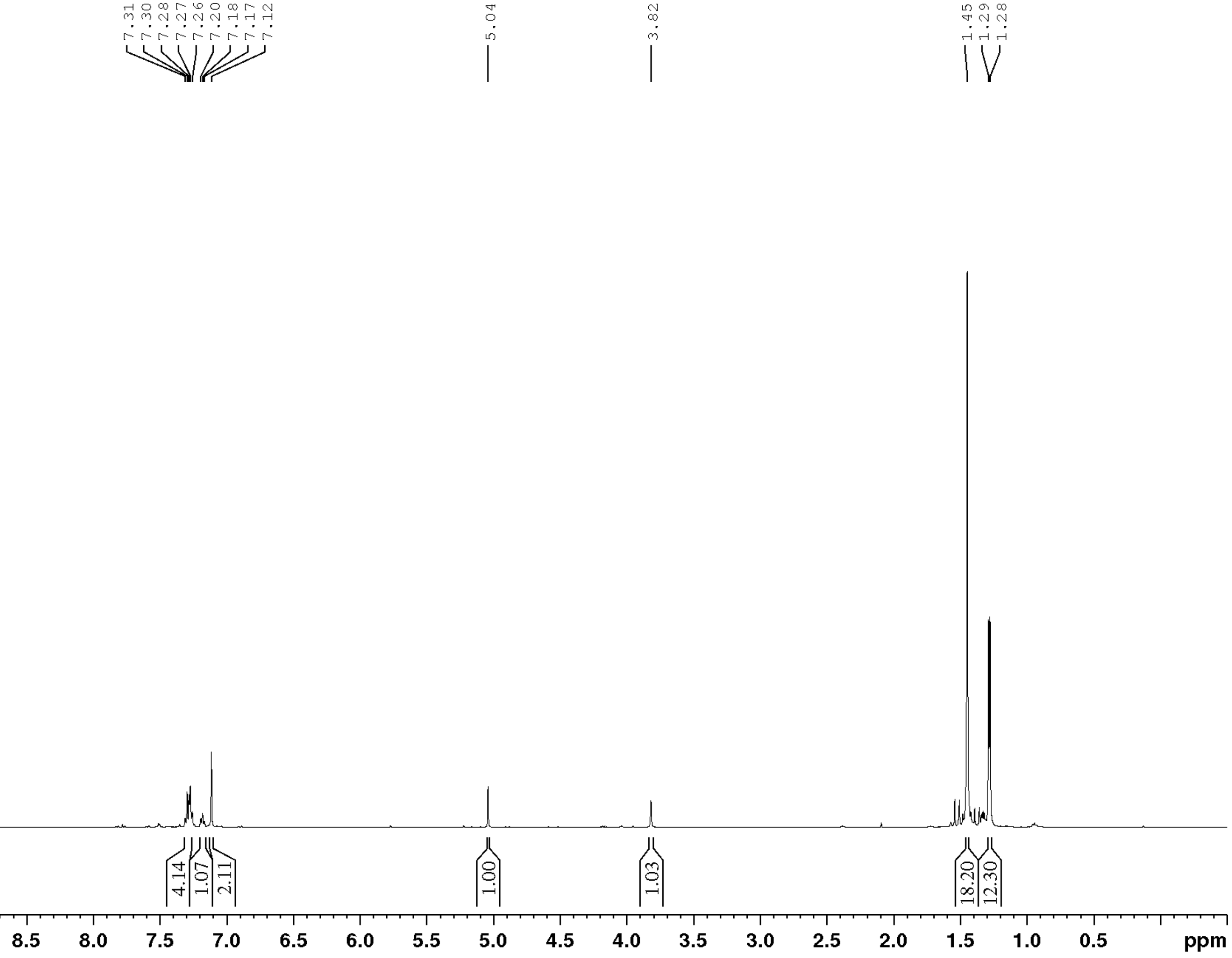

Figure S64. **$^{13}C\{^{1}H\}$ NMR** spectrum (126 MHz, $CDCl_3$, 298 K) of **5**.

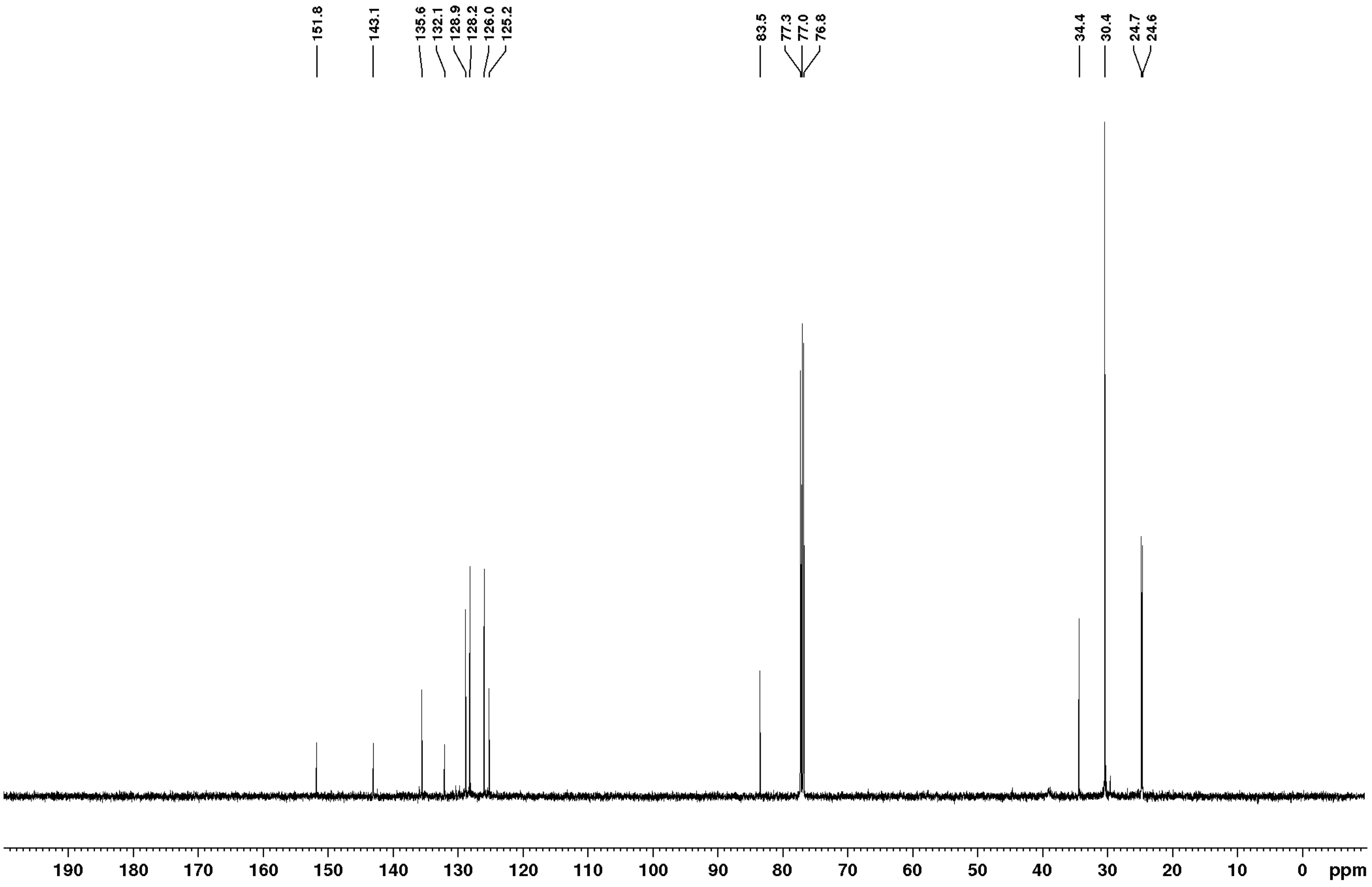

8 References

[S1] For the preparation of *rac*-Binap(O) **L5** and BPMOs **L1a(O)**, **L1b(O)**, **L1c(O)**, **L1c(O_2)**, **L2(O)**, **L3(O)**: see: a) *rac*-Binap(O): V. V. Grushin, *Organometallics* **2001**, *20*, 3950–3961; b) **L1a(O)**, **L1b(O)**, **L1c(O)**, **L2(O)**: J. Hu, H. Hirao, Y. Li, J. Zhou, *Angew. Chem. Int. Ed.* **2013**, *52*, 8676–8680; *Angew. Chem.* **2013**, *125,* 8838–8842; c) (*R*,*R*)-Quinoxp(O) **L3(O)**: H. Li, K. M. Belyk, J. Yin, Q. Chen, A. Hyde, Y. Ji, S. Oliver, M. T. Tudge, L.-C. Campeau, K. R. Campos, *J. Am. Chem. Soc.* **2015**, *137*, 13728–13731; d) (*S*)-xyl-Binap dioxide **L1c(O_2)**: T. Horibe, K. Nakagawa, T. Hazeyama, K. Takeda, K. Ishihara, *Chem. Commun.* **2019**, *55*, 13677–13680.

[S2] For the preparation of cyclobutenones **1a**–**t** and **1w**, see: a) H. A. Clement, M. Boghi, R. M. McDonald, L. Bernier, J. W. Coe, W. Farrell, C. J. Helal, M. R. Reese, N. W. Sach, J. C. Lee, D. G. Hall, *Angew. Chem. Int. Ed.* **2019**, *58*, 18405–18409; *Angew. Chem.* **2019**, *131*, 18576–18580; b) cyclobutenone **1u**: K. X. Rodriguez, N. Kaltwasser, T. A. Toni, B. L. Ashfeld, *Org. Lett.* **2017**, *19*, 2482–2485; c) cyclobutenone **1v**: R. L. Danheiser, S. Savariar, *Tetrahedron Lett.* **1987**, *28*, 3299–3302.

[S3] S.-L. Shi, S. L. Buchwald, *Nat. Chem.* **2015**, *7*, 38–44.

[S4] C. Jarava-Barrera, A. Parra, A. López, F. Cruz-Acosta, D. Collado-Sanz, D. J. Cárdenas, M. Tortosa, *ACS Catal.* **2016**, *6*, 442–446.
